# Unlocking radical reactivity of cyclic diaryl λ^3^-chloranes through NHC-catalyzed three-component coupling

**DOI:** 10.1039/d5sc09326k

**Published:** 2025-12-30

**Authors:** Anusree A. Kunhiraman, Koushik Patra, Venkata Surya Kumar Choutipalli, Manjeet Godara, Kevin L. Shuford, Mahiuddin Baidya

**Affiliations:** a Department of Chemistry, Indian Institute of Technology Madras Chennai 600036 India mbaidya@iitm.ac.in; b Department of Chemistry and Biochemistry, Baylor University One Bear Place #97348 Waco Texas 76798-7348 USA

## Abstract

Hypervalent halogens are central to contemporary organic synthesis, yet hypervalent chloranes, particularly cyclic λ^3^-chloranes, remain markedly underexplored, despite their unique electronic properties imparted by the highly electronegative chlorine atom. To date, their radical reactivity has not been documented. Herein, we report the first radical reaction of cyclic diaryl λ^3^-chloranes, enabled by N-heterocyclic carbene (NHC) catalysis in a three-component reaction with aromatic aldehydes and olefins at room temperature. This strategy leverages the strong reducing power of the NHC-derived Breslow enolate to generate a biaryl radical from λ^3^-chlorane, initiating a radical relay that culminates in regioselective vicinal aroylarylation of olefins. This transition-metal-free methodology provides streamlined access to *ortho*-substituted unsymmetrical biaryls in high yields, with broad functional group tolerance and compatibility with biorelevant scaffolds. Mechanistic insights from DFT calculations reveal that the key single-electron transfer (SET) from Breslow enolate to λ^3^-chlorane is a barrierless process, markedly distinct from that of the analogous λ^3^-bromane and λ^3^-iodane species. The favorable kinetics of the radical relay event and the thermodynamic stability of the aroylarylated products drive the reaction selectively along the desired three-component pathway.

## Abstract 2

The radical reactivity of cyclic diaryl λ^3^-chloranes under NHC catalysis is demonstrated through a three-component coupling of cyclic diaryl λ^3^-chloranes, aldehydes, and alkenes, enabling synthesis of a range of unsymmetrical biaryls in high yields.

## Graphical abstract

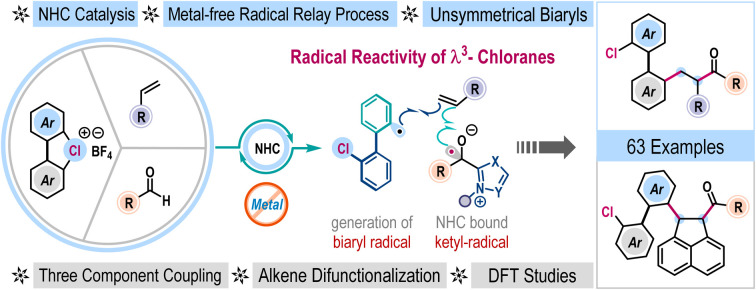

## Introduction

**Scheme 1:**
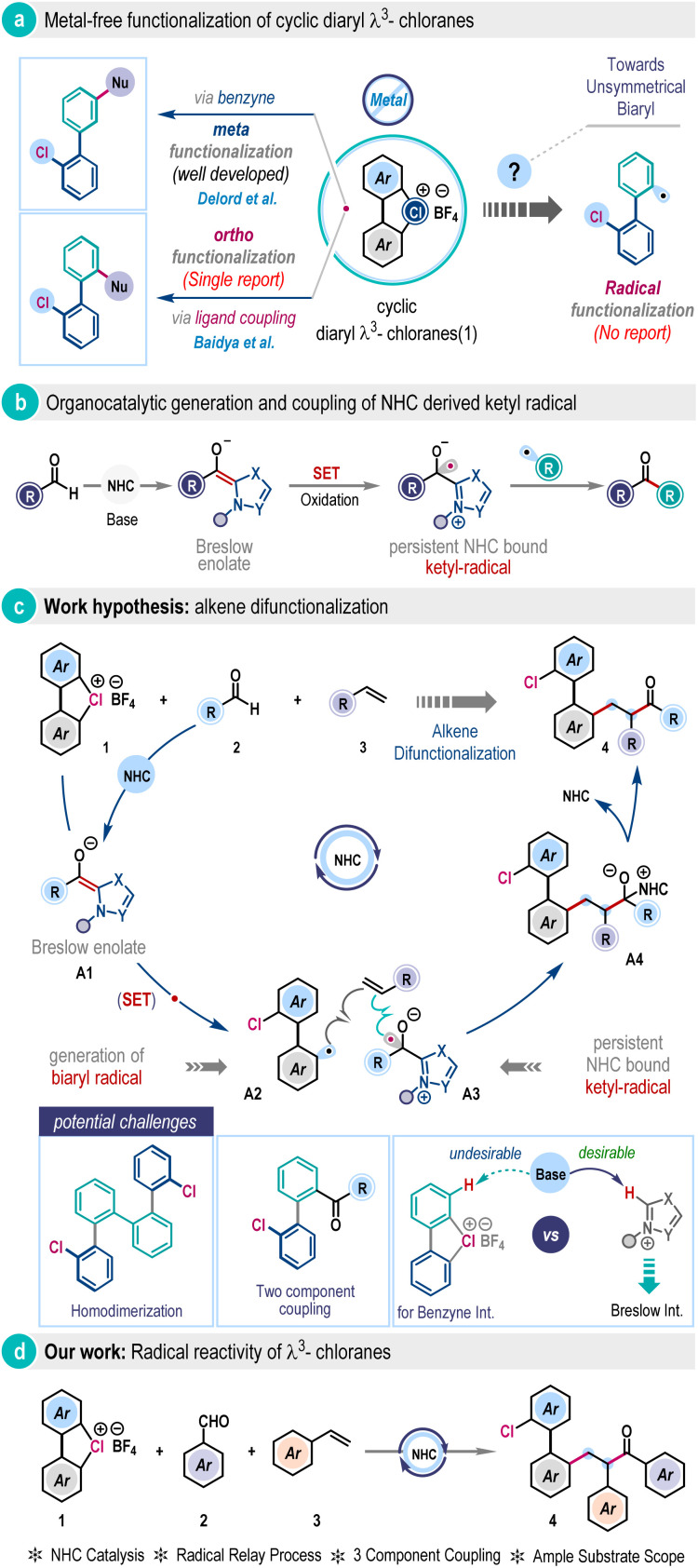
Cyclic diaryl λ^3^-chloranes and NHC radical catalysis.

The chemistry of hypervalent halogens has become a cornerstone in modern organic synthesis, offering innovative pathways for creating complex molecules under mild conditions.^1^ Their unique electronic structure and properties, coupled with low toxicity, contribute to distinct reactivity that often complements the mechanistic rationale of transition metal catalysis, amplifying their relevance in chemical science.^1,2^ Over the years, major advancements in this field have largely been driven by the development of λ^3^-iodanes and λ^3^-bromanes.^2,3^ Surprisingly, progress of their isoelectronic congener, λ^3^-chloranes, remains immature, albeit they could exhibit increased reactivity owing to the higher electronegativity and ionization potential of chlorine compared to iodine and bromine.^4^ A breakthrough was achieved recently with the introduction of cyclic diaryl λ^3^-chloranes (1) from the Wencel–Delord group (Scheme 1a).^5^ They have manifested the elevated nucleofugality property of 1 leading to the formation of a benzyne intermediate under basic conditions, which was then trapped by nucleophiles to expedite steric-effect governed preferential *meta*-functionalization (Scheme 1a). Our research group also leveraged nucleophile capture reactivity of 1 and disclosed the highly *ortho*-selective ligand coupling reactivity under metal-free conditions (Scheme 1a).^6^ However, these methodologies primarily reflect polar chemistry, the classical two-electron reaction pathway of λ^3^-chlorane (1). At this juncture, the one-electron reaction pathway, the so-called radical reaction modality, of 1 under metal-free conditions remains largely unexplored. Meanwhile, the thermal rearrangement of µ-sulfoxo diaryl cyclic λ^3^-chloranes to homodimerized biaryls was recently observed by the Wirth group, where a radical reaction pathway was proposed.^7^ Obviously, the successful realization of radical reactivity in versatile cyclic diaryl λ^3^-chloranes (1) beyond the classical polar mechanism, particularly in a multicomponent fashion, holds the potential to open new avenues in contemporary organic synthesis.

The persistent ketyl radicals have a rich history.^8^ Recently, they have been judiciously integrated within N-heterocyclic carbene (NHC) catalysis to harness NHC radical catalysis.^9^ This reactivity takes advantage of the strong reducing power of the Breslow enolate intermediate, which undergoes single electron transfer (SET) to generate the persistent ketyl radical for subsequent functionalization (Scheme 1b).^9,10^ In this context, the pioneering contribution from the Ohmiya group on the decarboxylative coupling of aryl aldehydes and redox-active esters to produce sterically congested ketone^11*l*^ and further advancements by others are highly intriguing.^11^ We question whether cyclic diaryl λ^3^-chloranes could be engaged as SET reagents in NHC radical catalysis to access biaryl radicals, which is so far elusive. We envisioned a three-component coupling involving λ^3^-chlorane (1), aldehyde (2), and alkene (3) (Scheme 1c). Strategically, the enolate form of the Breslow intermediate A1, generated through the reaction of aldehyde (2) with NHC, could potentially affect SET to the cyclic diaryl λ^3^-chlorane (1), exploiting the oxidizing power of λ^3^-chlorane and rapid carbon–chlorine bond cleavage to generate the pivotal biaryl radical A2 and NHC-bound ketyl-radical A3 (Scheme 1c). Then, they induce a radical relay process with alkene (3) to give intermediate A4, which would subsequently break down to release biaryl-embedded alkene difunctionalization product 4 with the regeneration of the NHC catalyst (Scheme 1c). However, significant challenges persist in mitigating the formation of homo-coupling^7^ and two-component byproducts, as well as in controlling regioselectivity. Furthermore, NHC catalysis operates under basic conditions that inherently favor the benzyne pathway,^5^ as discussed in the preceding section, which must be effectively suppressed to unlock the desired radical reactivity of λ^3^-chloranes (Scheme 1c, below).

Herein, we report the development of this approach and delineate the first example of the radical reactivity of cyclic diaryl λ^3^-chloranes through N-heterocyclic carbene (NHC) catalysis (Scheme 1d). This methodology capitalizes on the metal-free coupling of cyclic diaryl λ^3^-chloranes, aldehydes, and alkenes to offer a wide range of functionally enriched unsymmetrically *ortho*-disubstituted biaryls in high yields at room temperature. In a nutshell, it regioselectively installs an aroyl group and a biaryl unit across the olefin functionality in a single operation. This catalytic aroylarylation protocol is operationally simple, scalable, and applicable to a wide variety of substrates, including those relevant to pharmaceuticals and materials. DFT calculations were also performed to elucidate the intricacy in the reaction mechanism.

## Results and discussion

Our investigations began following the three-component coupling of cyclic diaryl λ^3^-chlorane 1a, 4-bromobenzaldehyde 2a, and styrene 3a as a model reaction (Table 1). Initially, we screened various solvents using thiazolium salt N1 as the NHC precursor in the presence of Cs_2_CO_3_ base (entries 1 and 2). However, most solvents were ineffective, leading primarily to the decomposition of λ^3^-chlorane 1a. A breakthrough was achieved when the reaction was conducted in DMF, which produced 4a, albeit in a modest 32% yield (entry 3). Notably, when DMSO was used as the solvent, the reaction proceeded cleanly, offering the desired product 4a in 86% isolated yield (entry 4). Employment of other thiazolium salts revealed a moderate reactivity for N2, while the reaction was unfruitful with the triazolium-based NHC precursor N3 (entry 5). Examination of other inorganic bases showed that KO^*t*^Bu was ineffective; however, K_2_CO_3_ and Na_2_CO_3_ promote this reaction, giving 4a in 70% and 63% yields, respectively (entry 6). Detrimental outcomes were also obtained for organic bases such as Et_3_N or DMAP (entry 7). The loading of reaction components was crucial for achieving high yields. Variations in the amounts of 1a or 3a resulted in suboptimal outcomes (entries 8–9). Additionally, reducing the catalyst loading to 20 mol% lowered the yield to 62%, and the reaction completely failed in the absence of the NHC catalyst (entries 10–11). Also, this NHC catalyzed SET process was not effective with the biaryl diazonium precursor 1-N_2_BF_4_, indicating the significance of cyclic diaryl λ^3^-chlorane for this coupling reaction (entry 12).

**Table 1:** Optimization of reaction conditions^a^^b^^c^

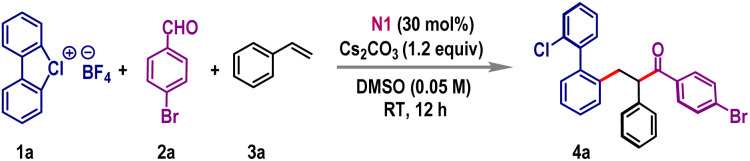		
Entry	Deviation from the standard conditions	Yield of 4a (%)^b^
1	DCE/CH_3_CN/TFT instead of DMSO	NR
2	1,4-Dioxane/DCM instead of DMSO	NR
3	DMF instead of DMSO	32
4	None	86
5	With N2/N3 instead of N1	41/NR
6	KO^*t*^Bu/Na_2_CO_3_/K_2_CO_3_ instead of Cs_2_CO_3_	NR/63/70
7	Et_3_N/DMAP instead of Cs_2_CO_3_	34/NR
8	1.0/2.0 equiv. of 1a instead of 1.5 equiv.	50/67
9	1.5/3.0 equiv. of 3a instead of 2.0 equiv.	72/80
10	With 20 mol% of N1	62
11	Without N1	NR
12	With 1-N_2_BF_4_ instead of 1a	NR
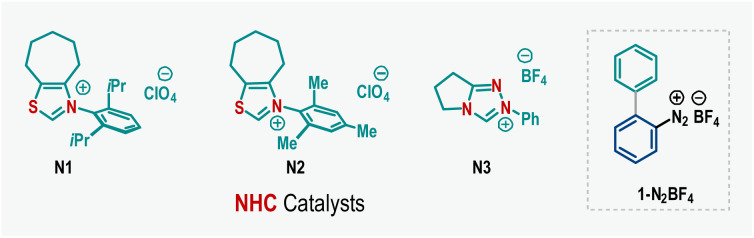		

a Reaction conditions: 1a (0.3 mmol), 2a (0.2 mmol), 3a (0.4 mmol), Cs_2_CO_3_ (1.2 equiv.), NHC (0.06 mmol) and solvent (4.0 mL), 12 h.

b Isolated yields are given.

c In all cases, 1 : 1 *dr* was obtained. NR: no reaction.

**Scheme 2:**
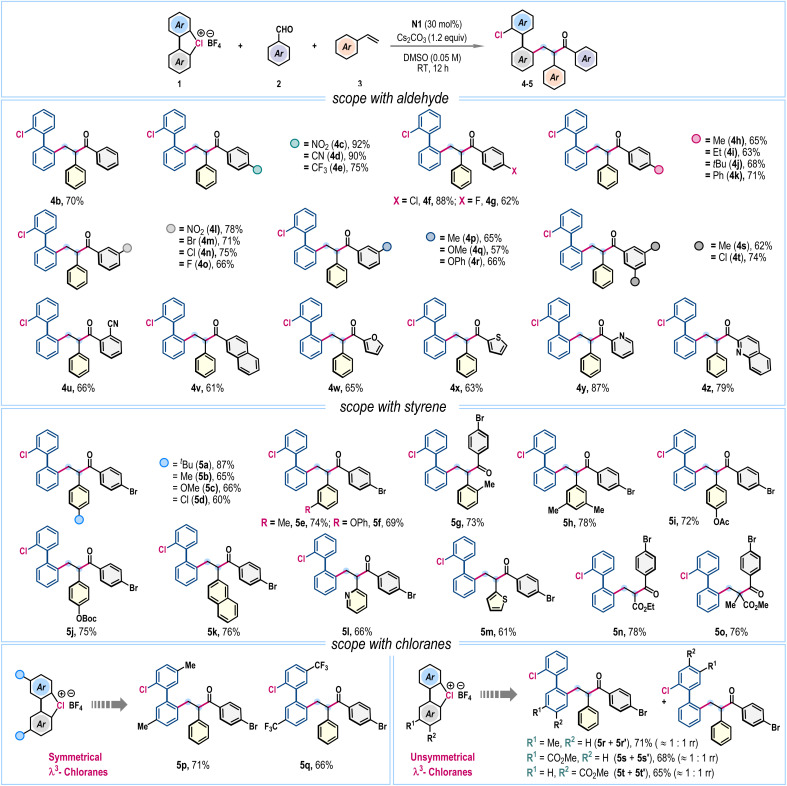
Exploration of substrate scope^*a,b,c*^. ^a^Reaction conditions: 1 (0.3 mmol), 2 (0.2 mmol), 3 (0.4 mmol), N1 (30 mol%), Cs_2_CO_3_ (1.2 equiv.), and DMSO (4.0 mL), rt, 12 h. ^b^Isolated yields are provided. ^c^≈1 : 1 *dr* was obtained for all cases.

After identifying the optimized reaction conditions (Table 1, entry 4), we turned our attention to exploring the scope of the three-component radical coupling reaction (Scheme 2). First, the influence of substitutions in aldehyde (2) was evaluated. Satisfyingly, along with parent benzaldehyde (4b), a wide range of aromatic aldehydes bearing electron-withdrawing nitro (4c), cyano (4d), trifluoromethyl (4e), and halogen (4f, 4g) functionalities and electron-donating alkyl (4h–4j) and phenyl (4k) groups at the *para*-position of the arene ring effectively participated, dispensing functionally enriched unsymmetrical 2,2′-disubstituted biphenyls in good to very high yields. Similarly, *meta*-substituted aromatic aldehydes smoothly furnished desired products 4l–4t in good yields. Sterically hindered *ortho*-substitution (4u) and bulky β-naphthaldehyde (4v) were also amenable. Importantly, aldehydes bearing heteroaromatic scaffolds such as furan (4w), thiophene (4x), pyridine (4y), and quinoline (4z) did not hamper the reaction, affording the corresponding products in 63–87% yields (Scheme 2). However, examination of aliphatic aldehydes under the standard reaction conditions was unsuccessful (SI, page S5).

Next, the compatibility of the styrene coupling partner (3) was examined, which also proved to be quite general (Scheme 2). Styrenes with alkyl, alkoxy, aryloxy, and halogen functionalities at various positions in the phenyl unit were suitable, forming the desired products 5a–5h in 60–87% yields. Common protecting groups such as acetyl and *tert*-butyloxycarbonyl (Boc) were also undisturbed to give 5i and 5j in 72% and 75% yields, respectively. Also, 2-vinylnaphthalene (5k), 2-vinylpyridine (5l), and 2-vinylthiophene (5m) were amenable to afford good yields. Significantly, three-component coupling was effective with electron-deficient alkenyl esters, for example, ethyl acrylate and methyl methacrylate, producing 5n and 5o in 78% and 76% yields, respectively (Scheme 2).

Furthermore, we explored the effect of different substitutions on cyclic diaryl λ^3^-chloranes (1). Symmetrically substituted λ^3^-chloranes, bearing electron-donating methyl and electron-withdrawing trifluoromethyl groups, gave unsymmetrical biaryls 5p and 5q in 71% and 66% yields, respectively (Scheme 2). When unsymmetrically substituted cyclic diaryl λ^3^-chloranes were employed, an inseparable mixture of regioisomers was obtained, attributed to radical functionalization occurring at both aromatic rings of the unsymmetrical λ^3^-chloranes (Scheme 2). A nearly 1 : 1 ratio of regioisomeric products was observed for substrates bearing either electron-donating (5r and 5r′) or electron-withdrawing (5s and 5s′; 5t and 5t′) substituents, suggesting that electronic effects play only a minor role in this radical functionalization process. Notably, this outcome contrasts sharply with our previous findings in ligand-coupling reactions, where functionalization preferentially occurred at the electron-deficient arene ring of unsymmetrical λ^3^-chloranes.^6^

**Scheme 3:**
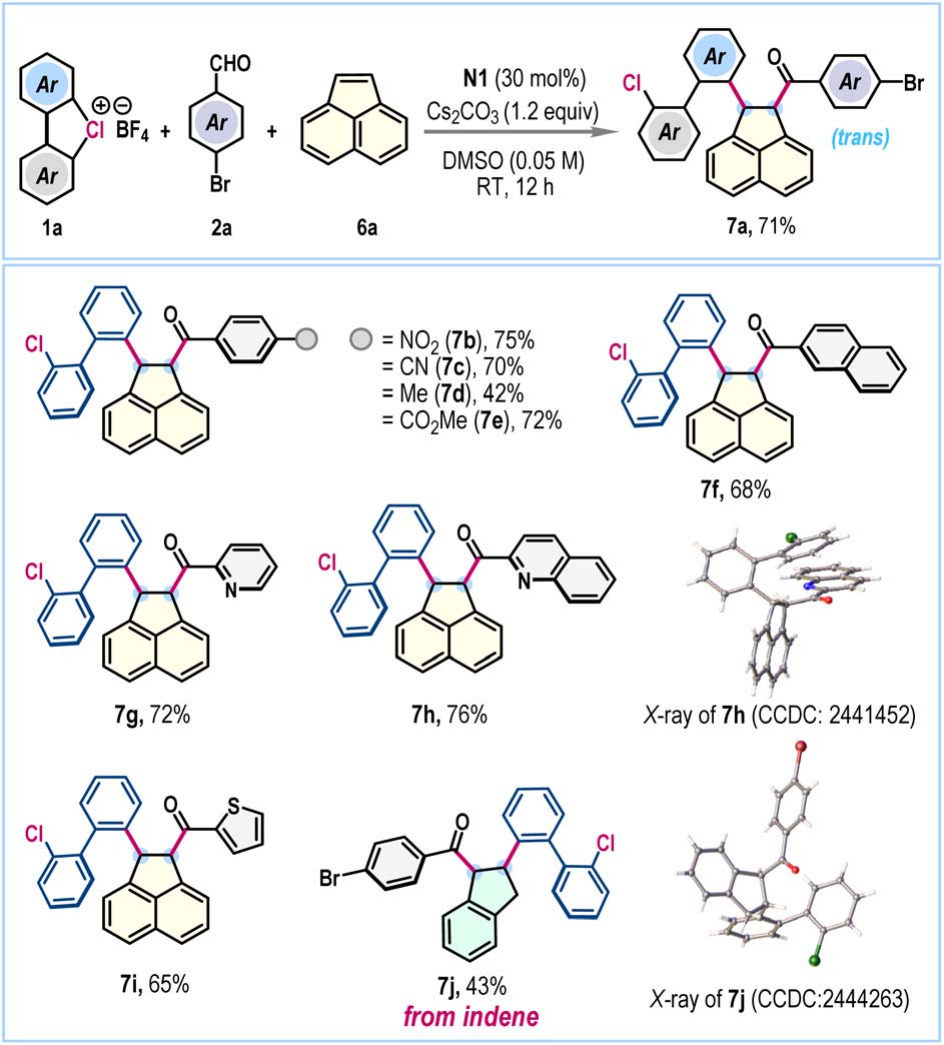
Towards difunctionalization of acenaphthylene and indene^a^. ^a^Reaction conditions: 1a (0.3 mmol), 2a (0.2 mmol), 6a (0.4 mmol), N1 (30 mol%), Cs_2_CO_3_ (1.2 equiv.), and DMSO (0.05 M), rt, 12 h. Isolated yields are provided and in all cases ≈1 : 1 *dr* was obtained.

To further augment the versatility of this NHC-catalyzed three-component radical coupling protocol, we considered the difunctionalization of acenaphthylene, a renowned scaffold in materials science.^12^ Gratifyingly, under the standard reaction conditions, the coupling of λ^3^-chlorane 1a, 4-bromobenzaldehyde 2a, and acenaphthylene 6a proceeded effectively to furnish the desired difunctionalized 1,2-dihydroacenaphthylene 7a in 71% yield (Scheme 3). We were pleased to observe exclusive *trans*-selectivity for the two newly installed functionalities. The protocol exhibited success across a range of aromatic and heteroaromatic aldehydes, facilitating the creation of a concise library of valuable 1,2-dihydroacenaphthylenes 7b–7i, generally obtained in good yields (Scheme 3). The compound 7h was crystalized and the single crystal X-ray analysis unambiguously confirmed both the product's structure and its stereochemistry. The reaction conditions were also effective for the difunctionalization of indene, successfully producing 7j in a synthetically useful yield without compromising *trans*-selectivity (Scheme 3).

**Scheme 4:**
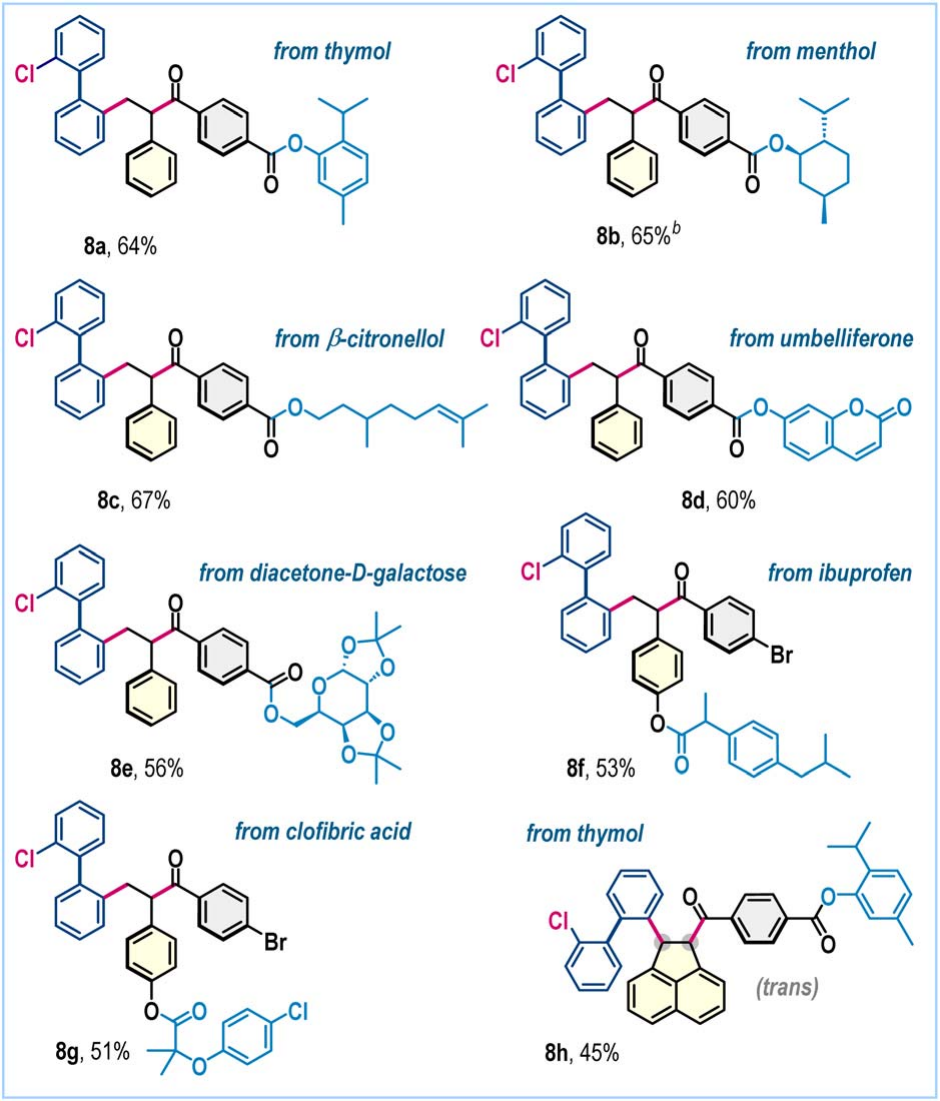
Three-component radical coupling with biorelevant scaffolds.^*a* a^Reaction conditions: as in Scheme 2. Isolated yields are provided and ≈1 : 1 *dr* was obtained. ^b^For 8b a *dr* of 1 : 1 : 1 : 1 was obtained.

To underscore the broad applicability and accommodate increased structural complexity within this three-component radical functionalization, substrates featuring biologically relevant scaffolds were investigated (Scheme 4). Notably, aldehydes derived from diverse bioactive frameworks, such as thymol (8a), (*L*)-menthol (8b), β-citronellol (8c), umbelliferone (8d), and diacetone-d-galactose (8e), underwent the transformation smoothly, affording the desired functionally enriched products in good yields. Likewise, styrenes bearing pharmacologically relevant motifs, including those derived from ibuprofen and clofibric acid, furnished products 8f and 8g in 53% and 51% yields, respectively (Scheme 4). With acenaphthylene, the aldehyde derived from thymol gave the desired product 8h in 45% yield (Scheme 4).

**Scheme 5:**
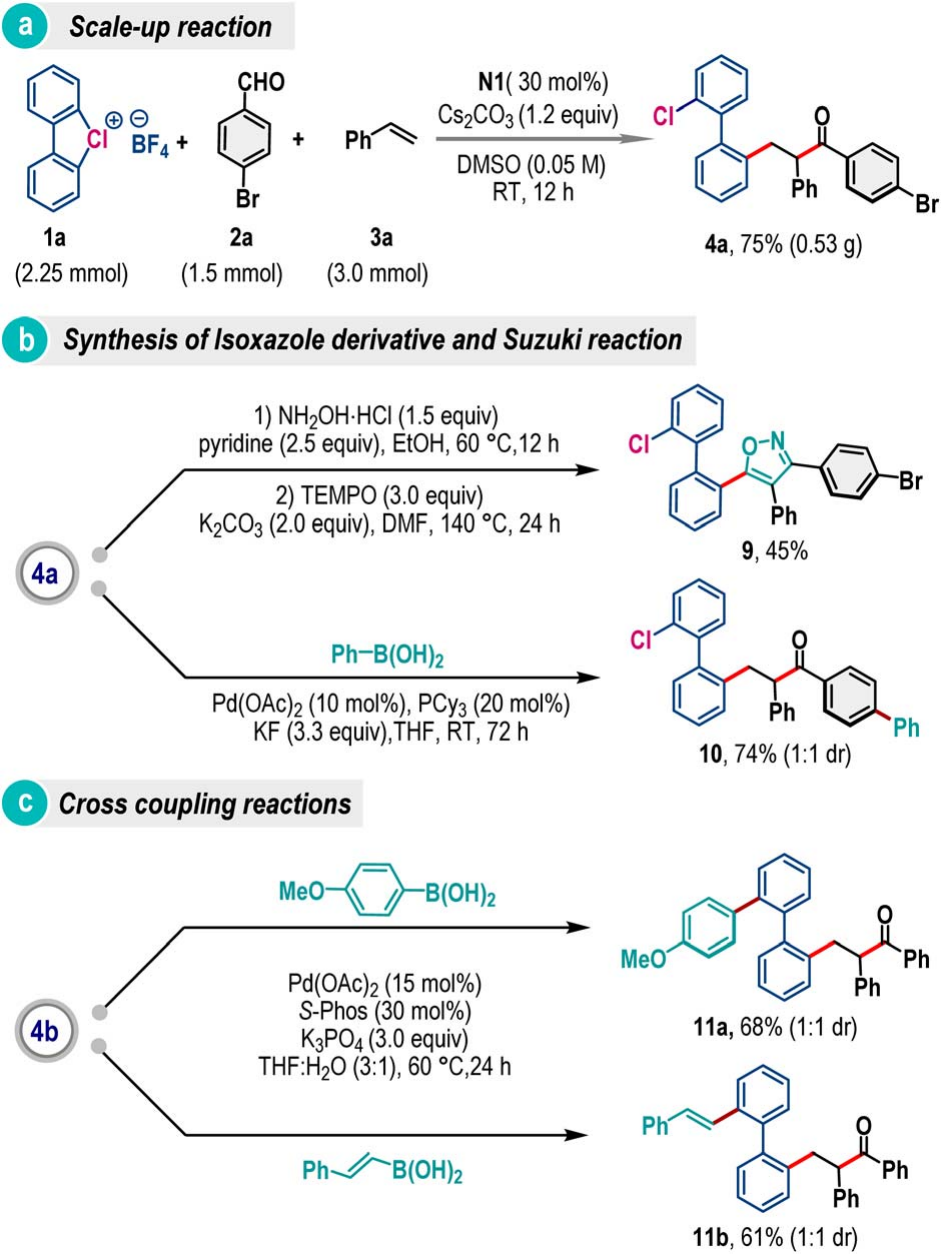
Scale-up reactions and post-synthetic applications.

To demonstrate the synthetic utility, we have carried out a scale–up reaction and product 4a was obtained in 75% yield from a 1.5 mmol scale reaction (Scheme 5a). The product 4a was also transformed into isoxazole embedded biaryl 9 by treating with hydroxylamine followed by the TEMPO mediated cyclization reaction (Scheme 5b).^13^ Further diversification has been accomplished through site-selective Suzuki coupling, offering biaryl 10 in 74% yield (Scheme 5b). Similarly, product 4b was exposed to palladium-catalyzed carbon–carbon coupling reaction conditions with 4-methoxyphenylboronic acid and *trans*-2-phenylvinylboronic acid, where products 11a and 11b were formed in 68% and 61% yields, respectively (Scheme 5c).

**Scheme 6:**
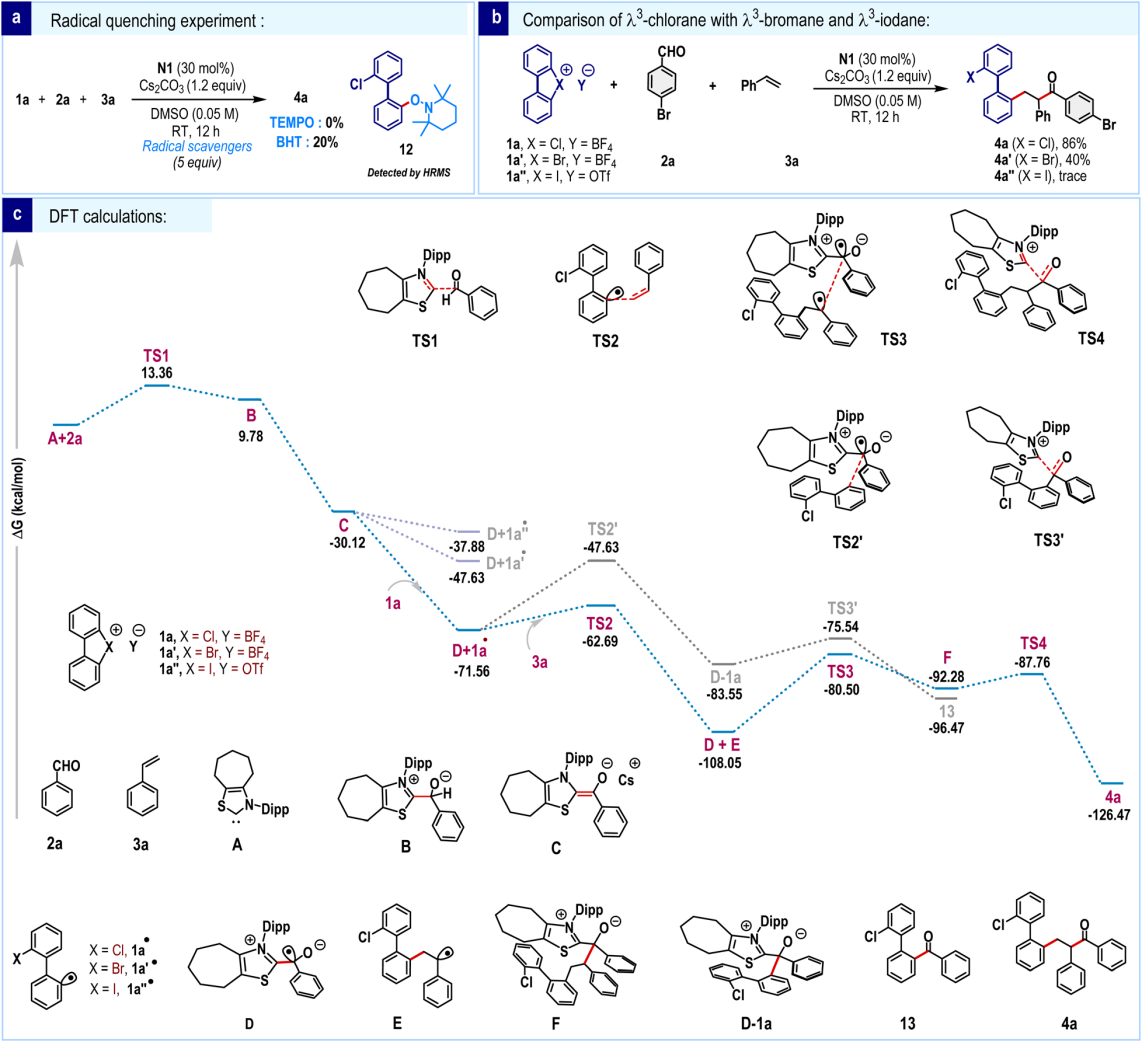
Mechanistic investigation.^*a* a^All DFT calculations were performed using Gaussian 16 (Rev. C.01) with the ωB97X-D functional and def2-TZVP basis set. Geometries were optimized, and vibrational frequency and IRC analyses were used to confirm minima and transition states. Solvent effects (DMSO) were included *via* the SMD model in single-point energy calculations to obtain thermodynamic parameters at the same level of theory. Relative free energies are given in kcal mol^−1^.

To provide insight into the mechanism, few control experiments were conducted. The reaction was significantly suppressed in the presence of radical scavengers such as TEMPO and BHT, suggesting the formation of a radical species (Scheme 6a). Additionally, we detected the TEMPO adduct 12 through HRMS, further supporting the formation of biaryl radicals. The reactivities of cyclic diaryl λ^3^-chlorane (1a), λ^3^-bromane (1a′) and λ^3^-iodane (1a″) were compared (Scheme 6b). Under the standard reaction conditions, 1a′ gave the three-component coupling product 4a′, albeit in significantly lower yield. In contrast, product formation was negligible for λ^3^-iodane (1a″). The superior reactivity of cyclic diaryl λ^3^-chlorane can be attributed to the higher electronegativity of chlorine compared to bromine and iodine, which leads to faster bond dissociation.

**Fig. 1:**
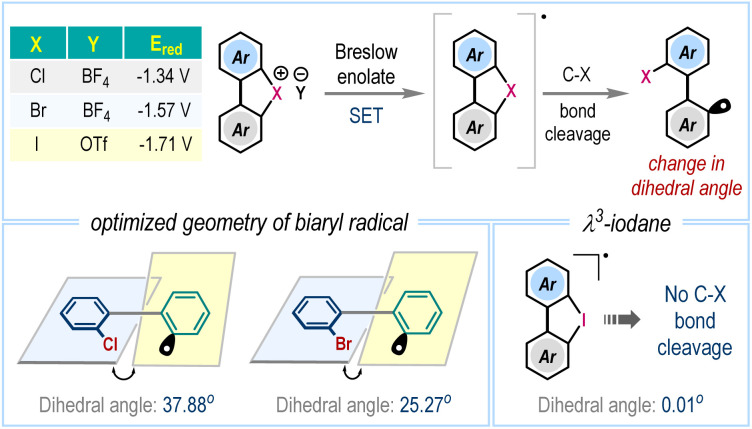
Optimized geometry of biaryl radicals.

To gain deeper insights into the reaction mechanism, density functional theory (DFT) calculations were performed (Scheme 6c). The process initiates with a nucleophilic attack by the carbene species A on aldehyde 2a, forming a tetrahedral intermediate B*via* the transition state TS1 (13.36 kcal mol^−1^). Subsequent base-mediated deprotonation leads to the formation of intermediate C (−30.12 kcal mol^−1^), commonly referred to as Breslow enolate, in a highly exergonic step. To examine the SET hypothesis, we constructed a combined system of C and 1a, mimicking the proposed experimental conditions. Upon geometry optimization, the system spontaneously evolved *via* C–Cl bond cleavage in the 1a unit, generating two neutral radicals D and 1a•. This transformation is exergonic by −41.44 kcal mol^−1^ (−71.56 kcal mol^−1^, Scheme 6c), reinforcing the feasibility of the SET-driven bond activation. Analogous calculations for the bromonium and iodonium congeners of 1a revealed similarly exothermic transformations, though to a lesser extent, with computed reaction energies of −17.51 kcal mol^−1^ and −7.76 kcal mol^−1^, respectively (Scheme 6c). A closer examination of the optimized geometries of the resulting radical species reveals notable differences in structural distortion at the biphenyl moiety. Specifically, the dihedral angle in the bromonium (1a′•) and chloronium radicals (1a•) is significantly more distorted, at 25.27° and 37.88°, respectively, whereas the radical species formed from λ^3^-iodane remains nearly planar at 0.01°(Fig. 1). The minimal structural perturbation and relatively modest reaction energy associated with the formation of the iodonium radical suggest that this species may be susceptible to a thermodynamically favorable back electron transfer reaction under realistic conditions.

In contrast, the more pronounced structural reorganization and highly exergonic nature of the 1a• formation underscore its greater thermodynamic stability and reinforce the viability of a SET-mediated C–Cl bond activation pathway. Furthermore, 1a• undergoes radical addition to styrene 3a to form intermediate E through a moderate energy barrier of 8.87 kcal mol^−1^ (TS2, −62.69 kcal mol^−1^). The consequent radical–radical coupling between D and E occurs *via*TS3 (−80.50 kcal mol^−1^), resulting in the formation of intermediate F. Finally, elimination of carbene species occurs through TS4 (−87.76 kcal mol^−1^), furnishing the desired three-component radical coupling product 4a, and regenerating the active carbene catalyst. Overall, the entire reaction pathway features favorable energetics with moderate activation barriers and highly exergonic steps, indicating a kinetically viable and thermodynamically favorable multistep transformation consistent with the proposed experimental observations. Additionally, we examined a potential radical-mediated two-component coupling involving direct interaction between radical species D and radical intermediate 1a•, occurring prior to the addition of olefin 3a (grey color). This competing pathway proceeds *via* transition states TS2′ (−47.63 kcal mol^−1^) and TS3′ (−75.54 kcal mol^−1^) to form the two-component product 13. However, the relatively high activation barrier associated with TS2′ (23.93 kcal mol^−1^) suggests that the formation of 13 is highly unfavourable under standard reaction conditions. In contrast, the radical addition of 1a• to styrene 3a proceeds with a significantly lower barrier of 8.87 kcal mol^−1^, rendering it as the kinetically preferred pathway.

## Conclusions

In summary, we have showcased for the first time the radical reaction modality of cyclic diaryl λ^3^-chloranes through the development of radical NHC catalysis. The protocol facilitates regioselective olefin difunctionalization in a three-component fashion involving cyclic diaryl λ^3^-chloranes, aromatic aldehydes, and olefins in the presence of an NHC-catalyst and offers a diverse range of unsymmetrical 2,2′-biaryls in high yields at room temperature. This aroylarylation protocol is operationally simple, scalable, features a wide substrate generality, and also remains effective in the presence of various medicinally relevant scaffolds. The biaryl products were further diversified *via* cross-coupling reactions and utilized in isoxazole synthesis, introducing additional molecular complexity. DFT studies reveal that the pivotal SET process from the Breslow enolate intermediate to the cyclic diaryl λ^3^-chlorane is a barrierless process, which is markedly distinct from the corresponding diaryl λ^3^-bromane and λ^3^-iodane congeners. Furthermore, the lower kinetic barriers associated with the radical relay process and the thermodynamic stability of the product jointly drive the reaction in its desired pathway, overcoming competitive two-component couplings. Notably, this work represents a pioneering advance in the use of λ^3^-chloranes in radical NHC catalysis and lays the groundwork for further exploration of their radical chemistry.

## Author contributions

The manuscript was written through contributions of all authors. All authors have given approval to the final version of the manuscript. M. B. and A. A. K conceptualized the idea. A. A. K., K. P. and M. G. carried out the experiments and mechanistic investigations, and analyzed the experimental data. K. L. S. and V. S. K. C. conducted the computational studies. All the authors discussed the results and co-wrote the manuscript.

## Conflicts of interest

There are no conflicts to declare.

## Supplementary Material

SC-OLF-D5SC09326K-s001

SC-OLF-D5SC09326K-s002

SC-OLF-D5SC09326K-s003

## Data Availability

General information, experimental procedures, characterization data for all new compounds, NMR spectra and details of DFT studies are available in the supplementary information (SI). Supplementary information is available. See DOI: https://doi.org/10.1039/d5sc09326k. CCDC 2441452 and 2444263 contain the supplementary crystallographic data for this paper.^14*a*,*b*^
