# Supplementary material for: Unlocking radical reactivity of cyclic diaryl λ3-chloranes through NHC-catalyzed three-component coupling
Source: Chem Sci. 2025 Dec 30;17(8):4277–84. doi: 10.1039/d5sc09326k (PMC12805865; doi:10.1039/d5sc09326k)

Supplementary Information (SI) for Chemical Science.
This journal is © The Royal Society of Chemistry 2025

-Electronic Supporting Information-

Part A

Unlocking Radical Reactivity of Cyclic Diaryl λ^3-Chloranes Through NHC-Catalyzed Three-Component Coupling

Anusree A. Kunhiraman[a], Koushik Patra[a], Venkata Surya Kumar Choutipalli[b], Manjeet Godara[a], Kevin L. Shuford[*b] and Mahiuddin Baidya[*a]

[a]Department of Chemistry, Indian Institute of Technology Madras,
Chennai, 600036, India.
Email: mbaidya@iitm.ac.in

[b]Department of Chemistry and Biochemistry, Baylor University, One Bear Place #97348,
Waco, Texas 76798-7348, United States.
Email: kevin_shuford@baylor.edu

–Table of contents –

1.	General information	S3
2.	General procedure for the synthesis of unsymmetrical 2,2'-disubstituted biaryls **4/5**	S4
3.	General procedure for the synthesis of difunctionalized 1,2-dihydroacenaphthylenes **7**	S5
4.	Gram scale synthesis of **4a**	S5
5.	Post-synthetic applications	S6
6.	Radical quenching experiment	S9
7.	Cyclic Voltammetry	S10
8.	Crystallographic experimental section	S11
9.	Spectroscopic data of isolated products	S16
10.	NMR spectra of synthesized compounds	S35

General Information:

All non-aqueous reactions were carried out under an atmosphere of nitrogen in flame-dried glassware and were stirred using a magnetic stir plate. All reactions were carried out using commercial-grade solvent unless otherwise noted. Dry DMF and DMSO were purchased from commercial sources and stored under nitrogen. CH_3CN, DCE, and CH_2Cl_2 were dried over calcium hydride. Dry THF was prepared by distilling over sodium ketyl.

All reactions were monitored by thin layer chromatography (TLC) on WhatmanPartisil® K6F TLC plates (silica gel 60 Å, 0.25 mm thickness) and visualized using a UV lamp (366 or 254 nm) or by use of one of the following visualization reagents: PMA: 10g phosphomolybdic acid/ 100 mL ethanol; $KMnO_4$: 0.75g potassium permanganate, 5g K_2CO_3, / 100 mL water. Products were isolated by column chromatography (Merck silica gel 100-200μm). Yields refer to chromatographically and spectroscopically homogenous materials unless noted otherwise. ^1H, ^{13}C, and ^{19}F NMR spectra were recorded on Bruker 400 or Bruker 500 MHz spectrometers. Chemical shift values (δ) are reported in ppm and calibrated to the residual solvent peak $CDCl_3$ δ = 7.2600 ppm for ^1H, δ = 77.16 for ^{13}C, DMSO-d_6 δ = 2.500 ppm for ^1H, δ = 39.500 ppm for ^{13}C; or calibrated to tetramethylsilane (δ = 0.00 ppm). All NMR spectra were recorded at ambient temperature (290 K) unless otherwise noted. ^1H NMR spectra are reported as follows: chemical shift (multiplicity, coupling constant, integration). The following abbreviations are used to indicate multiplicities: s, singlet; d, doublet; t, triplet; q, quartet; quint, quintet; sext, sextet; sept, septet; m, multiplet; dd, doublet of doublet; dt, doublet of triplet; dq, doublet of quartet; td, triplet of doublet; tt, triplet of triplet; dq, doublet of quartet; br, broad; app, apparent.

Mass spectra were recorded by electrospray ionization (ESI) method on a Q-TOF Micro with lock spray source. The crystal data were collected and integrated using a BrukerAxs kappa apex2 CCD diffractometer, with graphite monochromated Mo-Kα radiation.

Thiazolium salt **N1** was prepared by the reported procedure (*Eur. J. Org. Chem.* 2011, **2011**, 5475–5484*).* The λ^3-chloranes **1** were synthesized following literature procedures (*J. Am. Chem. Soc.* 2023, **145**, 345). All styrene derivatives were prepared by Wittig reaction from the corresponding aldehydes.

General procedure for the synthesis of unsymmetrical 2,2'- disubstituted biaryls (4,5):

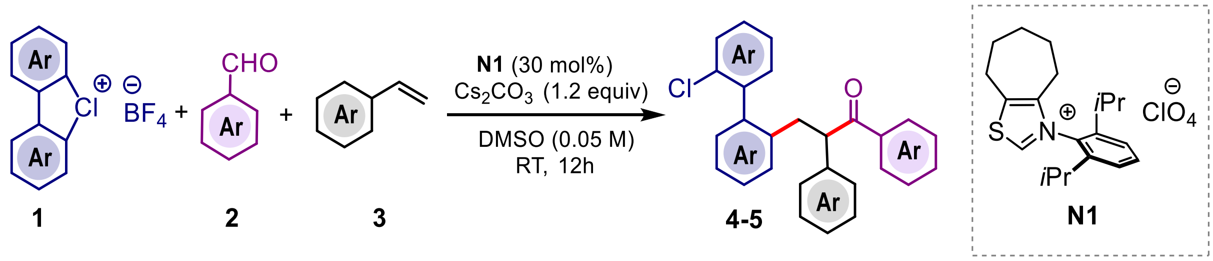

A 16×100 mm oven-dried reaction tube equipped with a magnetic stir bar was charged with corresponding λ^3-chlorane **1** (0.3 mmol, 1.5 equiv) and thiazolium salt **N1** (0.06 mmol, 0.3 equiv). The reaction tube was then sealed with a septum and purged with nitrogen. Degassed DMSO (4.0 mL), corresponding aldehyde **2** (0.2 mmol, 1 equiv), and styrene **3** (0.4 mmol, 2 equiv) were added. Next, Cs_2CO_3 (0.24 mmol, 1.2 equiv) was added under positive pressure of nitrogen, and the reaction mixture was stirred for 12 h at room temperature. After completion, the reaction was quenched with H_2O (5 mL) and then extracted with ethyl acetate (3x10 mL). The organic layer was washed with H_2O (3x10 mL), brine (1x10 mL), and then dried over sodium sulfate. The volatiles were removed under reduced pressure, and the crude product was purified by silica gel column chromatography to provide pure product **4/5**.

For solid aldehydes and styrenes, all components (**1**, **2**, **3**, and **N1)** were initially taken in the reaction tube prior to nitrogen purging.

General procedure for the synthesis of difunctionalized 1,2-dihydroacenaphthylenes (7):

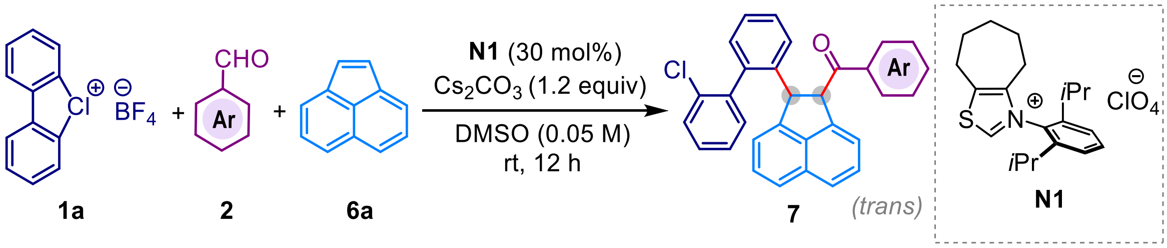

A 16×100 mm oven-dried reaction tube equipped with a magnetic stir bar was charged with corresponding λ^3-chlorane **1** (0.3 mmol, 1.5 equiv), thiazolium salt **N1** (0.06 mmol, 0.3 equiv), and acenaphthylene **6a** (0.4 mmol, 2 equiv). The reaction tube was then sealed with a septum and purged with nitrogen. Degassed DMSO (4.0 mL), and corresponding aldehyde **2** (0.2 mmol, 1 equiv) were added. Next, Cs_2CO_3 (0.24 mmol, 1.2 equiv) was added under positive pressure of nitrogen, and the reaction mixture was stirred for 12 h at room temperature. After completion, the reaction was quenched with H_2O (5 mL) and then extracted with ethyl acetate (3x10 mL). The organic layer was washed with H_2O (3x10 mL), brine (1x10 mL), and then dried over sodium sulfate. The volatiles were removed under reduced pressure, and the crude product was purified by silica gel column chromatography to provide pure product **7**.

For solid aldehydes all components (**1**, **2**, **6a**, and **N1)** were initially taken in the reaction tube prior to nitrogen purging.

Unsuccessful aliphatic aldehydes for this reaction:

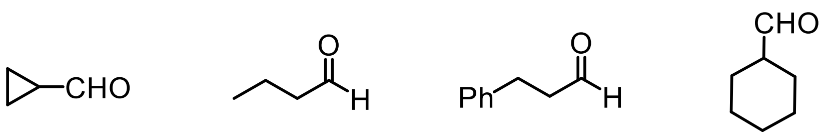

Gram scale synthesis of 4a:

A 100 mL oven dried round bottom flask equipped with a magnetic stir was charged with λ^3-chlorane **1a** (617.5 mg, 2.25 mmol, 1.5 equiv), 4-bromo benzaldehyde **2a** (277.5 mg, 1.5 mmol, 1 equiv) and thiazolium salt **N1** (0.45 mmol, 30 mol%)**.** The RB flask was then sealed with a septum and purged with nitrogen. Degassed DMSO (30.0 mL) were added to the tube. Next

styrene **3a** (344 µL, 3.0 mmol, 2 equiv) was added, followed by Cs_2CO_3 (587 mg, 1.8 mmol, 1.2 equiv) addition under positive pressure of nitrogen, and the reaction mixture was stirred for 12 h at room temperature. After completion, the reaction was quenched with H_2O (40 mL) and then extracted with ethyl acetate (3x 40mL). The organic layer was washed with H_2O (3x40 mL) and brine (1x40 mL), and then dried over sodium sulfate. The volatiles were removed under reduced pressure and the crude product was purified by silica gel column chromatography to provide pure product **4a**.

Post-synthetic applications:

a) General procedure for the synthesis of 9:

The compound was synthesized according to the previously reported procedure (*Org. Lett.* **2013**, *15*, 3214-3217). To an oven-dried screw-capped reaction tube equipped with a magnetic stir bar was added **4a** (95.1 mg, 0.2 mmol, 1 equiv), the screw-capped reaction tube was then evacuated and filled with nitrogen. Followed by the addition of EtOH (0.5 M), then hydroxylamine hydrochloride (20.8 mg, 0.3 mmol, 1.5 equiv) and pyridine (40 µL, 0.5 mmol, 2.5 equiv) was added to the reaction tube. The reaction mixture was then stirred at 60 °C for 12 h. The solvent was evaporated, and the residue was diluted with water and ethyl acetate. The aqueous layer was extracted with ethyl acetate. The combined organic layers were washed with 1N aqueous HCl and brine, dried over sodium sulfate, and concentrated. The crude reaction mixture was directly used for the next step.

To a solution of crude reaction mixture containing oxime in DMF (0.1 M) was added TEMPO (93.7 mg, 0.6 mmol, 3 equiv) and K_2CO_3 (55 mg, 0.4 mmol, 2 equiv). The reaction mixture was then stirred for 24 h at 140 °C under nitrogen atmosphere. The resulting mixture was cooled to room temperature and diluted with water and EtOAc. The aqueous layer was extracted with EtOAc for three times. The combined organic extracts were washed with brine, dried over sodium sulfate. After the volatiles were removed under reduced pressure and the crude product was purified by silica gel column chromatography to provide pure product **9** in 45% yield.

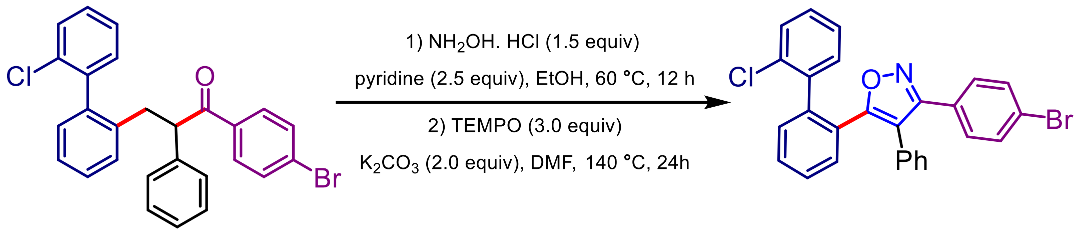

4a **9**, 45%

b) General procedure for the synthesis of 10:

The compound was synthesized according to the previously reported procedure (*J. Am. Chem. Soc.* **2023**, *145*, 345). A 16×100 mm oven-dried reaction tube equipped with a magnetic stir bar was charged with $Pd(OAC)_2$ (2.2 mg, 0.01 mmol, 10 mol%), phenylboronic acid (24.4 mg, 0.2 mmol, 2 equiv) and KF (19.2 mg, 0.33 mmol, 3.3 equiv). The reaction tube was sealed with a septum and then evacuated and filled with nitrogen. Then a solution of **4a** (47.6 mg, 0.1 mmol, 1 equiv.) in THF (0.2 mL, 0.5 M) was added *via* syringe. The resulting mixture was stirred at room temperature for 72 hours. The reaction was monitored with TLC. The crude was filtered through a pad of celite and washed with EtOAc (4 mL x 2 times). After the volatiles were removed under reduced pressure and the crude product was purified by silica gel column chromatography to provide pure product **10** in 74% yield.

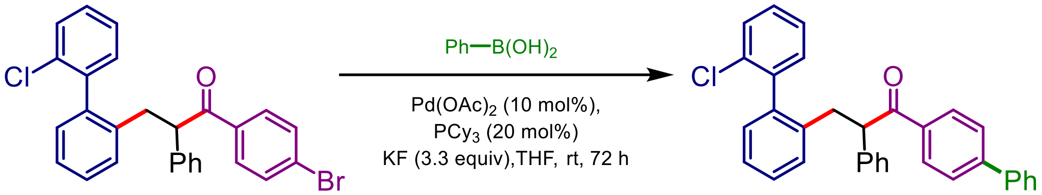

4a **10**, 74% (1:1 dr)

c) General procedure for the synthesis of 11a:

The compound was synthesized according to the previously reported procedure (*J. Am. Chem. Soc.* **2023**, 145, 345). A 16×100 mm oven-dried test tube equipped with a magnetic stir bar was charged with $Pd(OAC)_2$ (6.7 mg, 0.03 mmol, 15 mol%), and S-Phos (24.6 mg, 0.06 mmol, 30 mol%), the test tube was sealed with a septum and then evacuated and refilled with nitrogen. Then a mixture of THF-H_2O (3:1, 2 mL) was added. The resulting reaction mixture was stirred for 15 min to generate the active catalyst solution. Another 16×100 mm oven-dried reaction tube equipped with a magnetic stir bar was charged with **4b** (79.3, 0.2 mmol, 1 equiv), K_3PO_4

(127.4 mg, 0.6 mmol, 3 equiv), and 4-methoxyphenylboronic acid (36.5 mg, 0.24 mmol, 1.2 equiv). The reaction tube was sealed with a septum and then evacuated and refilled with nitrogen. Then the active catalyst solution was added to this reaction tube followed by the addition of a mixture of THF-H_2O (3:1, 1.8 mL). The resulting mixture was stirred at 60 °C for 24 hours. The crude reaction mixture was then cooled to room temperature, diluted with DCM, filtered through a pad of celite and washed with DCM (4x3 mL). After the volatiles were removed under reduced pressure and the crude product was purified by silica gel column chromatography to provide pure product **11a** in 68% yield.

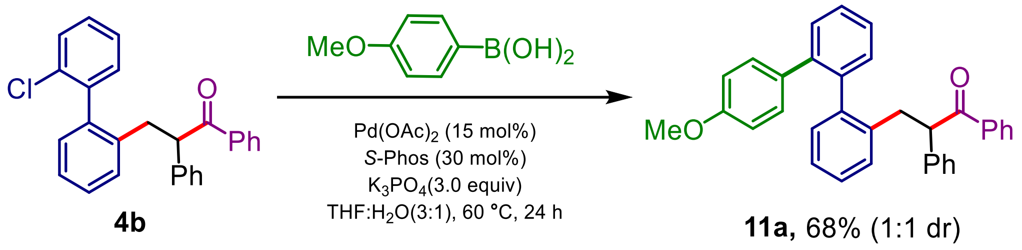

d) General procedure for the synthesis of 11b:

The compound was synthesized according to the previously reported procedure (*J. Am. Chem. Soc.* **2023**, 145, 345). A 16×100 mm oven-dried test tube equipped with a magnetic stir bar was charged with Pd(OAC)$_2$ (6.7 mg, 0.03 mmol, 15 mol%), and S-Phos (24.6 mg, 0.06 mmol, 30 mol%), the test tube was sealed with a septum and then evacuated and refilled with nitrogen. Then a mixture of THF-H_2O (3:1, 2 mL) was added. The resulting reaction mixture was stirred for 15 min to generate the active catalyst solution. Another 16×100 mm oven-dried reaction tube equipped with a magnetic stir bar was charged with **4b** (79.3, 0.2 mmol, 1 equiv), K_3PO_4 (127.4 mg, 0.6 mmol, 3 equiv), and *trans*-2-phenylvinylboronic acid (35.5 mg, 0.24 mmol, 1.2 equiv). The reaction tube was sealed with a septum and then evacuated and refilled with nitrogen. Then the active catalyst solution was added to this reaction tube followed by the addition of a mixture of THF-H_2O (3:1, 1.8 mL). The resulting mixture was stirred at 60 °C for 24 hours. The crude reaction mixture was then cooled to room temperature, diluted with DCM, filtered through a pad of celite and washed with DCM (4x3 mL). After the volatiles were removed under reduced pressure and the crude product was purified by silica gel column chromatography to provide pure product **11b** in 61% yield.

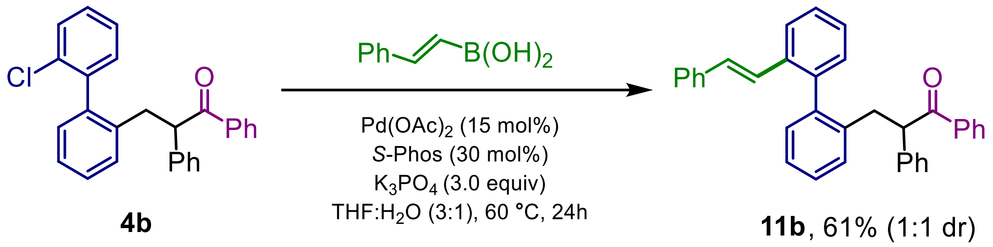

Radical quenching experiment:

A 16×100 mm oven-dried reaction tube equipped with a magnetic stir bar was charged with λ^3-chloranes **1a** (0.3 mmol, 1.5 equiv), thiazolium salt **N1** (0.06 mmol, 0.3 equiv), 4-bromo benzaldehyde **1a** (0.2 mmol, 1 equiv) and TEMPO or BHT (5.0 equiv, as a radical scavenger). The reaction tube was sealed with a septum and then evacuated and filled with nitrogen. Degassed DMSO (4.0 mL) was added to the tube. Next styrene **3** (0.4 mmol, 2 equiv) was added, followed by Cs_2CO_3 (0.24 mmol, 1.2 equiv). After 12 h stirring at room temperature, the reaction mixture was quenched with H_2O (5 mL) and then extracted with ethyl acetate (3x10 mL) and the organic layer was washed with H_2O (3x10 mL) and brine (1x10 mL) and dried over sodium sulfate. After the volatiles were removed under reduced pressure and the crude reaction mixture was submitted for HRMS analysis.

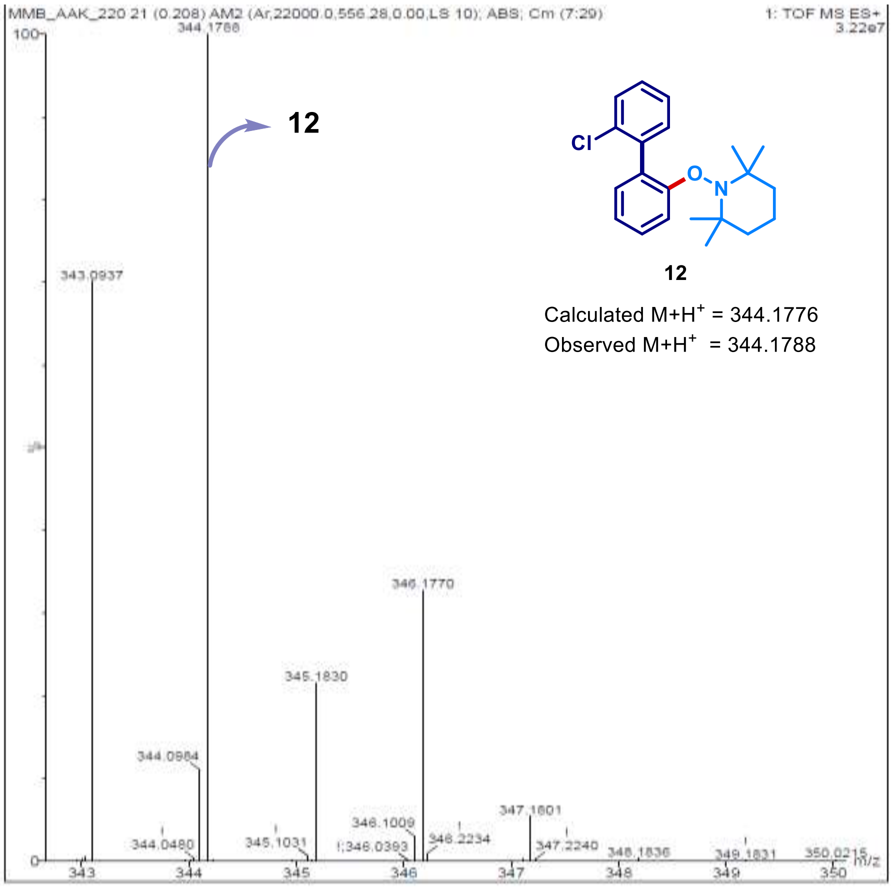

Cyclic Voltammetry:

CV measurements were carried out in DMSO solutions with 0.1 M of tetrabutylammonium tetrafluoroborate (Bu_4NBF_4) as a supporting electrolyte. Measurements were made with a glassy carbon electrode (area = 0.07 cm2), an Ag wire as reference electrode, and a Pt wire as counter electrode. All potentials are reported in V vs SCE. The $E_{1/2}$ of the Fc/Fc^+ redox couple used as a standard is 0.403 V vs SCE. The concentration of the sample solution was fixed at 10 mM and the sweep rates were set to 100 mV/s.

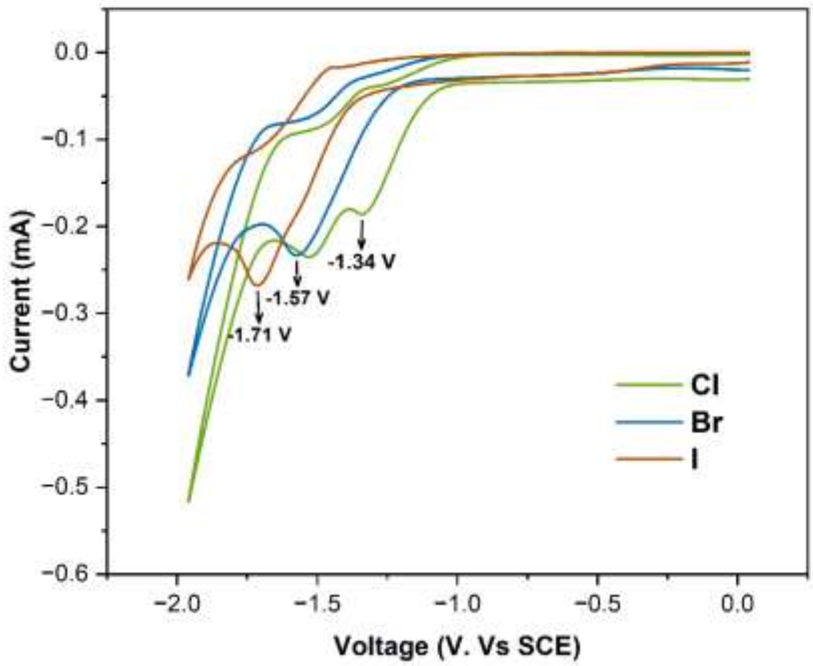

Crystallographic experimental section

a) X-ray crystal data of compound 7h:

Crystallization: Crystals of compound **7h** were obtained through a slow evaporation technique at room temperature from $CDCl_3$ solvent.

Crystal structure of compound **7h** (CCDC number: 2441452, Ellipsoid Probability 50%):

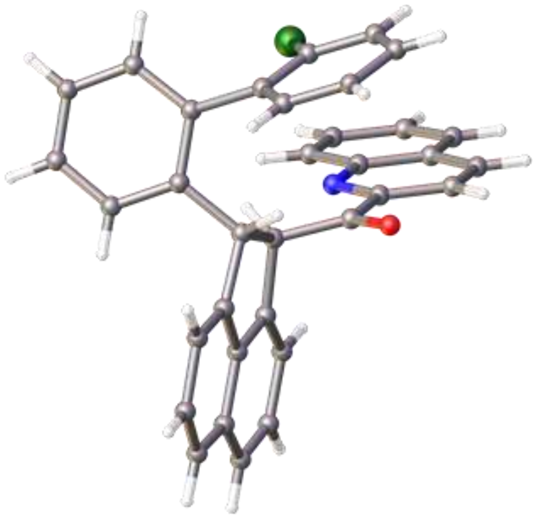

Identification code	**7h**
Empirical formula	C_{34} H_{22} Cl N O
Formula weight	495.97
Temperature	296(2) K
Wavelength	0.71073 A
Crystal system, space group	Monoclinic, P 21/n
Unit cell dimensions	a = 9.314(2) A alpha = 90 deg.
	b = 24.125(6) A beta = 92.692(6) deg.
	c = 11.578(3) A gamma = 90 deg.

Volume	2598.7(11) A^3
Z, Calculated density	4, 1.268 Mg/m^3
Absorption coefficient	0.175 mm^-1
F(000)	1032
Crystal size	0.596 x 0.520 x 0.406 mm
Theta range for data collection	2.346 to 25.264 deg.
Limiting indices	-11<=h<=11, -28<=k<=28, -13<=l<=13
Reflections collected / unique	37790 / 4593 [R(int) = 0.0955]
Completeness to theta = 25.242	97.7 %
Absorption correction	Semi-empirical from equivalents
Max. and min. transmission	1.0000 and 0.8641
Refinement method	Full-matrix least-squares on F^2
Data / restraints / parameters	4593 / 86 / 375
Goodness-of-fit on F^2	1.070
Final R indices [I>2sigma(I)]	R1 = 0.0756, wR2 = 0.1537
R indices (all data)	R1 = 0.1665, wR2 = 0.1824
Extinction coefficient	0.0034(5)

Largest diff. peak and hole	0.168 and -0.251 e.A^-3

b) X-ray crystal data of compound 7j:

Crystallization: Crystals of compound were obtained through a slow evaporation technique at room temperature from $CDCl_3$ solvent.

Crystal structure of compound **7j** (CCDC number: 2444263, Ellipsoid Probability 50%):

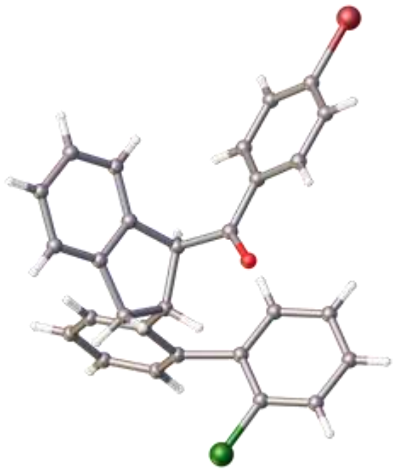

Identification code	**7j**
Empirical formula	C_{28} H_{20} Br Cl O
Formula weight	487.80
Temperature	298(2) K
Wavelength	0.71073 A
Crystal system, space group	Triclinic, P -1
Unit cell dimensions	a = 9.8017(10) A alpha = 81.365(3) deg.
	b = 10.1659(9) A beta = 84.820(4) deg.

	c = 11.4676(12) A gamma = 86.723(3) deg.
Volume	1124.00(19) A^3
Z, Calculated density	2, 1.441 Mg/m^3
Absorption coefficient	1.965 mm^-1
F(000)	496
Crystal size	0.176 x 0.162 x 0.130 mm
Theta range for data collection	2.510 to 25.038 deg.
Limiting indices	-11<=h<=11, -12<=k<=12, -13<=l<=13
Reflections collected / unique	50460 / 3966 [R(int) = 0.0814]
Completeness to theta = 25.038	99.6 %
Absorption correction	Semi-empirical from equivalents
Max. and min. transmission	0.7452 and 0.5656
Refinement method	Full-matrix least-squares on F^2
Data / restraints / parameters	3966 / 0 / 320
Goodness-of-fit on F^2	1.097
Final R indices [I>2sigma(I)]	R1 = 0.0430, wR2 = 0.1071
R indices (all data)	R1 = 0.0654, wR2 = 0.1198

Extinction coefficient	n/a
Largest diff. peak and hole	0.292 and -0.382 e.A^-3

Spectroscopic Data of Isolated Products

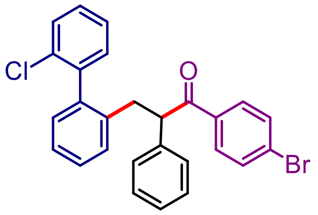

4a, 86%, dr = 1:1

Compound, 4a: pale yellow sticky liquid; eluent (1% ethyl acetate in hexane). Yield: 86 % (82 mg); **^{1}H NMR (500 MHz, $CDCl_3$) δ**: 7.53-7.49 (m, 4H), 7.37 (d, J = 7.8 Hz, 1H), 7.35 – 7.32 (m, 3H), 7.30-7.25 (m, 3H), 7.21-7.18 (m, 2H), 7.16-7.10 (m, 6H), 7.09-7.04 (m, 7H), 7.03 – 6.97 (m, 3H), 6.90-6.88 (m, 1H), 6.83-6.79 (m, 4H), 4.48 (dd, *J* = 9.1, 5.0 Hz,1H), 4.40 (dd, *J* = 8.2, 5.9 Hz, 1H), 3.41-3.33(m, 2H), 2.87-2.80(m, 2H). **^{13}C NMR (126 MHz, $CDCl_3$) δ**: 198.3, 198.0, 140.4, 140.2, 139.4, 139.19, 139.15, 138.5, 137.6, 137.5, 135.5, 135.3, 133.6, 133.4, 131.8, 131.7, 131.7, 131.5, 130.74, 130.68, 130.25, 130.22, 130.1, 130.0, 129.9, 129.7, 129.0, 128.93, 128.91, 128.3, 128.0, 127.9, 127.2, 127.0, 126.8, 126.3, 54.6, 53.9, 37.8, 37.6. HRMS (ESI/TOF-Q) m/z: [M+H]+ Calculated for $C_{27}H_{20}BrClOH^+$ 475.0459; Found 475.0457.

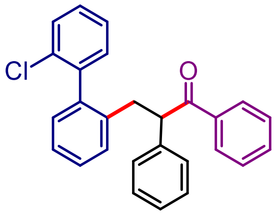

4b, 70%, dr = 1:1

Compound, 4b: colorless sticky liquid; eluent (1% ethyl acetate in hexane). Yield: 70% (55 mg); **^{1}H NMR (500 MHz, DMSO-d_6) δ** 7.74-7.71 (m, 4H), 7.56-7.39 (m, 7H), 7.36-7.32 (m, 5H), 7.29- 7.25 (m, 2H), 7.21-7.11(m, 7H),7.09-7.02 (m, 5H), 6.97-6.95 (m, 3H), 6.86-6.82 (m, 3H), 4.81-4.74 (m, 2H), 3.29 -3.24 (m, 1H), 3.18-3.14 (m, 1H), 2.83-2.78 (m, 1H), 2.69 -2.65 (m, 1H). **^{13}C NMR (126 MHz, DMSO-d_6) δ**: 198.7, 198.4, 139.6, 139.5, 139.0, 138.9, 138.8, 138.5, 137.2, 137.1, 135.79, 135.75, 133.3, 133.2, 132.4, 132.3, 131.7, 131.5, 129.8, 129.7, 129.6, 129.5, 129.3, 129.2, 128.8, 128.74, 128.73, 128.31, 128.29, 127.9, 127.84, 127.81, 127.7, 127.3, 127.13, 127.05, 126.9, 126.2, 53.1, 52.2, 36.8, 36.3. . HRMS (ESI/TOF-Q) m/z: [M+H]+ Calculated for $C_{27}H_{21}ClOH^+$ 397.1354; Found 397.1349.

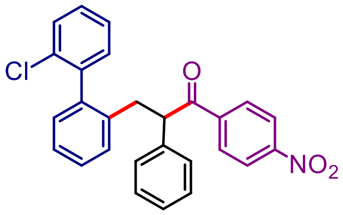

4c, 92%, dr = 1:1

Compound, 4c: yellow sticky liquid; eluent (3% ethyl acetate in hexane). Yield: 92% (81 mg); **^{1}H NMR (500 MHz, DMSO-d_6) δ** 8.20 (d, *J* = 8.4 Hz, 4H), 8.04-8.00(m, 4H), 7.59 (d, *J* = 7.4 Hz, 1H), 7.54 (d, *J* = 8.0 Hz, 1H), 7.51 – 7.37 (m, 5H), 7.31-7.28 (m, 2H), 7.24-7.19 (m, 6H), 7.15-7.13 (m, 4H), 7.10-7.09 (m, 1H), 7.04-7.00 (m, 3H), 6.87-6.85 (m, 2H), 6.75 (d, *J* = 7.5 Hz, 1H), 4.96-4.92 (m, 2H), 3.37 -3.33 (m, 1H), 3.24-3.20 (m, 1H), 2.93-2.89 (m, 1H), 2.77-2.72 (m, 1H). **^{13}C NMR (126 MHz, DMSO-d_6) δ**: 198.0, 197.7, 149.9, 149.8, 140.5, 140.3, 139.5, 139.4, 139.1, 138.9, 137.9, 137.6, 137.0, 136.8, 132.4, 132.3, 131.7, 131.5, 129.73, 129.65, 129.49, 129.46, 129.4, 129.3, 129.1, 129.0, 128.9, 128.1, 128.0, 127.9, 127.8, 127.4, 127.3, 127.2, 127.1, 126.31, 126.28, 123.91, 123.87, 54.1, 53.0, 36.5, 35.8. HRMS (ESI/TOF-Q) m/z: [M+H]+ Calculated for $C_{27}H_{20}ClNO_3H^+$ 442.1204; Found 442.1219.

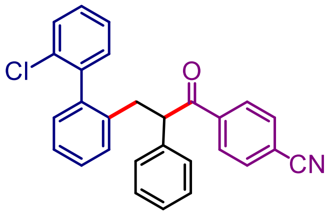

4d, 90%, dr = 1:1

Compound, 4d: pale yellow sticky liquid; eluent (3% ethyl acetate in hexane). Yield: 90% (76 mg); **^{1}H NMR (500 MHz, DMSO-d_6) δ** 7.95-7.93 (m, 3H), 7.91-7.87 (m, 5H), 7.60- 7.59 (m, 1H), 7.54 (d, *J* = 8.0 Hz, 1H), 7.51 – 7.45 (m, 3H), 7.42-7.37 (m, 2H), 7.29 (t, *J* = 7.4 Hz, 2H), 7.23-7.19 (m, 6H), 7.15-7.13 (m, 4H), 7.10 -7.08 (m, 1H), 7.04 -7.00 (m, 3H), 6.87-6.85 (m, 2H), 6.76 (d, *J* = 7.5 Hz, 1H), 4.94-4.90 (m, 2H), 3.36 -3.32 (m, 1H), 3.23-3.19 (m, 1H), 2.92-2.88 (m, 1H), 2.76 -2.71 (m, 1H). **^{13}C NMR (126 MHz, DMSO-d_6) δ**: 198.2, 197.9, 139.5, 139.4, 139.01, 138.99, 138.9, 138.0, 137.7, 136.9, 136.8, 132.82, 132.77, 132.4, 132.3, 131.7, 131.5, 129.7, 129.6, 129.5, 129.41, 129.40, 129.2, 129.1, 129.0, 128.9, 128.84, 128.80, 128.03, 127.95, 127.84, 127.76, 127.3, 127.1, 127.0, 126.3, 126.2, 118.0, 117.9, 115.3, 115.2, 53.7, 52.6, 36.5, 35.8. HRMS (ESI/TOF-Q) m/z: [M+H]+ Calculated for $C_{28}H_{20}ClNOH^+$ 422.1306; Found 422.1313.

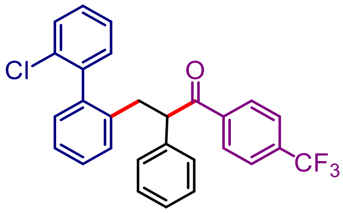

4e, 75%, dr = 1:1

Compound, 4e: yellow sticky liquid; eluent (2% ethyl acetate in hexane). Yield: 75% (70 mg); **^{1}H NMR (500 MHz, DMSO-d$_6$) δ**: 8.00-7.97 (m, 4H), 7.77 (d, *J* = 8.0 Hz, 4H), 7.61 – 7.59 (m, 1H), 7.55 (d, *J* = 8.0 Hz, 1H), 7.51-7.45 (m, 3H), 7.41-7.38 (m, 2H), 7.32-7.27 (m, 2H), 7.24-7.19 (m, 6H), 7.15 – 7.09 (m, 5H), 7.04-7.01(m, 3H), 6.88-6.86 (m, 2H), 6.81 (d, *J* = 7.3 Hz, 1H), 4.95-4.89 (m, 2H), 3.36 -3.33 (m, 1H), 3.26-3.21 (m, 1H), 2.93-2.89 (m, 1H), 2.77-2.73(m, 1H). **^{13}C NMR (126 MHz, DMSO-d$_6$) δ**: 198.2, 197.9, 139.5, 139.4, 139.02, 138.99, 138.9, 138.1, 137.9, 137.0, 136.9, 132.7, 132.6, 132.4, 132.34, 132.27, 131.7, 131.5, 129.68(*J* = 2.4 Hz), 129.43, 129.41, 129.2, 129.13, 129.10, 129.0, 128.9, 128.8, 128.0, 127.9, 127.83, 127.76, 127.3, 127.2, 127.1, 127.0, 126.2, 125.78, 125.75, 125.7, 123.56 (dd, *J* = 272.7, 3.1 Hz), 53.72, 52.67, 36.5, 35.9. **^{19}F NMR (471 MHz, DMSO-d$_6$) δ: -**61.75, -61.77. **HRMS (ESI/TOF-Q)** m/z: [M+H]+ Calculated for $C_{28}H_{20}ClF_3O$ H^+ 465.1228; Found 465.1239.

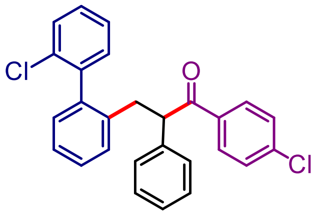

4f, 88%, dr = 1:1

Compound, 4f: colorless sticky liquid; eluent (1% ethyl acetate in hexane). Yield: 88% (76 mg); **^{1}H NMR (500 MHz, DMSO-d$_6$) δ**: 7.82-7.79 (m, 4H), 7.61-7.60 (m, 1H), 7.55 (d, *J* = 8.0 Hz, 1H), 7.50-7.46(m, 6H), 7.41-7.26 (m, 5H), 7.23-7.18 (m, 6H), 7.14-7.13 (m, 4H), 7.09-7.08 (m, 1H), 7.03-7.01 (m, 3H), 6.88-6.84 (m, 3H), 4.87-4.81(m, 2H), 3.34-3.30(m, 1H), 3.23-3.19 (m, 1H), 2.89-2.85 (m, 1H), 2.75-2.71 (m, 1H). **^{13}C NMR (126 MHz, DMSO-d$_6$) δ**: 197.7, 197.4, 139.5, 139.4, 139.0, 138.9, 138.5, 138.3, 138.24, 138.18, 137.1, 137.0, 134.43, 134.37, 132.4, 132.3, 131.7, 131.5, 130.21, 130.15, 129.70, 129.68, 129.5, 129.4, 129.23, 129.17, 128.9, 128.8, 127.9, 127.84, 127.81, 127.7, 127.3, 127.14, 127.07, 127.0, 126.2, 53.3, 52.2, 36.6, 36.1. **HRMS (ESI/TOF-Q) m/z**: [M+Na]+ Calculated for $C_{27}H_{20}Cl_2ONa^+$ 453.0783; Found 453.0786.

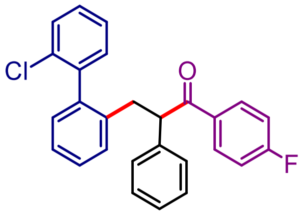

4g, 62%, dr = 1:1

Compound, 4g: brown sticky liquid; eluent (1% ethyl acetate in hexane). Yield: 62% (51 mg); **^{1}H NMR (500 MHz, DMSO-d$_6$) δ**: 7.91 – 7.86 (m, 3H), 7.62 – 7.60 (m, 1H), 7.55 (d, *J* = 8.0 Hz, 1H), 7.49 – 7.44 (m, 3H), 7.40 (t, *J* = 7.6 Hz, 1H), 7.36 – 7.31 (m, 3H), 7.29 – 7.19 (m, 10H), 7.15 – 7.08 (m, 6H), 7.04 – 7.02 (m, 3H), 6.89 – 6.86 (m, 3H), 4.87 – 4.81 (m, 2H), 3.34 – 3.30 (m, 1H), 3.21 (dd, *J* = 14.1, 8.2 Hz, 1H), 2.87 (dd, *J* = 14.2, 6.0 Hz, 1H), 2.73 (dd, *J* = 13.9, 8.0 Hz, 1H). **^{13}C NMR (126 MHz, DMSO-d$_6$) δ**: 197.3, 197.0, 164.9 (d, *J* = 252.4 Hz), 164.88 (d, *J* = 252.6 Hz 139.5, 139.4, 139.0, 138.9, 138.7, 138.4, 137.1, 137.0, 132.48, 132.46, 132.43, 132.35, 132.27, 131.7, 131.5, 131.4, 131.33, 131.30, 131.25, 129.71, 129.68, 129.5, 129.4, 129.3, 129.2, 128.9, 128.8, 127.9, 127.8, 127.7, 127.3, 127.10, 127.08, 127.0, 126.2, 115.9, 115.7, 53.1, 52.1, 36.7, 36.2. **^{19}F NMR (471 MHz, DMSO-d$_6$) δ: -**105.52, -105.61. **HRMS (ESI/TOF-Q) m/z**: [M+Na]+ Calculated for $C_{27}H_{20}ClFONa^+$ 437.1079; Found 437.1079.

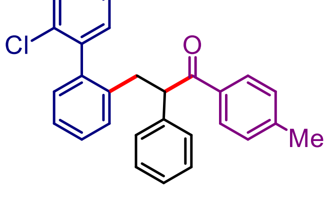

4h, 65%, dr = 1:1

Compound, 4h: colorless sticky liquid; eluent (1% ethyl acetate in hexane). Yield: 65% (53 mg); **^{1}H NMR (500 MHz, DMSO-d$_6$) δ**: 7.70-7.68 (m, 4H), 7.63-7.61(m, 1H), 7.57 (d, *J* = 7.9 Hz, 1H), 7.51 – 7.46 (m, 3H), 7.41 (t, *J* = 7.6 Hz, 1H), 7.35 (t, *J* = 7.4 Hz, 1H), 7.31-7.29 (m, 1H), 7.27-7.23 (m, 2H), 7.21-7.17 (m, 9H), 7.15 – 7.08 (m, 5H), 7.04 -7.00 (m, 3H), 6.93 (d, *J* = 8.4 Hz, 1H), 6.88 (d, *J* = 6.8 Hz, 2H), 4.83-4.76 (m, 2H), 3.31(dd, *J* = 14.0, 6.5 Hz,1H), 3.21 (dd, *J* = 14.2, 8.2 Hz,1H), 2.85 (dd, *J* = 14.2, 6.2 Hz, 1H), 2.72 (dd, *J* = 14.0, 7.8 Hz, 1H), 2.28 (s, 6H). **^{13}C NMR (126 MHz, DMSO-d$_6$) δ**: 198.2, 197.9, 143.8, 143.7, 139.6, 139.5, 139.02, 138.98, 138.9, 138.8, 137.3, 137.2, 133.31, 133.28, 132.4, 132.3, 131.7, 131.5, 129.8, 129.7, 129.6, 129.5, 129.30, 129.28, 129.25, 128.8, 128.7, 128.4, 127.9, 127.8, 127.7, 127.3, 127.1, 127.0, 126.9, 126.2, 52.9, 52.0, 36.8, 36.3, 21.1. **HRMS (ESI/TOF-Q) m/z**: [M+H]+ Calculated for $C_{28}H_{23}ClOH^+$ 411.1510; Found 411.1520.

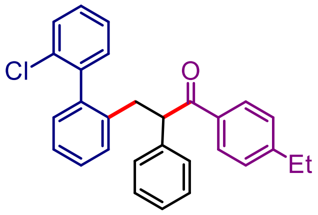

4i, 63%, dr = 1:1

Compound, 4i: yellow sticky liquid; eluent (1% ethyl acetate in hexane). Yield: 63% (54 mg); **^{1}H NMR (400 MHz, CDCl$_3$) δ**: 7.72-7.68 (m, 4H), 7.58-7.55(m, 1H), 7.48 – 7.46 (m, 1H), 7.39-7.34(m, 3H), 7.31-7.27 (m, 2H), 7.24-7.17 (m, 6H), 7.15-7.08 (m, 12H), 7.01-6.99 (m, 1H), 6.96-6.92 (m, 4H), 4.66 –4.63 (m, 1H), 4.57-4.54 (m, 1H), 3.51-3.42 (m, 2H), 2.96-2.88 (m, 2H), 2.64-2.58 (m, 4H), 1.21-1.17 (m, 6H). **^{13}C NMR (101 MHz, CDCl$_3$) δ**: 198.9, 198.6, 149.8, 140.5, 140.3, 139.8, 139.5, 139.3, 139.2, 138.0, 137.9, 134.5, 134.4, 133.7, 133.5, 131.7, 131.6, 130.8, 130.7, 130.2, 129.94, 129.88, 129.7, 129.03, 128.97, 128.92, 128.89, 128.86, 128.8, 128.4, 128.1, 128.0, 127.9, 127.0, 126.9, 126.8, 126.23, 126.19, 54.4, 53.8, 37.9, 37.7, 29.0, 15.2. **HRMS (ESI/TOF-Q) m/z**: [M+H]+ Calculated for $C_{29}H_{25}ClOH^+$ 425.1667; Found 425.1647.

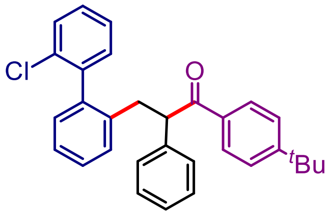

4j, 68%, dr = 1:1

Compound, 4j: yellow sticky liquid; eluent (1% ethyl acetate in hexane). Yield: 68% (62 mg); **^{1}H NMR (500 MHz, DMSO-d$_6$) δ**: 7.76-7.74 (m, 4H), 7.63-7.56 (m, 2H), 7.48-7.35 (m, 9H), 7.30 – 7.17 (m, 8H), 7.15-7.09 (m, 5H), 7.04-7.01 (m, 3H), 6.97 (d, J = 7.3 Hz, 1H), 6.89 (d, J = 7.2 Hz, 2H), 4.85 (t, J = 6.9 Hz, 1H), 4.78 (t, J = 6.9 Hz, 1H), 3.33-3.31(m, 1H), 3.25-3.21(m, 1H), 2.86-2.82 (m, 1H), 2.74-2.70 (m, 1H), 1.21 (bs, 18H). **^{13}C NMR (126 MHz, CDCl$_3$) δ**: 198.0, 197.7, 156.4, 156.3, 139.6, 139.5, 139.1, 139.0, 138.9, 138.8, 137.3, 137.2, 133.2, 132.34, 132.25, 131.7, 131.5, 129.8, 129.7, 129.4, 129.3, 129.2, 128.8, 128.7, 128.3, 127.81, 127.77, 127.75, 127.7, 127.3, 127.1, 127.0, 126.9, 126.2, 126.1, 125.6, 125.5, 53.0, 52.1, 36.7, 36.4, 34.7, 30.7. **HRMS (ESI/TOF-Q) m/z**: [M+H]+ Calculated for $C_{31}H_{29}ClOH^+$ 453.1980; Found 453.1986.

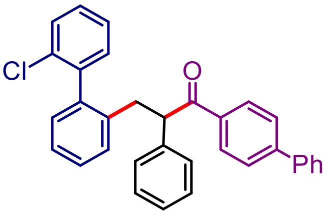

4k, 71%, dr = 1:1

Compound, 4k: yellow sticky liquid; eluent (1% ethyl acetate in hexane). Yield: 71% (67 mg); **^{1}H NMR (400 MHz, CDCl$_3$) δ:** 7.85 – 7.81 (m, 4H), 7.59 – 7.57 (m, 1H), 7.53 – 7.49 (m, 7H), 7.48 – 7.45 (m, 1H), 7.43 – 7.33 (m, 9H), 7.29 – 7.08 (m, 17H), 7.01 – 6.99 (m, 1H), 6.96 – 6.94 (m, 4H), 4.69 – 4.65 (m, 1H), 4.60 – 4.57 (m, 1H), 3.53 – 3.43 (m, 2H), 2.97 – 2.90 (m, 2H). **^{13}C NMR (101 MHz, CDCl$_3$) δ**: 198.9, 198.6, 145.5, 140.5, 140.3, 140.00, 139.96, 139.6, 139.5, 139.3, 139.0, 137.9, 137.8, 135.5, 135.3, 133.7, 133.5, 131.7, 131.6, 130.8, 130.7, 130.2, 130.0, 129.9, 129.7, 129.4, 129.3, 129.02, 128.99, 128.9, 128.4, 128.3, 128.2, 128.0, 127.9, 127.3, 127.2, 127.16, 127.08, 127.0, 126.8, 126.29, 126.27, 54.6, 54.0, 37.9, 37.7. **HRMS (ESI/TOF-Q) m/z**: [M+H]+ Calculated for $C_{33}H_{25}ClOH^+$ 473.1667; Found 473.1667.

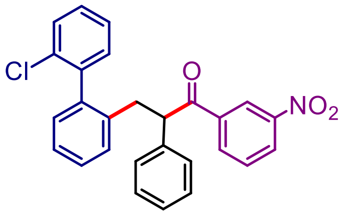

4l, 78%, dr = 1:1

Compound, 4l: yellow sticky liquid; eluent (1% ethyl acetate in hexane). Yield: 78% (69 mg); **^{1}H NMR (500 MHz, DMSO-d$_6$) δ**: 8.52 – 8.50 (m, 2H), 8.34 (d, J = 8.0 Hz, 2H), 8.22 (t, J = 7.9 Hz, 2H), 7.70 (t, J = 7.9 Hz, 1H), 7.61-7.59 (m, 1H), 7.54 – 7.53 (m, 1H), 7.49-7.42 (m, 4H), 7.38 (t, J = 7.8 Hz, 1H), 7.31-7.28 (m, 3H), 7.22-7.20 (m, 5H), 7.15-7.14 (m, 4H), 7.10-7.09 (m, 1H), 7.05-7.00 (m, 3H), 6.89 (d, J = 7.1 Hz, 2H), 6.79 (d, J = 7.5 Hz, 1H), 4.98-4.93 (m, 2H), 3.37-3.34(m, 1H), 3.28-3.23 (m, 1H), 2.94-2.90 (m, 1H), 2.80-2.75 (m, 1H). **^{13}C NMR (126 MHz, DMSO-d$_6$) δ**: 197.2, 196.9, 148.02, 147.99, 139.5, 139.4, 139.0, 138.9, 138.0, 137.7, 136.9, 136.82, 136.78, 134.4, 134.3, 132.4, 132.3, 131.7, 131.5, 130.7, 130.6, 129.8, 129.7, 129.51, 129.46, 129.4, 129.2, 129.13, 129.05, 128.9, 128.0, 127.9, 127.8, 127.6, 127.42, 127.36, 127.3, 127.2, 127.0, 126.3, 122.62, 122.56, 53.7, 52.6, 36.6, 35.9. **HRMS (ESI/TOF-Q) m/z**: [M+Na]+ Calculated for $C_{27}H_{20}ClNO_3Na^+$ 464.1024; Found 464.1027.

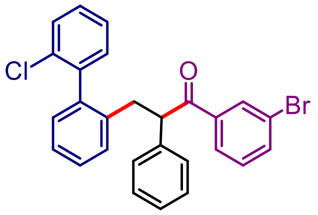

4m, 71%, dr = 1:1

Compound, 4m: brown sticky liquid; eluent (1% ethyl acetate in hexane). Yield: 71% (68 mg); **^{1}H NMR (500 MHz, DMSO-d$_6$) δ**: 7.93 (m, 2H), 7.79 (t, *J* = 7.4 Hz, 2H), 7.71 (d, *J* = 7.7 Hz, 2H), 7.61-7.60 (m, 1H), 7.55 (d, *J* = 7.9 Hz, 1H), 7.49 – 7.45 (m, 3H), 7.40-7.35 (m, 4H), 7.31-7.19 (m, 8H), 7.14-7.09 (m, 5H), 7.02-7.01 (m, 3H), 6.88 (d, *J* = 6.8 Hz, 2H), 6.81 (d, *J* = 7.6 Hz, 1H), 4.90-4.84 (m, 2H), 3.34-3.30 (m, 1H), 3.24-3.20 (m, 1H), 2.91-2.87 (m, 1H), 2.77-2.72 (m, 1H). **^{13}C NMR (126 MHz, DMSO-d$_6$) δ**: 197.6, 197.3, 139.5, 139.4, 139.0, 138.8, 138.3, 138.0, 137.8, 137.7, 137.0, 136.9, 135.9, 135.8, 132.34, 132.26, 131.7, 131.5, 131.0, 130.8, 130.7, 129.7, 129.44, 129.41, 129.2, 129.1, 128.9, 128.8, 127.9, 127.80, 127.76, 127.4, 127.29, 127.26, 127.2, 127.04, 127.00, 126.20, 126.18, 122.2, 122.1, 53.3, 52.3, 36.6, 36.0. **HRMS (ESI/TOF-Q) m/z**: [M+H]+ Calculated for $C_{27}H_{20}BrClOH^{+}$ 475.0459; Found 475.0439.

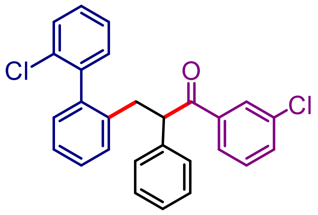

4n, 75%, dr = 1:1

Compound, 4n: yellow sticky liquid; eluent (1% ethyl acetate in hexane). Yield: 75% (65 mg); **^{1}H NMR (500 MHz, DMSO-d$_6$) δ**: 7.81-7.79 (m, 2H), 7.75 (t, *J* = 8.1 Hz, 2H), 7.61-7.54 (m, 4H), 7.51–7.49 (m, 1H), 7.47-7.37 (m, 8H), 7.31-7.19 (m, 7H), 7.14-7.09 (m, 4H), 7.04-7.01 (m, 3H), 6.89-6.88 (m, 2H), 6.81 (d, *J* = 7.5 Hz, 1H), 4.92-4.86 (m, 2H), 3.35-3.30 (m, 1H), 3.25-3.19 (m, 1H), 2.92-2.87 (m, 1H), 2.78-2.72 (m, 1H). **^{13}C NMR (126 MHz, DMSO-d$_6$) δ**: 197.7, 197.4, 139.5, 139.4, 139.0, 138.9, 138.4, 138.1, 137.62, 137.56, 137.0, 136.9, 133.8, 133.7, 133.0, 132.9, 132.4, 132.3, 131.7, 131.5, 130.7, 129.71, 129.69, 129.4, 129.21, 129.18, 129.1, 128.9, 128.8, 128.2, 128.0, 127.9, 127.83, 127.76, 127.3, 127.2, 127.05, 127.01, 126.9, 126.21, 126.19, 53.3, 52.3, 36.6, 36.0. **HRMS (ESI/TOF-Q) m/z**: [M+Na]+ Calculated for $C_{27}H_{20}Cl_2ONa^{+}$ 453.0783; Found 453.0784.

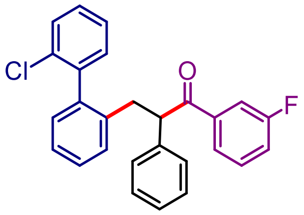

4o, 66%, dr = 1:1

Compound, 4o: yellow sticky liquid; eluent (1% ethyl acetate in hexane). Yield: 66% (55 mg); **^{1}H NMR (500 MHz, DMSO-d$_6$) δ**: 7.65 (t, *J* = 8.3 Hz, 2H), 7.61-7.54 (m, 4H), 7.51–7.45 (m, 5H), 7.39-7.37 (m, 4H), 7.32-7.26 (m, 2H), 7.22-7.19 (m, 6H), 7.14-7.08 (m, 5H), 7.05-7.01(m, 3H), 6.90 (d, *J* = 6.9 Hz, 2H), 6.84 (d, *J* = 7.4 Hz, 1H), 4.91-4.85 (m, 2H), 3.35-3.31 (m, 1H), 3.24-3.19 (m, 1H), 2.91-2.87 (m, 1H), 2.76-2.72 (m, 1H). **^{13}C NMR (126 MHz, DMSO-d$_6$) δ**: 197.7, 197.4, 162.1 (d, *J* = 245.6 Hz), 139.5, 139.4, 139.0, 138.9, 138.4, 138.2, 138.1, 138.01, 137.95, 137.04, 136.95, 132.4, 132.3, 131.7, 131.5, 131.0, 130.9, 129.69, 129.68, 129.44, 129.41, 129.23, 129.15, 128.9, 128.8, 128.0, 127.9, 127.83, 127.77, 127.3, 127.2, 127.1, 126.2(d, *J* = 2.4 Hz), 124.6 (d, *J* = 2.2 Hz), 124.5 (d, *J* = 2.6 Hz) , 120.3 (d, *J* = 13.8 Hz), 120.1(d, *J* = 13.9 Hz), 114.8 (d, *J* = 6.4 Hz), 114.6(d, *J* = 6.3 Hz) , 53.4, 52.4, 36.6, 36.0. **^{19}F NMR (471 MHz, DMSO-d$_6$) δ**: -111.84, -111.93. **HRMS (ESI/TOF-Q) m/z**: [M+Na]+ Calculated for $C_{27}H_{20}ClFONa^{+}$ 437.1079; Found 437.1084.

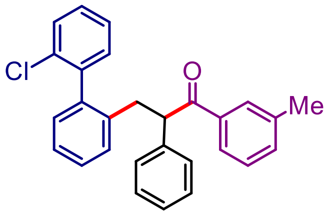

4p, 65%, dr = 1:1

Compound, 4p: yellow sticky liquid; eluent (1% ethyl acetate in hexane). Yield: 65% (53 mg); **^{1}H NMR (500 MHz, DMSO-d$_6$) δ**: 7.63-7.56 (m, 6H), 7.51 – 7.46 (m, 3H), 7.42-7.39 (m, 1H), 7.36-7.26 (m, 7H), 7.24-7.17 (m, 6H), 7.15-7.09 (m, 5H), 7.04-7.01 (m, 3H), 6.95-6.94 (m, 1H), 6.90 (d, *J* = 7.6 Hz, 2H), 4.86-4.78 (m, 2H), 3.36-3.30 (m, 1H), 3.26-3.21 (m, 1H), 2.90-2.86 (m, 1H), 2.77-2.73 (m, 1H), 2.28 (m, 6H). **^{13}C NMR (126 MHz, DMSO-d$_6$) δ**: 198.8, 198.5, 139.5, 139.4, 138.9, 138.8, 138.6, 138.1, 137.2, 137.1, 135.88, 135.86, 133.84, 133.78, 132.3, 132.2, 131.7, 131.5, 129.8, 129.7, 129.53, 129.45, 129.4, 129.24, 129.21, 128.8, 128.7, 128.58, 128.56, 128.5, 127.9, 127.8, 127.7, 127.2, 127.1, 127.0, 126.9, 126.1, 125.5, 53.0, 52.1, 36.8, 36.3, 20.8. **HRMS (ESI/TOF-Q) m/z**: [M+Na]+ Calculated for $C_{28}H_{23}ClONa^{+}$ 433.1330; Found 433.1322.

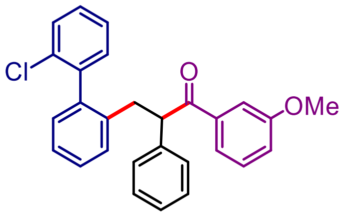

4q, 57%, dr = 1:1

Compound, 4q: yellow sticky liquid; eluent (1% ethyl acetate in hexane). Yield: 57% (49 mg); **^{1}H NMR (500 MHz, DMSO-d$_6$) δ**: 7.63-7.61 (m, 1H), 7.56 (d, *J* = 8.0 Hz, 1H), 7.51 – 7.45 (m, 3H), 7.42-7.38 (m, 3H), 7.36-7.30 (m, 6H), 7.27-7.09 (m, 14H), 7.04-7.00 (m, 3H), 6.93 (d, *J* = 7.4 Hz, 1H), 6.89 (d, *J* = 7.1 Hz, 2H), 4.87-4.78 (m, 2H), 3.73(s, 6H), 3.35-3.31 (m, 1H), 3.26-3.21 (m, 1H), 2.88-2.84 (m, 1H), 2.77-2.72 (m, 1H). **^{13}C NMR (126 MHz, DMSO-d$_6$) δ**: 198.4, 198.0, 159.3, 139.6, 139.5, 139.0, 138.93, 138.87, 138.6, 137.2, 137.1, 132.4, 132.3, 131.7, 131.5, 129.89, 129.86, 129.8, 129.7, 129.52, 129.46, 129.4, 129.3, 129.2, 128.8, 128.7, 127.9, 127.8, 127.7, 127.3, 127.1, 127.0, 126.9, 126.2, 120.79, 120.77, 119.2, 119.1, 112.92, 112.87, 55.2, 53.2, 52.3, 36.7, 36.3. **HRMS (ESI/TOF-Q) m/z**: [M+H]+ Calculated for $C_{28}H_{23}ClO_2H^+$ 427.1459; Found 427.1466.

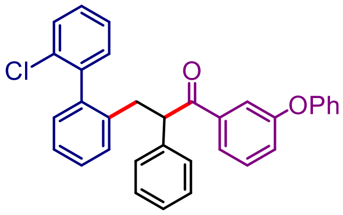

4r, 66%, dr = 1:1

Compound, 4r: orange sticky liquid; eluent (1% ethyl acetate in hexane). Yield: 66% (49 mg); **^{1}H NMR (500 MHz, DMSO-d$_6$) δ**: 7.57–7.50 (m, 4H), 7.44-7.36 (m,10H), 7.28 (d, *J* = 8.1 Hz, 4H), 7.24-7.16 (m, 11H), 7.13 – 7.12 (m, 4H), 7.07 (d, *J* = 6.8 Hz, 1H), 7.01 (d, *J* = 7.1 Hz, 1H), 6.97 (d, *J* = 7.8 Hz, 4H), 6.89-6.86 (m, 3H), 6.80 – 6.79 (m, 2H), 4.72-4.66 (m, 2H), 3.31 (dd, *J* = 13.9, 6.3 Hz, 1H), 3.22 (dd, *J* = 14.0, 8.6 Hz, 1H), 2.84 (dd, *J* = 14.1, 5.5 Hz, 1H), 2.72 (dd, *J* = 13.8, 7.8 Hz, 1H). **^{13}C NMR (126 MHz, DMSO-d$_6$) δ**: 198.0, 197.6, 157.4, 157.3, 155.6, 155.5, 139.5, 139.4, 139.0, 138.8, 138.7, 138.3, 137.5, 137.4, 137.1, 137.0, 132.4, 132.2, 131.6, 131.4, 130.6, 130.5, 130.3, 129.9, 129.8, 129.7, 129.5, 129.4, 129.3, 129.2, 128.9, 128.8, 127.84, 127.80, 127.75, 127.7, 127.3, 127.10, 127.07, 127.0, 126.2, 124.3, 124.2, 123.1, 123.02, 122.98, 119.5, 119.3, 116.9, 116.8, 53.4, 52.5, 36.7, 36.3. **HRMS (ESI/TOF-Q) m/z**: [M+Na]+ Calculated for $C_{33}H_{25}ClO_2Na^+$ 511.1435; Found 511.1441.

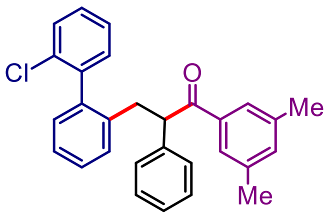

4s, 62%, dr = 1:1

Compound, 4s: pale yellow sticky liquid; eluent (1% ethyl acetate in hexane). Yield: 62% (53 mg); **^{1}H NMR (500 MHz, DMSO-d$_6$) δ**: 7.63-7.62 (m, 1H), 7.57 (d, *J* = 8.0 Hz, 1H), 7.49–7.46 (m, 3H), 7.42-7.40 (m, 5H), 7.35 (t, *J* = 7.5 Hz, 1H), 7.30-7.09 (m, 15H), 7.04 (d, *J* = 7.1 Hz, 1H), 7.00 (d, *J* = 7.7 Hz, 2H), 6.96 (d, *J* = 7.4 Hz, 1H), 6.91 (d, *J* = 7.5 Hz, 2H), 4.84-4.76 (m, 2H), 3.33-3.29 (m, 1H), 3.25-3.21 (m, 1H), 2.90-2.86 (m, 1H), 2.78-2.74 (m, 1H), 2.24 (s, 12H). **^{13}C NMR (126 MHz, DMSO-d$_6$) δ**: 198.9, 198.6, 139.6, 139.5, 138.9, 138.8, 138.6, 137.9, 137.2, 136.0, 134.6, 134.5, 132.32, 132.25, 131.7, 131.5, 129.8, 129.7, 129.6, 129.5, 129.4, 129.3, 129.2, 128.8, 128.7, 127.9, 127.8, 127.73, 127.71, 127.3, 127.1, 126.94, 126.86, 126.2, 126.00, 125.97, 52.8, 51.9, 36.8, 36.4, 20.7. **HRMS (ESI/TOF-Q) m/z**: [M+Na]+ Calculated for $C_{29}H_{25}ClONa^+$ 447.1486; Found 447.1486.

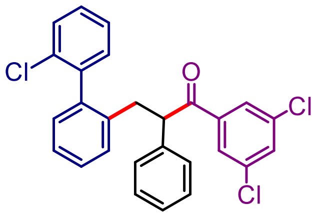

4t, 74%, dr = 1:1

Compound, 4t: yellow sticky liquid; eluent (1% ethyl acetate in hexane). Yield: 74% (69 mg); **^{1}H NMR (500 MHz, DMSO-d$_6$) δ**: 7.78-7.75 (m, 5H), 7.58-7.57 (m, 1H), 7.53–7.45 (m, 5H), 7.40-7.35 (m, 2H), 7.31-7.28 (m, 2H), 7.26-7.19 (m, 6H), 7.15-7.08 (m, 5H), 7.03 (d, *J* = 7.5 Hz, 2H), 6.99 (d, *J* = 7.5 Hz, 1H), 6.87-6.86 (m, 2H), 6.69 (d, *J* = 7.4 Hz, 1H), 4.97-4.93 (m, 2H), 3.31-3.28 (m, 1H), 3.21-3.16 (m, 1H), 2.91-2.87 (m, 1H), 2.75-2.70 (m, 1H). **^{13}C NMR (126 MHz, DMSO-d$_6$) δ**: 196.9, 196.5, 139.5, 139.4, 139.0, 138.9, 138.8, 138.7, 138.0, 137.7, 136.9, 136.8, 134.9, 134.8, 132.6, 132.4, 132.3, 131.7, 131.5, 129.8, 129.6, 129.5, 129.4, 129.3, 129.2, 129.1, 128.9, 128.1, 127.91, 127.88, 127.85, 127.5, 127.4, 127.3, 127.2, 126.97, 126.95, 126.9, 126.3, 126.2, 53.4, 52.3, 36.4, 35.6. **HRMS (ESI/TOF-Q) m/z**: [M+H]+ Calculated for $C_{27}H_{19}Cl_3OH^+$ 465.0574; Found 465.0564.

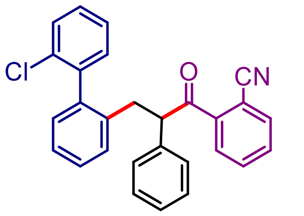

4u, 66%, dr = 1:1

Compound, 4u: yellow sticky liquid; eluent (2% ethyl acetate in hexane). Yield: 66% (56 mg); **^{1}H NMR (500 MHz, DMSO-d_6) δ**: 7.96 (d, *J* = 7.8 Hz, 1H),7.90-7.85 (m, 3H), 7.74–7.70 (m, 2H), 7.68 – 7.64 (m, 2H), 7.60-7.59 (m, 1H), 7.54 (d, *J* = 8.0 Hz, 1H), 7.49-7.45 (m, 4H), 7.39 (t, *J* = 7.7 Hz, 1H), 7.32-7.19 (m, 8H), 7.15-7.10 (m, 5H), 7.02-7.00 (m, 3H), 6.83-6.81(m, 2H), 6.73 (d, *J* = 7.4 Hz, 1H), 4.87-4.83 (m, 2H), 3.37-3.35 (m, 1H), 3.26-3.21 (m, 1H), 2.94-2.90 (m, 1H), 2.77-2.72 (m, 1H). **^{13}C NMR (126 MHz, DMSO-d_6) δ**: 198.3, 198.0, 139.4, 139.3, 139.1, 139.0, 138.91, 138.88, 137.5, 137.3, 136.8, 136.7, 135.5, 135.3, 133.07, 133.05, 132.9, 132.8, 132.33, 132.27, 131.7, 131.5, 129.7, 129.6, 129.5, 129.4, 129.3, 129.2, 129.1, 129.0, 128.8, 128.1, 128.0, 127.9, 127.8, 127.4, 127.3, 127.2, 127.1, 126.30, 126.27, 117.7, 117.6, 110.2, 54.6, 53.6, 36.1, 35.4. **HRMS (ESI/TOF-Q) m/z**: [M+Na]+ Calculated for $C_{28}H_{20}ClNONa^{+}$ 444.1126; Found 444.1136.

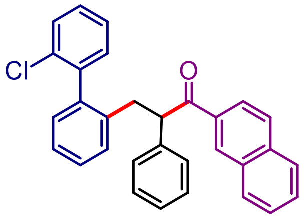

4v, 61%, dr = 1:1

Compound, 4v: colorless sticky liquid; eluent (2% ethyl acetate in hexane). Yield: 61% (54 mg); **^{1}H NMR (500 MHz, DMSO-d_6) δ**: 8.53 (s, 2H), 8.06 (d, *J* = 8.0 Hz, 2H), 7.92-7.91 (m, 4H), 7.86–7.82 (m, 2H), 7.64-7.55 (m, 7H), 7.51-7.44 (m, 2H), 7.42-7.39 (m, 2H), 7.36-7.33 (m, 1H), 7.31-7.19 (m, 7H), 7.17-7.09(m, 7H), 7.05 (d, *J* = 7.4 Hz, 1H), 7.00 (d, *J* = 7.5 Hz, 2H), 6.93 (d, *J* = 7.4 Hz, 1H), 5.10-5.04 (m, 2H), 3.43-3.40 (m, 1H), 3.32-3.28 (m, 1H), 2.99-2.95 (m, 1H), 2.85-2.81 (m, 1H). **^{13}C NMR (126 MHz, DMSO-d_6) δ**: 198.7, 198.4, 139.6, 139.5, 139.0, 138.9, 138.6, 137.3, 137.2, 134.89, 134.86, 133.13, 133.08, 132.4, 132.3, 131.9, 131.7, 131.5, 130.2, 130.1, 129.7, 129.6, 129.4, 129.23, 129.18, 128.79, 128.75, 128.7, 128.4, 128.0, 127.9, 127.8, 127.7, 127.5, 127.3, 127.1, 126.99, 126.97, 126.9, 126.2, 123.9, 123.8, 53.1, 52.0, 36.7, 36.2. **HRMS (ESI/TOF-Q) m/z**: [M+Na]+ Calculated for $C_{31}H_{23}ClONa^{+}$ 469.1330; Found 469.1332.

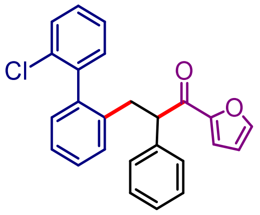

4w, 65%, dr = 1:1

Compound, 4w: pale yellow liquid; eluent (2% ethyl acetate in hexane). Yield: 65% (50 mg); **^{1}H NMR (500 MHz, DMSO-d_6) δ:** 7.89 (d, *J* = 6.2 Hz, 2H), 7.61-7.60 (m, 1H), 7.56 (d, *J* = 7.7 Hz, 1H), 7.45 – 7.34 (m, 7H), 7.30-7.26 (m, 2H), 7.24-7.20 (m, 6H), 7.17-7.14 (m, 4H), 7.09 (d, *J* = 7.1 Hz, 3H), 7.03 (t, *J* = 6.2 Hz, 2H), 6.97 (d, *J* = 7.1 Hz, 2H), 6.61 – 6.60 (m, 2H), 4.60 – 4.51 (m, 2H), 3.30 (dd, *J* = 13.8, 7.2 Hz, 1H), 3.19 (dd, *J* = 14.1, 8.4 Hz, 1H), 2.88 (dd, *J* = 14.2, 6.2 Hz, 1H), 2.74 (dd, *J* = 13.9, 7.5 Hz, 1H). **^{13}C NMR (126 MHz, DMSO-d_6) δ**: 187.1, 186.8, 151.1, 148.3, 148.2, 139.42, 139.37, 139.0, 138.9, 138.7, 138.4, 137.0, 136.9, 132.3, 132.2, 131.6, 131.5, 129.7, 129.6, 129.5, 129.42, 129.38, 129.3, 129.2, 128.7, 128.6, 127.82, 127.77, 127.75, 127.2, 127.1, 127.0, 126.3, 126.2, 119.5, 119.3, 112.6, 112.5, 53.4, 52.6, 35.8, 35.6. **HRMS (ESI/TOF-Q) m/z**: [M+H]+ Calculated for $C_{25}H_{19}ClO_2H^{+}$ 387.1146; Found 387.1149.

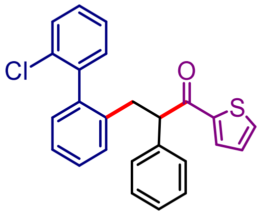

4x, 63%, dr = 1:1

Compound, 4x: yellow liquid; eluent (2% ethyl acetate in hexane). Yield: 63% (51 mg); **^{1}H NMR (500 MHz, DMSO-d_6) δ** 7.92 – 7.91 (m, 2H), 7.77-7.76 (m, 2H), 7.62 – 7.60 (m, 1H), 7.57 (d, *J* = 7.7 Hz, 1H), 7.46 – 7.37 (m, 5H), 7.30 (d, *J* = 7.3 Hz, 1H), 7.27 – 7.20 (m, 7H), 7.17 – 7.10 (m, 9H), 7.04 (t, *J* = 6.8 Hz, 2H), 6.98 (d, *J* = 7.1 Hz, 2H), 4.74 - 4.64 (m, 2H), 3.32 (dd, *J* = 13.8, 7.1 Hz, 1H), 3.20 (dd, *J* = 14.1, 8.3 Hz, 1H), 2.88 (dd, *J* = 14.2, 6.0 Hz, 1H), 2.75 (dd, *J* = 13.9, 7.5 Hz, 1H).**^{13}C NMR (126 MHz, DMSO-d_6) δ**: 191.8, 191.6, 143.04, 142.97, 139.5, 139.40, 138.98, 138.96, 138.9, 138.7, 137.0, 136.9, 135.6, 135.5, 133.5, 133.4, 132.33, 132.26, 131.7, 131.6, 129.7, 129.5, 129.43, 129.41, 129.33, 129.27, 128.8, 128.72, 128.67, 127.84, 127.76, 127.7, 127.3, 127.2, 127.1, 126.29, 126.25, 54.2, 53.4, 36.4, 36.1. **HRMS (ESI/TOF-Q) m/z**: [M+H]+ Calculated for $C_{25}H_{19}ClOSH^{+}$ 403.0918; Found 403.0937.

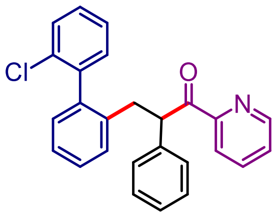

4y, 87%, dr = 1:1

Compound, 4y: yellow sticky liquid; eluent (5% ethyl acetate in hexane). Yield: 87% (69 mg); **^{1}H NMR (500 MHz, DMSO-d$_6$) δ** 8.64 – 8.62 (m, 2H), 7.88 – 7.85 (m, 4H), 7.59 – 7.57 (m, 1H), 7.56 – 7.52 (m, 3H), 7.42 – 7.37 (m, 5H), 7.31 (d, *J* = 7.3 Hz, 1H), 7.26 – 7.05 (m, 14H), 7.02 (d, *J* = 6.7 Hz, 2H), 6.93 (d, *J* = 7.2 Hz, 2H), 5.56 – 5.49 (m, 2H), 3.35 (m, 1H), 3.26 (dd, *J* = 14.1, 8.7 Hz, 1H), 2.94 (dd, *J* = 14.2, 6.1 Hz, 1H), 2.79 (dd, *J* = 13.8, 7.1 Hz, 1H). **^{13}C NMR (126 MHz, DMSO-d$_6$) δ**: 200.0, 199.8, 152.0, 151.9, 149.10, 149.05, 139.5, 139.4, 139.1, 138.9, 138.6, 138.4, 137.52, 137.47, 137.4, 137.3, 132.4, 132.3, 131.5 , 129.7, 129.53, 129.45, 129.32, 129.25, 129.18, 129.15, 128.5, 128.4, 128.21, 128.17, 127.8, 127.72, 127.65, 127.1, 127.0, 126.8, 126.7, 126.2, 126.1, 122.2, 50.9, 50.2, 35.93, 35.88. **HRMS (ESI/TOF-Q) m/z**: [M+H]+ Calculated for $C_{26}H_{20}ClNOH^{+}$ 398.1306; Found 398.1315.

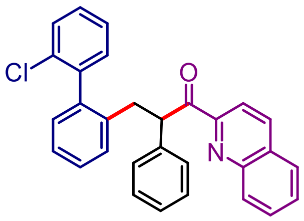

4z, 79%, dr = 1:1

Compound, 4z: white colored sticky liquid; eluent (5% ethyl acetate in hexane). Yield: 79% (71 mg); **^{1}H NMR (400 MHz, DMSO-d$_6$) δ** 8.42 – 8.38 (m, 2H), 8.19 - 8.14 (m, 2H), 7.98-7.96 (m, 2H), 7.94-7.91 (m, 2H), 7.89 – 7.84 (m, 2H), 7.72 – 7.67 (m, 2H), 7.64 – 7.60 (m, 1H), 7.56-7.54 (m, 1H), 7.50 – 7.38 (m, 5H), 7.32 – 7.30 (m, 1H), 7.29 – 7.27 (m, 1H), 7.25 – 7.23 (m, 1H), 7.21 – 7.16 (m, 4H), 7.15 – 7.03 (m, 12H), 5.79 – 5.73 (m, 2H), 3.43 (dd, *J* = 13.8, 7.9 Hz, 1H), 3.37 – 3.32 (m, 1H), 3.04 (dd, *J* = 14.2, 6.2 Hz, 1H), 2.89 (dd, *J* = 13.8, 7.1 Hz, 1H). **^{13}C NMR (101 MHz, DMSO-d$_6$) δ**: 200.0, 199.8, 151.5, 151.4, 146.2, 139.4, 139.1, 138.9, 138.7, 138.4, 137.64, 137.55, 137.3, 132.5, 132.3, 131.7, 131.5, 130.6, 130.5, 130.0, 129.9, 129.8, 129.6, 129.5, 129.4, 129.3, 129.2, 129.1, 128.98, 128.95, 128.5, 128.4, 128.2, 127.89, 127.85, 127.8, 127.7, 127.1, 127.0, 126.8, 126.7, 126.2, 126.1, 118.12, 118.10, 50.5, 50.0, 36.1, 35.8. **HRMS (ESI/TOF-Q) m/z**: [M+H]+ Calculated for $C_{30}H_{22}ClNOH^{+}$ 448.1463; Found 448.1469.

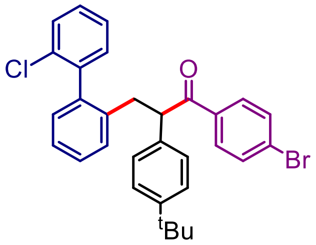

5a, 87%, dr = 1:1

Compound, 5a: brown sticky liquid; eluent (1% ethyl acetate in hexane). Yield: 87 % (92 mg); **^{1}H NMR (500 MHz, CDCl$_3$) δ**: 7.64 – 7.57 (m, 5H), 7.47-7.42 (m, 5H), 7.39 – 7.28 (m, 6H), 7.22 – 7.09 (m, 12H), 6.89 (d, *J* = 7.1 Hz, 1H), 6.79 (d, *J* = 7.4 Hz, 3H), 4.58 – 4.55 (m, 1H), 4.49 – 4.46 (m, 1H), 3.47 – 3.38 (m, 2H), 2.95 – 2.86 (m, 2H), 1.25 (s, 9H), 1.23 (s, 9H). **^{13}C NMR (126 MHz, CDCl$_3$) δ**: 198.4, 198.2, 150.1, 150.0, 140.4, 140.3, 139.5, 139.2, 137.82, 137.79, 136.0, 135.6, 135.5, 135.4, 133.7, 133.6, 131.84, 131.75, 131.5, 130.8, 130.7, 130.4, 130.3, 130.0, 129.9, 129.7, 129.0, 128.9, 128.03, 127.96, 127.93, 127.85, 127.5, 127.0, 126.7, 126.30, 126.27, 126.0, 125.9, 54.3, 53.5, 37.9, 37.6, 34.5, 31.42, 31.38. **HRMS (ESI/TOF-Q)** m/z: [M+H]+ Calculated for $C_{31}H_{28}BrClOH^{+}$ 531.1085; Found 531.1082.

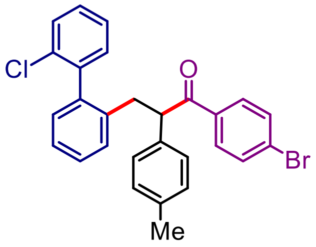

5b, 65%, dr = 1:1

Compound, 5b: yellow sticky liquid; eluent (1% ethyl acetate in hexane). Yield: 65 % (64 mg); **^{1}H NMR (500 MHz, DMSO-d$_6$) δ**: 7.72-7.69 (m, 4H), 7.61-7.60 (m, 5H), 7.55 (d, *J* = 8.1 Hz, 1H), 7.46 (d, *J* = 4.6 Hz, 3H), 7.40 (t, J = 7.7 Hz, 1H), 7.35-7.31 (m, 2H), 7.27 (t, *J* = 7.5 Hz, 1H), 7.23-7.21 (m, 4H), 7.09-7.08 (m, 1H), 7.03-6.98 (m, 3H), 6.94 (d, *J* = 7.7 Hz, 2H), 6.89 (t, *J* = 6.3 Hz, 3H), 6.75 (d, *J* = 7.6 Hz, 2H), 4.81-4.74 (m, 2H), 3.32-3.29 (m, 1H), 3.19 (dd, *J* = 14.1, 8.1 Hz, 1H), 2.84 (dd, *J* = 14.2, 6.0 Hz, 1H), 2.71 (dd, *J* = 14.0, 7.9 Hz, 1H), 2.16 (s, 6H). **^{13}C NMR (126 MHz, DMSO-d$_6$) δ**: 198.0, 197.7, 139.5, 139.4, 139.0, 138.8, 137.2, 137.1, 136.3, 136.2, 135.4, 135.2, 134.8, 134.7, 132.3, 132.2, 131.8, 131.6, 131.5, 130.22, 130.17, 129.7, 129.6, 129.42, 129.38, 129.3, 129.2, 129.1, 127.8, 127.7, 127.6, 127.4, 127.2, 127.0, 126.1, 52.8, 51.9, 36.5, 36.0, 20.4. **HRMS (ESI/TOF-Q) m/z**: [M+H]+ Calculated for $C_{28}H_{22}BrClOH^{+}$ 489.0615; Found 489.0611.

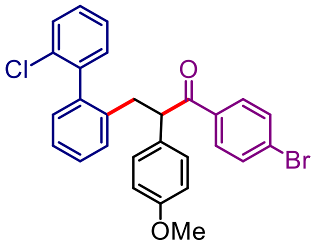

5c, 66%, dr = 1:1

Compound, 5c: yellow sticky liquid; eluent (1% ethyl acetate in hexane). Yield: 66 % (67 mg); **^{1}H NMR (500 MHz, DMSO-d$_6$) δ**: 7.73-7.70 (m, 4H), 7.62 – 7.59 (m, 5H), 7.56 (d, *J* = 8.0 Hz, 1H), 7.48 – 7.45 (m, 3H), 7.40 (t, *J* = 7.7 Hz, 1H), 7.35 – 7.33 (m, 2H), 7.27 (t, *J* = 7.4 Hz, 1H), 7.23 – 7.20 (m, 4H), 7.09 – 7.08 (m, 1H), 7.02 (d, *J* = 7.4 Hz, 1H), 6.92 (t, *J* = 8.1 Hz, 3H), 6.79-6.75 (m, 4H), 6.70 (d, *J* = 8.4 Hz, 2H), 4.80-4.73 (m, 2H), 3.64 (s, 6H), 3.28 (dd, *J* = 13.8, 6.0 Hz, 1H), 3.16 (dd, *J* = 14.3, 8.0 Hz, 1H), 2.84 (dd, *J* = 14.3, 6.4 Hz, 1H), 2.70 (dd, *J* = 14.0, 8.0 Hz, 1H). **^{13}C NMR (126 MHz, DMSO-d$_6$) δ**: 198.1, 197.8, 158.24, 158.20, 139.6, 139.5, 139.0, 138.9, 137.2, 137.1, 134.82, 134.76, 132.4, 132.3, 131.8, 131.7, 131.5, 130.3, 130.2, 130.0, 129.7, 129.6, 129.4, 129.24, 129.18, 129.0, 128.9, 127.8, 127.7, 127.4, 127.3, 127.1, 126.1, 114.3, 114.2, 54.9, 52.3, 51.3, 36.6, 36.0. **HRMS (ESI/TOF-Q) m/z**: [M+H]+ Calculated for $C_{28}H_{22}BrClO_2H^+$ 505.0564; Found 505.0566.

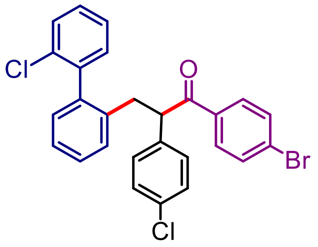

5d, 60%, dr = 1:1

Compound, 5d: brown sticky liquid; eluent (1% ethyl acetate in hexane). Yield: 60% (61 mg); **^{1}H NMR (500 MHz, CDCl$_3$) δ**: 7.60 – 7.55 (m, 4H), 7.49 – 7.43 (m, 5H), 7.41 – 7.36 (m, 2H), 7.35 – 7.34 (m, 1H), 7.33 – 7.32 (m, 1H), 7.31 – 7.27 (m, 3H), 7.25 – 7.20 (m, 5H), 7.16 – 7.12 (m, 5H), 7.04 (d, *J* = 7.6 Hz, 1H), 7.00 (dd, *J* = 7.3, 1.9 Hz, 1H), 6.85 – 6.81 (m, 4H), 4.53 (dd, *J* = 8.7, 5.3 Hz, 1H), 4.45 (dd, *J* = 8.6, 5.5 Hz, 1H), 3.47 – 3.39 (m, 2H), 2.93 – 2.86 (m, 2H). **^{13}C NMR (126 MHz, CDCl$_3$) δ**: 198.1, 197.8, 140.3, 140.1, 139.4, 139.2, 137.6, 137.2, 137.1, 137.0, 135.3, 135.1, 133.6, 133.4, 133.23, 133.20, 132.0, 131.9, 131.7, 131.5, 130.71, 130.67, 130.3, 130.2, 130.1, 130.0, 129.8, 129.7, 129.3, 129.2, 129.1, 129.0, 128.3, 128.1, 128.0, 127.0, 126.9, 126.6, 126.5, 53.8, 53.3, 37.8, 37.6. **HRMS (ESI/TOF-Q) m/z**: [M+H]+ Calculated for $C_{27}H_{19}BrCl_2OH^+$ 509.0069; Found 509.0060.

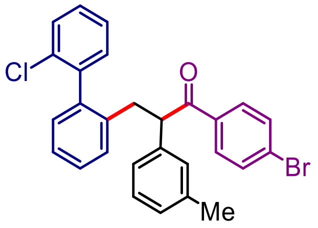

5e, 74%, dr = 1:1

Compound, 5e: yellow sticky liquid; eluent (1% ethyl acetate in hexane). Yield: 74% (72 mg); **^{1}H NMR (500 MHz, CDCl$_3$) δ**: 7.62 – 7.59 (m, 4H), 7.45 – 7.21 (m, 16H), 7.14 – 7.01 (m, 6H), 6.94 – 6.91 (m, 3H), 6.64 - 6.62 (m, 3H), 4.52 – 4.50 (m, 1H), 4.44 - 4.42 (m, 1H), 3.47 – 3.39 (m, 2H), 2.90 – 2.84 (m, 2H), 2.20 (s, 3H), 2.18 (s, 3H). **^{13}C NMR (126 MHz, CDCl$_3$) δ**: 198.3, 198.0, 140.4, 140.3, 139.5, 139.2, 138.8, 138.6, 138.5, 137.8, 137.7, 135.5, 135.3, 133.7, 133.5, 131.9, 131.8, 131.7, 131.5, 130.8, 130.6, 130.34, 130.29, 130.2, 130.0, 129.9, 129.7, 129.0, 128.89, 128.86, 128.8, 128.7, 128.4, 128.1, 128.0, 127.9, 127.0, 126.7, 126.4, 126.3, 125.5, 124.9, 54.7, 54.0, 37.8, 37.6, 21.5. **HRMS (ESI/TOF-Q) m/z**: [M+H]+ Calculated for $C_{28}H_{22}BrClOH^+$ 489.0615; Found 489.0615.

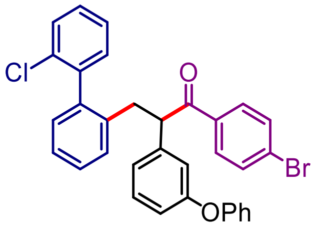

5f, 69%, dr = 1:1

Compound, 5f: brown sticky liquid; eluent (2% ethyl acetate in hexane). Yield: 69% (78 mg); **^{1}H NMR (500 MHz, DMSO-d$_6$) δ**: 7.72 – 7.69 (m, 4H), 7.63 – 7.61 (m, 4H), 7.55 – 7.51 (m, 2H), 7.46 – 7.44 (m, 1H), 7.42 – 7.39 (m, 2H), 7.37 – 7.32 (m, 6H), 7.29 - 7.25 (m, 3H), 7.24 -7.22 (m, 2H), 7.21 – 7.18 (m, 2H), 7.16 – 7.08 (m, 4H), 7.03 (dd, *J* = 7.2, 1.6 Hz, 1H), 6.86 – 6.82 (m, 3H), 6.80 – 6.75 (m, 5H), 6.72 (s, 1H), 6.62 (d, *J* = 7.7 Hz, 1H), 6.55 (s, 1H), 4.82 – 4.79 (m, 2H), 3.29 (dd, *J* = 14.1, 5.6 Hz, 1H), 3.17 (dd, *J* = 14.2, 7.3 Hz, 1H), 2.89 (dd, *J* = 14.2, 7.0 Hz, 1H), 2.74 (dd, *J* = 13.9, 8.8 Hz, 1H). **^{13}C NMR (126 MHz, DMSO-d$_6$) δ**: 197.8, 197.5, 156.6, 156.5, 156.4, 156.2, 140.4, 140.1, 139.4, 139.3, 138.9, 138.8, 136.8, 136.7, 134.7, 134.6, 132.3, 131.8, 131.6, 131.4, 130.4, 130.3, 130.24, 130.21, 129.9, 129.71, 129.66, 129.6, 129.4, 129.3, 129.2, 129.1, 127.8, 127.7, 127.5, 127.4, 127.2, 127.0, 126.2, 123.4, 122.7, 118.6, 118.5, 118.3, 118.2, 117.4, 117.3, 53.0, 52.1, 36.4, 35.8. **HRMS (ESI/TOF-Q) m/z**: [M+NH$_4$]+ Calculated for $C_{33}H_{24}BrClO_2NH_4^+$ 584.0986; Found 584.0964.

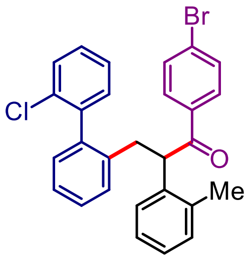

5g, 73%, dr = 1:1

Compound, 5g: brown sticky liquid; eluent (1% ethyl acetate in hexane). Yield: 73% (71 mg); **^{1}H NMR (500 MHz, DMSO-d$_6$) δ**: 7.59 – 7.54 (m, 8H), 7.49 (d, *J* = 8.0 Hz, 1H), 7.46 – 7.41 (m, 4H), 7.37 - 7.28 (m, 3H), 7.25 – 7.17 (m, 5H), 7.07 – 7.02 (m, 6H), 6.98 (d, *J* = 7.5 Hz, 1H), 6.94 – 6.91 (m, 2H), 6.74 (d, *J* = 7.7 Hz, 1H), 6.69 (dd, *J* = 7.6, 1.7 Hz, 1H), 4.88 (t, *J* = 7.0 Hz, 1H), 4.80 (dd, *J* = 8.3, 6.0 Hz, 1H), 3.33 (dd, *J* = 14.0, 6.4 Hz, 1H), 3.16 (dd, *J* = 14.2, 8.4 Hz, 1H), 2.83 (dd, *J* = 14.2, 5.9 Hz, 1H), 2.70 (dd, *J* = 14.0, 7.6 Hz, 1H), 1.94 (s, 3H), 1.83 (s, 3H). **^{13}C NMR (126 MHz, DMSO-d$_6$) δ**: 198.7, 198.2, 139.54, 139.46, 139.0, 138.8, 137.2, 136.8, 136.7, 136.6, 135.6, 135.41, 135.35, 135.2, 132.5, 132.2, 131.90, 131.86, 131.6, 131.5, 131.0, 130.9, 130.3, 130.2, 129.9, 129.8, 129.6, 129.5, 129.14, 129.05, 127.8, 127.7, 127.4, 127.3, 127.2, 127.1, 127.0, 126.9, 126.6, 126.3, 50.5, 49.1, 36.3, 36.0, 18.8, 18.6. **HRMS (ESI/TOF-Q) m/z**: [M+H]+ Calculated for $C_{28}H_{22}BrClOH^+$ 489.0615; Found 489.0590.

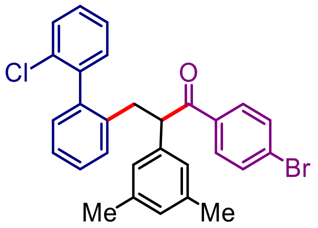

5h, 78%, dr = 1:1

Compound, 5h: yellow sticky liquid; eluent (1% ethyl acetate in hexane). Yield: 78% (79 mg); **^{1}H NMR (500 MHz, DMSO-d$_6$) δ**: 7.74 – 7.70 (m, 4H), 7.65 – 7.61 (m, 5H), 7.55 (dd, *J* = 8.0, 1.1 Hz, 1H), 7.50 – 7.41 (m, 4H), 7.38 (d, *J* = 7.0 Hz, 1H), 7.37 – 7.34 (m, 1H), 7.31 -7.27 (m, 1H), 7.26 – 7.21 (m, 4H), 7.10 – 7.08 (m, 1H), 7.01 (dd, *J* = 7.5, 1.2 Hz, 1H), 6.87 (dd, *J* = 7.5, 1.7 Hz, 1H), 6.75 (d, *J* = 5.2 Hz, 2H), 6.50 (s, 2H), 6.38 (s, 2H), 4.72 – 4.68 (m, 2H), 3.29 (dd, *J* = 14.0, 6.7 Hz, 1H), 3.20 (dd, *J* = 14.2, 8.7 Hz, 1H), 2.81 (dd, *J* = 14.2, 5.4 Hz, 1H), 2.67 (dd, *J* = 14.0, 7.4 Hz, 1H), 2.11 (s, 6H), 2.07 (s, 6H). **^{13}C NMR (126 MHz, DMSO-d$_6$) δ**: 197.9, 197.5, 139.5, 139.0, 138.8, 138.6, 138.2, 137.9, 137.7, 137.4, 137.2, 134.9, 134.8, 132.4, 132.3, 131.9, 131.8, 131.7, 131.5, 130.3, 130.2, 129.8, 129.7, 129.6, 129.5, 129.4, 129.3, 129.2, 128.6, 128.5, 127.83, 127.78, 127.5, 127.3, 127.0, 126.18, 126.16, 125.4, 125.3, 53.4, 52.4, 36.7, 36.1, 20.82, 20.79. **HRMS (ESI/TOF-Q) m/z**: [M+Na]+ Calculated for $C_{29}H_{24}BrClONa^+$ 525.0591; Found 525.0562.

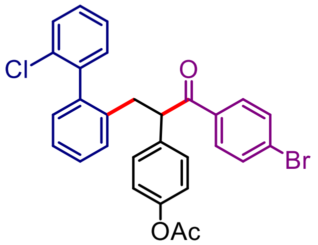

5i, 72%, dr = 1:1

Compound, 5i: brown sticky liquid; eluent (5% ethyl acetate in hexane). Yield: 72% (77 mg); **^{1}H NMR (500 MHz, CDCl$_3$) δ**: 7.77 -7.72 (m, 4H), 7.63 – 7.61 (m, 3H), 7.60 – 7.59 (m, 1H), 7.54 – 7.53 (m, 1H), 7.50 – 7.44 (m, 3H), 7.42 (d, *J* = 7.5 Hz, 1H), 7.39 -7.36 (m, 1H), 7.32 – 7.27 (m, 3H), 7.23 – 7.20 (m, 4H), 7.10 – 7.07 (m, 3H), 7.01 – 6.97 (m, 3H), 6.91 – 6.86 (m, 4H), 6.76 (dd, *J* = 7.5, 1.7 Hz, 1H), 4.92 – 4.86 (m, 2H), 3.30 (dd, *J* = 14.0, 5.7 Hz, 1H), 3.20 (dd, *J* = 14.3, 8.0 Hz, 1H), 2.89 (dd, *J* = 14.3, 6.5 Hz, 1H), 2.72 (dd, *J* = 14.0, 8.6 Hz, 1H), 2.20 (s, 3H), 2.19 (s, 3H). **^{13}C NMR (126 MHz, CDCl$_3$) δ**: 198.0, 197.7, 169.1, 169.0, 149.5, 149.4, 139.5, 139.3, 139.1, 138.9, 136.9, 136.8, 135.7, 135.5, 134.7, 134.6, 132.4, 132.2, 131.9, 131.8, 131.7, 131.6, 130.4, 130.3, 129.68, 129.66, 129.42, 129.36, 129.2, 129.0, 128.94, 128.92, 127.84, 127.76, 127.7, 127.5, 127.3, 127.1, 126.3, 126.2, 122.2, 122.1, 52.8, 51.4, 36.6, 36.0, 20.7. **HRMS (ESI/TOF-Q) m/z:** [M+H]+ Calculated for $C_{29}H_{22}BrClO_3H^+$ 533.0514; Found 533.0513.

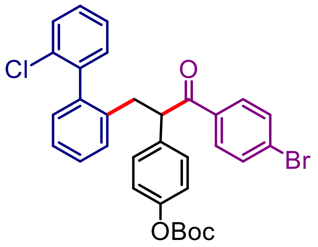

5j, 75%, dr = 1:1

Compound, 5j: brown sticky liquid; eluent (5% ethyl acetate in hexane). Yield: 75% (89 mg); **^{1}H NMR (400 MHz, CDCl$_3$) δ**: 7.61 – 7.56 (m, 5H), 7.47 – 7.42 (m, 5H), 7.41 – 7.31 (m, 3H), 7.29 – 7.27 (m, 1H), 7.26 – 7.19 (m, 6H), 7.15 – 7.13 (m, 1H), 7.11 – 7.08 (m, 2H), 6.99 – 6.95 (m, 5H), 6.89 – 6.86 (m, 4H), 4.57 (dd, *J* = 9.3, 4.8 Hz, 1H), 4.49 (dd, *J* = 8.3, 5.9 Hz, 1H), 3.48 – 3.38 (m, 2H), 2.93 – 2.86 (m, 2H), 1.53 (s, 9H), 1.52 (s, 9H). **^{13}C NMR (101 MHz, CDCl$_3$) δ**: 198.2, 197.9, 151.9, 151.8, 150.3, 150.2, 140.3, 140.1, 139.5, 139.2, 137.4, 137.3, 136.5, 135.9, 135.4, 135.2, 133.6, 133.4, 131.9, 131.8, 131.7, 131.6, 130.7, 130.6, 130.30, 130.26, 130.14, 130.06, 129.9, 129.7, 129.2, 129.1, 128.9, 128.8, 128.2, 128.1, 128.0, 127.1, 126.9, 126.5, 126.4, 121.8, 121.7, 83.74, 83.70, 53.9, 53.2, 37.9, 37.7, 27.8. **HRMS (ESI/TOF-Q) m/z:** [M+Na]+ Calculated for $C_{32}H_{28}BrClO_4Na^+$ 613.0752; Found 613.0748.

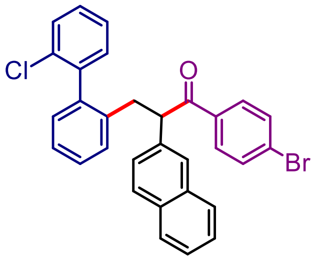

5k, 76%, dr = 1:1

Compound, 5k: yellow liquid; eluent (5% ethyl acetate in hexane). Yield: 76% (80 mg); **^{1}H NMR (500 MHz, DMSO-*d$_6$*) δ**: 7.79 - 7.73 (m, 8H), 7.70 – 7.65 (m, 2H), 7.60 – 7.58 (m, 4H), 7.54 – 7.51 (m, 3H), 7.48 – 7.46 (m, 2H), 7.43 – 7.41 (m, 5H), 7.38 – 7.34 (m, 3H), 7.29 – 7.16 (m, 6H), 7.10 (t, *J* = 7.8 Hz, 2H), 7.02 (d, *J* = 8.4 Hz, 1H), 6.98 (d, *J* = 7.6 Hz, 1H), 6.65 (d, *J* = 7.5 Hz, 1H), 5.02 – 4.98 (m, 2H), 3.42 – 3.39 (m, 1H), 3.30 (dd, *J* = 14.3, 7.7 Hz, 1H), 3.00 (dd, *J* = 14.3, 6.5 Hz, 1H), 2.85 (dd, *J* = 14.1, 8.2 Hz, 1H). **^{13}C NMR (126 MHz, DMSO-*d$_6$*) δ**: 197.9, 197.6, 139.6, 139.4, 139.0, 138.9, 137.2, 137.0, 136.1, 135.9, 134.8, 134.7, 133.0, 132.9, 132.4, 132.3, 132.0, 131.88, 131.85, 131.8, 131.4, 130.4, 130.3, 129.8, 129.6, 129.52, 129.50, 129.4, 129.23, 129.19, 128.6, 128.4, 127.9, 127.8, 127.6, 127.5, 127.44, 127.36, 126.9, 126.7, 126.6, 126.4, 126.31, 126.27, 126.12, 126.07, 126.0, 125.9, 53.5, 52.5, 36.6, 36.0. **HRMS (ESI/TOF-Q)** m/z: [M+H]+ Calculated for $C_{31}H_{22}BrClOH^+$ 525.0615; Found 525.0625.

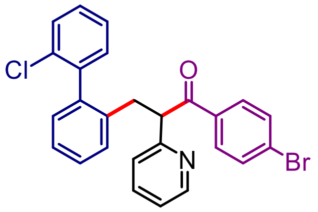

5l, 66%, dr = 1:1

Compound, 5l: pale yellow sticky liquid; eluent (2% ethyl acetate in hexane). Yield: 66% (63 mg); **^{1}H NMR (500 MHz, DMSO-*d$_6$*) δ**: 8.33 (d, *J* = 4.8 Hz, 1H), 8.24 (d, *J* = 4.8 Hz, 1H), 7.71 – 7.68 (m, 4H), 7.63 (t, *J* = 7.8 Hz, 1H), 7.59 - 7.55 (m, 6H), 7.53 - 7.40 (m, 5H), 7.36 (t, *J* = 7.8 Hz, 1H), 7.29 (t, *J* = 7.7 Hz, 1H), 7.26 – 7.18 (m, 5H), 7.16 – 7.06 (m, 4H), 6.99 (d, *J* = 7.6 Hz, 1H), 6.92 (d, *J* = 7.8 Hz, 1H), 6.67 (d, *J* = 7.5 Hz, 1H), 5.03 – 4.99 (m, 2H), 3.35 – 3.34 (m, 1H), 3.23 (dd, *J* = 14.3, 7.0 Hz, 1H), 3.01 (dd, *J* = 14.3, 7.3 Hz, 1H), 2.84 (dd, *J* = 14.0, 8.9 Hz, 1H). **^{13}C NMR (126 MHz, DMSO-*d$_6$*) δ**: 196.6, 196.5, 158.2, 149.29, 149.26, 139.5, 139.4, 139.0, 138.9, 137.1, 137.0, 136.8, 135.1, 135.0, 132.30, 132.26, 131.7, 131.6, 131.2, 130.3, 130.2, 129.7, 129.39, 129.36, 129.12, 129.09, 127.8, 127.7, 127.21, 127.18, 127.0, 126.2, 123.4, 123.3, 122.2, 122.0, 55.9, 54.9, 34.9, 34.4. **HRMS (ESI/TOF-Q) m/z**: [M+H]+ Calculated for $C_{26}H_{19}BrClNOH^+$ 476.0411; Found 476.0416.

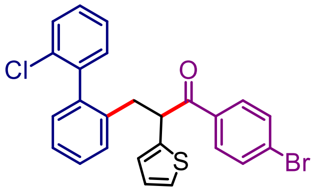

5m, 61%, dr = 1:1

Compound, 5m: pale yellow sticky liquid; eluent (1% ethyl acetate in hexane). Yield: 61% (59 mg); **^{1}H NMR (500 MHz, CDCl$_3$) δ**: 7.57 – 7.55 (m, 4H), 7.52 (d, *J* = 7.8 Hz, 1H), 7.48 -7.44 (m, 4H), 7.41 – 7.27 (m, 6H), 7.24 (d, *J* = 7.3 Hz, 2H), 7.22 – 7.18 (m, 2H), 7.16 – 7.13 (m, 3H), 7.11 - 7.05 (m, 4H), 6.80 - 6.78 (m, 2H), 6.56– 6.55 (m, 2H), 4.80 (dd, *J* = 8.1, 6.2 Hz, 1H), 4.75 (dd, *J* = 8.6, 5.8 Hz, 1H), 3.47 – 3.37 (m, 2H), 3.03 – 2.93 (m, 2H). **^{13}C NMR (126 MHz, CDCl$_3$) δ**: 197.6, 197.3, 141.1, 140.5, 140.4, 140.2, 139.4, 139.3, 137.0, 136.9, 135.2, 135.1, 133.7, 133.6, 132.0, 131.9, 131.7, 131.6, 130.7, 130.3, 130.2, 130.14, 130.05, 129.9, 129.2, 129.1, 128.4, 128.2, 128.0, 127.1, 127.00, 126.96, 126.9, 126.7, 126.4, 125.7, 125.4, 125.2, 49.0, 48.5, 39.0, 38.8. **HRMS (ESI/TOF-Q) m/z**: [M+Na]+ Calculated for $C_{25}H_{18}BrClOSNa^+$ 502.9842; Found 502.9818.

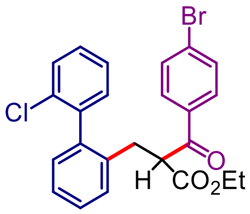

5n, 78%, dr = 1:1

Compound, 5n: yellow sticky liquid; eluent (1% ethyl acetate in hexane). Yield: 78% (74 mg); **^{1}H NMR (500 MHz, CDCl$_3$) δ**: 7.48 – 7.43 (m, 9H), 7.34 – 7.30 (m, 7H), 7.27 – 7.24 (m, 5H), 7.20 (d, *J* = 6.6 Hz, 1H), 7.13 – 7.09 (m, 2H), 4.29 – 4.23 (m, 2H), 4.02 – 3.96 (m, 4H), 3.23 – 3.11 (m, 4H), 1.05-1.02 (m, 6H). **^{13}C NMR (126 MHz, CDCl$_3$) δ**: 194.0, 193.8, 168.92, 168.86, 140.1, 140.0, 139.4, 139.3, 136.2, 136.1, 134.8, 134.7, 133.7, 133.6, 132.1, 132.0, 131.6, 131.5, 130.7, 130.6, 130.5, 130.23, 130.16, 130.0, 129.9, 129.21, 129.16, 128.8, 128.32, 128.28, 127.0, 126.9, 61.5, 54.7, 54.6, 32.8, 32.6, 14.0. **HRMS (ESI/TOF-Q) m/z**: [M+H]+ Calculated for $C_{24}H_{20}BrClO_3H^+$ 471.0357; Found 471.0355.

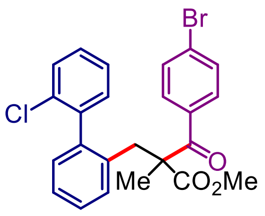

5o, 76%, dr = 1:1

Compound, 5o: white colored liquid; eluent (1% ethyl acetate in hexane). Yield: 76% (72 mg); **^{1}H NMR (400 MHz, CDCl$_3$) δ**: 7.54 (d, *J* = 8.5 Hz, 2H), 7.44 – 7.41 (m, 4H), 7.36 (d, *J* = 8.6 Hz, 2H), 7.31 (d, *J* = 6.8 Hz, 1H), 7.27 – 7.25 (m, 1H), 7.19 – 7.11 (m, 7H), 7.09 – 6.97 (m, 6H), 6.87 (d, *J* = 7.5 Hz, 1H), 3.48 – 3.41 (m, 8H), 3.24 (d, *J* = 14.5 Hz, 2H), 1.10 (s, 3H), 1.08 (s, 3H). **^{13}C NMR (101 MHz, CDCl$_3$) δ**: 195.87, 195.85, 174.5, 174.3, 140.54, 140.47, 140.0, 134.5, 134.4, 134.3, 134.2, 133.7, 133.6, 132.5, 132.3, 132.0, 131.8, 131.0, 130.64, 130.60, 130.5, 130.2, 129.94, 129.87, 129.5, 128.84, 128.78, 128.0, 127.88, 127.85, 127.7, 127.1, 126.9, 126.6, 58.5, 58.4, 52.63, 52.58, 38.2, 37.9, 21.0, 20.7. **HRMS (ESI/TOF-Q) m/z**: [M+H]+ Calculated for $C_{24}H_{20}BrClO_3H^+$ 471.0357; Found 471.0356.

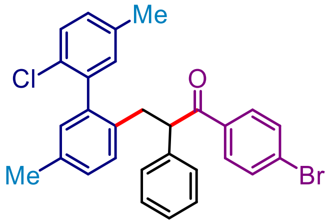

5p, 71%, dr = 1:1

Compound, 5p: yellow sticky liquid; eluent (1% ethyl acetate in hexane). Yield: 71% (72 mg); **^{1}H NMR (500 MHz, CDCl$_3$) δ:** 7.53 – 7.50 (m, 4H), 7.36 – 7.33 (m, 4H), 7.24 (d, *J* = 8.1 Hz, 1H), 7.17 (s, 1H), 7.08 – 6.99 (m, 10H), 6.91 (d, *J* = 7.5 Hz, 2H), 6.86 – 6.85 (m, 2H), 6.82 – 6.80 (m, 5H), 6.64 (s, 1H), 4.48 (dd, *J* = 8.7, 5.0 Hz, 1H), 4.42 – 4.39 (m, 1H), 3.36 – 3.25 (m, 2H), 2.82 – 2.77 (m, 2H), 2.25 (s, 3H), 2.21 (s, 9H). **^{13}C NMR (126 MHz, CDCl$_3$) δ**: 198.6, 198.2, 140.2, 140.1, 139.5, 139.4, 139.2, 138.7, 136.8, 136.5, 135.82, 135.78, 135.6, 135.4, 134.6, 134.3, 132.24, 132.20, 131.84, 131.75, 130.8, 130.6, 130.5, 130.3, 130.2, 129.7, 129.6, 129.3, 129.0, 128.9, 128.7, 128.6, 128.4, 127.99, 127.97, 127.2, 127.1, 54.6, 54.1, 37.5, 37.3, 21.09, 21.06, 21.0. **HRMS (ESI/TOF-Q) m/z**: [M+H]+ Calculated for $C_{29}H_{24}BrClOH^+$ 503.0772; Found 503.0770.

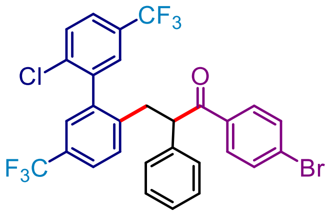

5q, 66%, dr = 1:1

Compound, 5q: yellow liquid; eluent (1% ethyl acetate in hexane). Yield: 66% (40 mg, (0.1 mmol scale)); **^{1}H NMR (400 MHz, CDCl$_3$) δ:** 7.71 – 7.56 (m, 9H), 7.51 – 7.44 (m, 5H), 7.40 (d, *J* = 8.1 Hz, 2H), 7.32 – 7.30 (m, 2H), 7.22 – 7.10 (m, 8H), 6.94 – 6.91 (m, 2H), 6.82 – 6.79 (m, 2H), 4.53 – 4.48 (m, 2H), 3.50 – 3.39 (m, 2H), 2.98 (dd, *J* = 14.2, 5.9 Hz, 1H), 2.90 (dd, *J* = 14.0, 8.0 Hz, 1H). **^{13}C NMR (101 MHz, CDCl$_3$) δ**: 197.4, 197.3, 142.2, 142.0, 139.9, 139.7, 138.9, 138.5, 138.3, 137.9, 135.2, 135.1, 132.7, 132.1, 132.0, 131.4, 131.3, 131.2, 130.7, 130.5, 130.23, 130.18, 129.4, 129.3, 128.5, 128.22, 128.19, 128.15, 128.0, 127.9, 127.8, 127.7, 127.0 (q, *J* = 3.4 Hz), 126.8 (q, *J* = 3.5 Hz), 126.5 – 126.4 (m), 125.5 (q, *J* = 3.4 Hz), 122.7, 122.4, 55.1, 54.0, 37.5, 37.2. **^{19}F NMR (376 MHz, CDCl$_3$) δ:** -62.27, -62.45, -62.50, -62.51. **HRMS (ESI/TOF-Q) m/z**: [M+H]+ Calculated for $C_{29}H_{18}BrClF_6OH^+$ 611.0207; Found 611.2090.

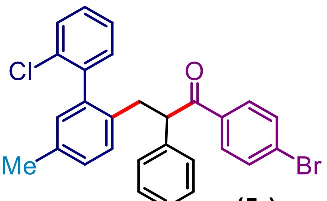

(5r)

+

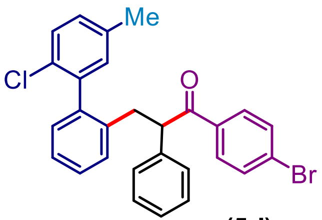

(5r')

71%, 1:1 rr

Compound, 5r and 5r': brown sticky liquid; eluent (1% ethyl acetate in hexane). Yield: 71% (72 mg); **^{1}H NMR (500 MHz, $CDCl_3$) δ:** 7.74 – 7.69 (m, 8H), 7.58 – 7.53 (m, 8H), 7.46 – 7.39 (m, 4H), 7.37 (s, 2H), 7.33 – 7.24 (m, 22H), 7.22 – 7.20 (m, 4H), 7.17 – 7.08 (m, 6H), 7.03 – 7.00 (m, 8H), 6.85 (s, 2H), 4.72 – 4.57 (m, 4H), 3.61 – 3.49 (m, 4H), 3.07 – 2.96 (m, 4H), 2.46 – 2.42 (m, 12H). **^{13}C NMR (126 MHz, $CDCl_3$) δ**: 198.5, 198.4, 198.1, 140.6, 140.4, 140.1, 139.9, 139.6, 139.4, 139.3, 139.1, 138.7, 138.5, 137.7, 137.4, 136.8, 136.5, 135.9, 135.5, 135.4, 135.3, 134.5, 134.4, 133.6, 133.4, 132.20, 132.17, 131.9, 131.8, 131.7, 131.6, 130.84, 130.76, 130.62, 130.58, 130.30, 130.28, 130.21, 130.18, 130.0, 129.9, 129.8, 129.7, 129.6, 129.4, 129.0, 128.94, 128.88, 128.82, 128.78, 128.7, 128.4, 128.3, 128.04, 128.00, 127.9, 127.8, 127.21, 127.17, 127.0, 126.8, 126.33, 126.30, 54.7, 54.6, 54.1, 54.0, 37.9, 37.6, 37.5, 37.3, 21.09, 21.06, 21.0. **HRMS (ESI/TOF-Q) m/z**: [M+Na]+ Calculated for $C_{28}H_{22}BrClONa^{+}$ 511.0435; Found 511.0408.

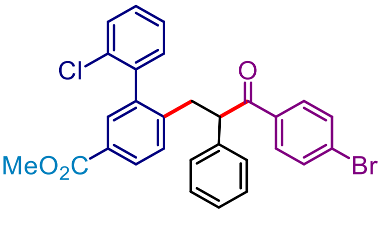

5s

+

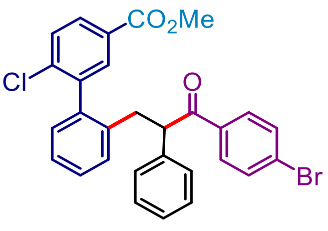

5s'

68%, 1:1 rr

Compound, 5s and 5s': yellow liquid; eluent (3% ethyl acetate in hexane). Yield: 68% (73 mg); **^{1}H NMR (400 MHz, $CDCl_3$) δ:** 8.03 – 7.99 (m, 2H), 7.97 – 7.94 (m, 1H), 7.88 – 7.85 (m, 2H), 7.84 – 7.79 (m, 2H), 7.63 – 7.58 (m, 10H), 7.54 – 7.41 (m, 11H), 7.39 – 7.28 (m, 6H), 7.25 – 7.21 (m, 4H), 7.20 – 7.10 (m, 16H), 7.08 – 7.05 (m, 1H), 6.95 – 6.84 (m, 9H), 4.57 – 4.46 (m, 4H), 3.93 - 3.87 (m, 12H), 3.51 – 3.37 (m, 4H), 3.00 – 2.85 (m, 4H). **^{13}C NMR (101 MHz, $CDCl_3$) δ**: 198.1, 197.9, 197.6, 166.94, 166.92, 166.2, 143.1, 140.6, 140.5, 139.7, 139.5, 139.4, 139.2, 138.8, 138.74, 138.73, 138.6, 138.5, 138.33, 138.28, 138.1, 137.7, 137.6, 135.3, 135.22, 135.19, 135.1, 133.6, 133.4, 132.8, 132.7, 131.90, 131.85, 131.8, 131.6, 131.49, 131.47, 131.3, 131.0, 130.9, 130.7, 130.6, 130.24, 130.21, 130.18, 130.0, 129.91, 129.88, 129.8, 129.5, 129.3, 129.2, 129.14, 129.11, 129.02, 128.98, 128.8, 128.42, 128.40, 128.37, 128.3, 128.1, 128.0, 127.9, 127.42, 127.37, 127.3, 127.1, 127.0, 126.54, 126.52, 54.9, 54.4, 54.3, 53.7, 52.5, 52.4, 52.2, 37.9, 37.64, 37.61, 37.5. **HRMS (ESI/TOF-Q) m/z**: [M+Na]+ Calculated for $C_{29}H_{22}BrClO_3Na^{+}$ 555.0333; Found 555.0294

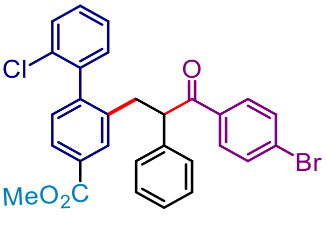

5t, 1 : 1 rr

Compound, 5t: colorless sticky liquid; eluent (3% ethyl acetate in hexane). Yield: 33% (35 mg); **^{1}H NMR (500 MHz, $CDCl_3$) δ:** 7.94 (d, *J* = 1.7 Hz, 1H), 7.89 (d, *J* = 7.8 Hz, 2H), 7.83 (d, *J* = 1.7 Hz, 1H), 7.62 – 7.57 (m, 5H), 7.47 – 7.36 (m, 7H), 7.34 – 7.29 (m, 2H), 7.22 (d, *J* = 8.0 Hz, 2H), 7.20 – 7.12 (m, 7H), 6.89 – 6.87 (m, 2H), 6.85 – 6.83 (m, 2H), 6.75 (dd, *J* = 7.5, 1.5 Hz, 1H), 4.57 (dd, *J* = 9.0, 5.2 Hz, 1H), 4.51 (dd, *J* = 8.7, 5.6 Hz, 1H), 3.91 – 3.89 (m, 6H), 3.50 – 3.42 (m, 2H), 3.00 – 2.93 (m, 2H). **^{13}C NMR (126 MHz, $CDCl_3$) δ**: 197.9, 197.6, 166.99, 166.96, 144.2, 143.9, 139.5, 139.2, 138.8, 138.2, 138.1, 135.4, 135.1, 133.3, 133.1, 131.9, 131.80, 131.75, 131.6, 131.2, 131.1, 130.6, 130.30, 130.25, 130.0, 129.8, 129.7, 129.5, 129.3, 129.2, 129.1, 128.3, 128.13, 128.11, 127.9, 127.6, 127.5, 127.38, 127.35, 127.1, 126.8, 54.8, 53.9, 52.2, 37.6, 37.2. **HRMS (ESI/TOF-Q) m/z**: [M+H]+ Calculated for $C_{29}H_{22}BrClO_3H^{+}$ 533.0514; Found 533.0540.

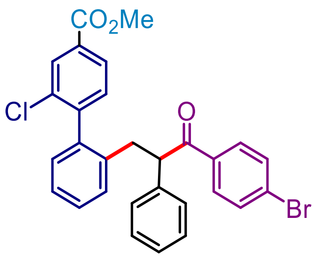

5t', 1 : 1 rr

Compound, 5t': colorless sticky liquid; eluent (3% ethyl acetate in hexane). Yield: 32% (34 mg); **^{1}H NMR (500 MHz, $CDCl_3$) δ:** 8.23 (d, *J* = 1.6 Hz, 1H), 8.11 (d, *J* = 1.7 Hz, 1H), 8.00 (dd, *J* = 7.8, 1.7 Hz, 1H), 7.85 (dd, *J* = 7.8, 1.7 Hz, 1H), 7.62 – 7.59 (m, 4H), 7.45 – 7.42 (m, 5H), 7.26 – 7.23 (m, 5H), 7.18 – 7.09 (m, 8H), 7.06 – 7.04 (m, 1H), 6.91 – 6.85 (m, 5H), 4.55 (dd, *J* = 9.0, 5.1 Hz, 1H), 4.46 (dd, *J* = 8.8, 5.4 Hz, 1H), 3.99 – 3.96 (m, 6H), 3.45 – 3.37 (m, 2H), 2.92 – 2.83 (m, 2H). **^{13}C NMR (126 MHz, $CDCl_3$) δ**: 198.1, 197.8, 165.91, 165.88, 145.1, 144.8, 138.9, 138.7, 138.4, 138.3, 137.4, 137.3, 135.4, 135.1, 134.0, 133.8, 131.90, 131.85, 131.8, 131.6, 131.1, 131.0, 130.9, 130.7, 130.5, 130.3, 130.2, 129.8, 129.6, 129.2, 129.1, 128.5, 128.4, 128.3, 128.17, 128.15, 128.0, 127.9, 127.8, 127.4, 127.3, 126.5, 55.0, 54.2, 52.7, 52.6, 37.6, 37.3. **HRMS (ESI/TOF-Q) m/z**: [M+H]+ Calculated for $C_{29}H_{22}BrClO_3H^+$ 533.0514; Found 533.0540.

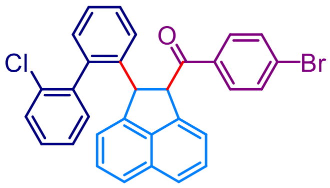

7a, 71%, dr = 1:1

Compound, 7a: orange colored solid; eluent (1% ethyl acetate in hexane). Yield: 71% (74 mg); **^{1}H NMR (500 MHz, $CDCl_3$) δ:** 7.84 (d, *J* = 8.5 Hz, 2H), 7.71 – 7.68 (m, 6H), 7.63 (d, *J* = 8.5 Hz, 2H), 7.59 (d, *J* = 8.5 Hz, 2H), 7.56 – 7.49 (m, 3H), 7.42 – 7.28 (m, 8H), 7.25 – 7.22 (m, 4H), 7.20 – 7.12 (m, 3H), 6.97 – 6.91 (m, 3H), 6.87 – 6.84 (m, 2H), 6.76 (d, *J* = 7.2 Hz, 1H), 5.49 (s, 1H), 5.39 (d, *J*= 2.3 Hz, 2H), 5.25 (d, *J*= 3.3 Hz, 1H). **^{13}C NMR (126 MHz, $CDCl_3$) δ**: 195.9, 195.1, 147.6, 147.2, 143.4, 143.3, 141.6, 141.5, 139.9, 139.7, 139.5, 139.3, 138.1, 137.9, 135.4, 135.3, 133.7, 133.4, 132.1, 132.0, 131.9, 131.8, 131.7, 131.4, 131.0, 130.9, 130.0, 129.62, 129.59, 129.2, 129.0, 128.9, 128.80, 128.76, 128.7, 128.6, 128.53, 128.46, 128.1, 127.9, 127.2, 126.9, 126.6, 126.5, 124.40, 124.35, 123.5, 123.4, 121.5, 121.3, 120.5, 120.2, 62.5, 61.7, 50.6, 49.0. **HRMS (ESI/TOF-Q) m/z**: [M+H]+ Calculated for $C_{31}H_{20}BrClOH^+$ 523.0459; Found 523.0462.

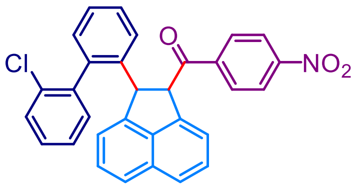

7b, 75%, dr = 1:1

Compound, 7b: white colored solid; eluent (5% ethyl acetate in hexane). Yield: 75% (74 mg); **^{1}H NMR (400 MHz, $CDCl_3$) δ:** 8.32 (d, *J* = 8.9 Hz, 2H), 8.25 (d, *J* = 8.9 Hz, 2H), 8.09 (d, *J* = 8.9 Hz, 2H), 7.90 (d, *J* = 8.8 Hz, 2H), 7.74 -7.70 (m, 4H), 7.59 – 7.50 (m, 3H), 7.39 – 7.29 (m, 8H), 7.26 – 7.09 (m, 7H), 6.97 (d, *J* = 7.3 Hz, 1H), 6.90 – 6.83 (m, 4H), 6.79 (d, *J* = 6.7 Hz, 1H), 5.50 (d, *J*= 2.3 Hz, 1H), 5.43-5.41 (m, 2H), 5.26 (d, *J*= 3.5 Hz, 1H). **^{13}C NMR (101 MHz, $CDCl_3$) δ**: 195.5, 194.7, 150.5, 150.4, 147.2, 146.8, 143.02, 142.96, 141.3, 141.1, 140.9, 140.8, 139.9, 139.7, 139.4, 139.2, 138.1, 137.9, 133.6, 133.5, 132.0, 131.9, 131.7, 131.5, 130.4, 130.23, 130.15, 130.1, 129.7, 129.6, 129.3, 129.0, 128.9, 128.6, 128.1, 127.9, 127.3, 127.1, 126.7, 126.5, 124.72, 124.68, 123.9, 123.7, 123.6, 121.7, 121.5, 120.5, 120.2, 63.3, 62.3, 50.7, 49.1. **HRMS (ESI/TOF-Q) m/z**: [M+H]+ Calculated for $C_{31}H_{20}ClNO_3H^+$ 490.1204; Found 490.1216.

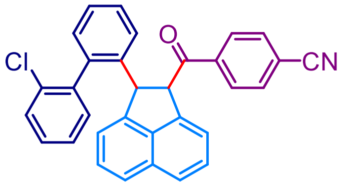

7c, 70%, dr = 1:1

Compound, 7c: white colored solid; eluent (3% ethyl acetate in hexane). Yield: 70% (69 mg); **^{1}H NMR (400 MHz, $CDCl_3$) δ:** 8.01 (d, *J* = 8.0 Hz, 2H), 7.84 (d, *J* = 8.1 Hz, 2H), 7.77 (d, *J* = 8.0 Hz, 2H), 7.73 – 7.69 (m, 6H), 7.58 - 7.52 (m, 2H), 7.49 (d, *J* = 7.6 Hz, 1H), 7.39 – 7.28 (m, 8H), 7.26 – 7.22 (m, 4H), 7.20 – 7.09 (m, 3H), 6.97 (d, *J* = 7.4 Hz, 1H), 6.89 – 6.81 (m, 4H), 6.76 (d, *J* = 7.4 Hz, 1H), 5.47 (d, *J*= 2.9 Hz, 1H), 5.39 (s, 2H), 5.23 (d, *J*= 3.2 Hz, 1H). **^{13}C NMR (101 MHz, $CDCl_3$) δ**: 195.8, 195.0, 147.3, 146.9, 143.1, 143.0, 141.1, 139.93, 139.87, 139.73, 139.67, 139.5, 139.3, 138.2, 137.9, 133.7, 133.5, 132.5, 132.0, 131.9, 131.7, 131.5, 130.2, 130.1, 129.8, 129.7, 129.6, 129.3, 129.0, 128.92, 128.89, 128.6, 128.1, 127.9, 127.3, 127.1, 126.7, 126.4, 124.7, 124.6, 123.7, 123.6, 121.7, 121.4, 120.5, 120.2, 118.1, 117.9, 116.6, 63.2, 62.2, 50.8, 49.2. **HRMS (ESI/TOF-Q) m/z**: [M+Na]+ Calculated for $C_{32}H_{20}ClNONa^+$ 492.1126; Found 492.1095.

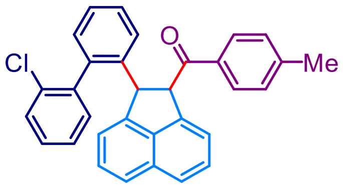

7d, 42%, dr = 1:1

Compound, 7d: brown colored solid; eluent (1% ethyl acetate in hexane). Yield: 42% (36 mg); **^{1}H NMR (400 MHz, $CDCl_3$) δ:** 7.88 (d, *J* = 8.2 Hz, 2H), 7.77 (d, *J* = 8.2 Hz, 2H), 7.68 – 7.64 (m, 4H), 7.54 – 7.48 (m, 3H), 7.41 (d, *J* = 8.0 Hz, 1H), 7.38 – 7.36 (m, 1H), 7.34 – 7.32 (m, 3H), 7.31 – 7.29 (m, 3H), 7.28 – 7.27 (m, 2H), 7.25 – 7.23 (m, 3H), 7.22 – 7.17 (m, 4H), 7.15 – 7.11 (m, 2H), 6.99 – 6.94 (m, 3H), 6.88 – 6.82 (m, 2H), 6.74 (d, *J* = 7.5 Hz, 1H), 5.49 (d, *J* = 2.8 Hz, 1H), 5.45 (d, *J* = 2.4 Hz, 1H), 5.38 (d, *J* = 3.5 Hz, 1H), 5.33 (d, *J* = 3.5 Hz, 1H), 2.45 (s, 3H), 2.44 (s, 3H). **^{13}C NMR (101 MHz, $CDCl_3$) δ**: 196.6, 195.9, 148.2, 147.8, 144.1, 143.9, 143.7, 142.3, 140.0, 139.8, 139.6, 139.4, 138.3, 138.1, 134.5, 134.3, 134.0, 133.5, 131.92, 131.88, 131.8, 131.4, 130.1, 130.0, 129.71, 129.69, 129.6, 129.5, 129.4, 129.1, 128.9, 128.8, 128.72, 128.65, 128.6, 128.3, 127.91, 127.88, 127.0, 126.7, 126.5, 126.4, 124.2, 124.1, 123.33, 123.30, 121.4, 121.1, 120.6, 120.2, 62.3, 61.8, 50.7, 49.1, 21.8. **HRMS (ESI/TOF-Q) m/z**: [M+H]+ Calculated for $C_{32}H_{23}ClOH^{+}$ 459.1510; Found 459.1510.

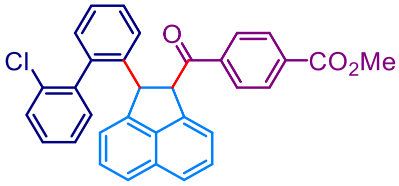

7e, 72%, dr = 1:1

Compound, 7e: white colored solid; eluent (5% ethyl acetate in hexane). Yield: 72% (72 mg); **^{1}H NMR (500 MHz, $CDCl_3$) δ:** 8.14 (d, *J* = 8.4 Hz, 2H), 8.09 (d, *J* = 8.3 Hz, 2H), 8.01 (d, *J* = 8.3 Hz, 2H), 7.87 (d, *J* = 8.3 Hz, 2H), 7.70 – 7.65 (m, 4H), 7.55 – 7.48 (m, 3H), 7.40 (d, *J* = 8.0 Hz, 1H), 7.36 (t, *J* = 7.1 Hz, 2H), 7.32 – 7.27 (m, 5H), 7.24 – 7.20 (m, 4H), 7.18 – 7.17 (m, 1H), 7.14 – 7.09 (m, 2H), 6.95 (d, *J* = 7.4 Hz, 1H), 6.89 (t, *J* = 6.4 Hz, 2H), 6.85 – 6.80 (m, 2H), 6.75 (d, *J* = 7.1 Hz, 1H), 5.50 (s, 1H), 5.44 (s, 1H), 5.41 (d, *J* = 3.5 Hz, 1H), 5.30 (d, *J* = 3.6 Hz, 1H), 3.96 (s, 3H), 3.95 (s, 3H). **^{13}C NMR (126 MHz, $CDCl_3$) δ:** 196.3, 195.5, 166.4, 166.3, 147.5, 147.1, 143.34, 143.27, 141.4, 141.3, 139.9, 139.8, 139.6, 139.5, 139.2, 138.1, 137.9, 134.02, 133.98, 133.7, 133.4, 131.9, 131.8, 131.6, 131.4, 130.0, 129.9, 129.8, 129.6, 129.4, 129.22, 129.20, 129.0, 128.9, 128.78, 128.75, 128.7, 128.5, 128.1, 127.9, 127.1, 126.9, 126.6, 126.5, 124.41, 124.36, 123.5, 123.4, 121.6, 121.3, 120.6, 120.2, 62.70, 62.00, 52.60, 52.57, 50.5, 48.9.**HRMS (ESI/TOF-Q) m/z**: [M+H]+ Calculated for $C_{33}H_{23}ClO_3H^{+}$ 503.1408; Found 503.1427.

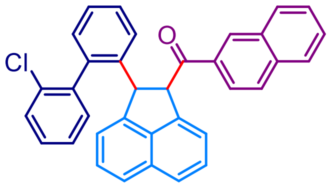

7f, 68%, dr = 1:1

Compound, 7f: white colored solid; eluent (2% ethyl acetate in hexane). Yield: 68% (67 mg); **^{1}H NMR (500 MHz, $CDCl_3$) δ:** 8.59 (s, 1H), 8.42 (s, 1H), 8.01 – 7.98 (m, 2H), 7.94 – 7.89 (m, 6H), 7.71 – 7.63 (m, 6H), 7.60 – 7.52 (m, 5H), 7.42 (d, *J* = 8.0 Hz, 1H), 7.39 – 7.36 (m, 2H), 7.34 – 7.27 (m, 7H), 7.25 – 7.24 (m, 1H), 7.22 – 7.18 (m, 2H), 7.12 – 7.09 (m, 2H), 7.05 – 7.03 (m, 1H), 6.97 (d, *J* = 7.0 Hz, 2H), 6.92 – 6.91 (m, 1H), 6.75 (t, *J* = 7.4 Hz, 1H), 6.68 (d, *J* = 7.3 Hz, 1H), 5.64 (d, *J* = 2.7 Hz, 1H), 5.59 (d, *J* = 3.0 Hz, 1H), 5.52 (d, *J* = 3.5 Hz, 1H), 5.46 (d, *J* = 3.5 Hz, 1H). **^{13}C NMR (126 MHz, $CDCl_3$) δ**: 196.8, 196.0, 147.9, 147.6, 143.71, 143.65, 142.09, 142.06, 139.9, 139.7, 139.6, 139.4, 138.3, 138.1, 135.8, 134.3, 134.1, 133.9, 133.4, 132.8, 131.9, 131.8, 131.4, 131.3, 131.2, 130.13, 130.10, 129.88, 129.85, 129.7, 129.6, 129.2, 129.0, 128.81, 128.78, 128.74, 128.71, 128.66, 128.6, 128.5, 128.3, 127.94, 127.91, 127.1, 127.0, 126.9, 126.8, 126.5, 126.4, 125.3, 125.0, 124.3, 124.2, 123.40, 123.38, 121.4, 121.3, 120.7, 120.4, 62.3, 61.8, 50.8, 49.1. **HRMS (ESI/TOF-Q) m/z**: [M+H]+ Calculated for $C_{35}H_{23}ClOH^{+}$ 495.1510; Found 495.1510.

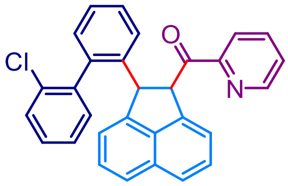

7g, 72%, dr = 1:1

Compound, 7g: brown colored solid; eluent (5% ethyl acetate in hexane). Yield: 72% (74 mg); **^{1}H NMR (500 MHz, $CDCl_3$) δ:** 8.82 (dd, *J* = 14.5, 4.5 Hz, 2H), 8.01 (d, *J* = 7.8 Hz, 1H), 7.91 (d, *J* = 7.8 Hz, 1H), 7.83 (t, *J* = 7.6 Hz, 2H), 7.70 – 7.65 (m, 4H), 7.57 – 7.48 (m, 6H), 7.41 – 7.38 (m, 2H), 7.36 – 7.27 (m, 4H), 7.24 – 7.18 (m, 8H), 7.14 (d, *J* = 6.9 Hz, 1H), 6.98 (d, *J* = 6.9 Hz, 1H), 6.92 (dd, *J* = 14.4, 7.5 Hz, 2H), 6.85 (d, *J* = 7.7 Hz, 1H), 6.79 (t, *J* = 7.5 Hz, 1H), 6.24 (s, 1H), 6.06 (s, 1H), 5.46 (s, 2H). **^{13}C NMR (126 MHz, $CDCl_3$) δ**: 196.94, 196.91, 153.2, 149.2, 149.1, 148.4, 147.5, 144.0, 143.9, 142.5, 142.1, 140.21, 140.18, 139.3, 139.0, 138.3, 137.9,

137.0, 136.9, 134.0, 133.5, 131.8, 131.74, 131.72, 131.5, 129.7, 129.59, 129.56, 129.4, 129.1, 128.9, 128.7, 128.62, 128.60, 128.5, 128.4, 128.2, 128.0, 127.9, 127.2, 127.1, 127.0, 126.5, 126.32, 126.29, 123.91, 123.88, 123.6, 123.24, 123.19, 121.6, 121.4, 120.90, 120.88, 60.5, 49.2, 48.4. **HRMS (ESI/TOF-Q) m/z**: [M+H]+ Calculated for $C_{30}H_{20}ClNOH^+$ 446.1306; Found 446.1311.

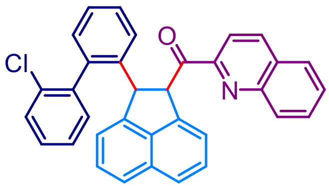

7h, 76%, dr = 1:1

Compound, 7h: yellow colored solid; eluent (5% ethyl acetate in hexane). Yield: 76% (75 mg); **^{1}H NMR (500 MHz, CDCl$_3$) δ:** 8.32 (d, J = 8.5 Hz, 1H), 8.27 (d, J = 8.5 Hz, 2H), 8.25 (d, J = 8.5 Hz, 1H), 8.07 (d, J = 8.5 Hz, 1H), 7.98 (d, J = 8.5 Hz, 1H), 7.93 (dd, J = 7.9, 4.7 Hz, 2H), 7.84 – 7.81 (m, 2H), 7.73 – 7.64 (m, 6H), 7.59 – 7.49 (m, 4H), 7.42 – 7.38 (m, 2H), 7.32 – 7.27 (m, 6H), 7.24 – 7.17 (m, 5H), 7.14 (d, J = 8.0 Hz, 2H), 7.05 (d, J = 6.9 Hz, 1H), 6.97 – 6.96 (m, 1H), 6.92 (d, J = 7.6 Hz, 1H), 6.84 (d, J = 7.5 Hz, 1H), 6.66 (t, J = 7.5 Hz, 1H), 6.45 (s, 1H), 6.28 (s, 1H), 5.54 (s, 1H), 5.51 (s, 1H). **^{13}C NMR (126 MHz, CDCl$_3$) δ**: 197.2, 197.0, 152.9, 152.8, 148.4, 147.7, 147.4, 144.2, 143.9, 142.8, 142.4, 140.3, 140.2, 139.5, 139.2, 138.5, 138.1, 136.9, 136.8, 134.1, 133.6, 131.9, 131.83, 131.76, 131.5, 131.0, 130.9, 130.2, 130.1, 129.8, 129.72, 129.65, 129.5, 129.0, 128.92, 128.87, 128.8, 128.7, 128.6, 128.43, 128.39, 128.0, 127.9, 127.80, 127.77, 127.0, 126.5, 126.32, 126.29, 123.9, 123.3, 123.2, 121.6, 121.5, 121.2, 121.0, 119.60, 119.56, 60.62, 60.58, 49.7, 48.7. **HRMS (ESI/TOF-Q) m/z**: [M+H]+ Calculated for $C_{34}H_{22}ClNOH^+$ 496.1463; Found 496.1457.

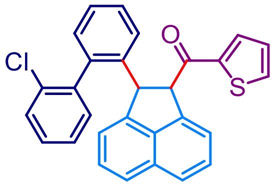

7i, 65%, dr = 1:1

Compound, 7i: white colored solid; eluent (2% ethyl acetate in hexane). Yield: 65% (59 mg); **^{1}H NMR (500 MHz, CDCl$_3$) δ:** 7.81 (d, J = 3.7 Hz, 1H), 7.71 – 7.68 (m, 5H), 7.67 – 7.65 (m, 1H), 7.56 – 7.53 (m, 2H), 7.51 – 7.48 (m, 2H), 7.41 – 7.28 (m, 9H), 7.25 – 7.18 (m, 6H), 7.16 – 7.07 (m, 4H), 7.00 (d, J = 7.2 Hz, 1H), 6.94 – 6.89 (m, 2H), 6.79 (d, J = 7.1 Hz, 1H), 5.47 (d, J = 2.4 Hz, 1H), 5.32 (d, J = 3.8 Hz, 1H), 5.25 (d, J = 2.5 Hz, 1H), 5.11 (d, J = 3.7 Hz, 1H). **^{13}C NMR (126 MHz, CDCl$_3$) δ**: 190.4, 189.3, 147.6, 147.3, 144.3, 144.2, 143.3, 143.2, 141.94, 141.92, 139.7, 139.6, 139.5, 139.3, 138.3, 138.0, 134.5, 133.8, 133.3, 133.1, 132.9, 131.8, 131.7, 131.3, 130.1, 129.9, 129.6, 129.5, 129.3, 129.0, 128.8, 128.7, 128.6, 128.5, 128.3, 128.2, 128.99, 127.96, 127.1, 126.8, 126.5, 124.33, 124.28, 123.40, 123.36, 121.3, 121.2, 120.5, 120.2, 64.1, 62.9, 51.2, 49.2. **HRMS (ESI/TOF-Q) m/z**: [M+H]+ Calculated for $C_{29}H_{19}ClOSH^+$ 451.0918; Found 451.0927.

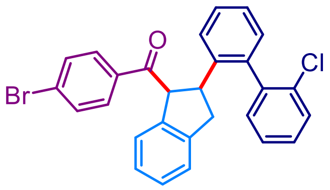

7j, 43%, dr = 1:1

Compound, 7j: White colored solid; eluent (2% ethyl acetate in hexane). Yield: 43% (42 mg); **^{1}H NMR (400 MHz, CDCl$_3$) δ:** 7.74 (d, J = 8.1 Hz, 2H), 7.62 (d, J = 8.1 Hz, 2H), 7.57 – 7.49 (m, 5H), 7.41 – 7.37 (m, 2H), 7.31 – 7.27 (m, 6H), 7.25 – 7.15 (m, 7H), 7.12 – 7.09 (m, 5H), 6.92 (dd, J = 16.9, 7.6 Hz, 2H), 6.74 (d, J = 7.6 Hz, 1H), 5.10 (dd, J = 18.1, 6.0 Hz, 2H), 4.03 – 3.98 (m, 1H), 3.90 (q, J = 7.9 Hz, 1H), 3.60 – 3.48 (m, 2H), 3.23 (dd, J = 16.3, 7.6 Hz, 1H), 3.09 (dd, J = 16.4, 4.8 Hz, 1H). **^{13}C NMR (101 MHz, CDCl$_3$) δ**: 198.81, 198.77, 143.8, 143.7, 143.6, 142.7, 141.03, 140.99, 139.9, 139.8, 139.5, 138.9, 135.9, 135.7, 133.7, 133.6, 132.0, 131.6, 131.4, 130.7, 130.6, 130.2, 130.0, 129.7, 129.3, 129.0, 128.9, 128.8, 128.5, 128.3, 128.03, 127.98, 126.9, 126.71, 126.66, 126.5, 126.4, 126.0, 125.8, 125.1, 124.90, 124.87, 124.7, 61.7, 61.2, 45.8, 43.9, 41.6, 41.5. **HRMS (ESI/TOF-Q) m/z**: [M+H]+ Calculated for $C_{28}H_{20}BrClOH^+$ 487.0459; Found 487.0421.

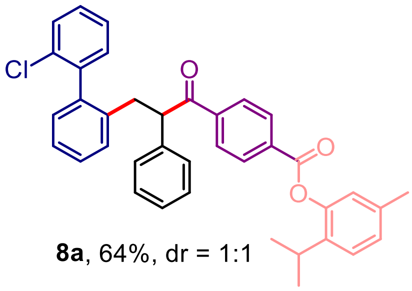

8a, 64%, dr = 1:1

Compound, 8a: yellow sticky liquid; eluent (5% ethyl acetate in hexane). Yield: 64% (73 mg); **^{1}H NMR (500 MHz, $CDCl_3$) δ**: 8.17 – 8.14 (m, 3H), 7.90 – 7.86 (m, 4H), 7.62 (d, *J* = 7.7 Hz, 1H), 7.51 (d, *J* = 7.8 Hz, 1H), 7.43 – 7.38 (m, 3H), 7.35 – 7.24 (m, 9H), 7.22 – 7.07 (m, 12H), 7.03 (d, *J* = 7.2 Hz, 1H), 6.96 – 6.91 (m, 6H), 4.69 – 4.59 (m, 2H), 3.55 – 3.48 (m, 2H), 3.01 – 2.94 (m, 4H), 2.34 (s, 6H), 1.20 (d, *J* = 6.6 Hz, 12H). **^{13}C NMR (126 MHz, $CDCl_3$) δ**: 198.8, 198.5, 164.6, 148.1, 140.6, 140.4, 140.2, 139.5, 139.3, 138.9, 138.3, 137.6, 137.4, 137.2, 136.9, 133.7, 133.5, 133.1, 133.1, 131.7, 131.6, 130.8, 130.7, 130.31, 130.25, 130.1, 130.0, 129.8, 129.1, 129.03, 128.97, 128.8, 128.7, 128.4, 128.1, 127.99, 127.95, 127.5, 127.3, 127.0, 126.9, 126.7, 126.4, 122.8, 55.1, 54.5, 37.9, 37.6, 27.4, 23.1, 21.0. **HRMS (ESI/TOF-Q) m/z**: [M+H]+ Calculated for $C_{38}H_{33}ClO_3H^+$ 573.2191; Found 573.2191.

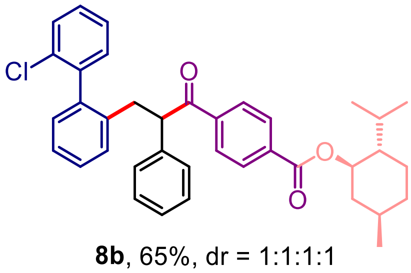

8b, 65%, dr = 1:1:1:1

Compound, 8b: yellow sticky liquid; eluent (15% ethyl acetate in hexane). Yield: 65% (75 mg); **^{1}H NMR (500 MHz, DMSO-d_6) δ**: 7.93 – 7.89 (m, 15H), 7.61 – 7.45 (m, 9H), 7.41 – 7.26 (m, 9H), 7.23 – 7.17 (m, 11H), 7.13 – 7.08 (m, 10H), 7.02 (d, *J* = 6.5 Hz, 6H), 6.86 (d, *J* = 5.3 Hz, 6H), 4.91 – 4.79 (m, 8H), 3.33 – 3.31 (m, 2H), 3.22 (dd, *J* = 14.0, 8.0 Hz, 2H), 2.89 (dd, *J* = 14.1, 5.7 Hz, 2H), 2.76 – 2.72 (m, 2H), 1.94 (d, *J* = 11.6 Hz, 4H), 1.81 – 1.79 (m, 4H), 1.63 (d, *J* = 11.2 Hz, 8H), 1.49 (t, *J* = 10.4 Hz, 8H), 1.10 – 1.01 (m, 8H), 0.90 – 0.79 (m, 30H), 0.70 – 0.68 (m, 12H).**^{13}C NMR (126 MHz, DMSO-d_6) δ**: 198.5, 198.4, 198.2, 198.1, 164.32, 164.29, 139.5, 139.4, 139.24, 139.15, 139.1, 139.0, 138.9, 138.2, 138.00, 137.99, 137.1, 137.04, 136.95, 136.9, 133.62, 133.60, 133.54, 133.51, 132.4, 132.3, 131.7, 131.5, 129.71, 129.68, 129.5, 129.42, 129.37, 129.21, 129.16, 128.89, 128.87, 128.78, 128.76, 128.63, 128.58, 127.97, 127.95, 127.92, 127.87, 127.8, 127.7, 127.3, 127.18, 127.16, 127.1, 127.0, 126.2, 74.7, 53.74, 53.65, 52.70, 52.65, 46.5, 40.3, 36.6, 36.5, 36.1, 35.9, 33.6, 30.8, 26.14, 26.06, 23.14, 23.07, 21.8, 20.41, 20.37, 16.4, 16.3. **HRMS (ESI/TOF-Q) m/z**: [M+H]+ Calculated for $C_{38}H_{39}ClO_3H^+$ 579.2660; Found 579.2663.

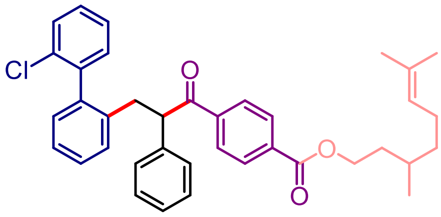

8c, 67%, dr = 1:1

Compound, 8c: yellow sticky liquid; eluent (5% ethyl acetate in hexane). Yield: 67% (78 mg); **^{1}H NMR (500 MHz, DMSO-d_6) δ**: 7.93 – 7.87 (m, 8H), 7.60-7.58 (m, 1H), 7.55-7.53(m, 1H), 7.50-7.47(m, 1H), 7.46-7.42(m, 2H), 7.40-7.35 (m, 2H), 7.31 (td, J = 7.5, 1.2 Hz, 1H), 7.27 (td, J = 7.6, 1.5 Hz, 1H), 7.23 – 7.17 (m, 6H), 7.14 – 7.08 (m, 5H), 7.02-7.00 (m, 3H), 6.87 – 6.83 (m, 3H), 5.02 (t, *J* = 7.0 Hz, 2H), 4.89 – 4.84 (m, 2H), 4.32 – 4.22 (m, 4H), 3.35 (dd, *J* = 14.0, 6.2 Hz, 1H), 3.23 (dd, *J* = 14.3, 8.1 Hz, 1H), 2.89 (dd, *J* = 14.3, 6.3 Hz, 1H), 2.74 (dd, *J* = 14.0, 8.0 Hz, 1H), 1.98 – 1.86 (m, 4H), 1.73 – 1.66 (m, 2H), 1.61 – 1.45 (m, 16H), 1.34 – 1.27 (m, 2H), 1.18 – 1.10 (m, 2H), 0.87 (d, *J* = 6.5 Hz, 6H). **^{13}C NMR (126 MHz, DMSO- d_6) δ**: 198.5, 198.2, 164.9, 164.8, 139.5, 139.4, 139.3, 139.2, 139.0, 138.9, 138.3, 138.0, 137.1, 136.9, 133.5, 133.4, 132.4, 132.3, 131.7, 131.5, 130.6, 129.73, 129.70, 129.5, 129.43, 129.35, 129.22, 129.17, 128.9, 128.8, 128.6, 128.5, 128.0, 127.9, 127.83, 127.75, 127.3, 127.2, 127.08, 127.05, 126.2, 124.4, 63.4, 53.7, 52.7, 36.6, 36.33, 36.31, 36.0, 34.8, 28.8, 25.4, 24.8, 19.2, 17.4. **HRMS (ESI/TOF-Q) m/z**: [M+Na]+ Calculated for $C_{38}H_{39}ClO_3Na^+$ 601.2480; Found 601.2483.

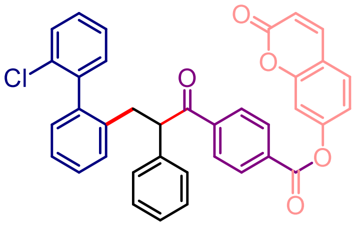

8d, 60%, dr = 1:1

Compound, 8d: Pale yellow colored solid; eluent (20% ethyl acetate in hexane). Yield: 60% (70 mg); **^{1}H NMR (400 MHz, $CDCl_3$) δ**: 8.12 (dd, *J* = 8.5, 5.6 Hz, 4H), 7.84 (dd, *J* = 8.4, 6.1 Hz, 4H), 7.71 (d, *J* = 9.6 Hz, 2H), 7.64 – 7.59 (m, 1H), 7.53 (dd, *J* = 8.5, 1.5 Hz, 2H), 7.48 (dd, *J* = 7.6, 1.7 Hz, 1H), 7.43 – 7.36 (m, 3H), 7.34 – 7.31 (m, 2H), 7.28 (dd, *J* = 7.4, 1.9 Hz, 2H), 7.24 (d, *J* = 1.8 Hz, 1H), 7.23 – 7.22 (m, 2H), 7.21 – 7.19 (m, 3H), 7.18 – 7.15 (m, 6H), 7.13 – 7.11 (m, 2H), 7.07 (dd, *J* = 7.4, 1.6 Hz, 1H), 7.00 (dd, *J* = 7.2, 2.1 Hz, 1H), 6.92 – 6.89 (m, 3H), 6.88 – 6.79 (m, 2H), 6.43 – 6.22 (m, 2H), 4.62 (dd, *J* = 9.1, 4.9 Hz, 1H), 4.55 (dd, *J* = 8.2, 5.9 Hz, 1H), 3.52 – 3.44 (m, 2H), 2.99 – 2.90 (m, 2H). **^{13}C NMR (101 MHz, $CDCl_3$) δ**: 198.8, 198.5, 163.8, 160.4, 154.9, 153.3, 143.0, 141.0, 140.8, 140.4, 140.2, 139.5, 139.2, 138.8, 138.2, 137.5, 137.4, 133.7, 133.5, 132.13, 132.09, 131.7, 131.6, 130.9, 130.7, 130.5, 130.4, 130.3, 130.1, 130.0, 129.8, 129.2, 129.1, 129.0, 128.9, 128.83, 128.77, 128.4, 128.1, 128.0, 127.4, 127.1, 126.9, 126.5, 118.5, 117.1, 116.4, 110.6, 55.1, 54.6, 37.9, 37.6. **HRMS (ESI/TOF-Q) m/z**: [M+Na]+ Calculated for $C_{37}H_{25}ClO_5Na^+$ 607.1283; Found 607.1276.

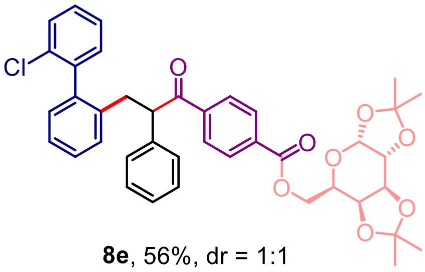

8e, 56%, dr = 1:1

Compound, 8e: brown sticky liquid; eluent (20% ethyl acetate in hexane). Yield: 60% (70 mg); **^{1}H NMR (500 MHz, $CDCl_3$) δ**: 7.97 -7.95 (m, 4H), 7.77 – 7.74 (m, 4H), 7.58 (d, *J* = 7.7 Hz, 1H), 7.46 (d, *J* = 7.9 Hz, 1H), 7.39 – 7.34 (m, 3H), 7.30 – 7.19 (m, 7H), 7.17 – 7.06 (m, 9H), 6.98 (d, *J* = 6.8 Hz, 1H), 6.89-6.88 (m, 4H), 5.55 (d, *J* = 4.9 Hz, 2H), 4.64 - 4.58 (m, 3H), 4.54 – 4.49 (m, 3H), 4.42 – 4.38 (m, 2H), 4.34 (bs, 2H), 4.28 (d, *J* = 8.0 Hz, 2H), 4.15 – 4.13 (m, 2H), 3.49 – 3.42 (m, 2H), 2.97 – 2.88 (m, 2H), 1.48 (s, 6H), 1.46 (s, 6H), 1.34 (s, 6H), 1.32 (s, 6H). **^{13}C NMR (126 MHz, $CDCl_3$) δ**: 198.9, 198.6, 165.6, 140.4, 140.2, 140.0, 139.4, 139.2, 138.9, 138.3, 137.5, 137.4, 133.6, 133.48, 133.45, 133.4, 131.6, 131.5, 130.8, 130.7, 130.2, 130.0, 129.92, 129.85, 129.8, 129.7, 129.1, 129.0, 128.9, 128.6, 128.5, 128.4, 128.01, 127.97, 127.9, 127.2, 127.0, 126.8, 126.4, 109.8, 108.9, 96.4, 71.2, 70.8, 70.6, 66.2, 64.34, 64.31, 54.9, 54.4, 37.8, 37.6, 26.11, 26.06, 25.1, 24.6. **HRMS (ESI/TOF-Q) m/z**: [M+Na]+ Calculated for $C_{40}H_{39}ClO_8Na^+$ 705.2226; Found 705.2201.

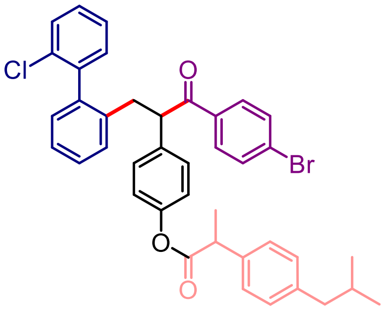

8f, 53%, dr = 1:1

Compound, 8f: yellow sticky liquid; eluent (2% ethyl acetate in hexane). Yield: 53% (72 mg); **^{1}H NMR (500 MHz, DMSO-*d_6*) δ**: 7.74 – 7.69 (m, 4H), 7.62 – 7.60 (m, 3H), 7.59 – 7.57 (m, 1H), 7.52 (d, *J* = 8.0 Hz, 1H), 7.49 – 7.43 (m, 3H), 7.41 – 7.33 (m, 3H), 7.30 – 7.18 (m, 10H), 7.14 – 7.12 (m, 4H), 7.08 – 7.04 (m, 3H), 6.98 – 6.97 (m, 1H), 6.87 – 6.82 (m, 4H), 6.78 (d, *J* = 8.7 Hz, 2H), 6.71 (d, *J* = 7.5 Hz, 1H), 4.88 – 4.82 (m, 2H), 4.00 – 3.94 (m, 2H), 3.28 (dd, *J* = 14.0, 5.4 Hz, 1H), 3.16 (dd, *J* = 14.2, 7.7 Hz, 1H), 2.86 (dd, *J* = 14.3, 6.6 Hz, 1H), 2.69 (dd, *J* = 13.8, 8.9 Hz, 1H), 2.41 (d, *J* = 7.1 Hz, 4H), 1.83 – 1.75 (m, 2H), 1.44 (t, *J* = 7.0 Hz, 6H), 0.83 (d, *J* = 6.6 Hz, 12H). **^{13}C NMR (126 MHz, DMSO-*d_6*) δ**: 198.0, 197.7, 172.7, 172.6, 149.5, 149.4, 140.0, 139.5, 139.3, 139.1, 138.9, 137.4, 136.9, 136.8, 135.86, 135.85, 135.6, 134.7, 134.6, 132.3, 132.2, 131.9, 131.8, 131.7, 131.59, 131.57, 130.4, 130.3, 129.7, 129.5, 129.43, 129.36, 129.3, 129.2, 129.04, 129.02, 127.9, 127.8, 127.7, 127.5, 127.3, 127.13, 127.07, 126.3, 126.2, 121.84, 121.77, 52.8, 51.5, 44.2, 44.1, 36.6, 35.9, 29.6, 22.2, 18.4. **HRMS (ESI/TOF-Q) m/z**: [M+Na]+ Calculated for $C_{40}H_{36}BrClO_3Na^+$ 701.1429; Found 701.1412.

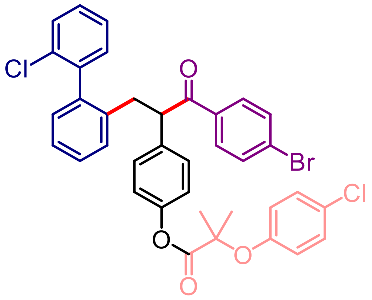

8g, 51%, dr = 1:1

Compound, 8g: yellow sticky liquid; eluent (5% ethyl acetate in hexane). Yield: 51% (70 mg); **^{1}H NMR (400 MHz, $CDCl_3$) δ**: 7.59 – 7.55 (m, 5H), 7.48 – 7.42 (m, 5H), 7.41 – 7.27 (m, 5H), 7.24 – 7.20 (m, 9H), 7.15 - 7.13 (m, 1H), 7.11 – 7.09 (m, 1H), 7.08 – 7.06 (m, 1H), 6.95 (dd, *J* = 7.4, 1.8 Hz, 1H), 6.90 – 6.79 (m, 12H), 4.56 (dd, *J* = 9.0, 5.0 Hz, 1H), 4.48 (dd, *J* = 8.5, 5.7 Hz, 1H), 3.48 – 3.38 (m, 2H), 2.93 – 2.85 (m, 2H), 1.70 (s, 6H), 1.69 (s, 6H). **^{13}C NMR (101 MHz, $CDCl_3$) δ**: 198.2, 197.9, 172.64, 172.59, 154.1, 149.7, 149.6, 140.3, 140.1, 139.5, 139.2, 137.3, 137.2, 137.1, 136.4, 135.3, 135.1, 133.6, 133.4, 132.0, 131.9, 131.7, 131.6, 130.7, 130.6, 130.3, 130.2, 130.1, 130.0, 129.7, 129.44, 129.40, 129.1, 129.0, 128.3, 128.1, 128.0, 127.7, 127.1, 126.9, 126.5, 121.72, 121.66, 120.6, 120.5, 79.6, 53.9, 53.2, 37.9, 37.6, 25.5, 25.4. **HRMS (ESI/TOF-Q) m/z**: [M+Na]+ Calculated for $C_{37}H_{29}BrCl_2O_4Na^+$ 709. 0518; Found 709.0484.

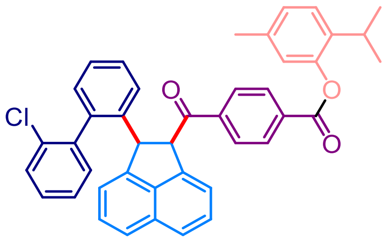

8h, 45%, dr = 1:1

Compound, 8h: brown coloured solid; eluent (10% ethyl acetate in hexane). Yield: 45% (56 mg); **^{1}H NMR (500 MHz, $CDCl_3$) δ**: 8.17 (d, *J* = 7.9 Hz, 2H), 8.12 (d, *J* = 7.9 Hz, 2H), 7.94 (d, *J* = 8.0 Hz, 2H), 7.80 (d, *J* = 8.0 Hz, 2H), 7.57 – 7.53 (m, 3H), 7.42 – 7.36 (m, 3H), 7.29 – 7.08 (m, 15H), 7.05 (d, *J* = 7.5 Hz, 2H), 6.99 (t, *J* = 7.9 Hz, 1H), 6.95 (d, *J* = 8.2 Hz, 2H), 6.83 – 6.78 (m, 5H), 6.74 – 6.70 (m, 2H), 6.66 (d, *J* = 7.7 Hz, 1H), 5.37 (s, 1H), 5.34 (s, 1H), 5.29 (s, 1H), 5.19 (s, 1H), 2.96 – 2.88 (m, 2H), 2.21 (s, 6H), 1.09 (t, *J* = 6.6 Hz, 12H). **^{13}C NMR (126 MHz, $CDCl_3$) δ**: 196.3, 195.6, 164.8, 164.7, 148.14, 148.09, 147.6, 147.1, 143.4, 143.3, 141.31, 141.30, 140.5, 140.4, 139.9, 139.7, 139.5, 139.3, 138.1, 137.9, 137.3, 137.2, 137.0, 136.9, 133.7, 133.51, 133.47, 133.4, 131.9, 131.8, 131.7, 131.4, 130.5, 130.4, 130.04, 129.99, 129.63, 129.61, 129.5, 129.3, 129.1, 128.9, 128.83, 128.79, 128.5, 128.2, 127.92, 127.90, 127.62, 127.58, 127.2, 127.0, 126.77, 126.75, 126.6, 126.5, 124.50, 124.46, 123.52, 123.47, 122.84, 122.81, 121.6, 121.4, 120.6, 120.3, 62.9, 62.1, 50.5, 48.9, 27.5, 27.4, 23.23, 23.20, 21.0. **HRMS (ESI/TOF-Q) m/z**: [M+H]+ Calculated for $C_{42}H_{33}ClO_3H^+$ 621.2191; Found 621.2195.

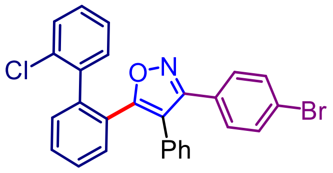

9, 45%, dr = 1:1

Compound, 9: white colored liquid; eluent (5% ethyl acetate in hexane). Yield: 45% (44 mg); **^{1}H NMR (400 MHz, $CDCl_3$) δ:** 7.56 – 7.50 (m, 2H), 7.47 – 7.41 (m, 3H), 7.37 (d, *J* = 7.6 Hz, 1H), 7.29 – 7.23 (m, 4H), 7.19 – 7.11 (m, 3H), 7.06 (t, *J* = 7.5 Hz, 1H), 6.94 – 6.92 (m, 1H), 6.81 – 6.79 (m, 2H). **^{13}C NMR (101 MHz, $CDCl_3$) δ**: 167.1, 160.5, 139.2, 138.7, 132.9, 131.8, 131.6, 131.5, 130.5, 130.2, 130.0, 129.8, 129.52, 129.48, 128.8, 128.7, 128.10, 128.07, 127.8, 127.4, 126.5, 123.9, 116.8. **HRMS (ESI/TOF-Q) m/z**: [M+H]+ Calculated for $C_{27}H_{17}BrClNOH^+$ 486.0255; Found 486.0231.

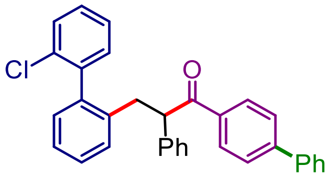

10, 74%, dr = 1:1

Compound, 10: yellow colored sticky liquid; eluent (2% ethyl acetate in hexane). Yield: 74% (70 mg); **^{1}H NMR (400 MHz, $CDCl_3$) δ:** 7.88 – 7.83 (m, 4H), 7.62 – 7.59 (m, 1H), 7.56 – 7.52 (m, 8H), 7.50 – 7.48 (m, 1H), 7.46 – 7.40 (m, 4H), 7.39 – 7.36 (m, 4H), 7.36 – 7.26 (m, 4H), 7.25 – 7.22 (m, 4H), 7.21 – 7.11 (m, 9H), 7.04 – 7.01 (m, 1H), 6.99 – 6.96 (m, 4H), 4.70 (dd, *J* = 9.0, 5.1 Hz, 1H), 4.61 (dd, *J* = 8.1, 6.2 Hz, 1H), 3.55 – 3.46 (m, 2H), 3.00 – 2.92 (m, 2H). **^{13}C NMR (101 MHz, $CDCl_3$) δ**: 198.9, 198.6, 145.5, 140.5, 140.3, 140.02, 139.97, 139.6, 139.5, 139.3, 139.0, 137.9, 137.8, 135.5, 135.4, 133.7, 133.5, 131.7, 131.6, 130.8, 130.7, 130.2, 130.0, 129.9, 129.8, 129.4, 129.3, 129.02, 129.00, 128.98, 128.9, 128.4, 128.3, 128.2, 128.0, 127.9, 127.3, 127.22, 127.16, 127.1, 127.0, 126.8, 126.30, 126.27, 54.6, 54.0, 37.9, 37.7. **HRMS (ESI/TOF-Q) m/z**: [M+H]+ Calculated for $C_{33}H_{25}ClOH^+$ 473.1667; Found 473.1665.

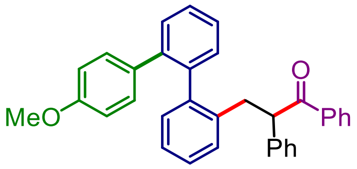

11a, 68%, dr = 1:1

Compound, 11a: yellow sticky liquid; eluent (2% ethyl acetate in hexane). Yield: 68% (64 mg); **^{1}H NMR (400 MHz, CDCl$_3$) δ:** 7.71 – 7.69 (m, 2H), 7.60 – 7.57 (m, 2H), 7.51 – 7.47 (m, 2H), 7.46 – 7.45 (m, 3H), 7.43 – 7.35 (m, 4H), 7.31 – 7.28 (m, 4H), 7.24 – 7.19 (m, 2H), 7.18 – 7.10 (m, 9H) 7.06 – 7.00 (m, 7H), 6.95 – 6.93 (m, 2H), 6.78 (d, *J* = 7.6 Hz, 1H), 6.74 – 6.72 (m, 2H), 6.67 – 6.61 (m, 4H), 4.49 (dd, *J* = 8.3, 6.3 Hz, 1H), 4.39 (dd, *J* = 8.2, 6.0 Hz, 1H), 3.73 (s, 3H), 3.72 (s, 3H), 3.16 (dd, *J* = 14.3, 6.2 Hz, 1H), 3.04 (dd, *J* = 14.0, 8.3 Hz, 1H), 2.77 (dd, *J* = 14.4, 8.6 Hz, 1H), 2.67 – 2.59 (m, 1H). **^{13}C NMR (101 MHz, CDCl$_3$) δ**: 199.9, 199.1, 158.6, 158.5, 141.8, 141.5, 140.6, 140.5, 140.3, 140.1, 139.6, 139.0, 137.5, 137.00, 136.96, 133.9, 133.7, 132.74, 132.70, 131.4, 131.3, 131.1, 130.72, 130.67, 130.5, 130.2, 128.82, 128.75, 128.7, 128.63, 128.57, 128.5, 128.4, 128.1, 128.0, 127.9, 127.2, 127.03, 126.98, 126.95, 126.9, 126.1, 113.50, 113.45, 55.24, 55.21, 54.4, 53.1, 37.8, 37.7. **HRMS (ESI/TOF-Q) m/z**: [M+H]+ Calculated for $C_{34}H_{28}O_2H^+$ 469.2162; Found 469.2172.

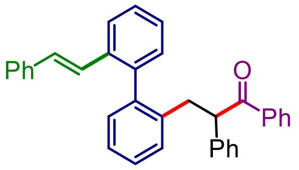

11b, 61%, dr = 1:1

Compound, 11b: colorless sticky liquid; eluent (2% ethyl acetate in hexane). Yield: 61% (57 mg); **^{1}H NMR (400 MHz, CDCl$_3$) δ:** 7.91 (d, *J* = 7.9 Hz, 1H), 7.84 (d, *J* = 7.9 Hz, 1H), 7.63 – 7.60 (m, 3H), 7.49 – 7.33 (m, 5H), 7.32 – 7.30 (m, 6H), 7.27 – 7.19 (m, 10H), 7.16 – 7.14 (m, 5H), 7.12 – 7.01 (m, 13H), 6.93 (d, *J* = 16.4 Hz, 1H), 6.89 – 6.84 (m, 4H), 6.69 (d, *J* = 16.4 Hz, 1H), 4.58 – 4.50 (m, 2H), 3.47 – 3.37 (m, 2H), 3.02 (dd, *J* = 13.7, 4.3 Hz, 1H), 2.93 (dd, *J* = 13.7, 7.8 Hz, 1H). **^{13}C NMR (101 MHz, CDCl$_3$) δ**: 199.4, 199.2, 140.92, 140.85, 140.8, 140.6, 139.5, 138.9, 138.1, 137.9, 137.5, 137.4, 136.84, 136.81, 135.9, 135.8, 132.74, 132.67, 131.2, 131.1, 130.8, 130.7, 130.6, 130.4, 129.8, 128.9, 128.79, 128.75, 128.7, 128.62, 128.58, 128.55, 128.5, 128.4, 128.04, 127.95, 127.9, 127.8, 127.7, 127.64, 127.59, 127.5, 127.1, 127.0, 126.9, 126.74, 126.71, 126.4, 126.3, 125.4, 125.2, 54.1, 53.8, 38.1, 37.9. **HRMS (ESI/TOF-Q) m/z**: [M+H]+ Calculated for $C_{35}H_{28}OH^+$ 465.2213; Found 465.2210.

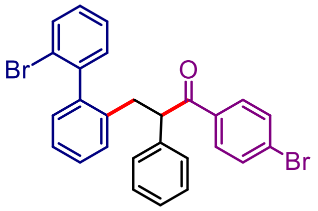

4a', 40%, dr = 1:1

Compound, 4a': pale yellow sticky solid; eluent (1% ethyl acetate in hexane). Yield: 40% (41 mg); **^{1}H NMR (400 MHz, CDCl$_3$) δ:** 7.79 – 7.77 (m, 1H), 7.66 – 7.58 (m, 5H), 7.46 – 7.41 (m, 4H), 7.40 – 7.38 (m, 1H), 7.36 – 7.28 (m, 3H), 7.26 – 7.11 (m, 13H), 7.10 – 7.08 (m, 1H), 7.03 (dd, *J* = 7.3, 1.7 Hz, 1H), 6.97 (dd, *J* = 7.5, 1.8 Hz, 1H), 6.93 – 6.91 (m, 2H), 6.87 – 6.85 (m, 2H), 4.57 (dd, *J* = 9.2, 4.7 Hz, 1H), 4.50 (dd, *J* = 8.4, 5.7 Hz, 1H), 3.49 – 3.39 (m, 2H), 2.93 – 2.85 (m, 2H). **^{13}C NMR (101 MHz, CDCl$_3$) δ**: 198.4, 198.0, 142.4, 142.2, 141.2, 141.0, 139.3, 138.6, 137.4, 137.2, 135.5, 135.3, 133.1, 132.9, 131.9, 131.8, 131.6, 131.5, 130.8, 130.7, 130.4, 130.3, 130.2, 130.0, 129.2, 129.1, 129.0, 128.4, 128.1, 127.9, 127.6, 127.4, 127.2, 126.3, 124.2, 124.0, 54.5, 54.0, 37.8, 37.6. **HRMS (ESI/TOF-Q) m/z**: [M+H]+ Calculated for $C_{27}H_{20}Br_2OH^+$ 518.9954; Found 518.9949.

NMR spectra of synthesized compounds

1-(4-bromophenyl)-3-(2'-chloro-[1,1'-biphenyl]-2-yl)-2-phenylpropan-1-one(4a)

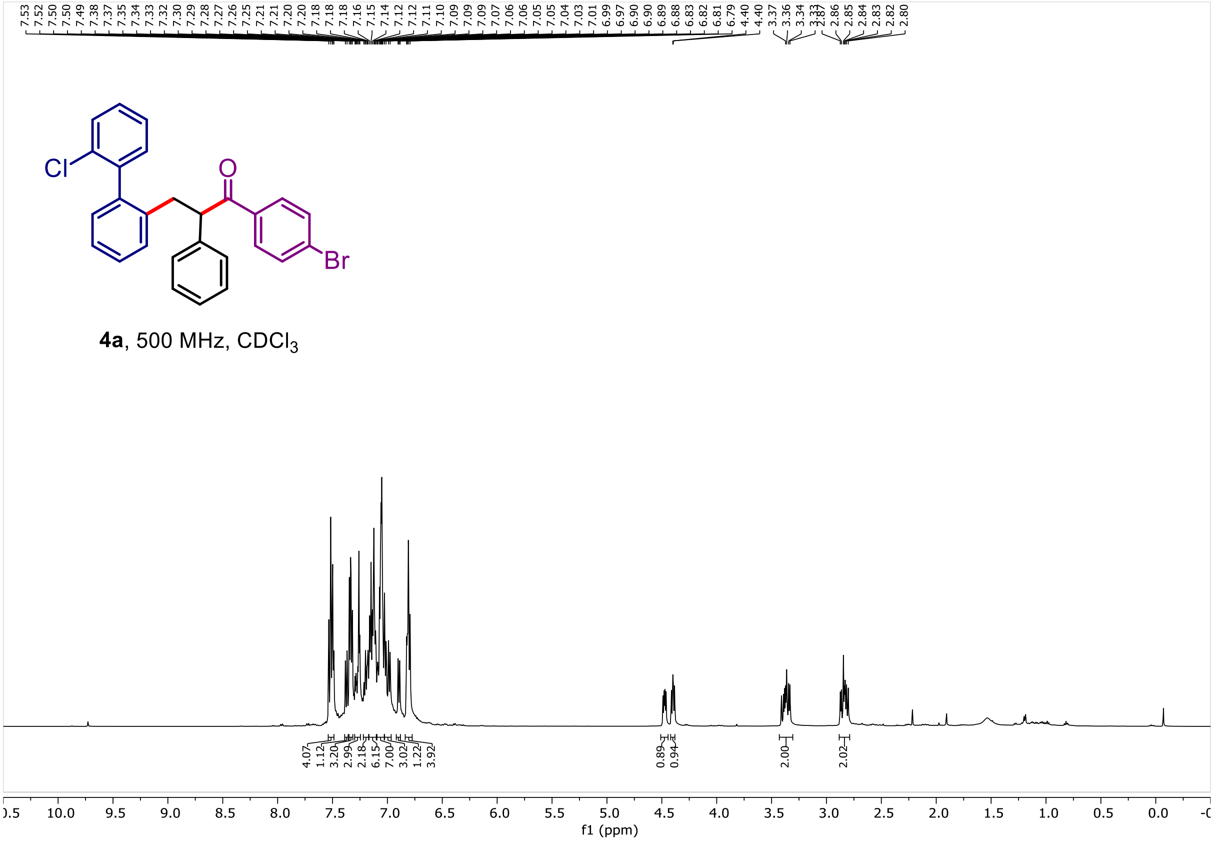

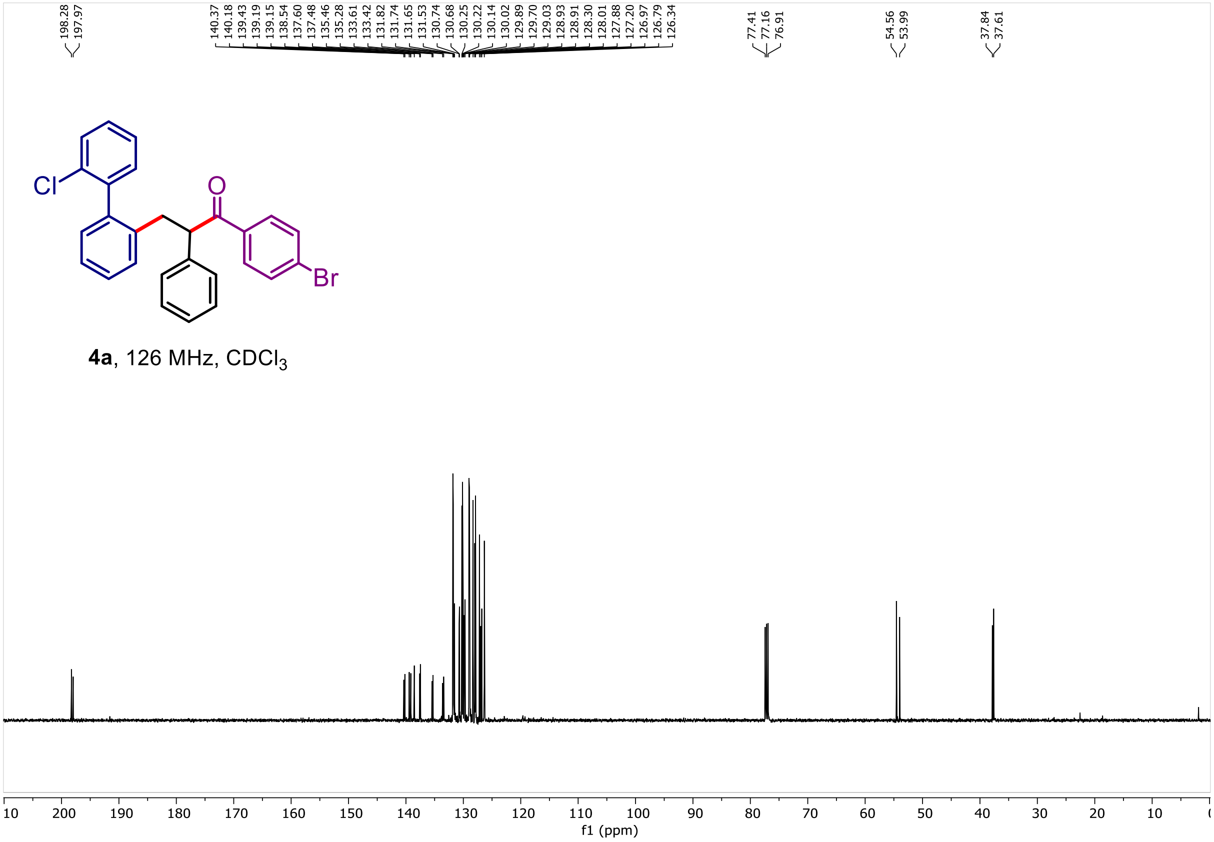

3-(2'-chloro-[1,1'-biphenyl]-2-yl)-1,2-diphenylpropan-1-one(4b)

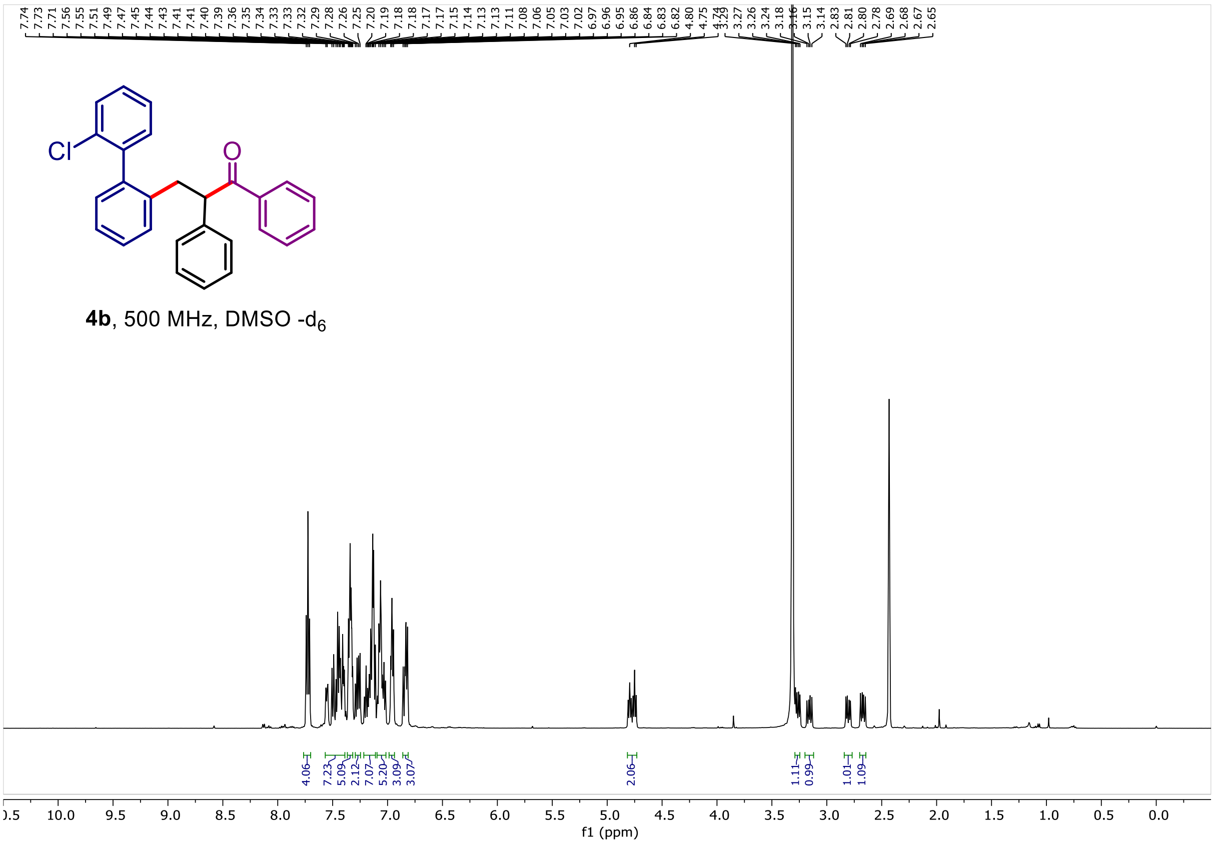

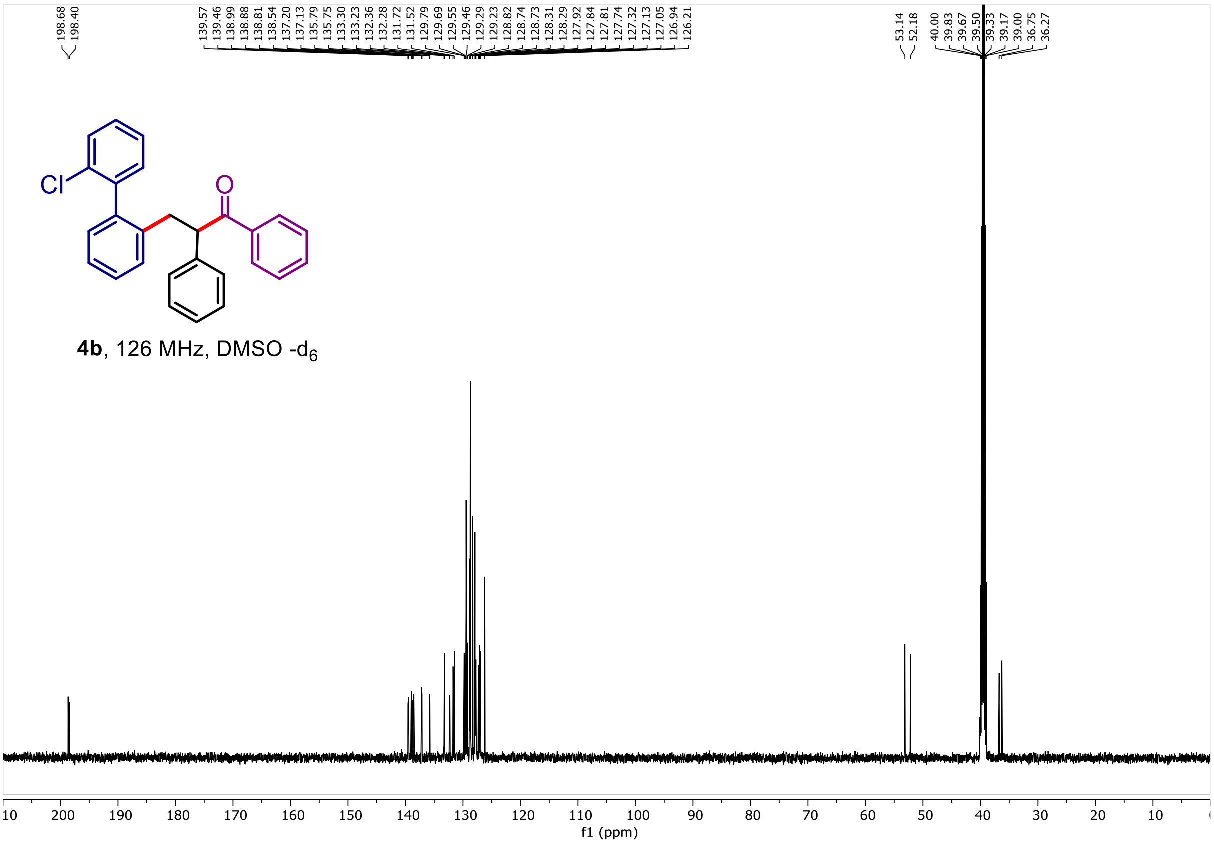

3-(2'-chloro-[1,1'-biphenyl]-2-yl)-1-(4-nitrophenyl)-2-phenylpropan-1-one(4c)

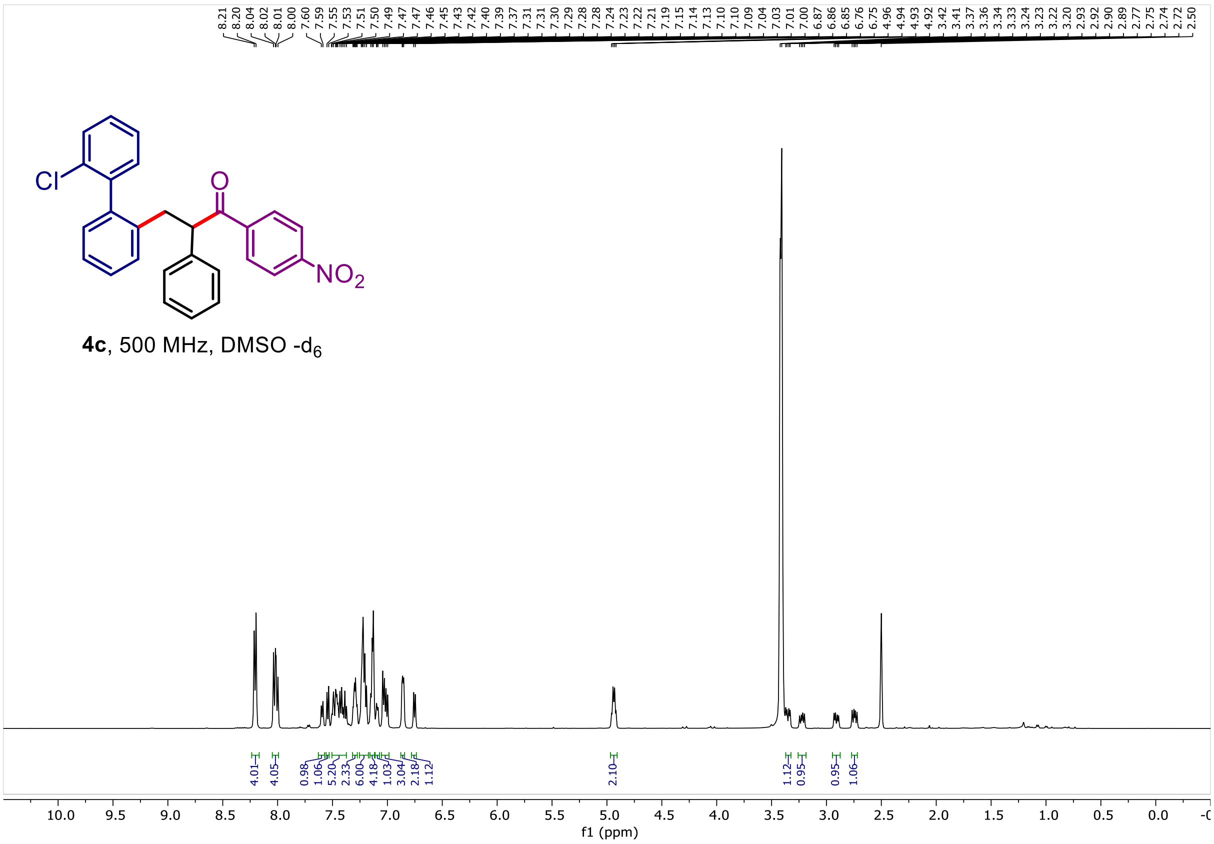

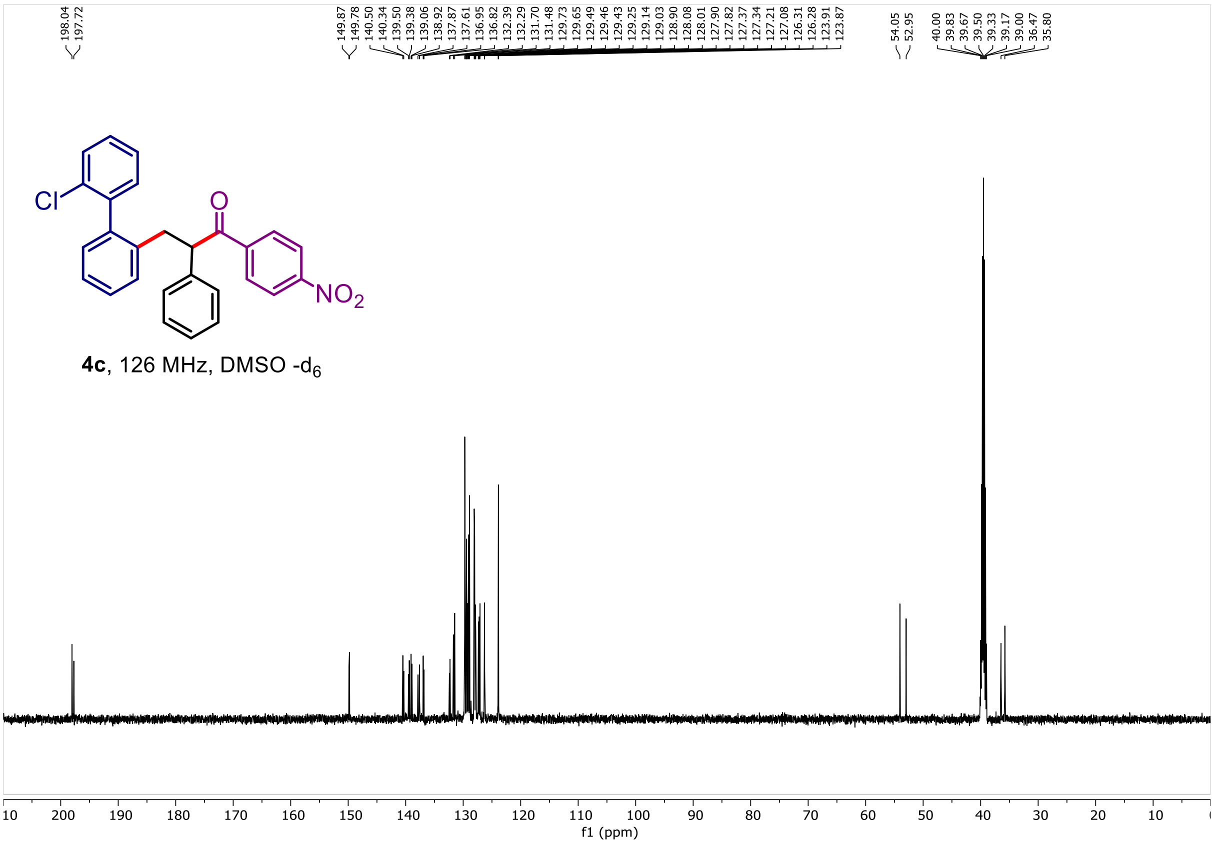

4-(3-(2'-chloro-[1,1'-biphenyl]-2-yl)-2-phenylpropanoyl)benzonitrile (4d)

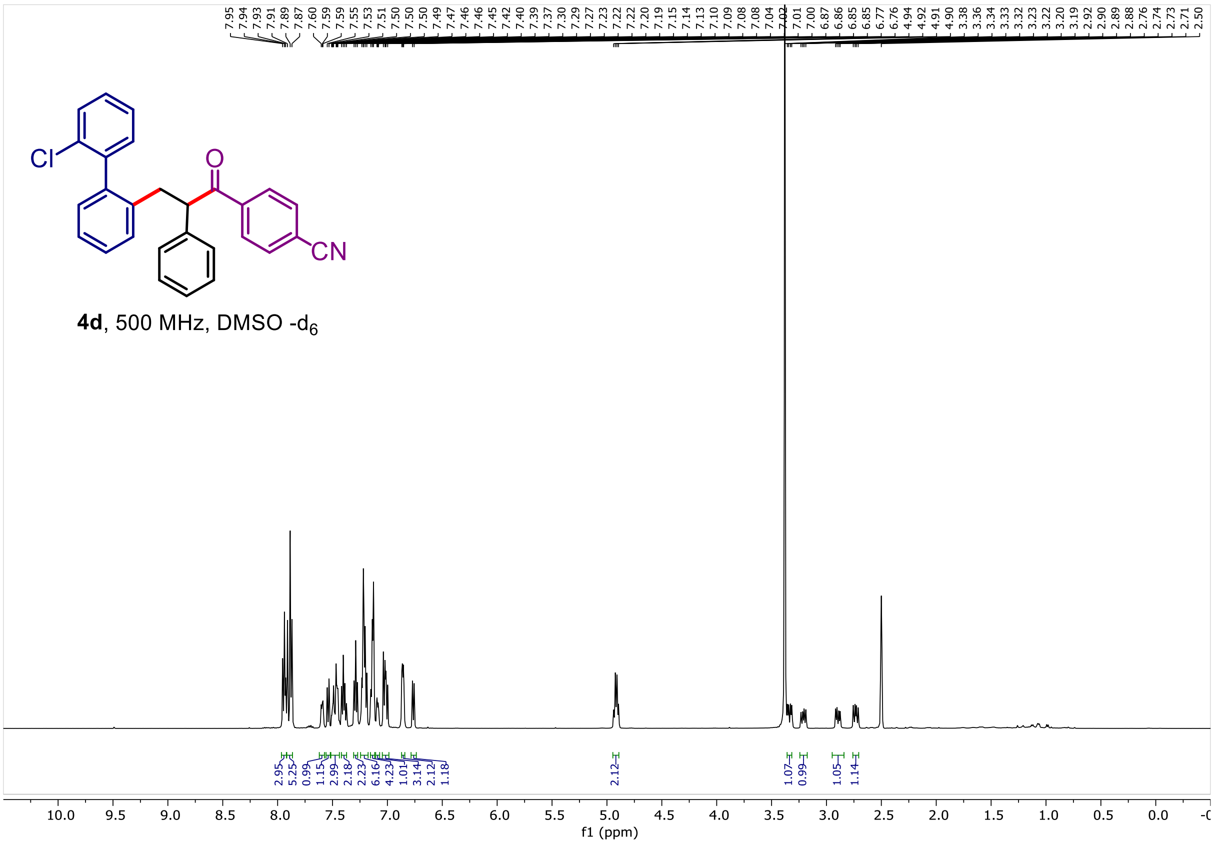

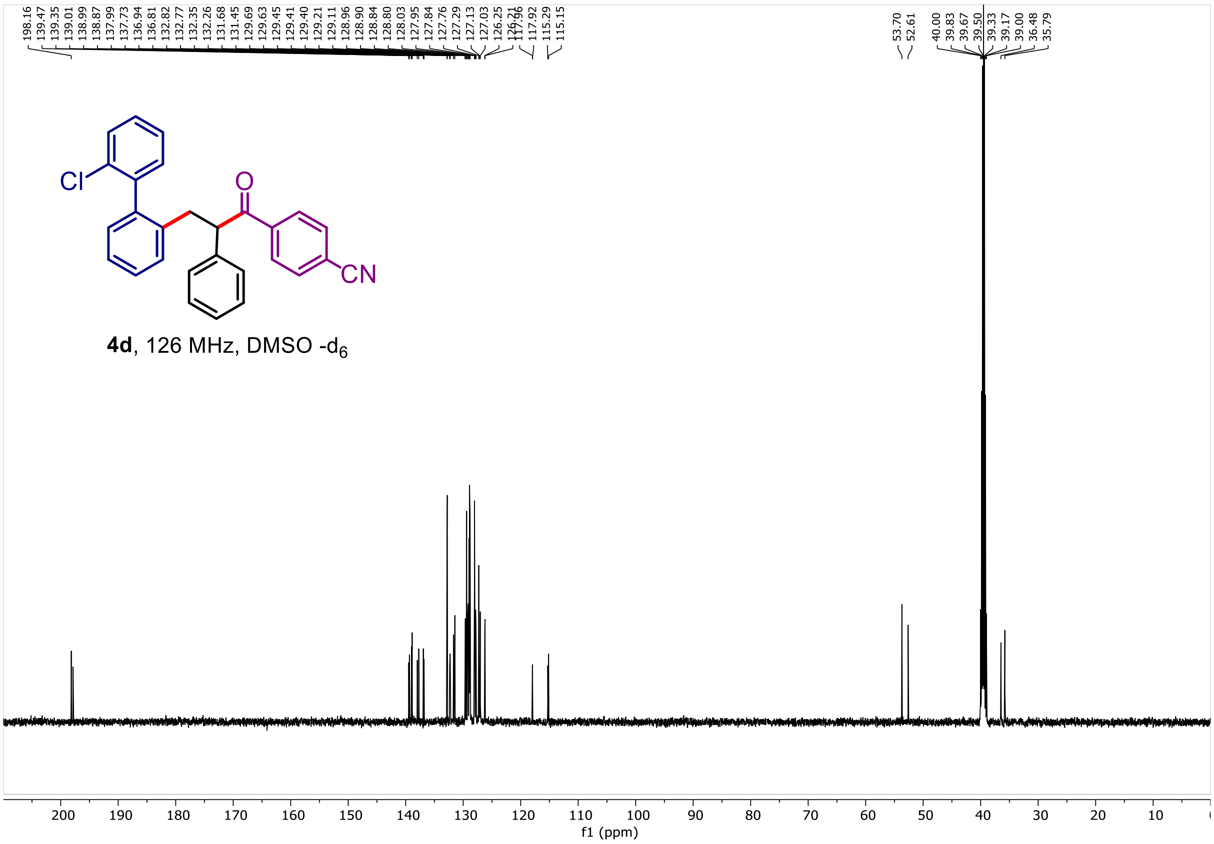

3-(2'-chloro-[1,1'-biphenyl]-2-yl)-2-phenyl-1-(4-(trifluoromethyl)phenyl)propan-1-one (4e)

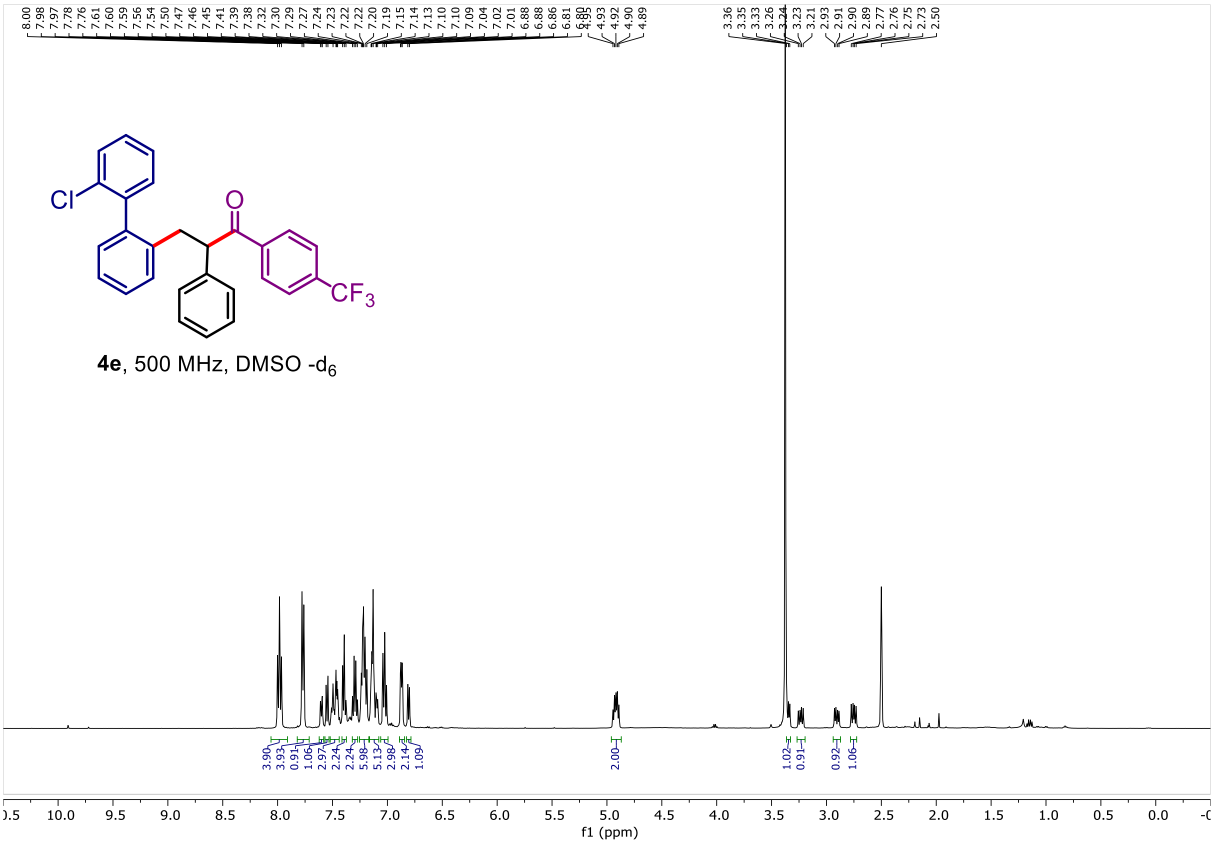

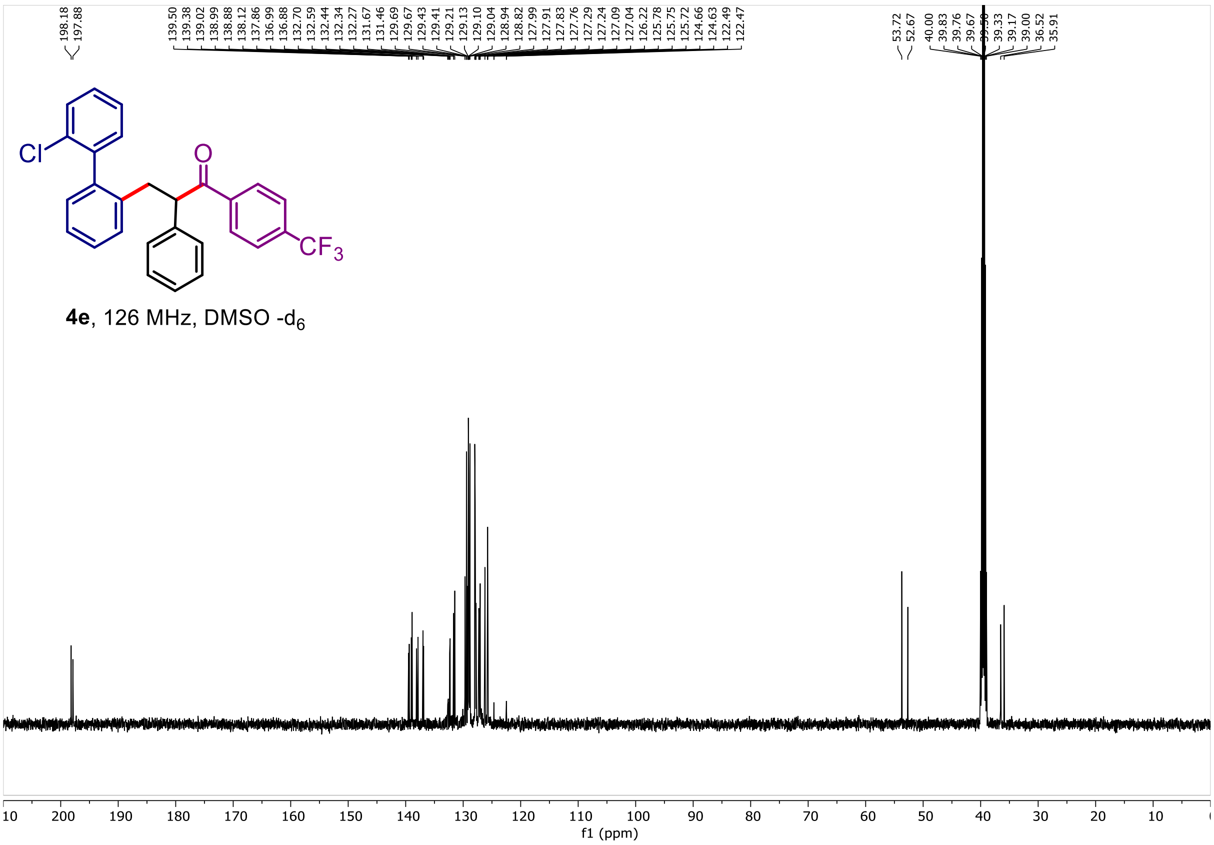

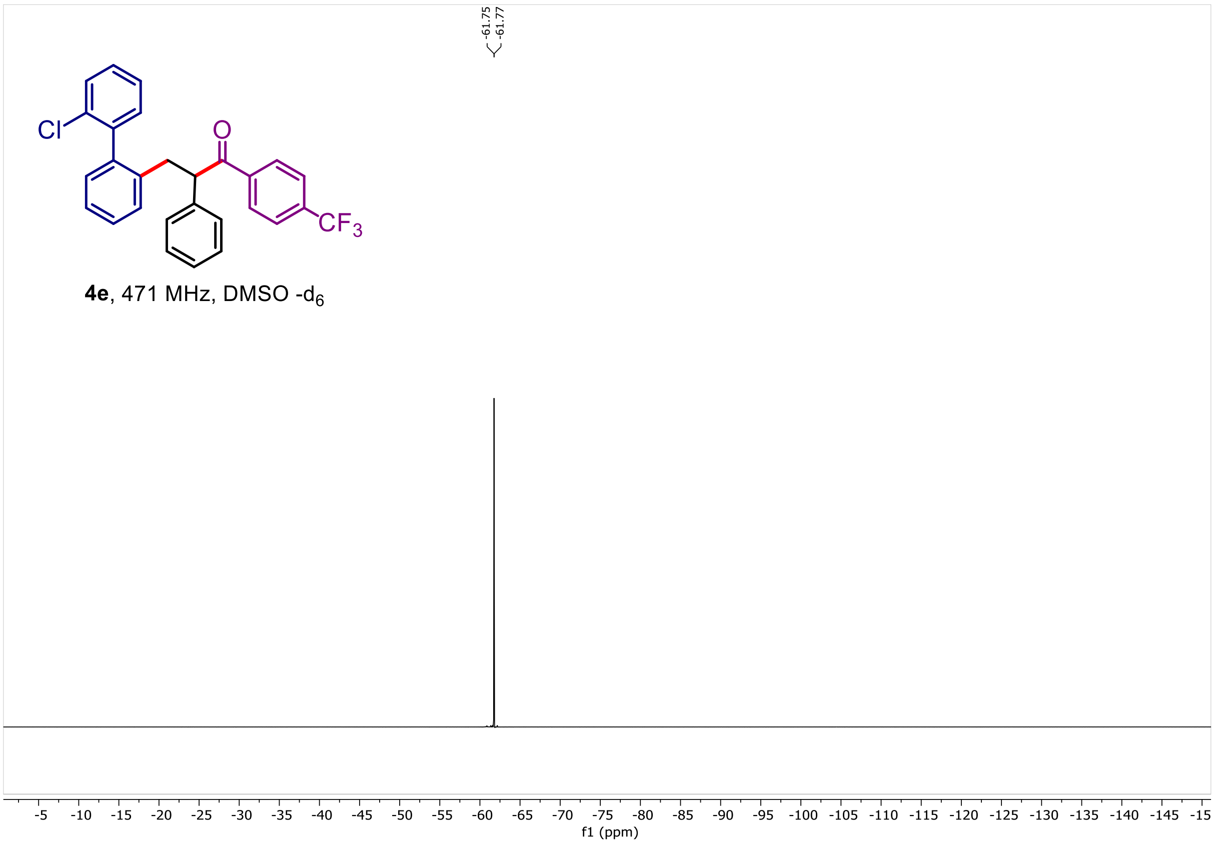

3-(2'-chloro-[1,1'-biphenyl]-2-yl)-1-(4-chlorophenyl)-2-phenylpropan-1-one(4f)

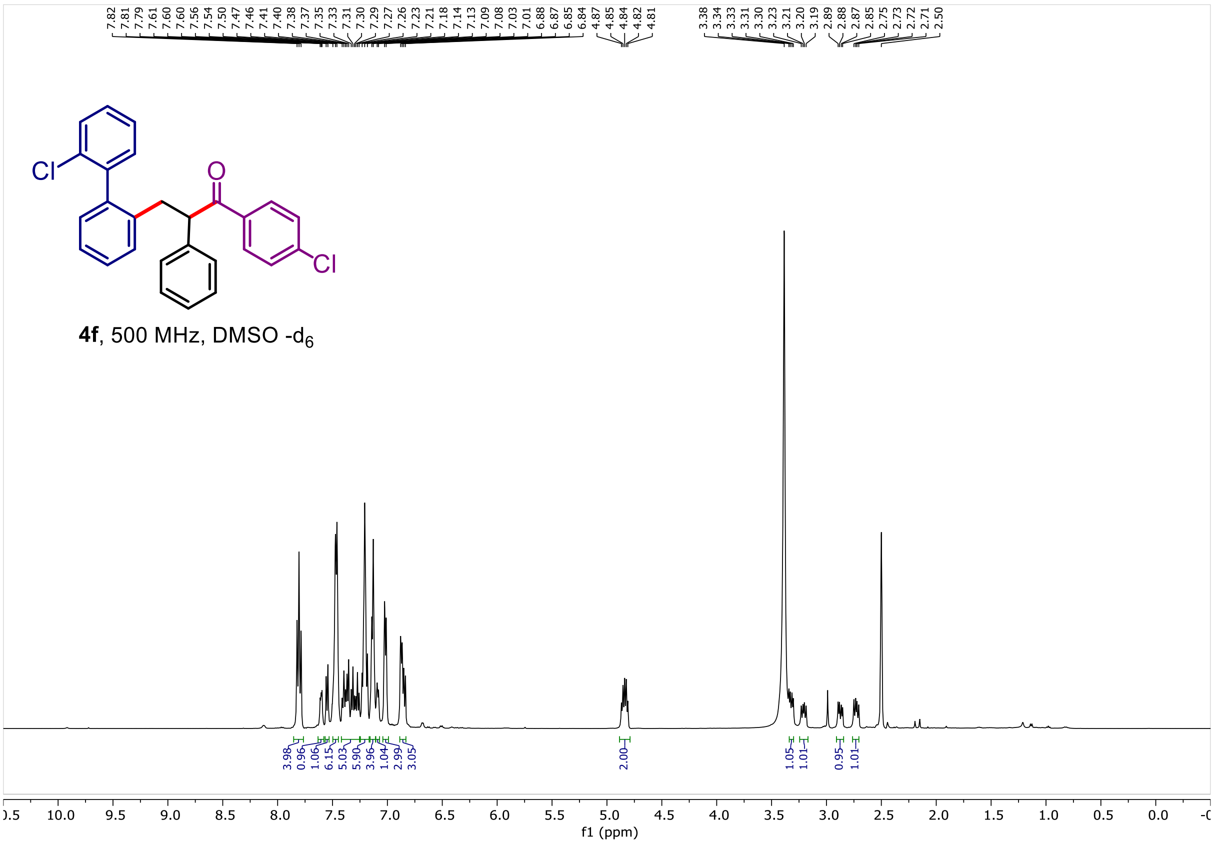

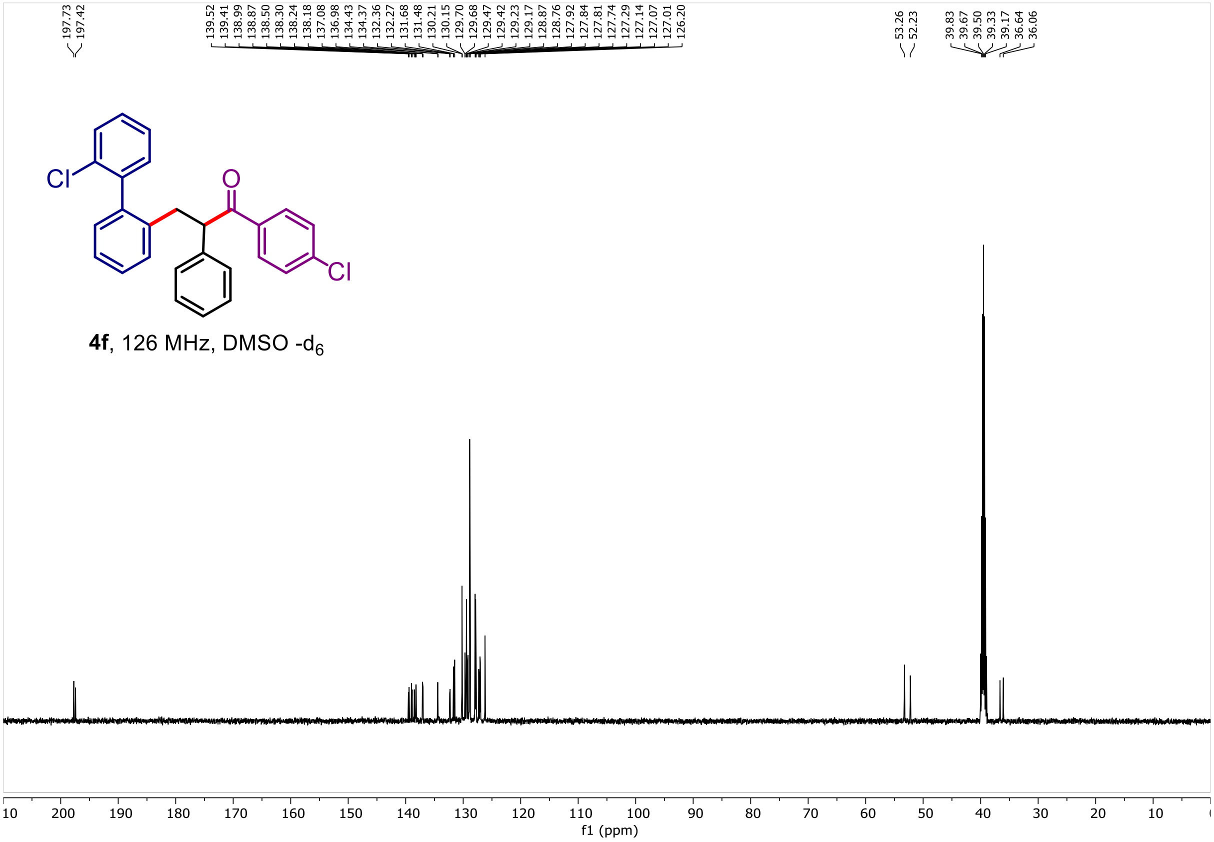

3-(2'-chloro-[1,1'-biphenyl]-2-yl)-1-(4-fluorophenyl)-2-phenylpropan-1-one(4g)

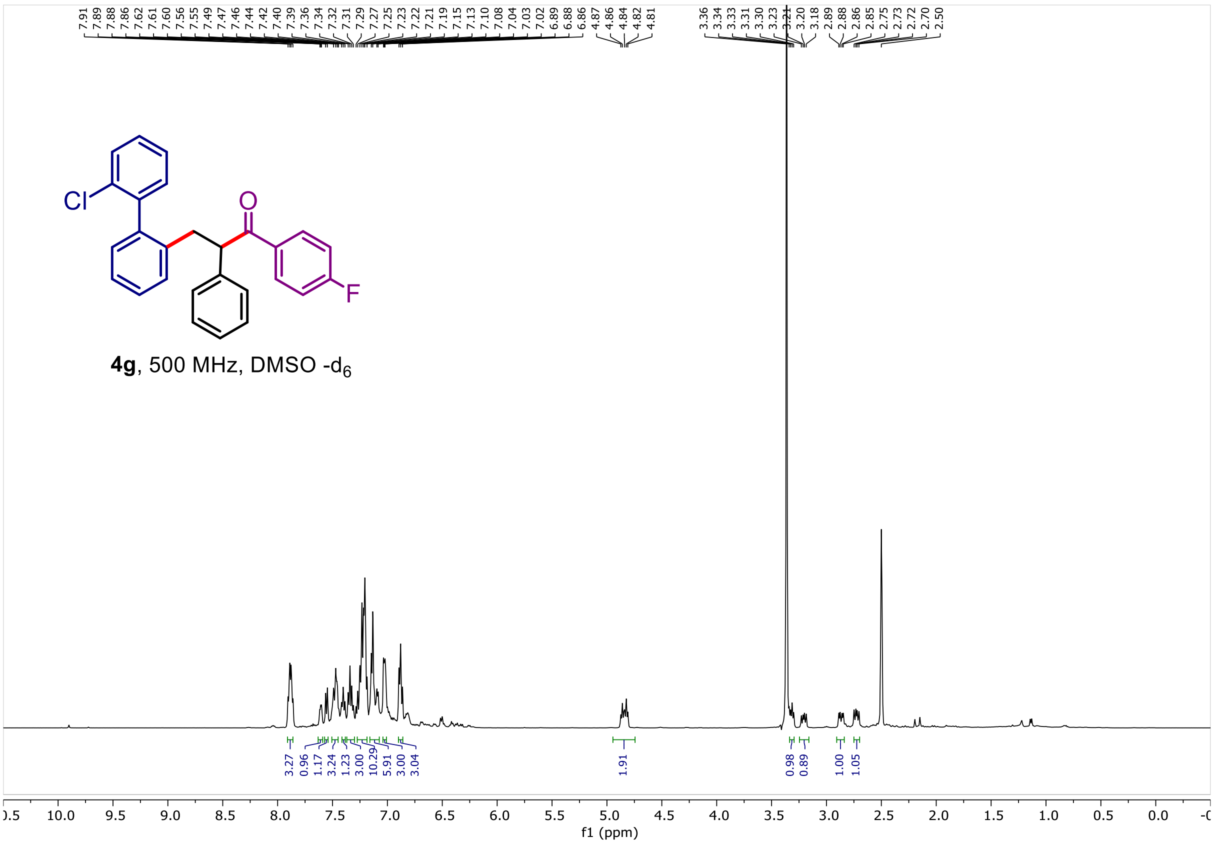

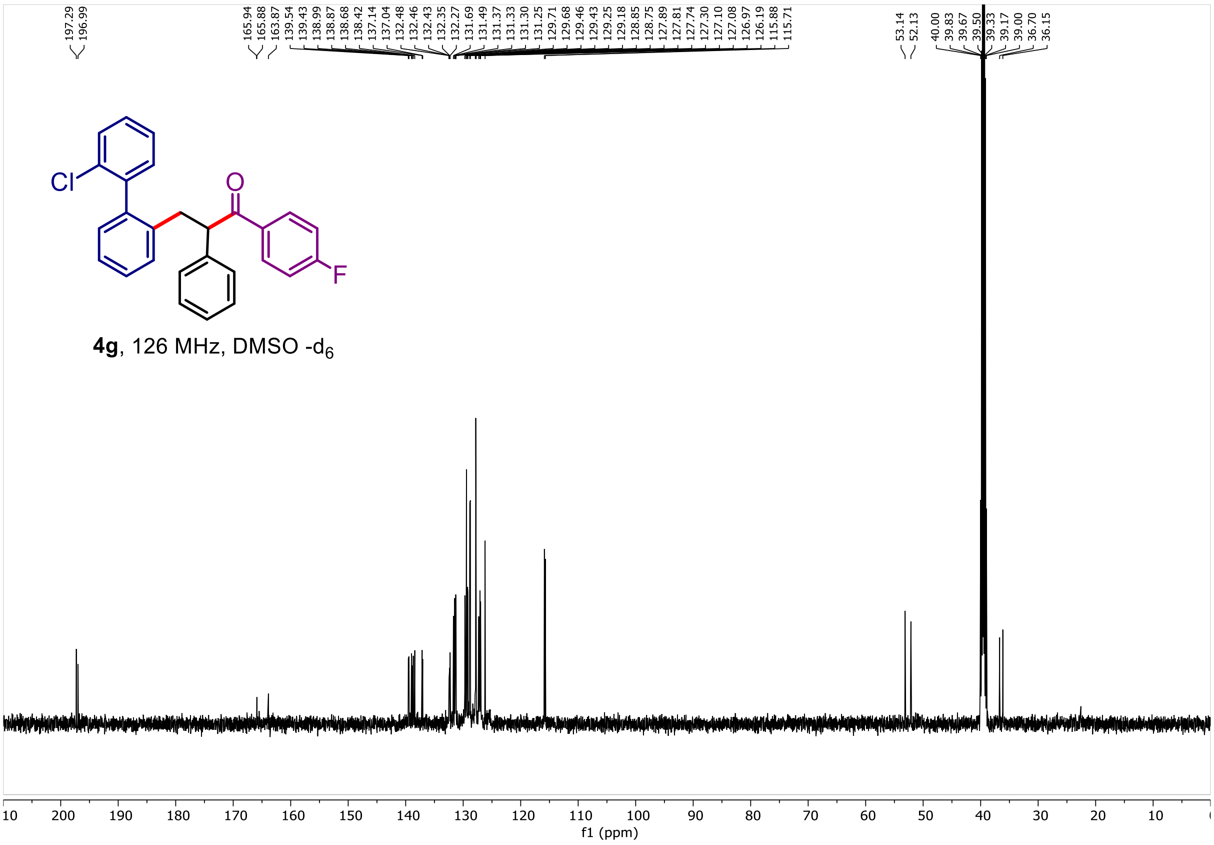
197.29
196.99
165.94
165.88
163.87
139.54
139.43
138.99
138.87
138.68
138.42
137.14
137.04
132.48
132.46
132.43
132.35
132.27
131.69
131.49
131.37
131.33
131.30
131.25
129.71
129.68
129.46
129.43
129.25
129.18
128.85
128.75
127.89
127.81
127.74
127.30
127.10
127.08
126.97
126.19
115.88
115.71
53.14
52.13
40.00
39.83
39.67
39.50
39.33
39.17
39.00
36.70
36.15
Cl
O
F
4g, 126 MHz, DMSO -d$_6$
f1 (ppm)

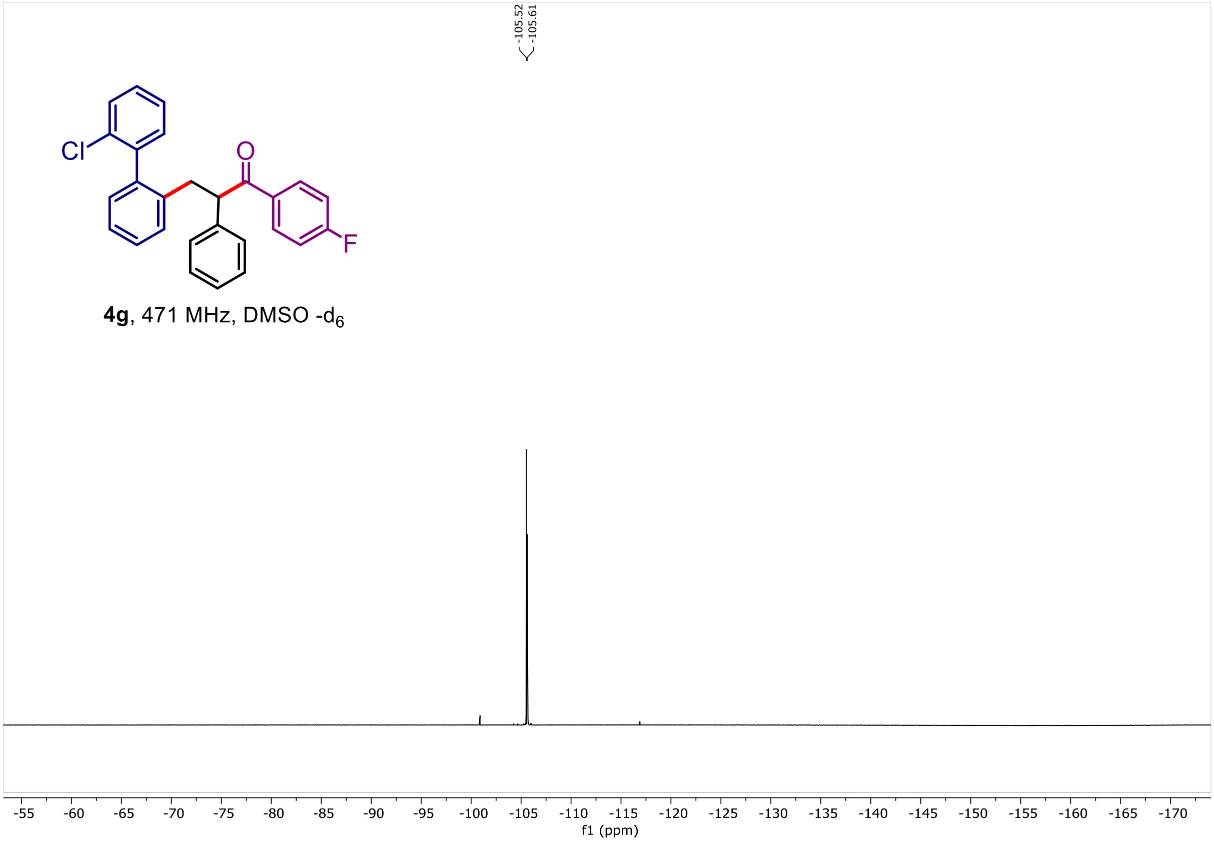
-105.52
-105.61
Cl
O
F
4g, 471 MHz, DMSO -d$_6$
f1 (ppm)

3-(2'-chloro-[1,1'-biphenyl]-2-yl)-2-phenyl-1-(p-tolyl)propan-1-one(4h)

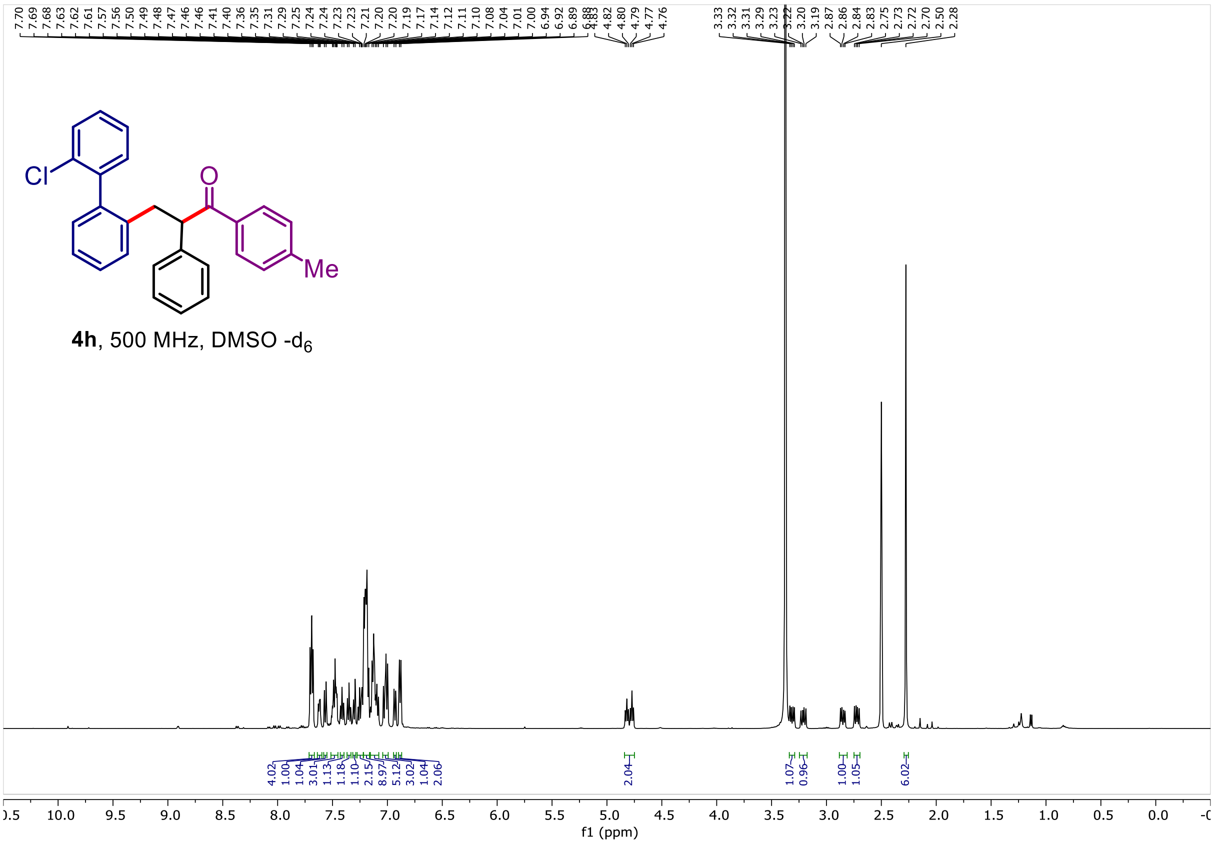

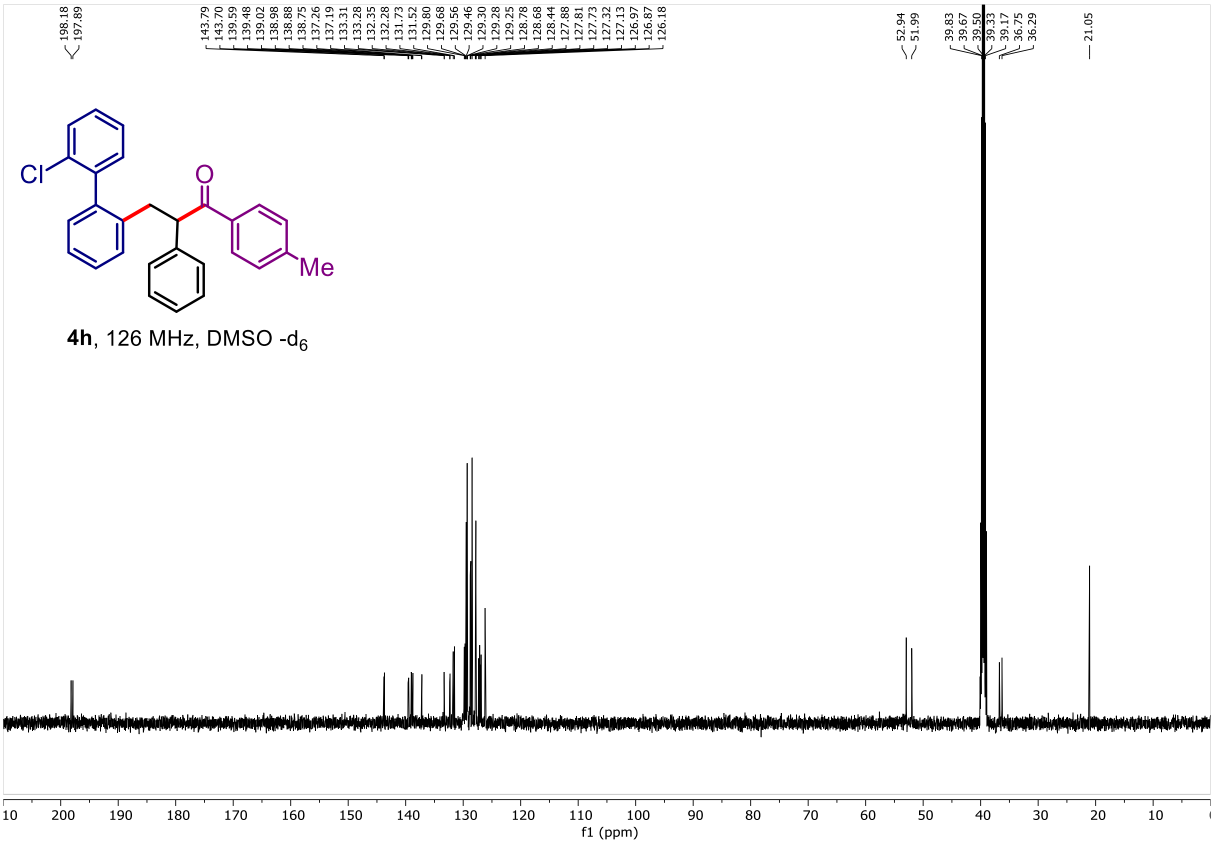

3-(2'-chloro-[1,1'-biphenyl]-2-yl)-1-(4-ethylphenyl)-2-phenylpropan-1-one(4i)

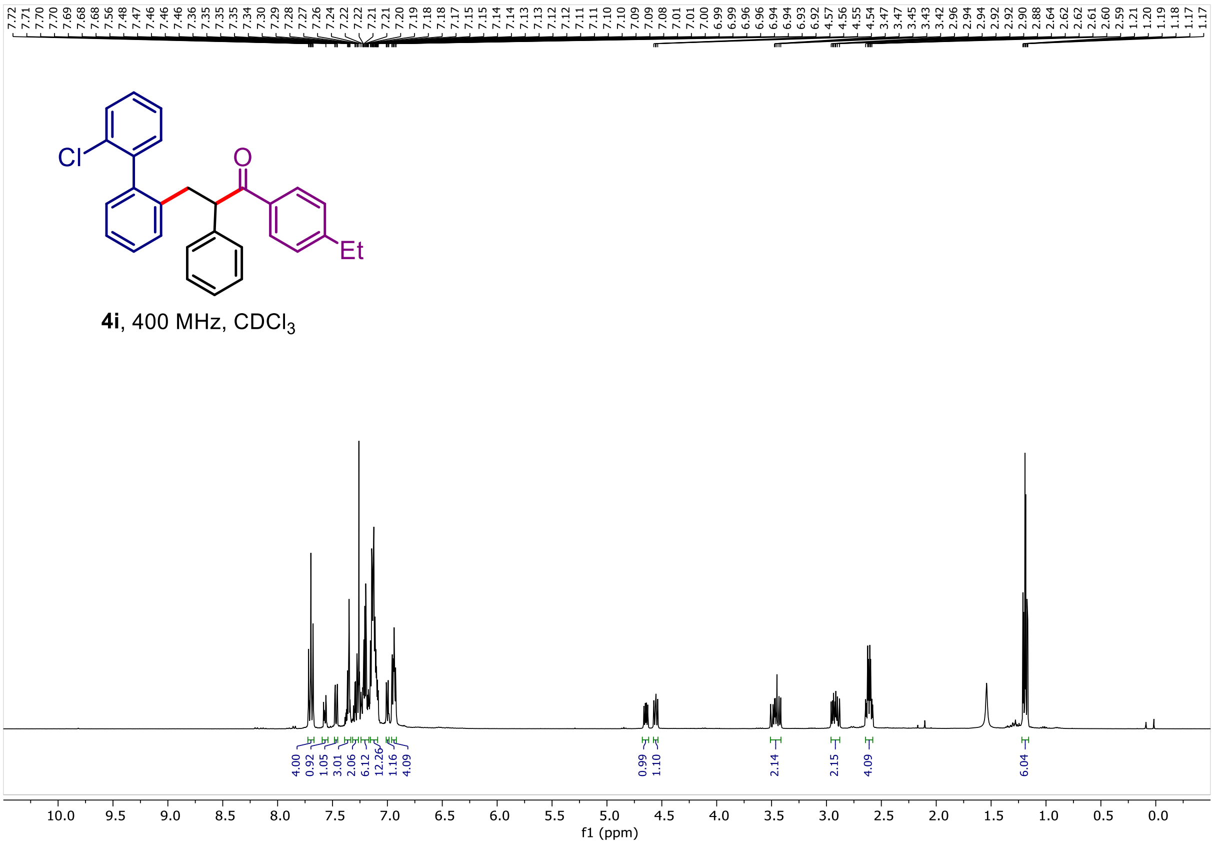

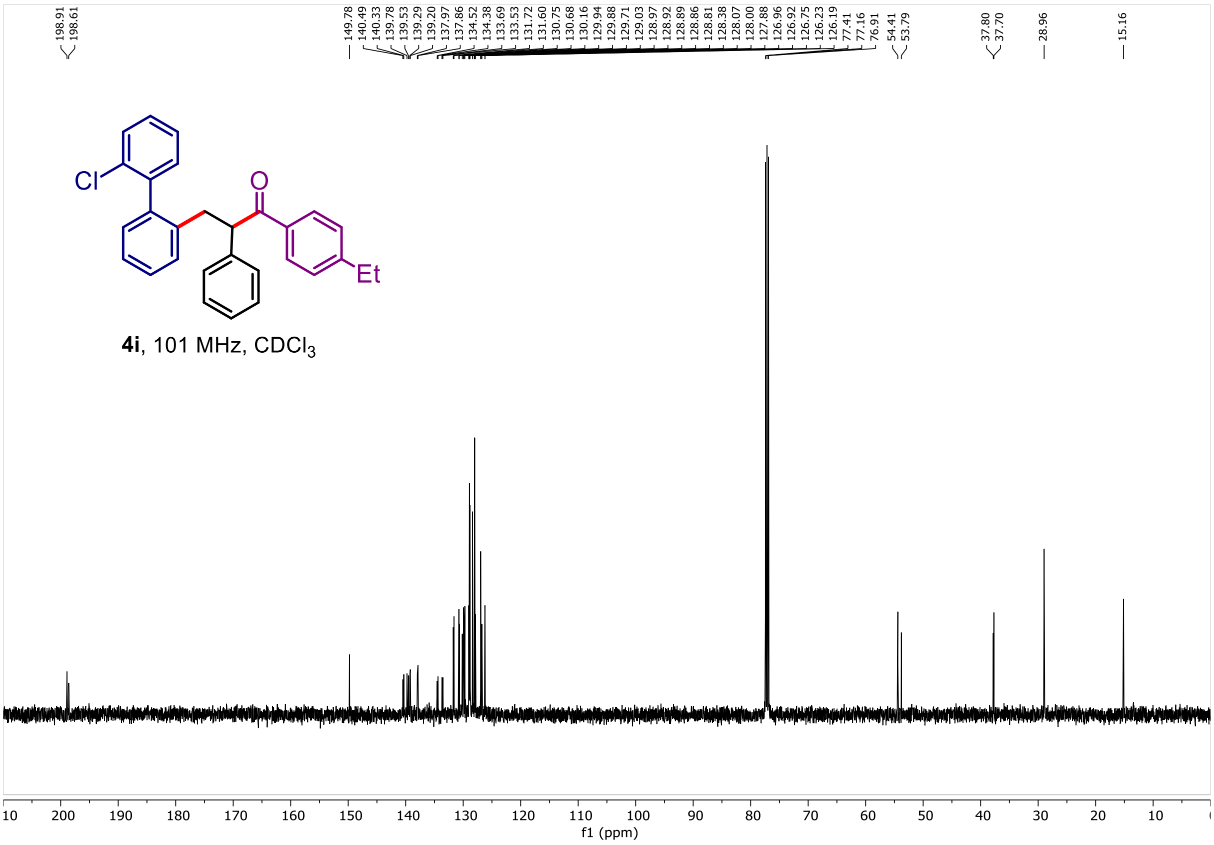

1-(4-(tert-butyl)phenyl)-3-(2'-chloro-[1,1'-biphenyl]-2-yl)-2-phenylpropan-1-one (4j)

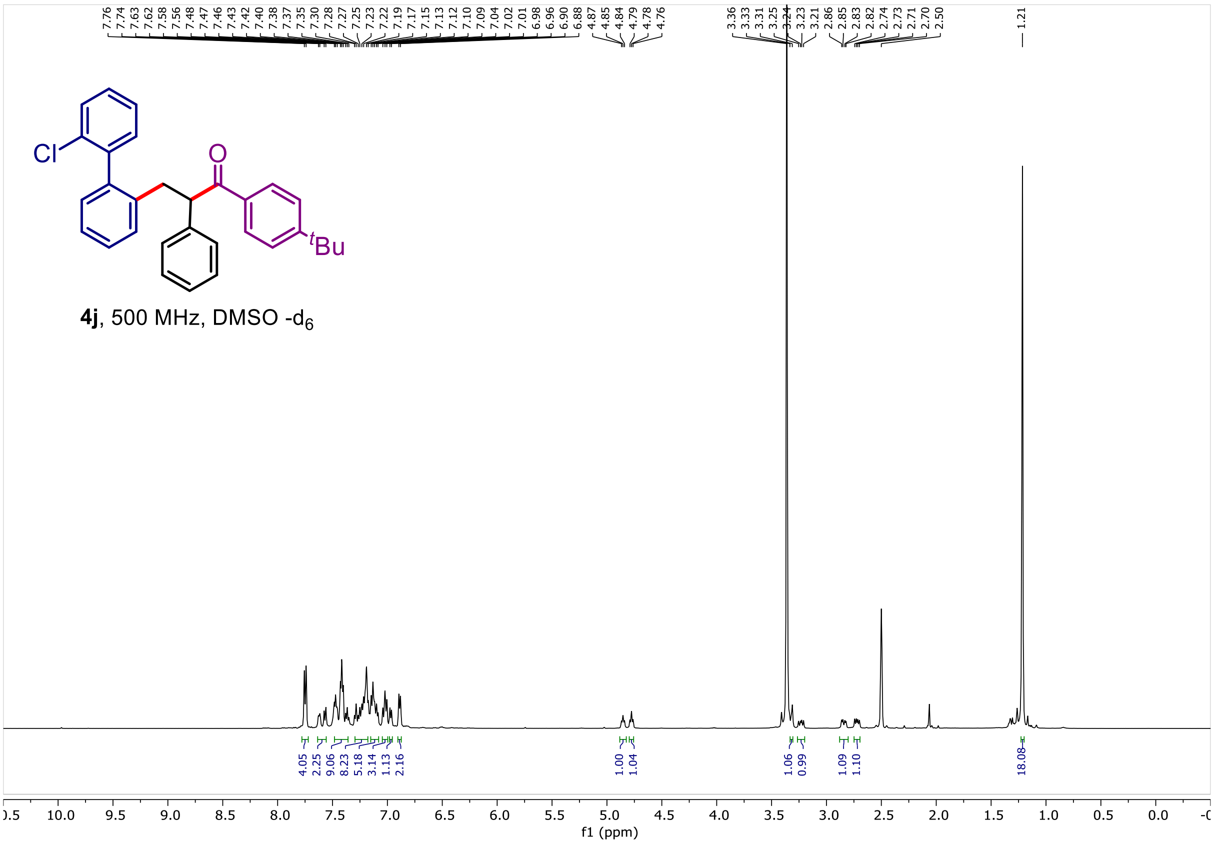

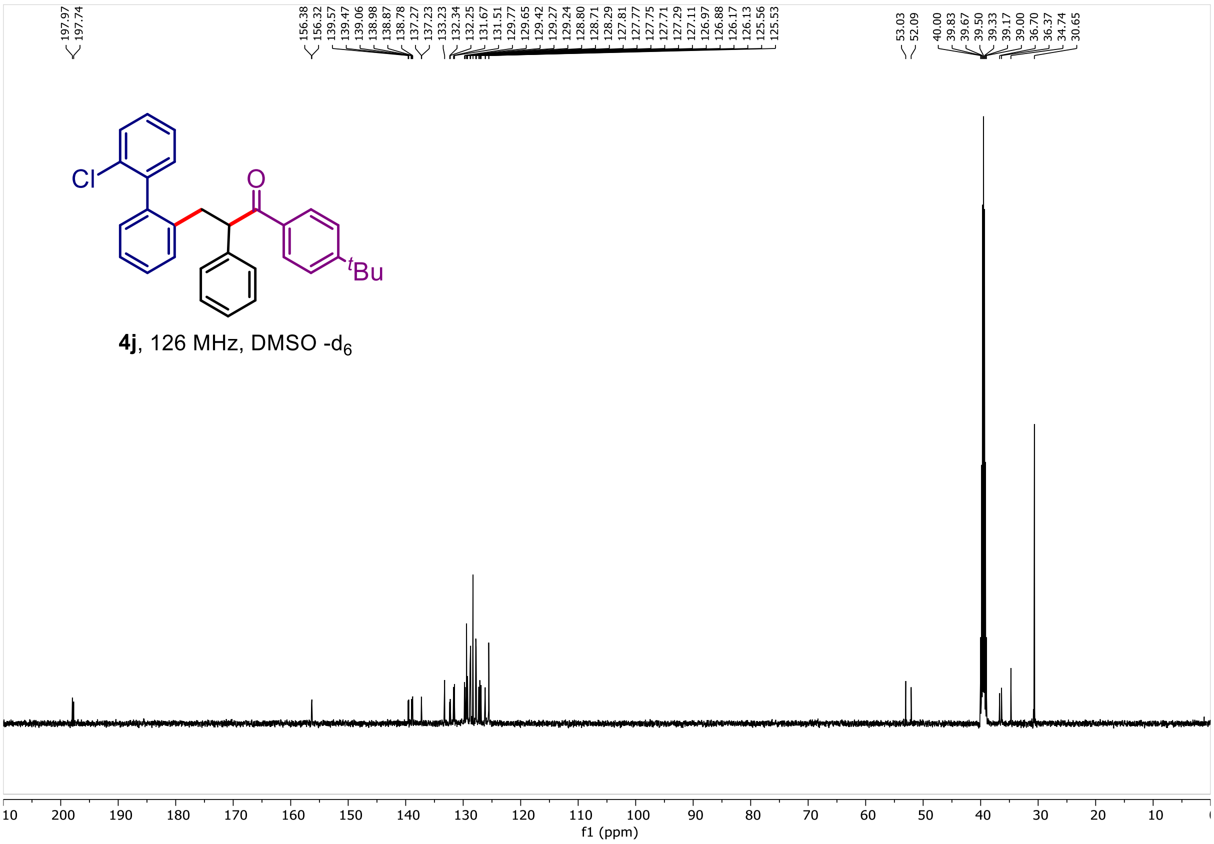

1-([1,1'-biphenyl]-4-yl)-3-(2'-chloro-[1,1'-biphenyl]-2-yl)-2-phenylpropan-1-one (4k)

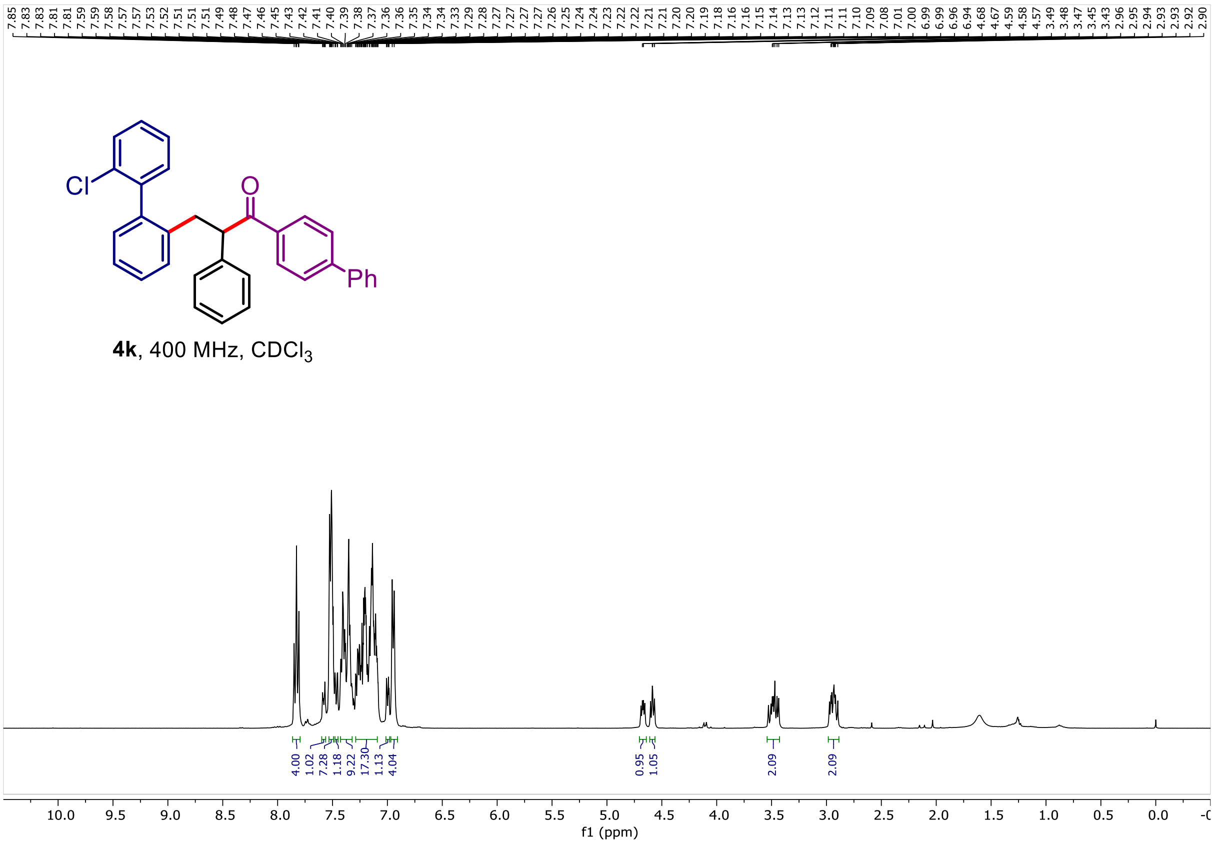

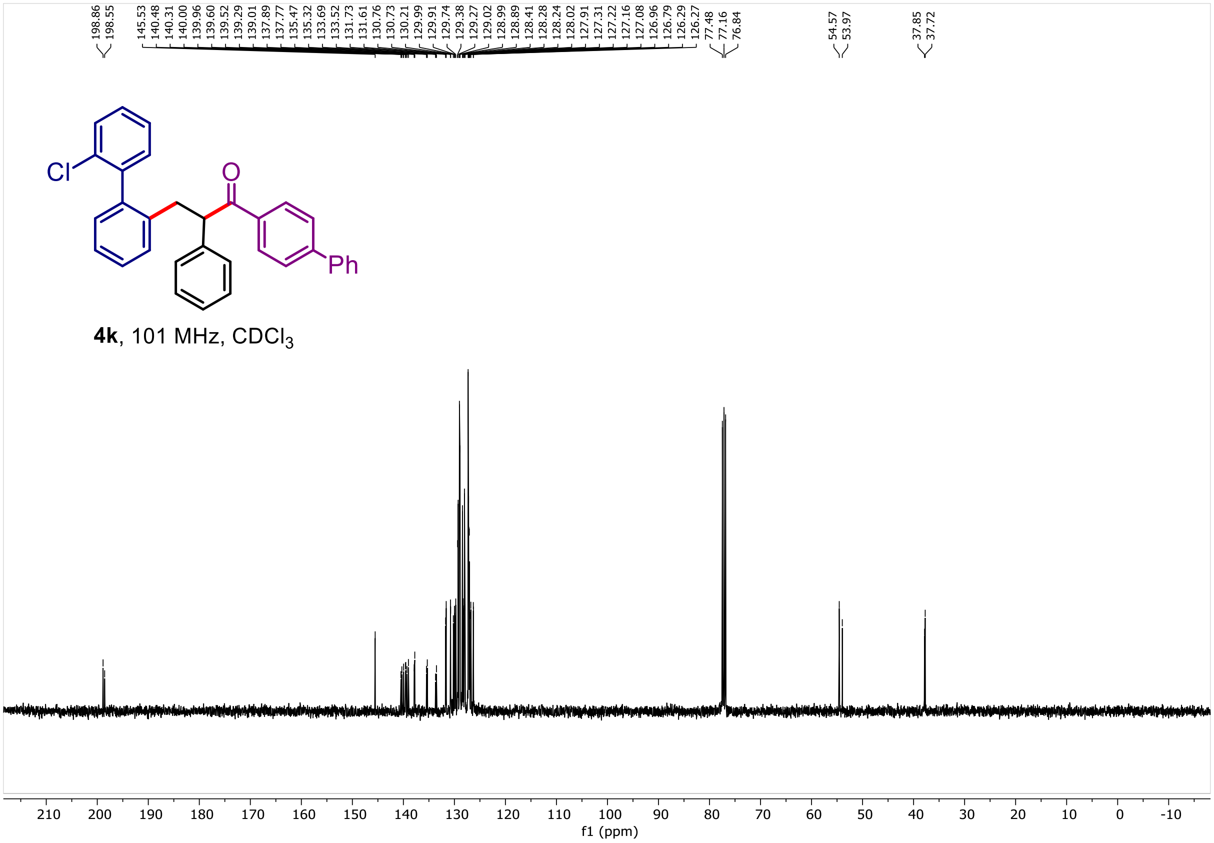

3-(2'-chloro-[1,1'-biphenyl]-2-yl)-1-(3-nitrophenyl)-2-phenylpropan-1-one(4l)

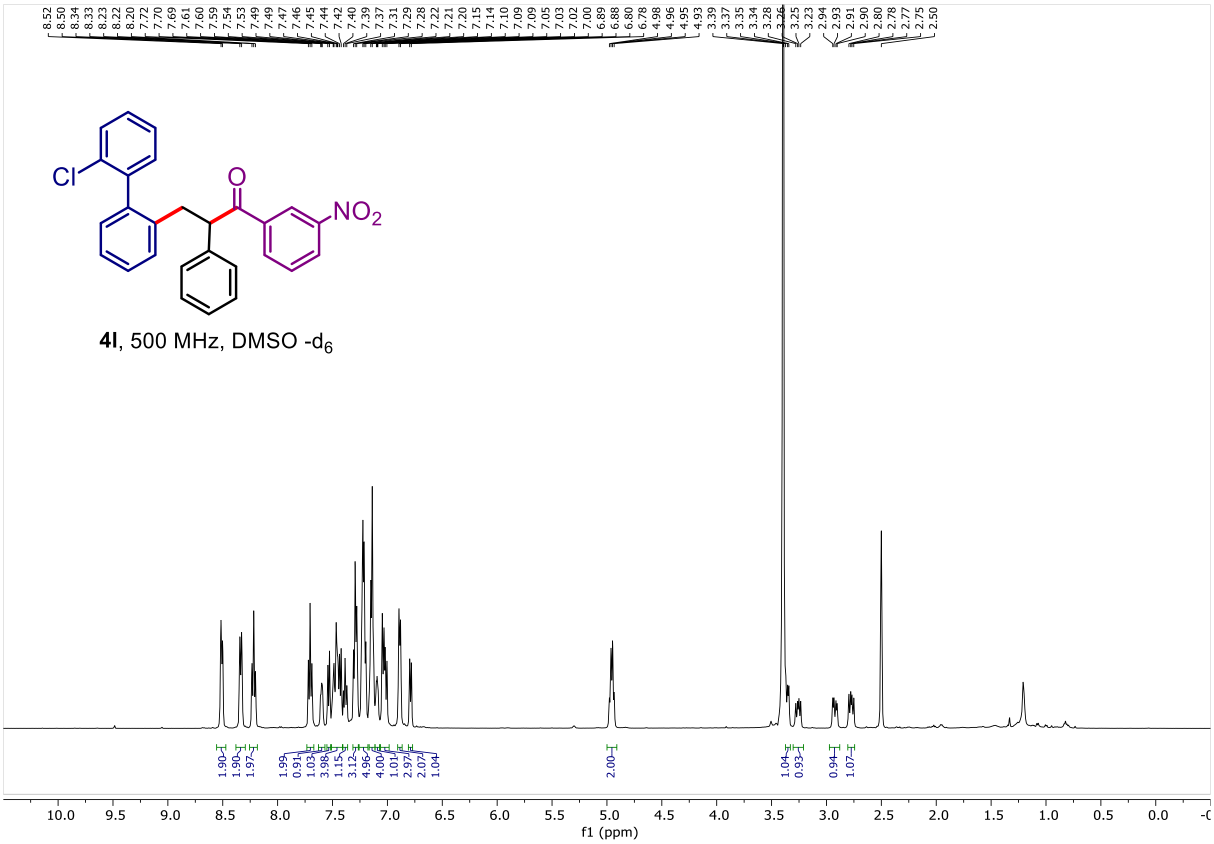

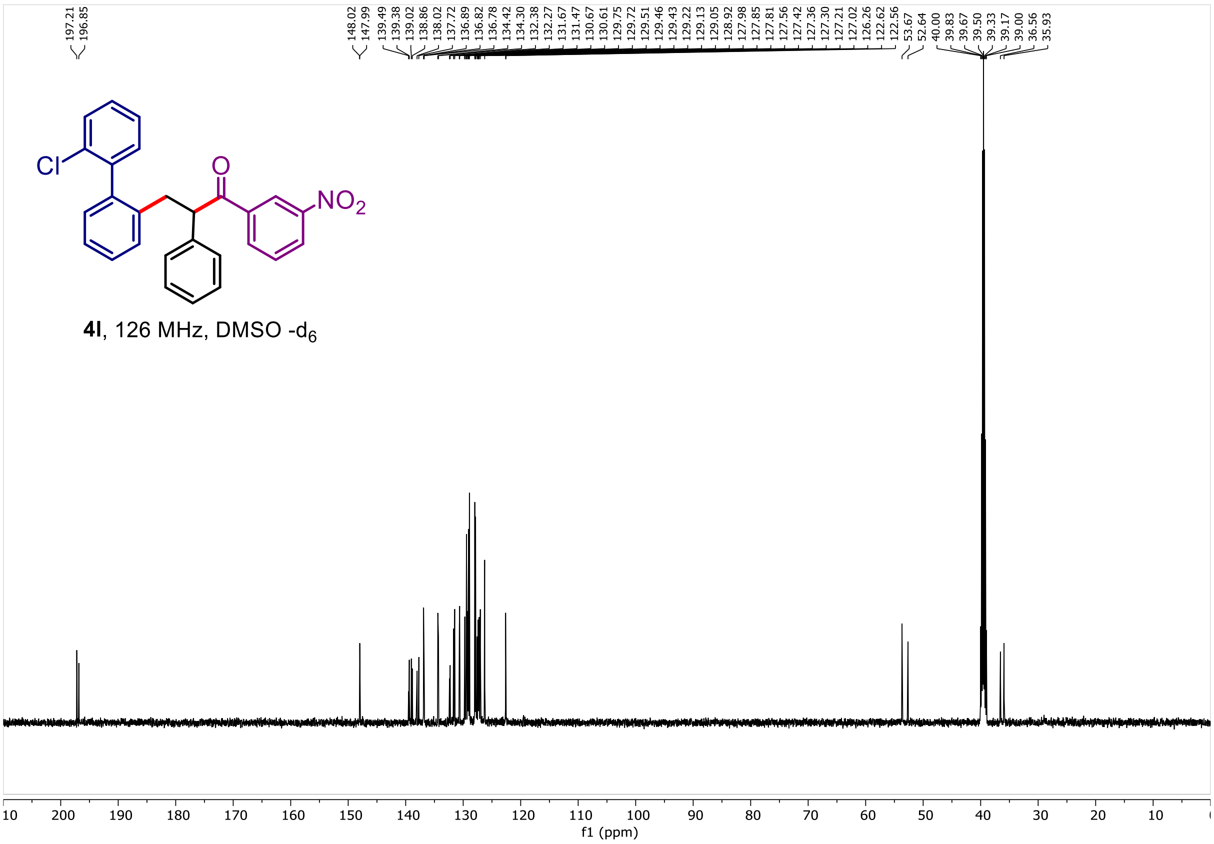

1-(3-bromophenyl)-3-(2'-chloro-[1,1'-biphenyl]-2-yl)-2-phenylpropan-1-one (4m)

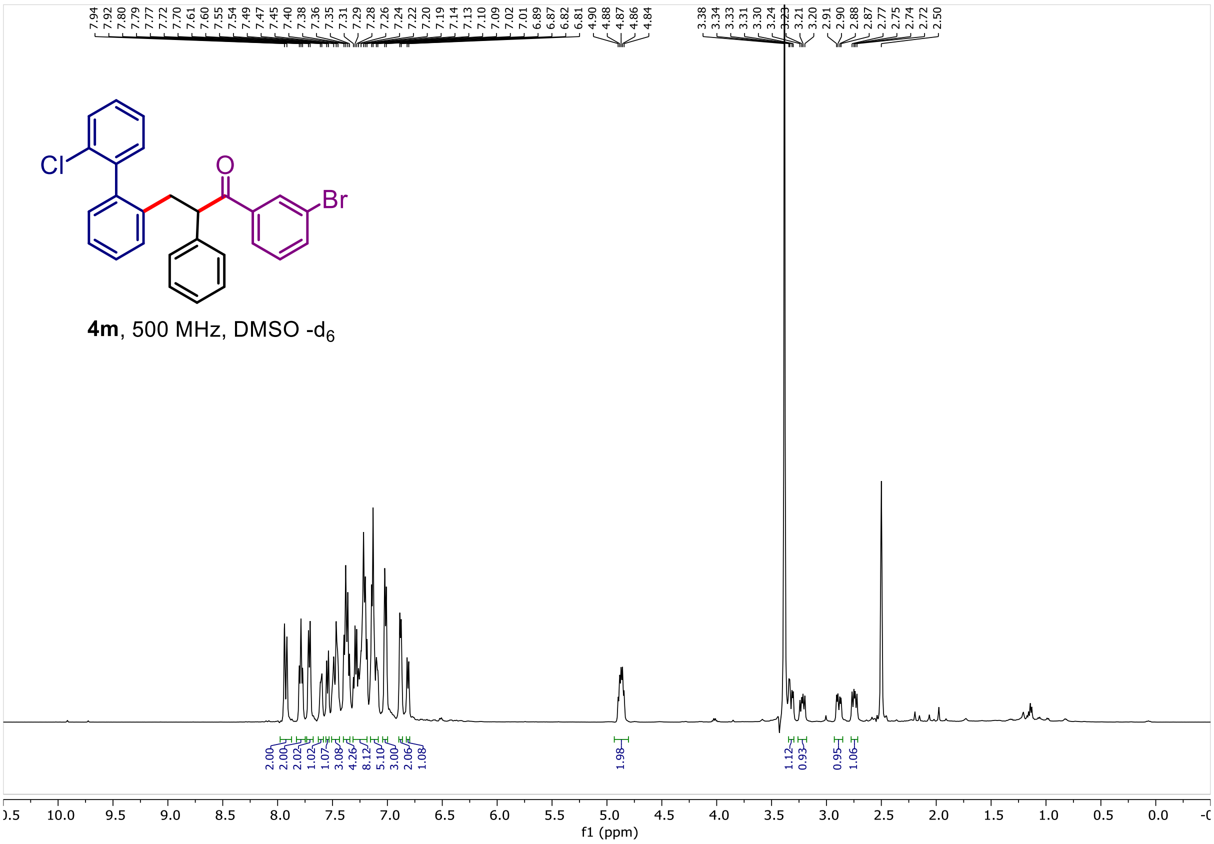

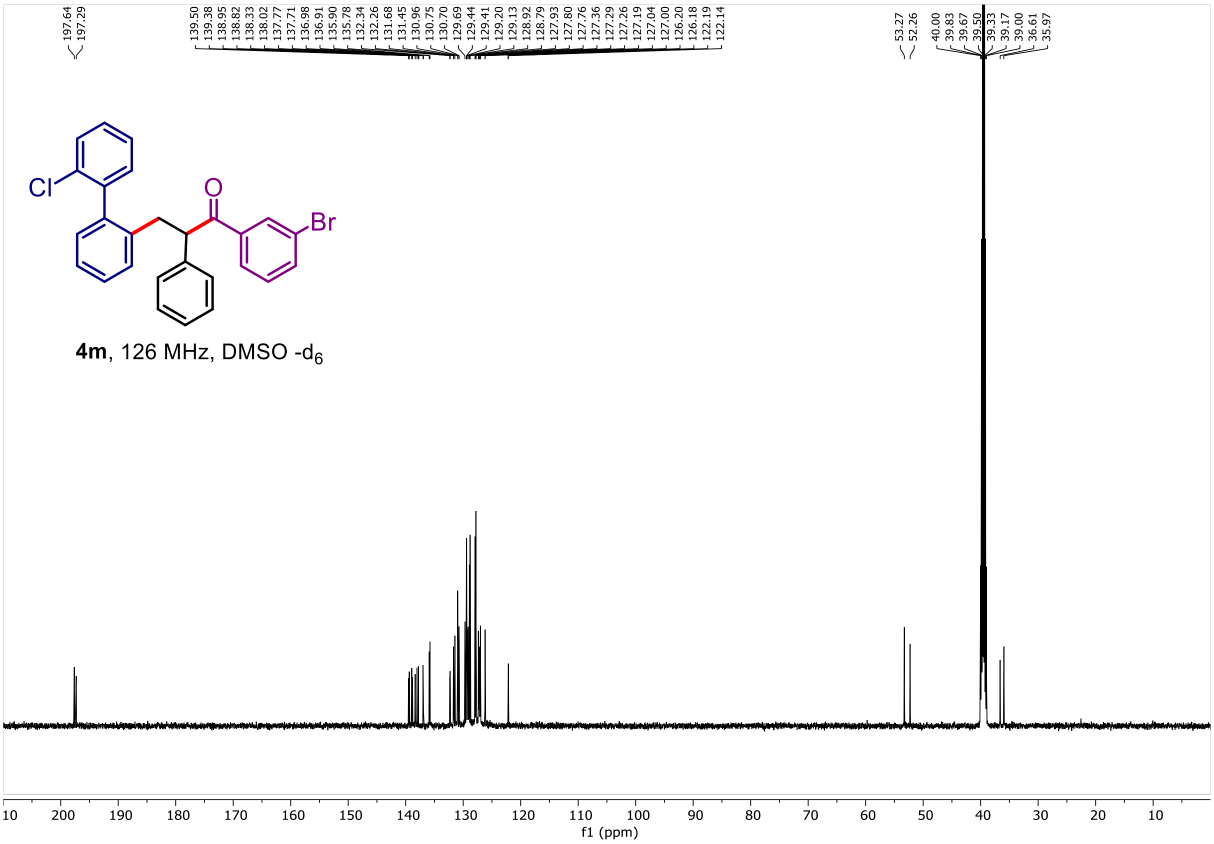

3-(2'-chloro-[1,1'-biphenyl]-2-yl)-1-(3-chlorophenyl)-2-phenylpropan-1-one (4n)

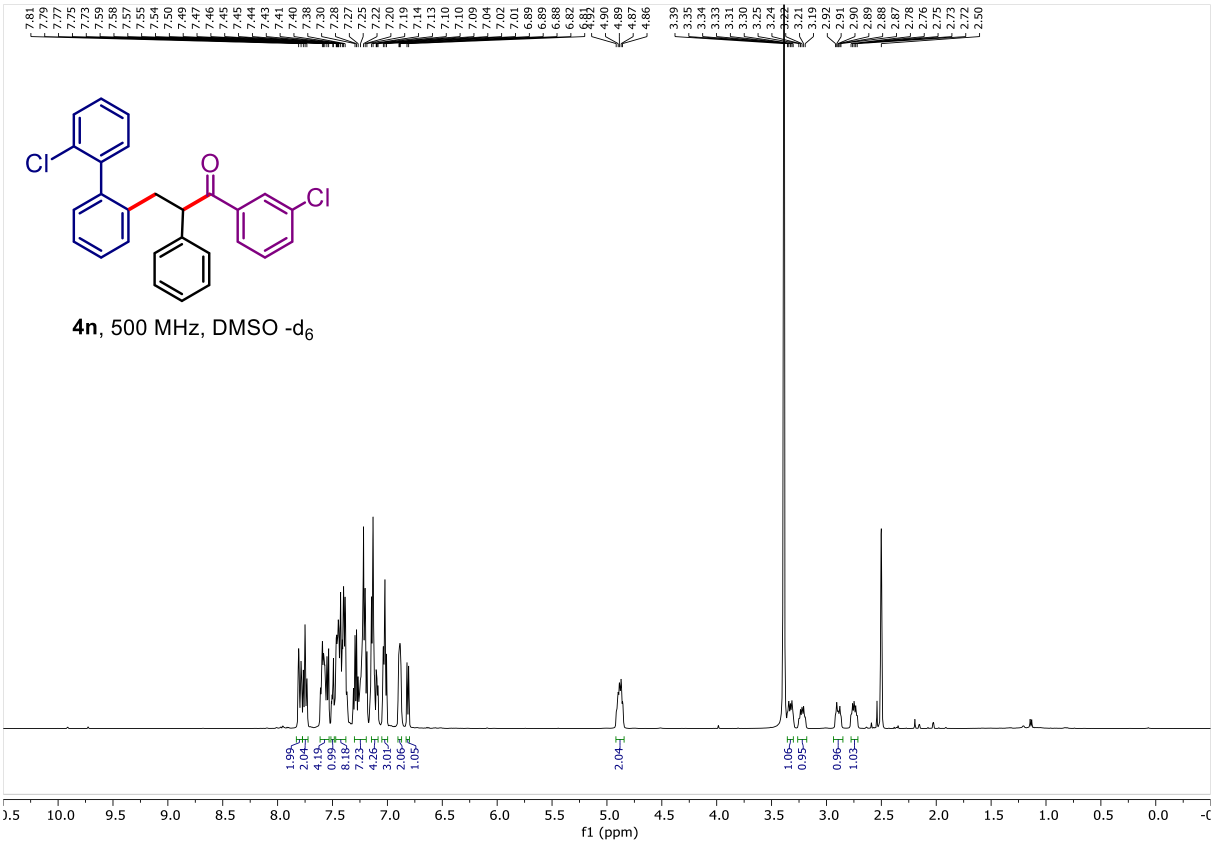

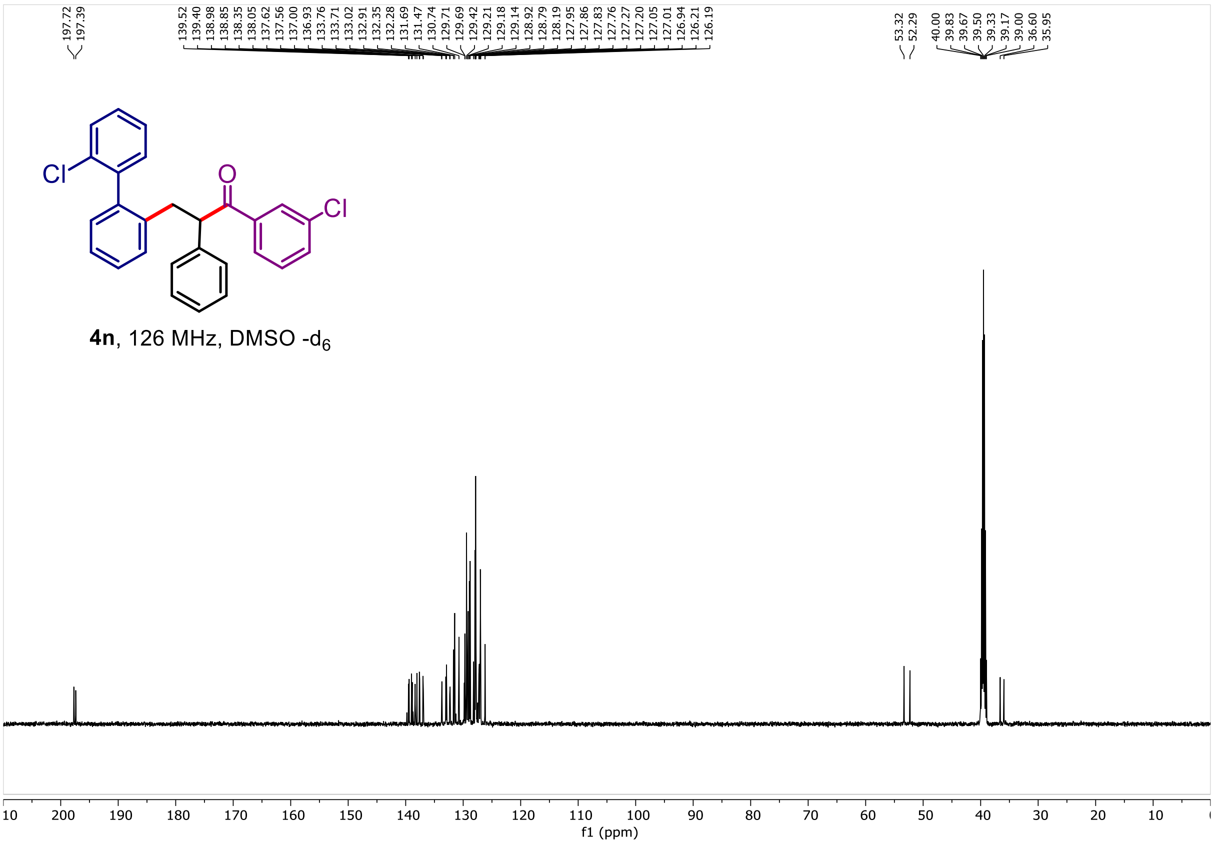

3-(2'-chloro-[1,1'-biphenyl]-2-yl)-1-(3-fluorophenyl)-2-phenylpropan-1-one (4o)

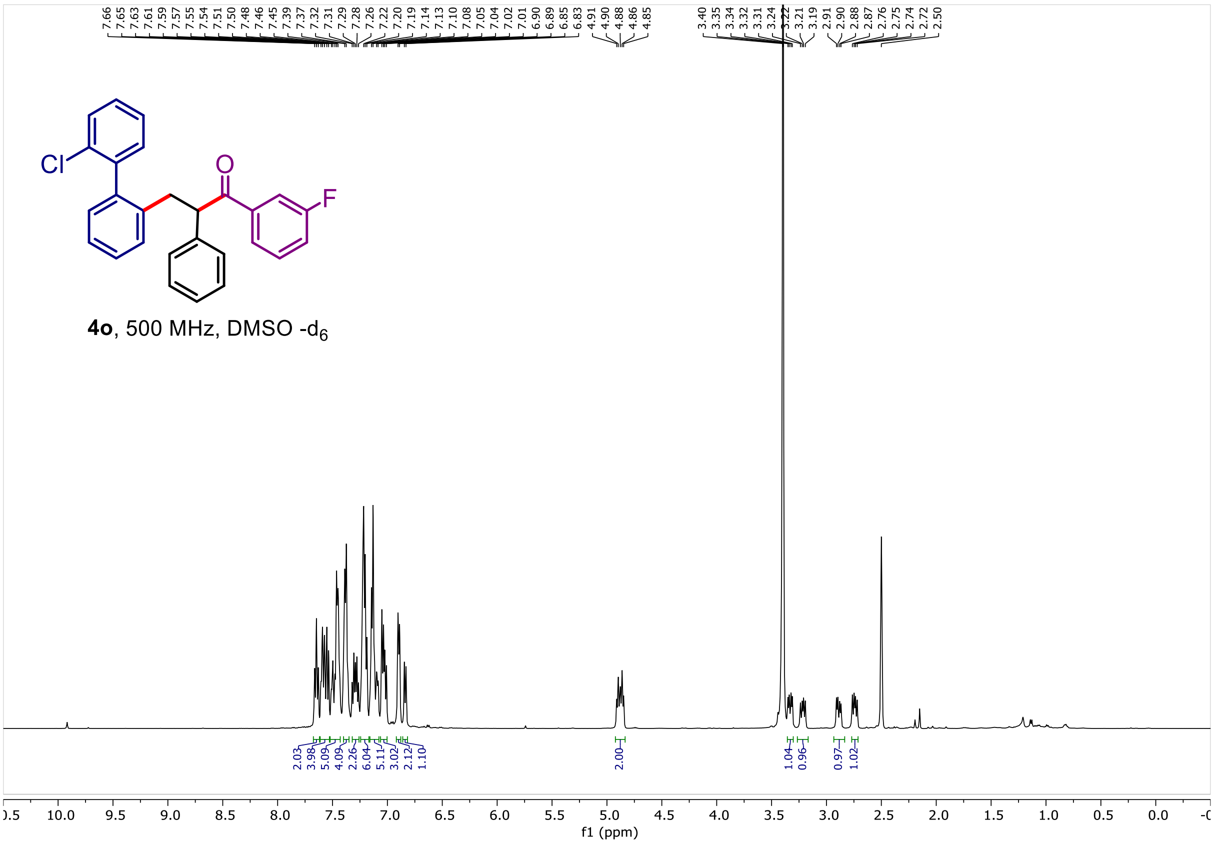

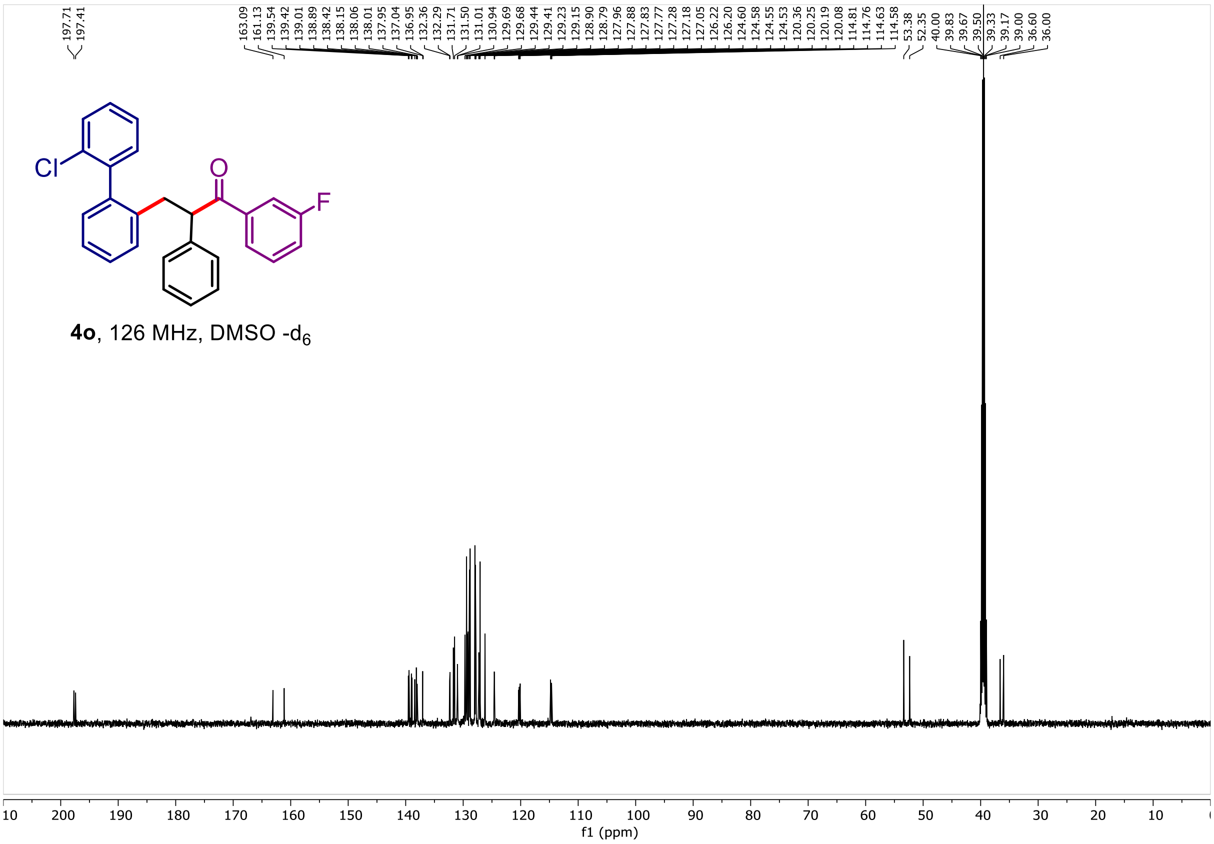

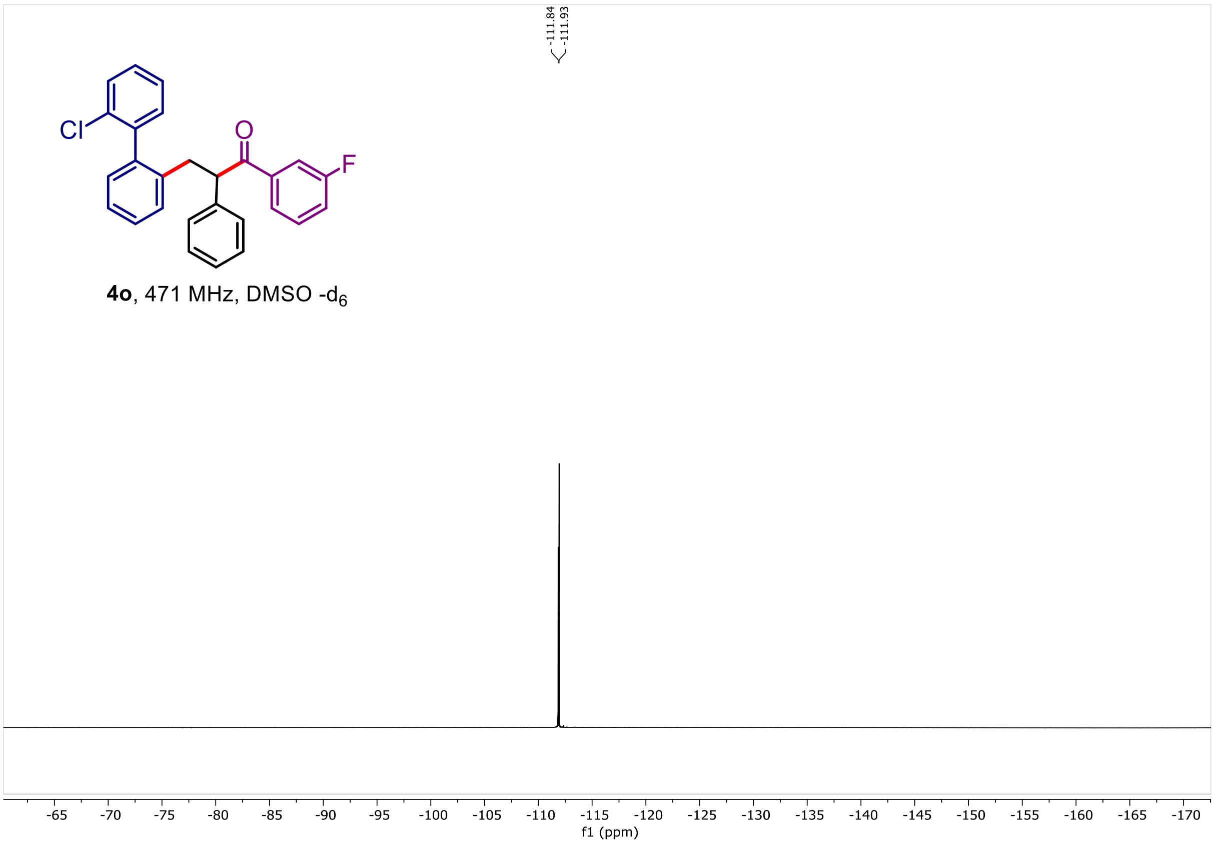

3-(2'-chloro-[1,1'-biphenyl]-2-yl)-2-phenyl-1-(*m*-tolyl)propan-1-one (4p)

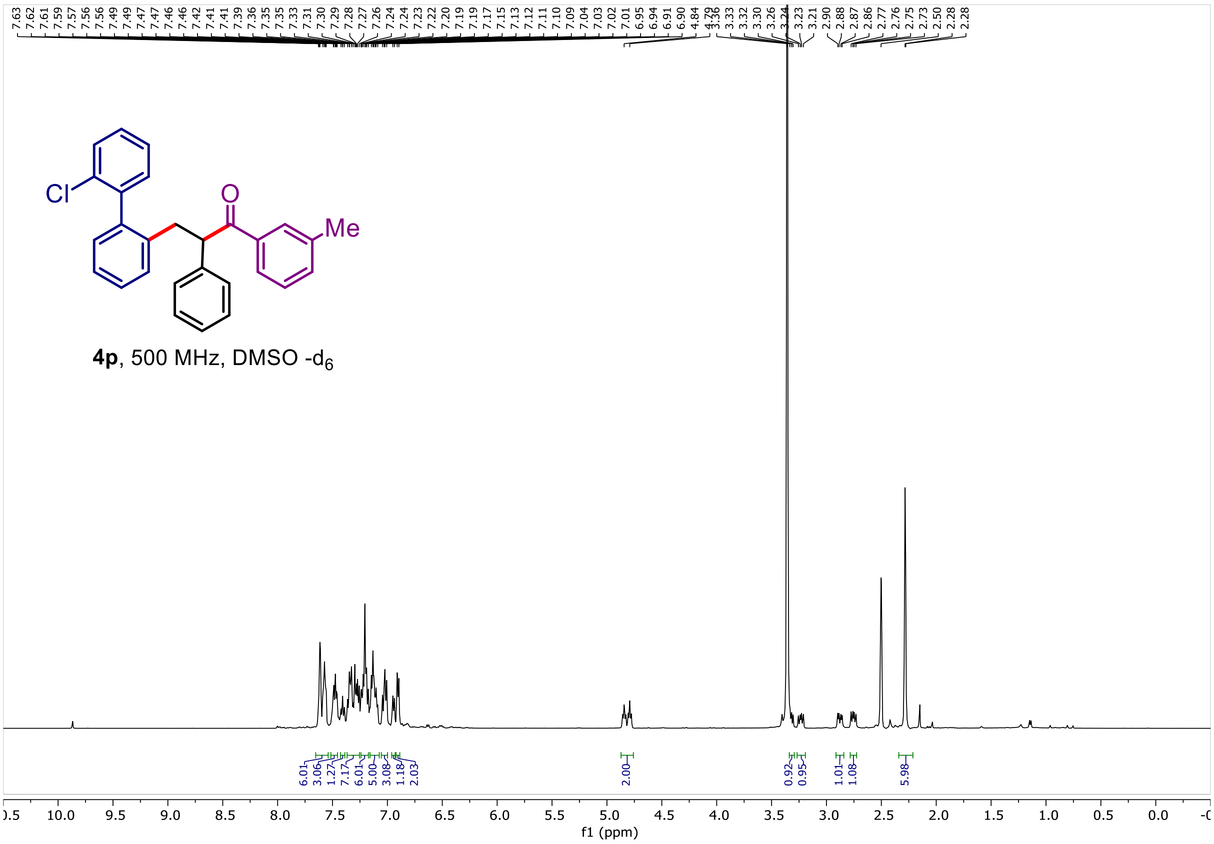

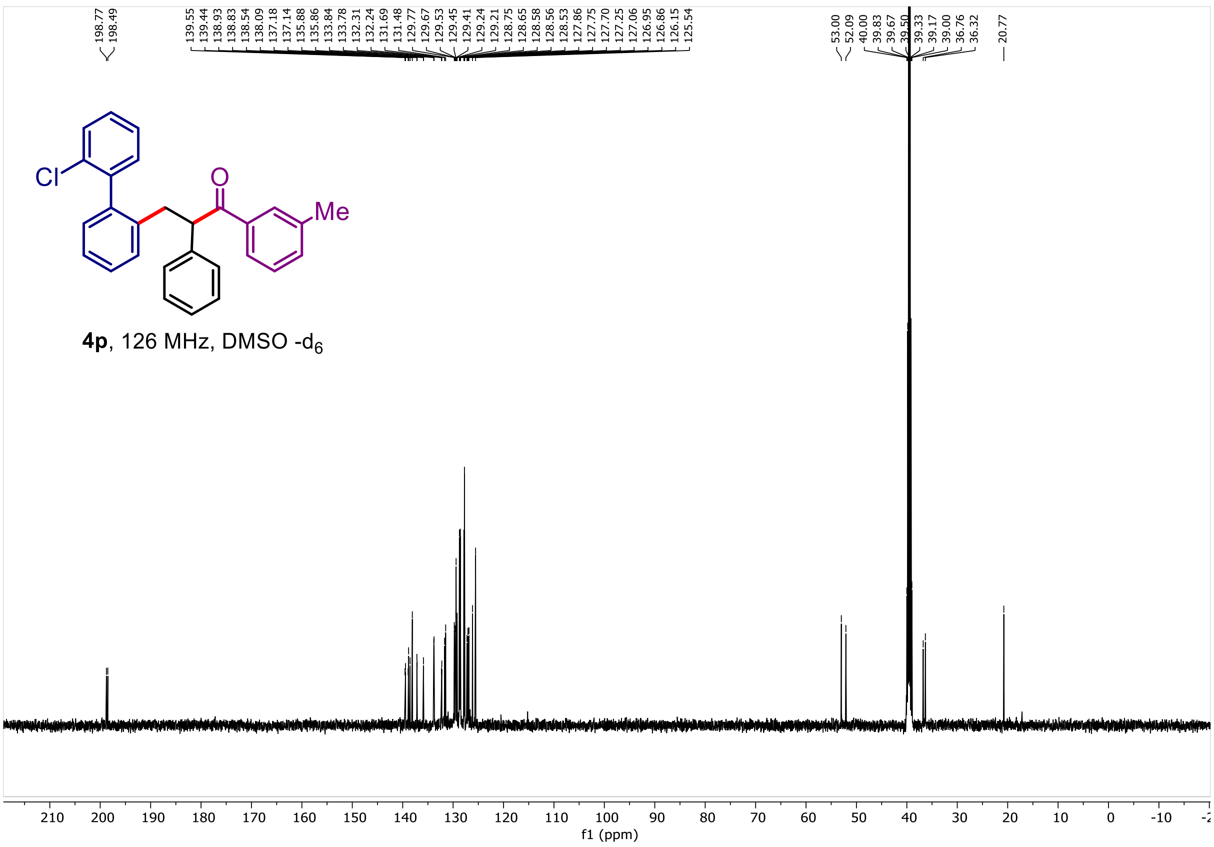

3-(2'-chloro-[1,1'-biphenyl]-2-yl)-1-(3-methoxyphenyl)-2-phenylpropan-1-one(4q)

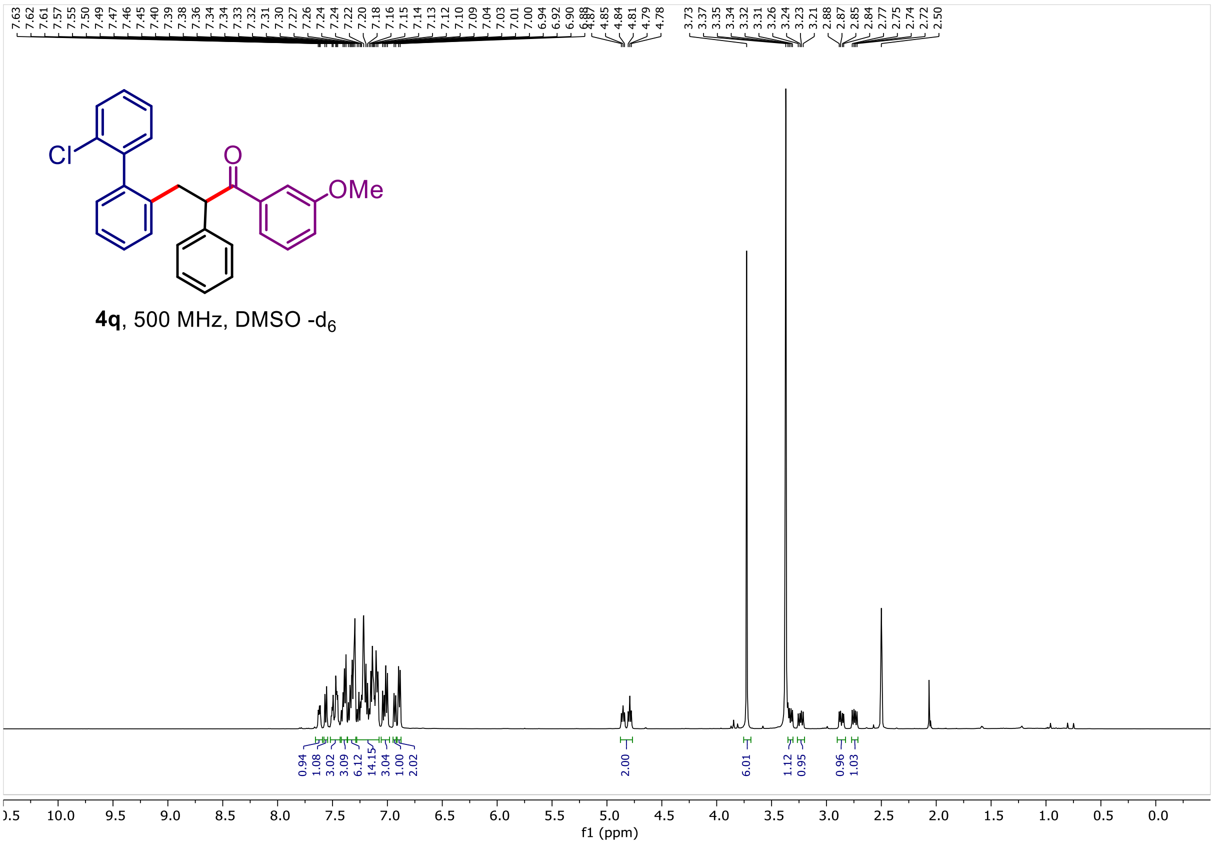

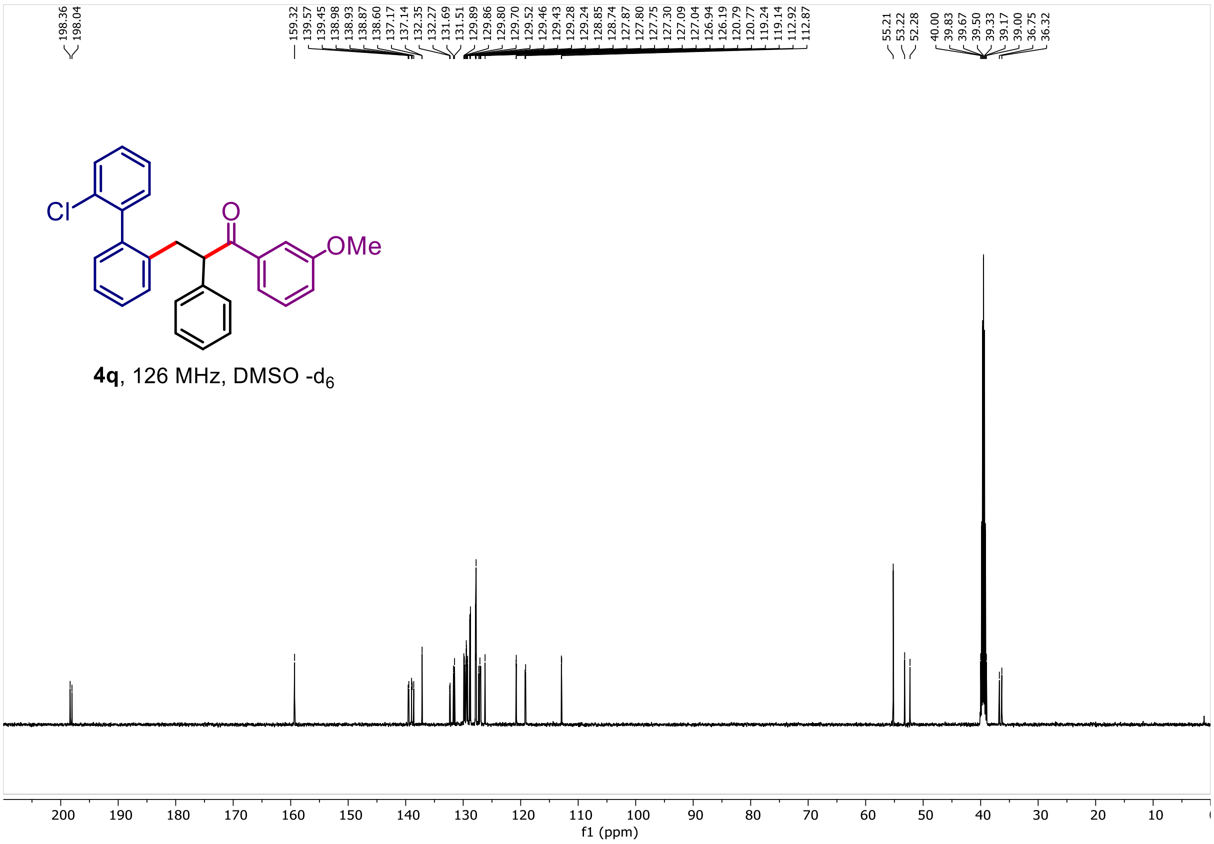

3-(2'-chloro-[1,1'-biphenyl]-2-yl)-1-(3-phenoxyphenyl)-2-phenylpropan-1-one(4r)

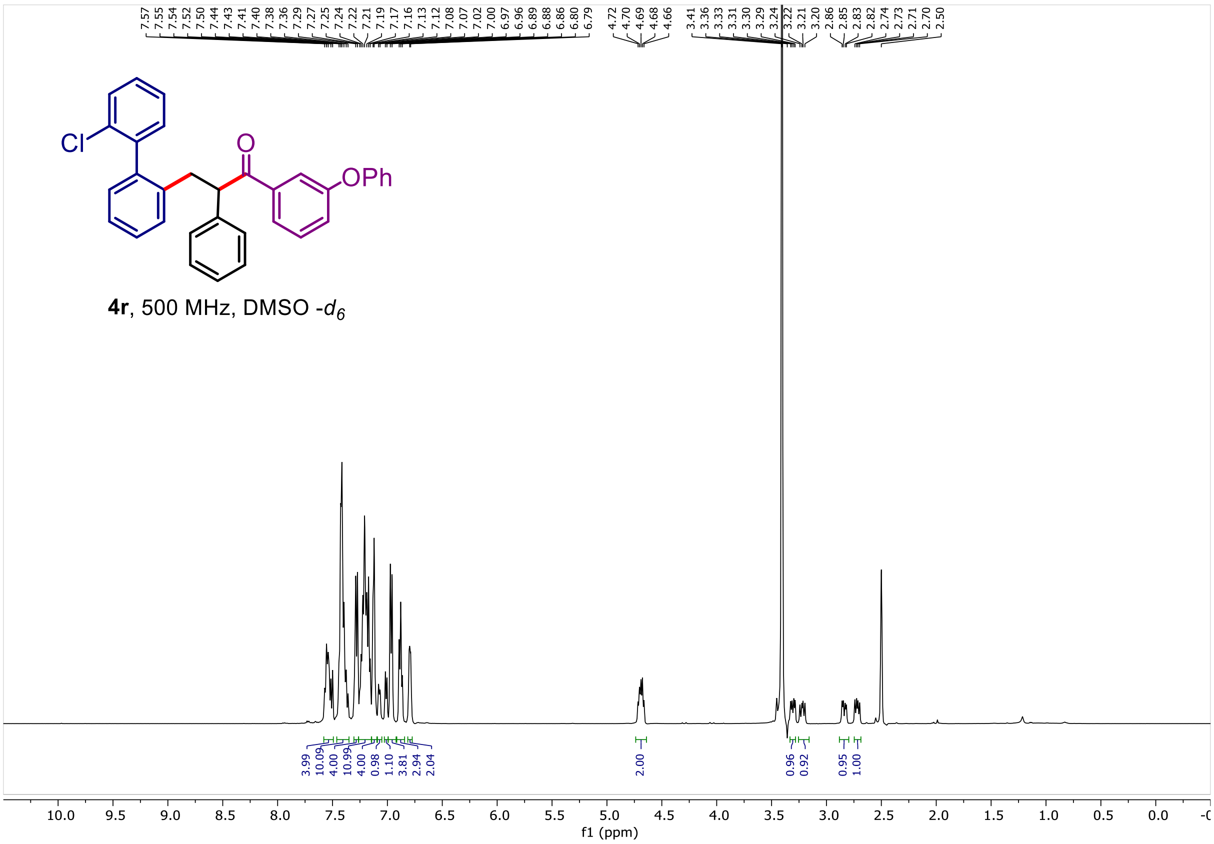

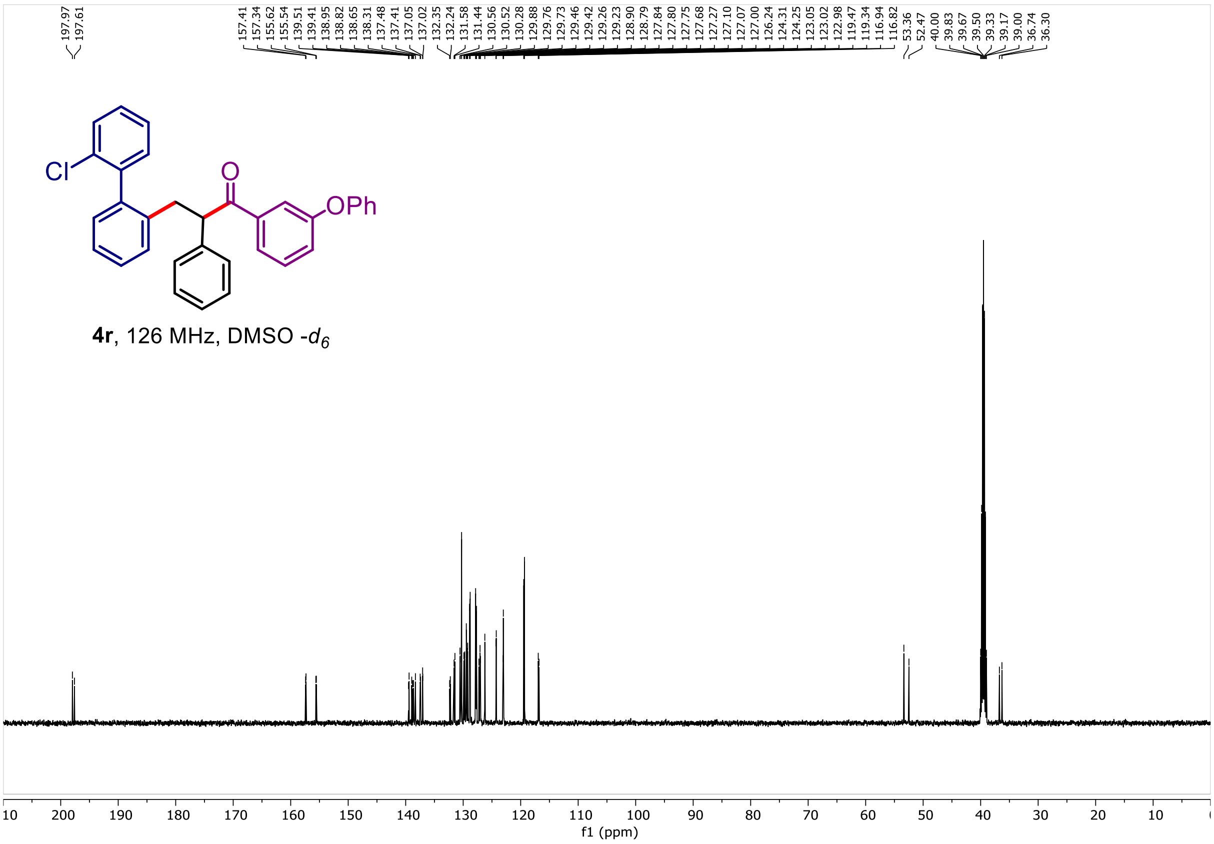

3-(2'-chloro-[1,1'-biphenyl]-2-yl)-1-(3,5-dimethylphenyl)-2-phenylpropan-1-one (4s)

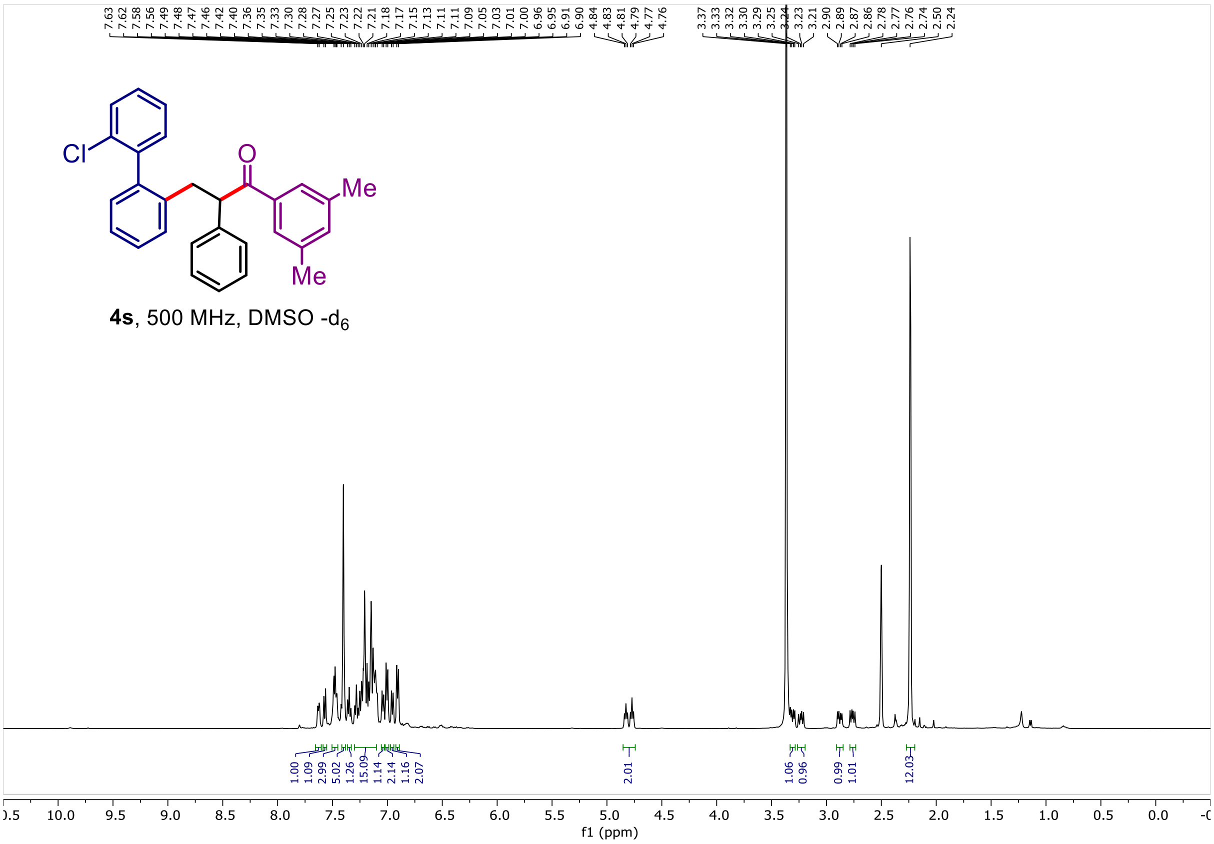

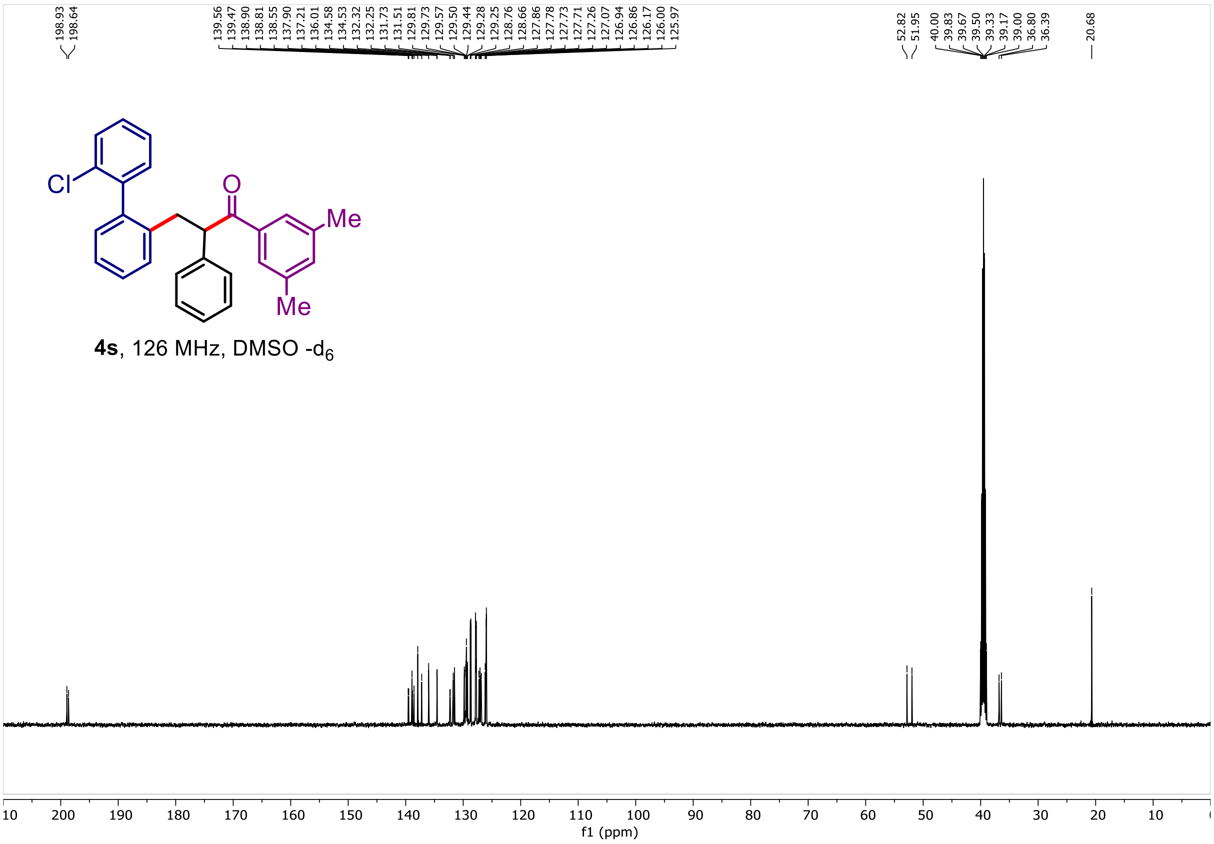

3-(2'-chloro-[1,1'-biphenyl]-2-yl)-1-(3,5-dichlorophenyl)-2-phenylpropan-1-one (4t)

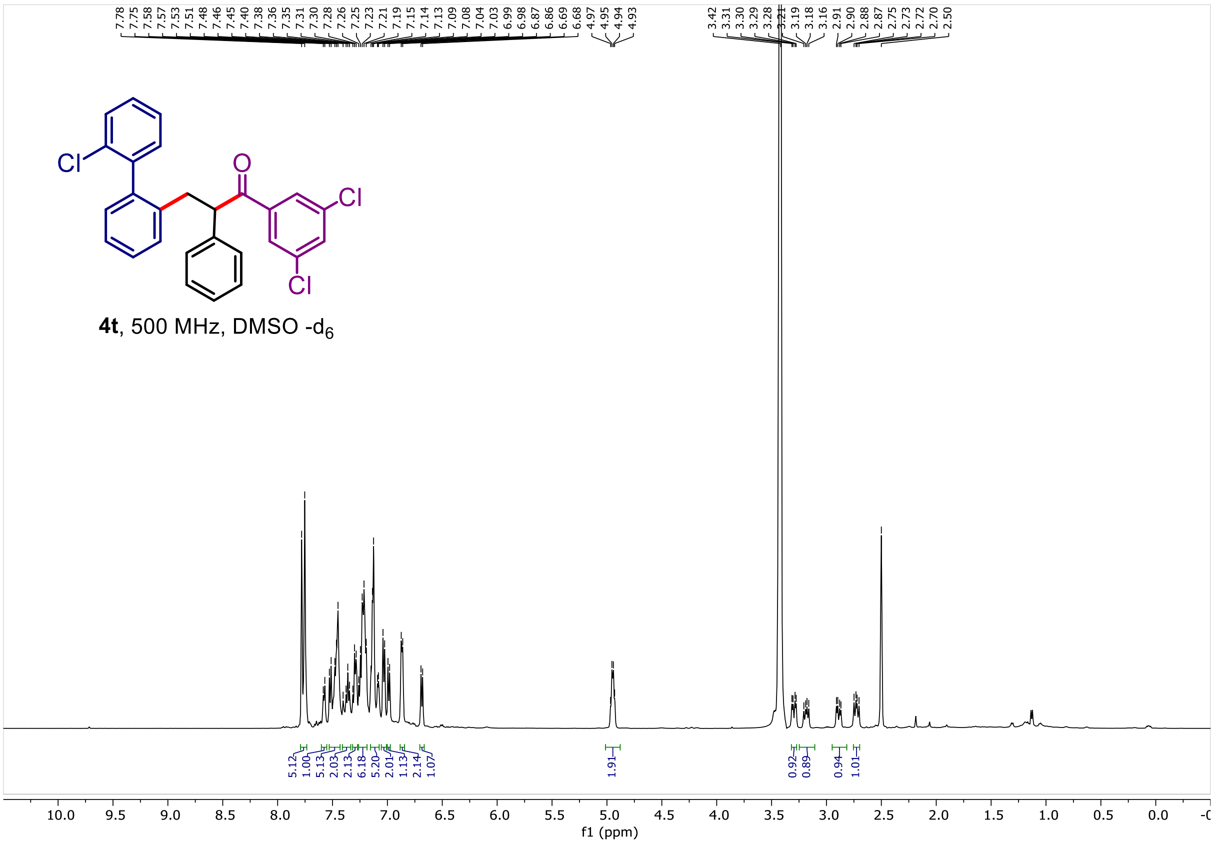

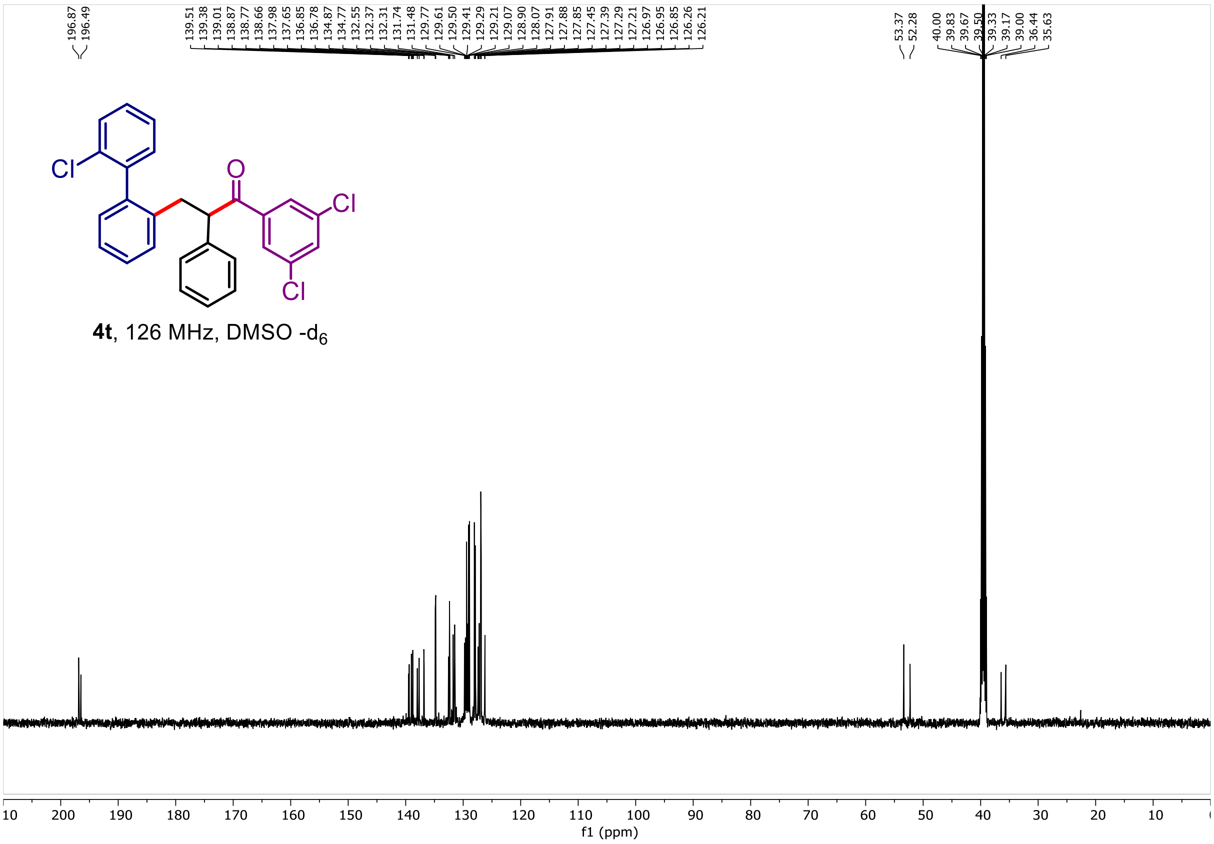

2-(3-(2'-chloro-[1,1'-biphenyl]-2-yl)-2-phenylpropanoyl)benzonitrile (4u)

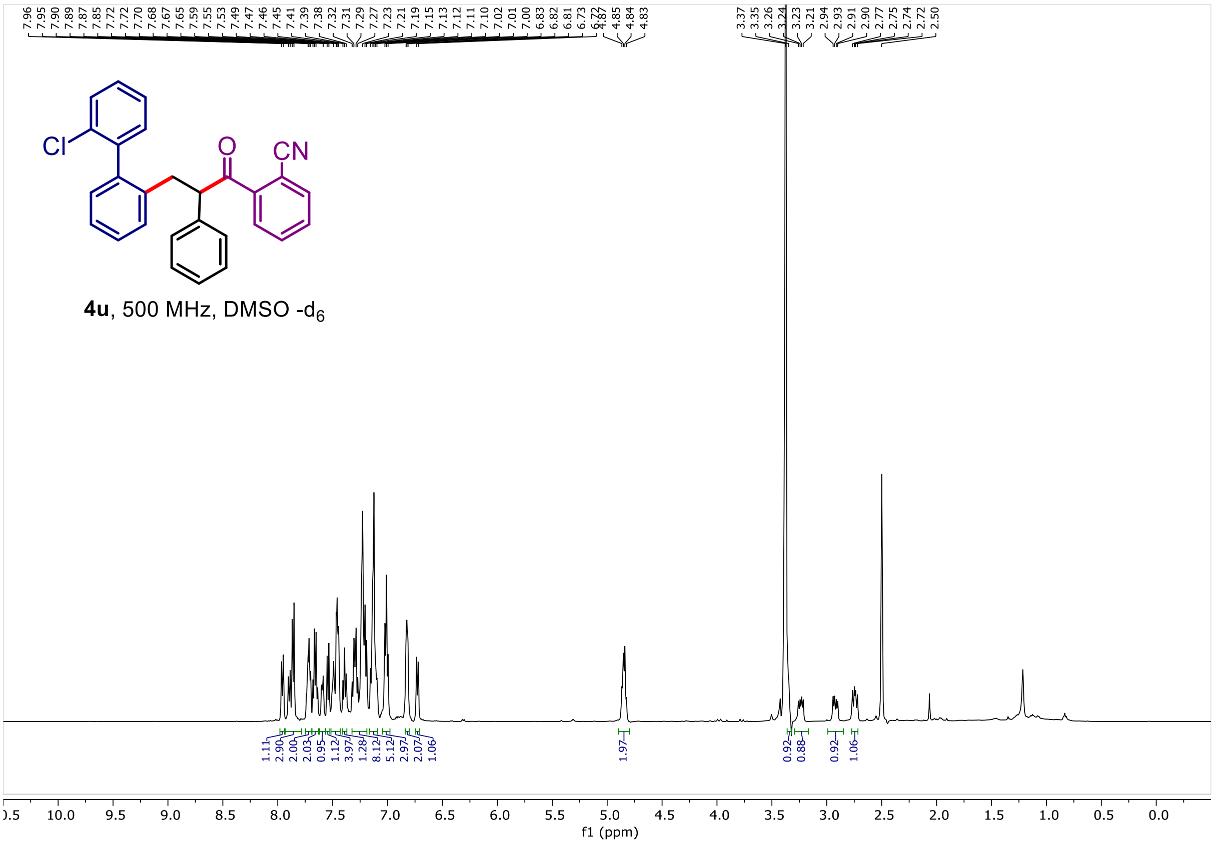

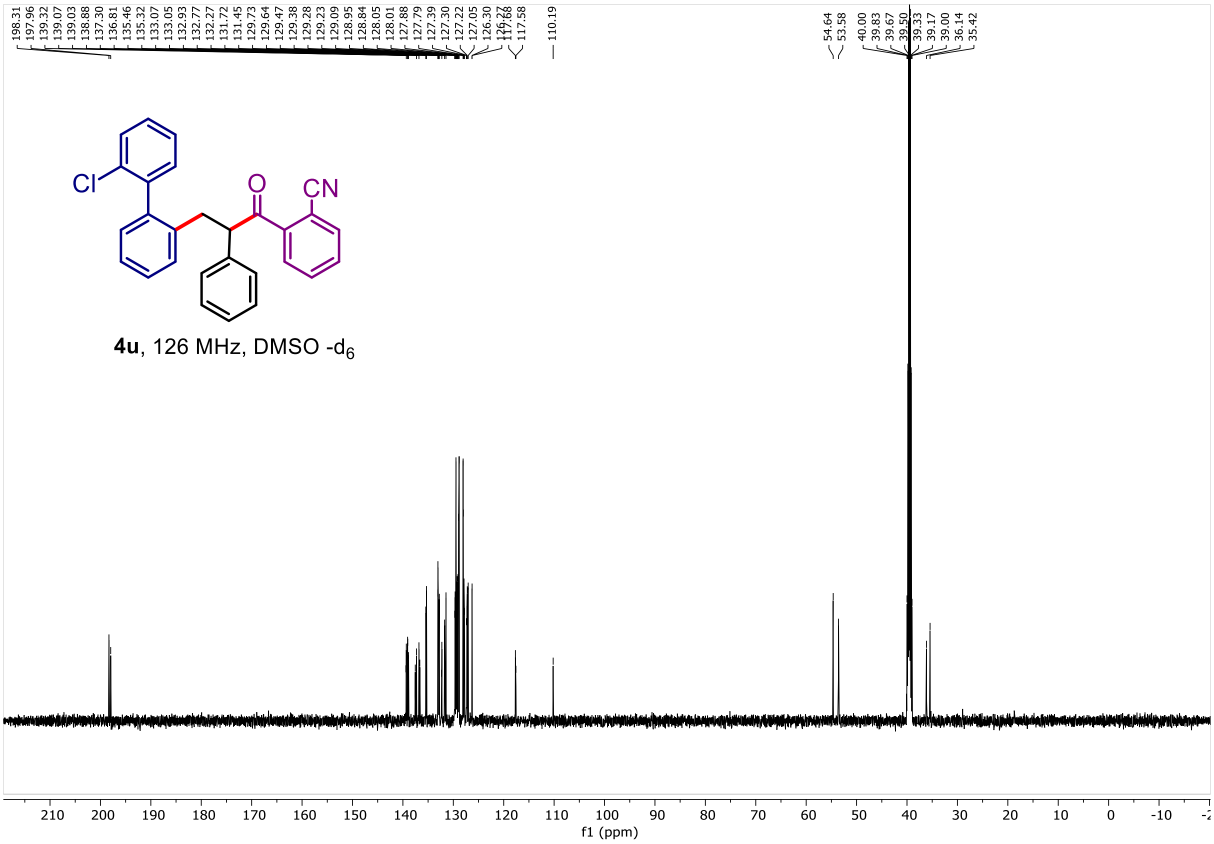

3-(2'-chloro-[1,1'-biphenyl]-2-yl)-1-(naphthalen-2-yl)-2-phenylpropan-1-one (4v)

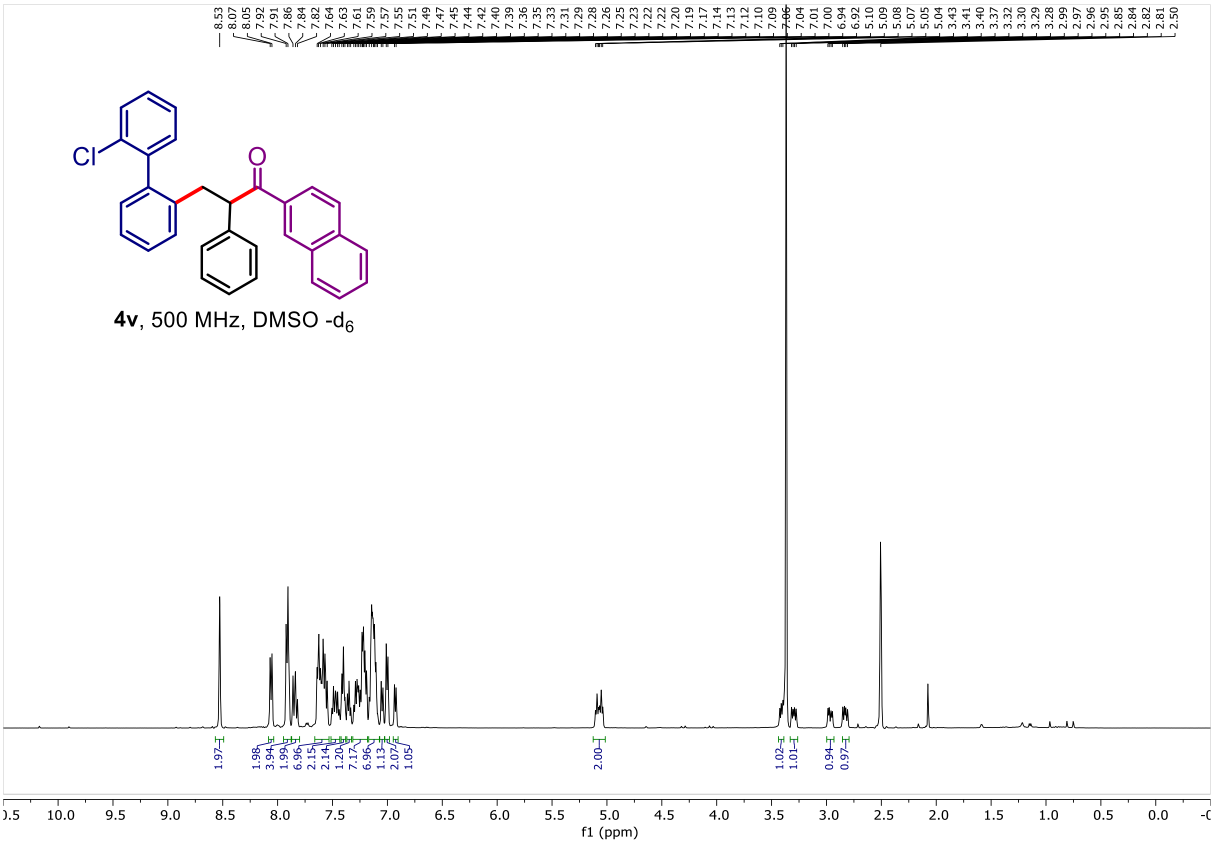

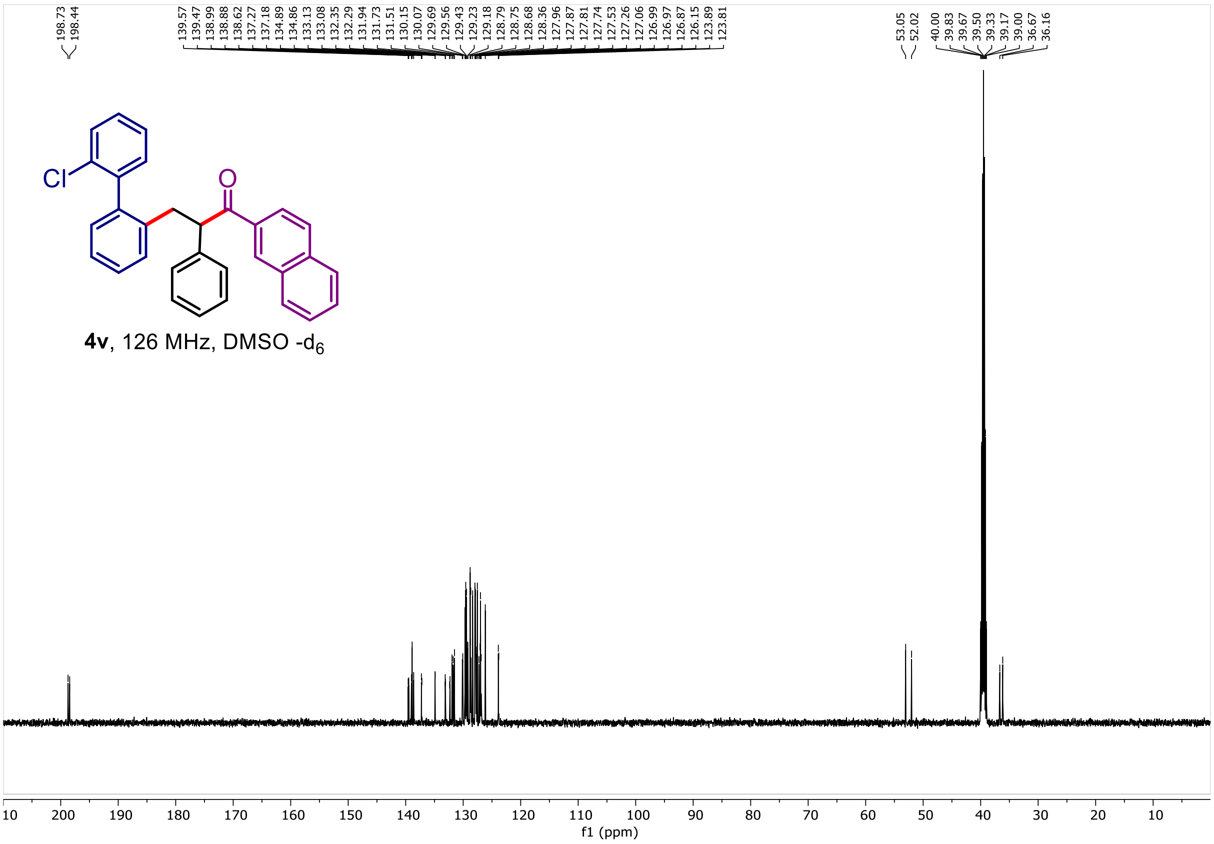

3-(2'-chloro-[1,1'-biphenyl]-2-yl)-1-(furan-2-yl)-2-phenylpropan-1-one (4w)

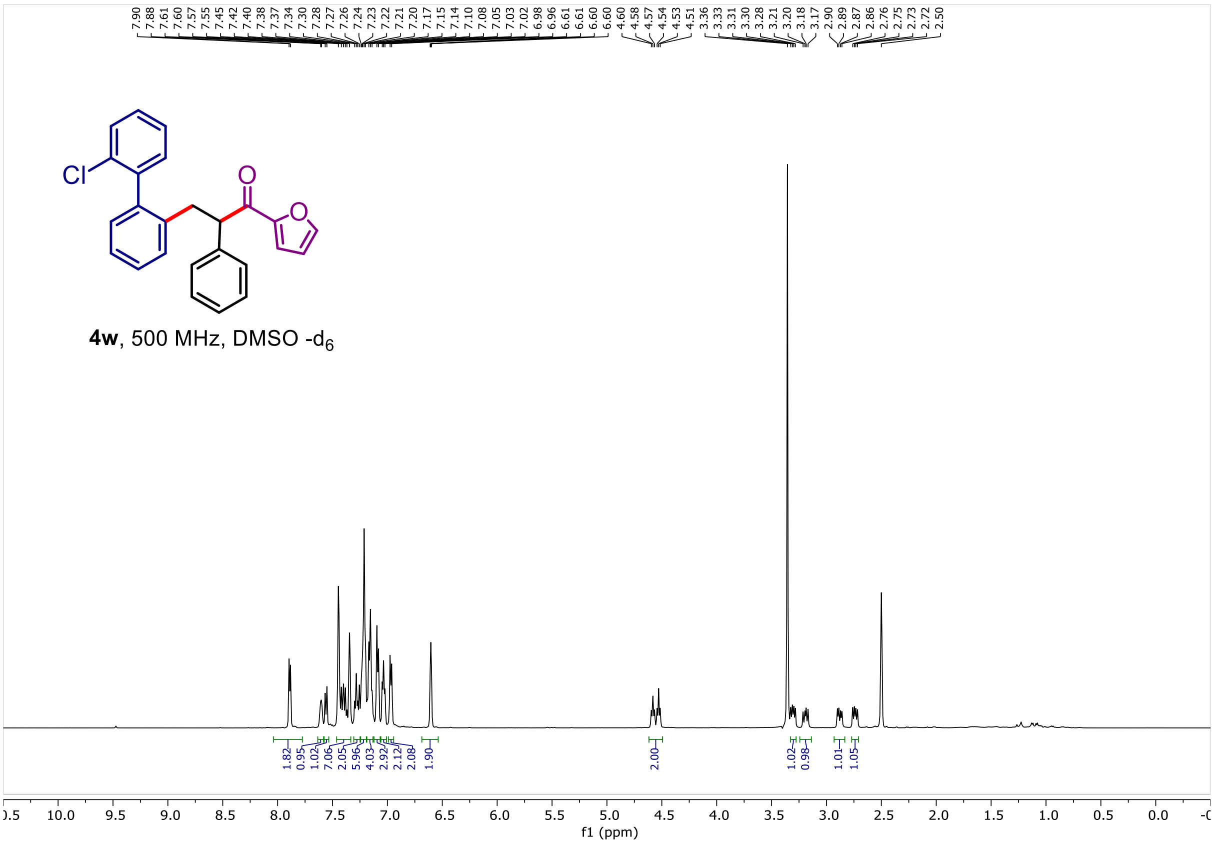

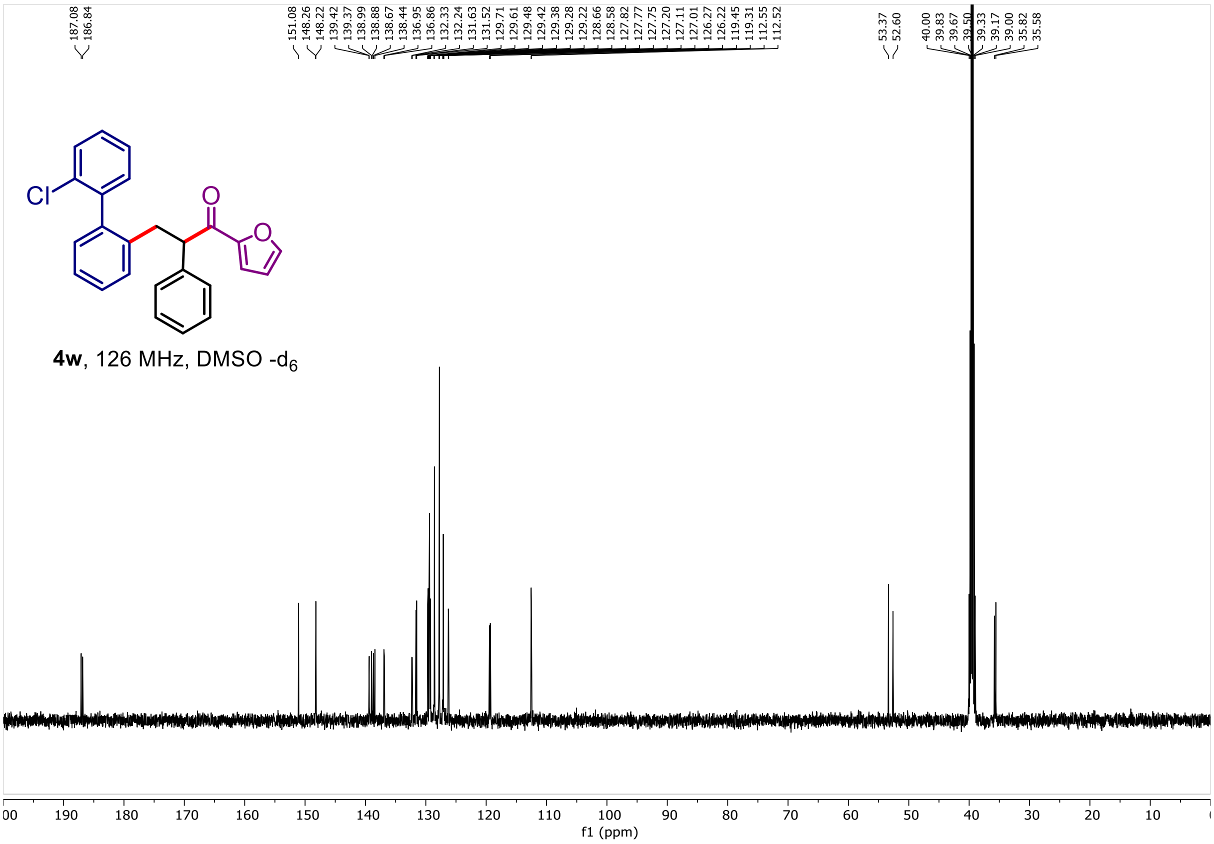

3-(2'-chloro-[1,1'-biphenyl]-2-yl)-2-phenyl-1-(thiophen-2-yl)propan-1-one (4x)

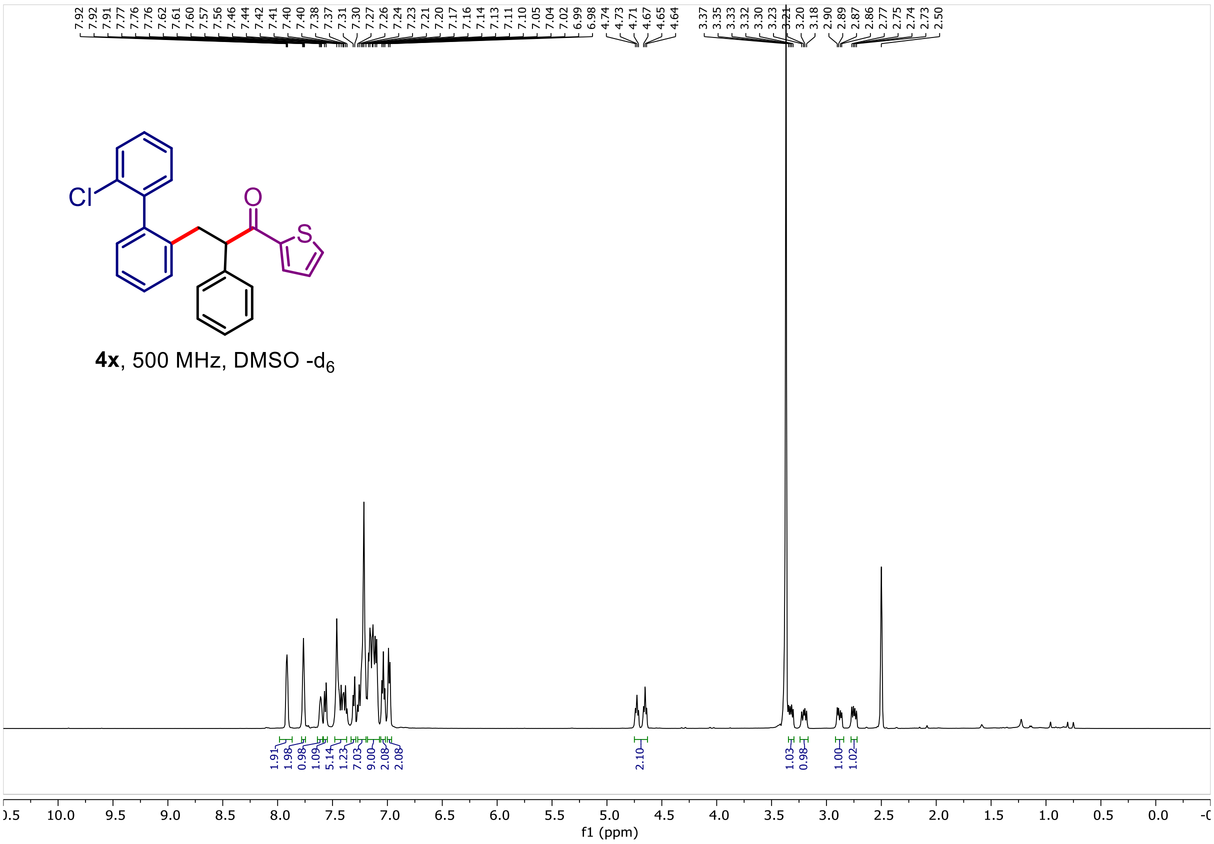

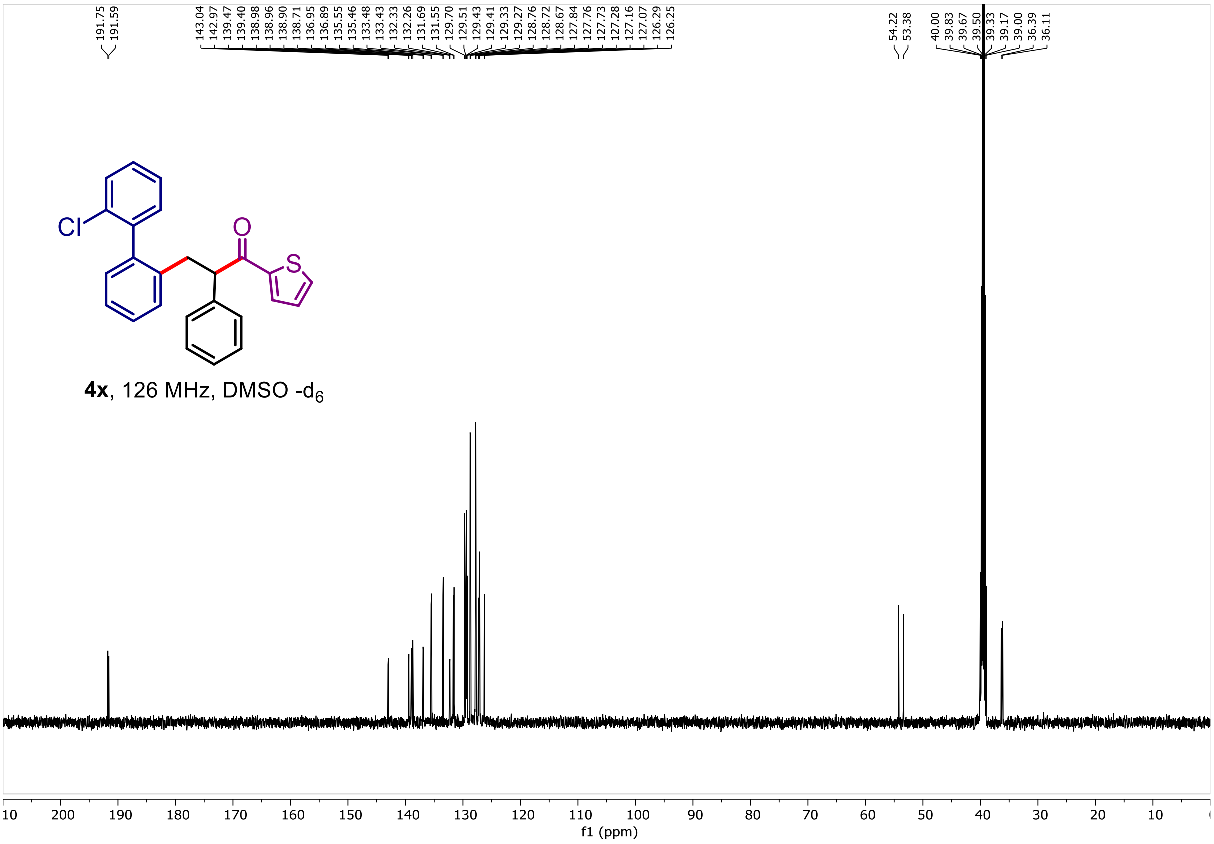

3-(2'-chloro-[1,1'-biphenyl]-2-yl)-2-phenyl-1-(pyridin-2-yl)propan-1-one (4y)

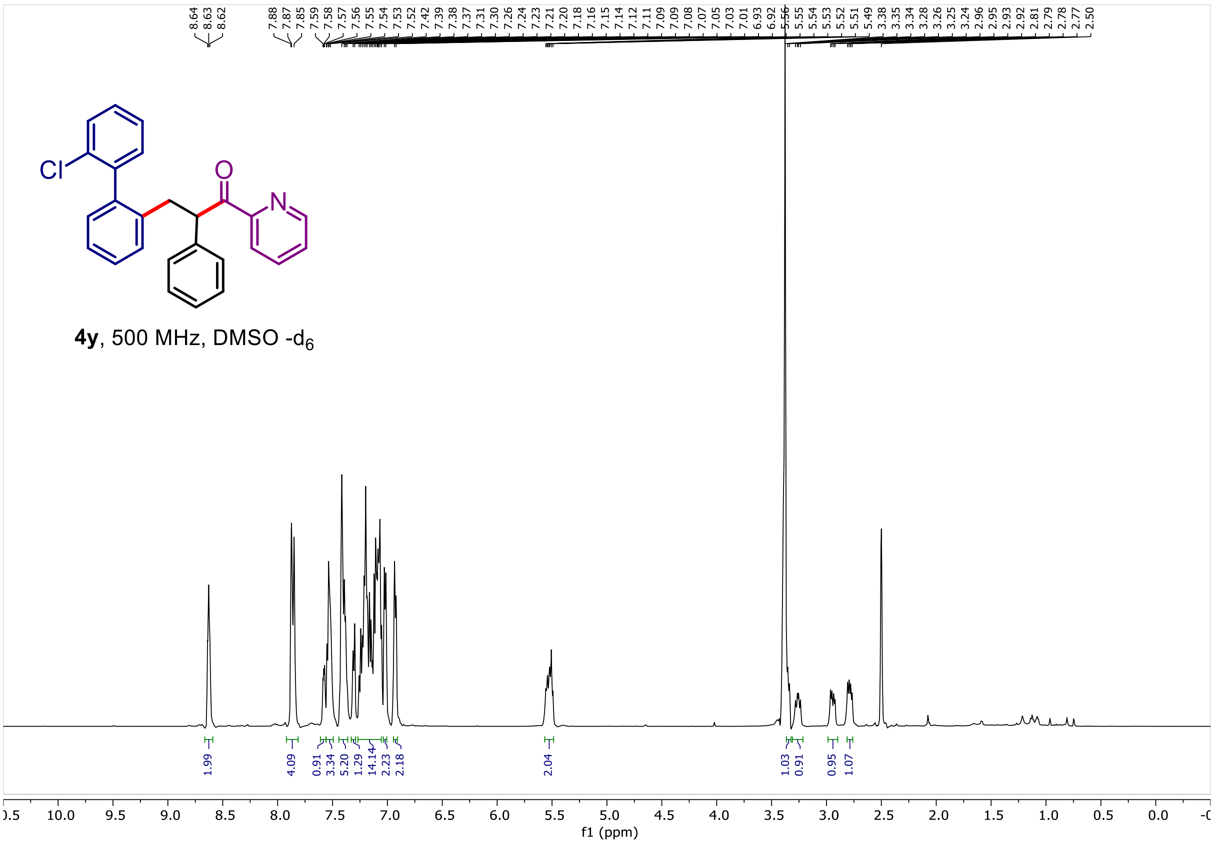

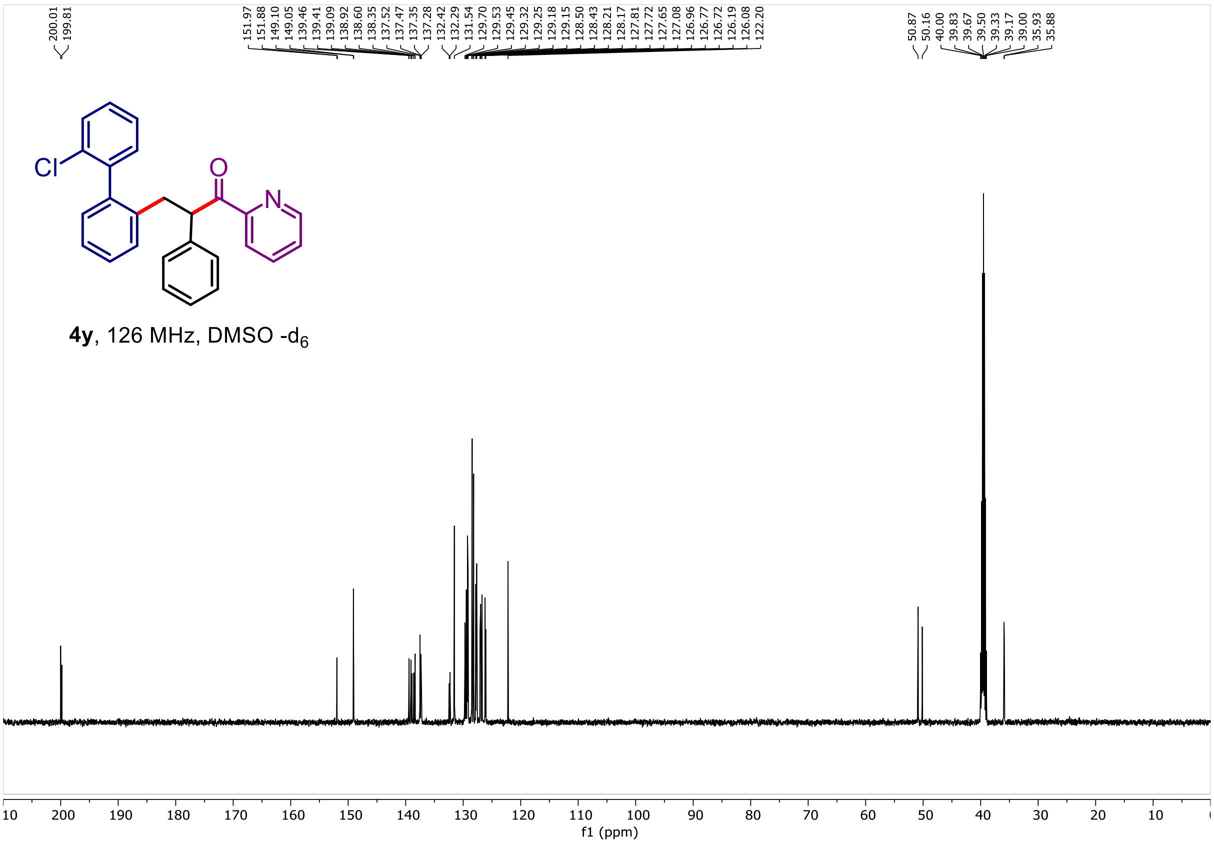

3-(2'-chloro-[1,1'-biphenyl]-2-yl)-2-phenyl-1-(quinolin-2-yl)propan-1-one (4z)

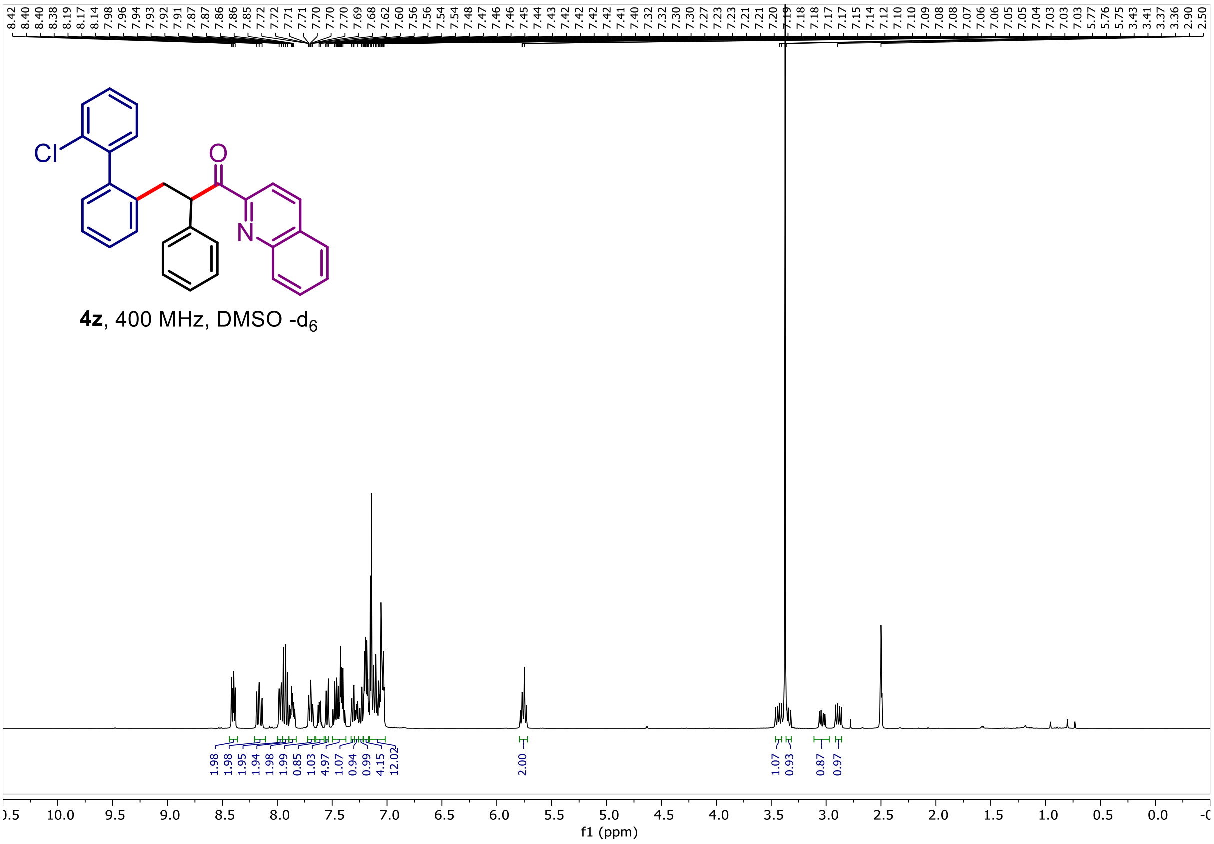

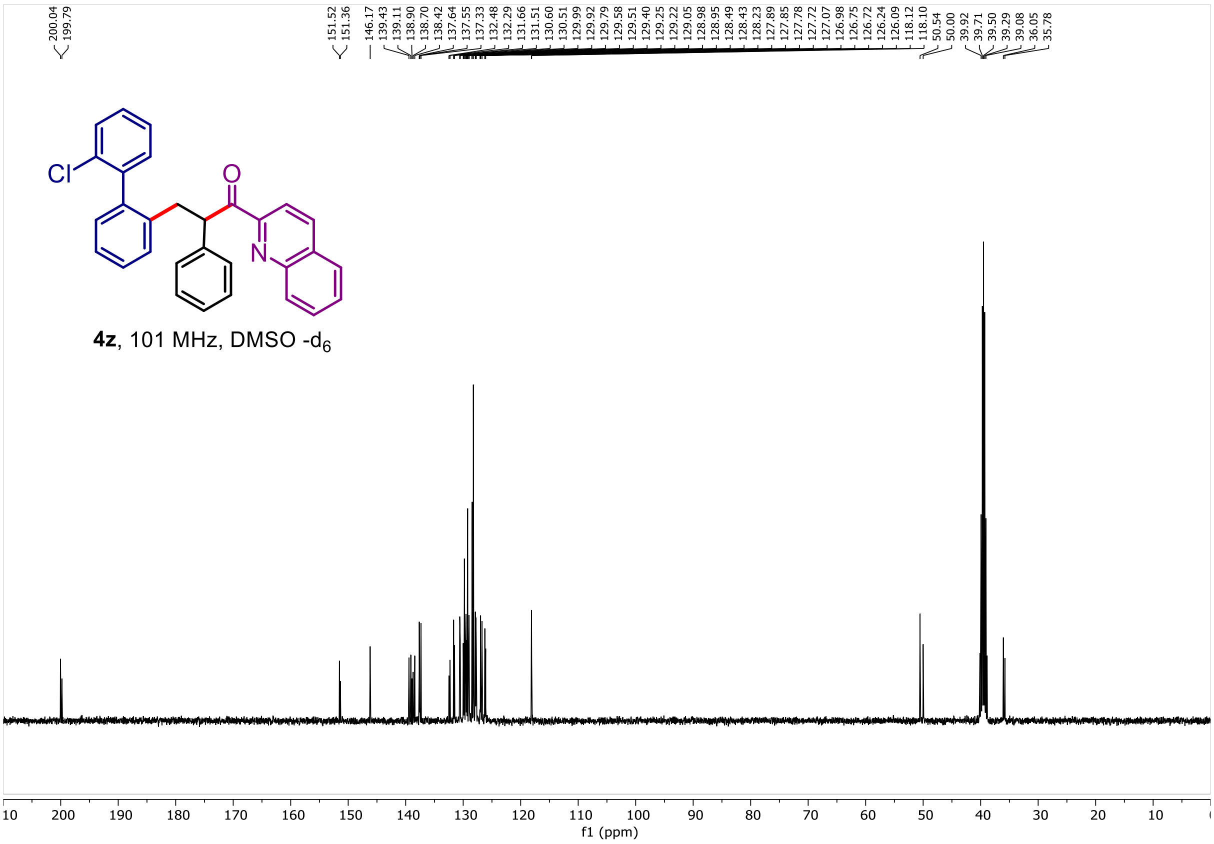

1-(4-bromophenyl)-2-(4-(tert-butyl)phenyl)-3-(2'-chloro-[1,1'-biphenyl]-2-yl)propan-1-one (5a)

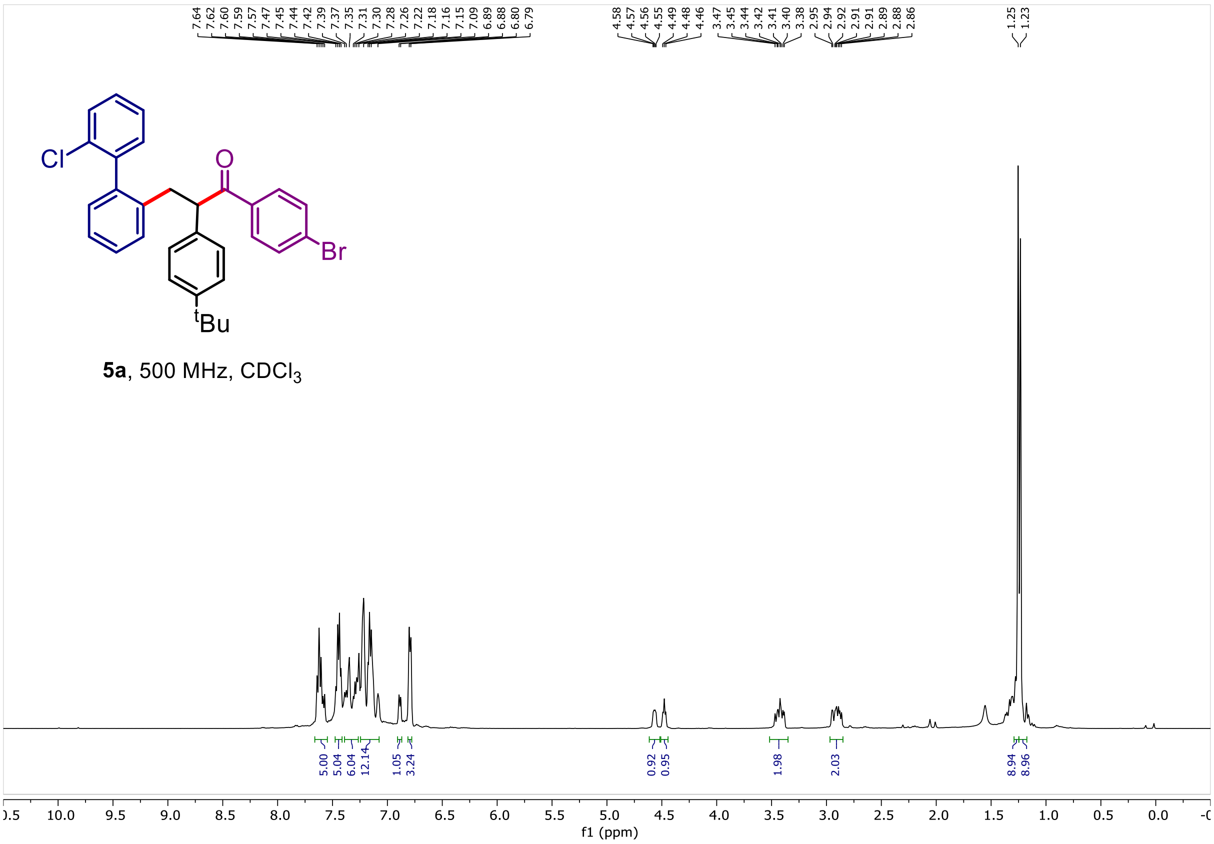

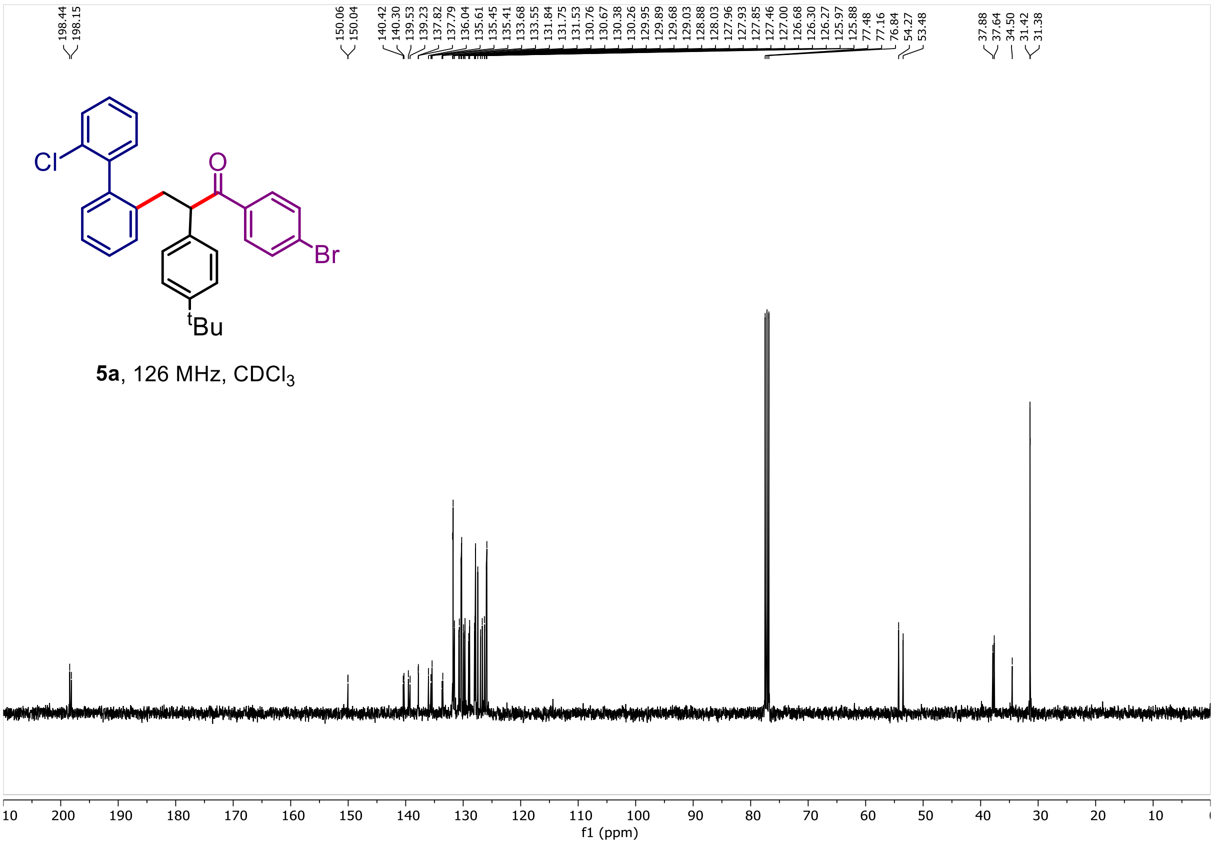

1-(4-bromophenyl)-3-(2'-chloro-[1,1'-biphenyl]-2-yl)-2-(p-tolyl)propan-1-one (5b)

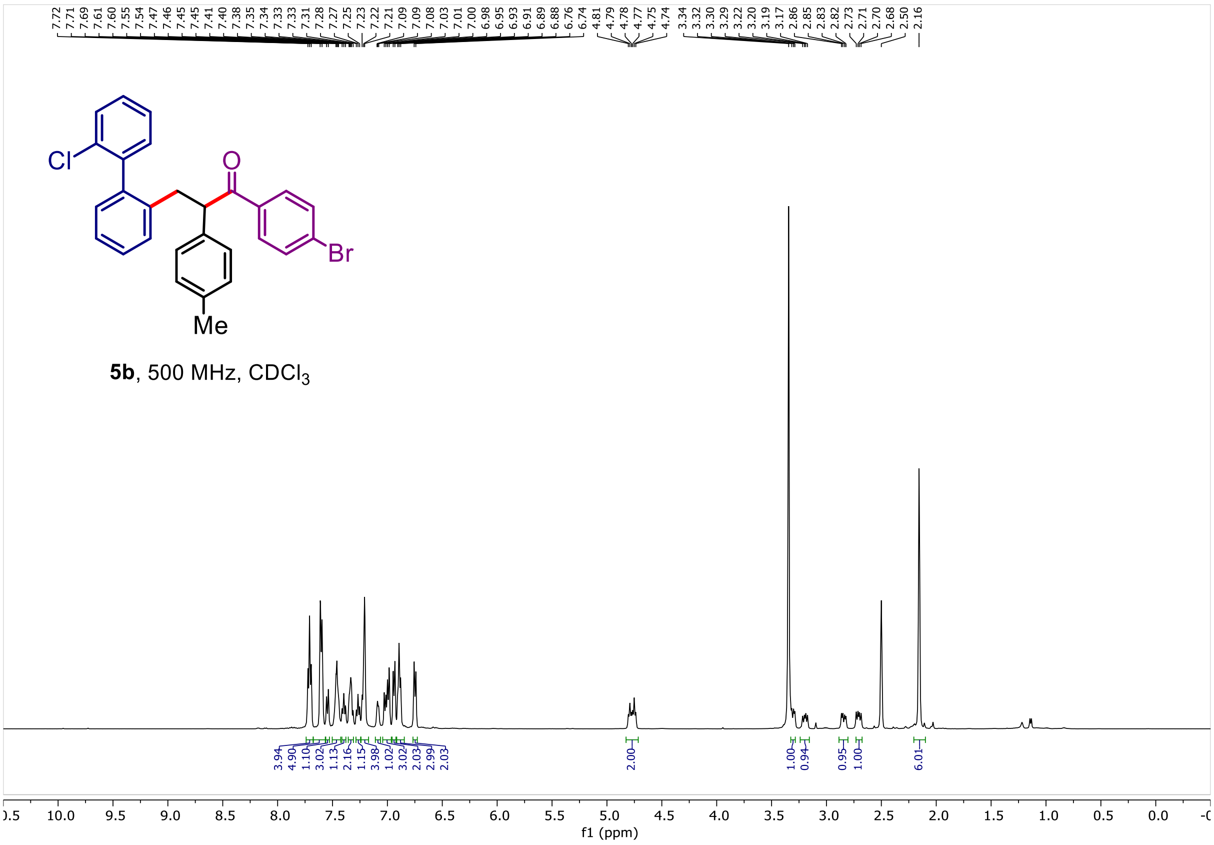

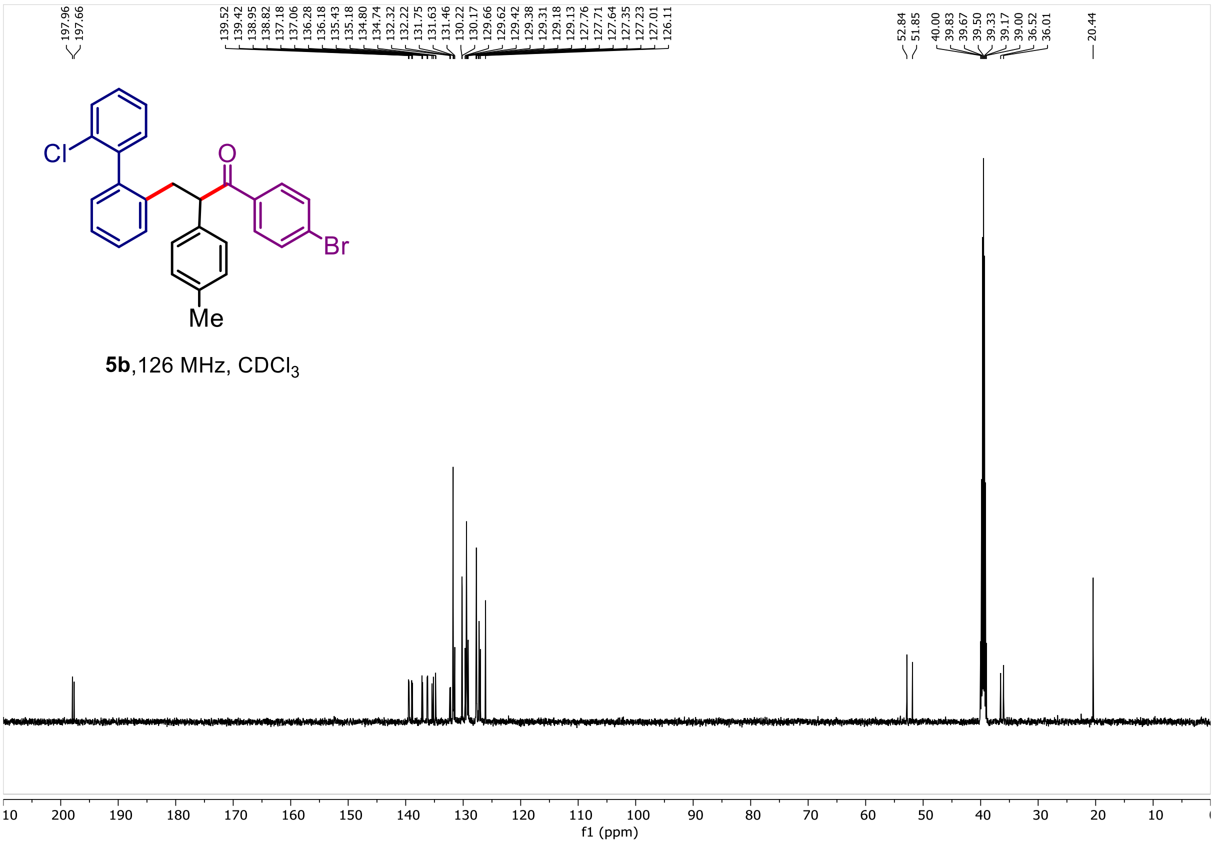

1-(4-bromophenyl)-3-(2'-chloro-[1,1'-biphenyl]-2-yl)-2-(4-methoxyphenyl)propan-1-one(5c)

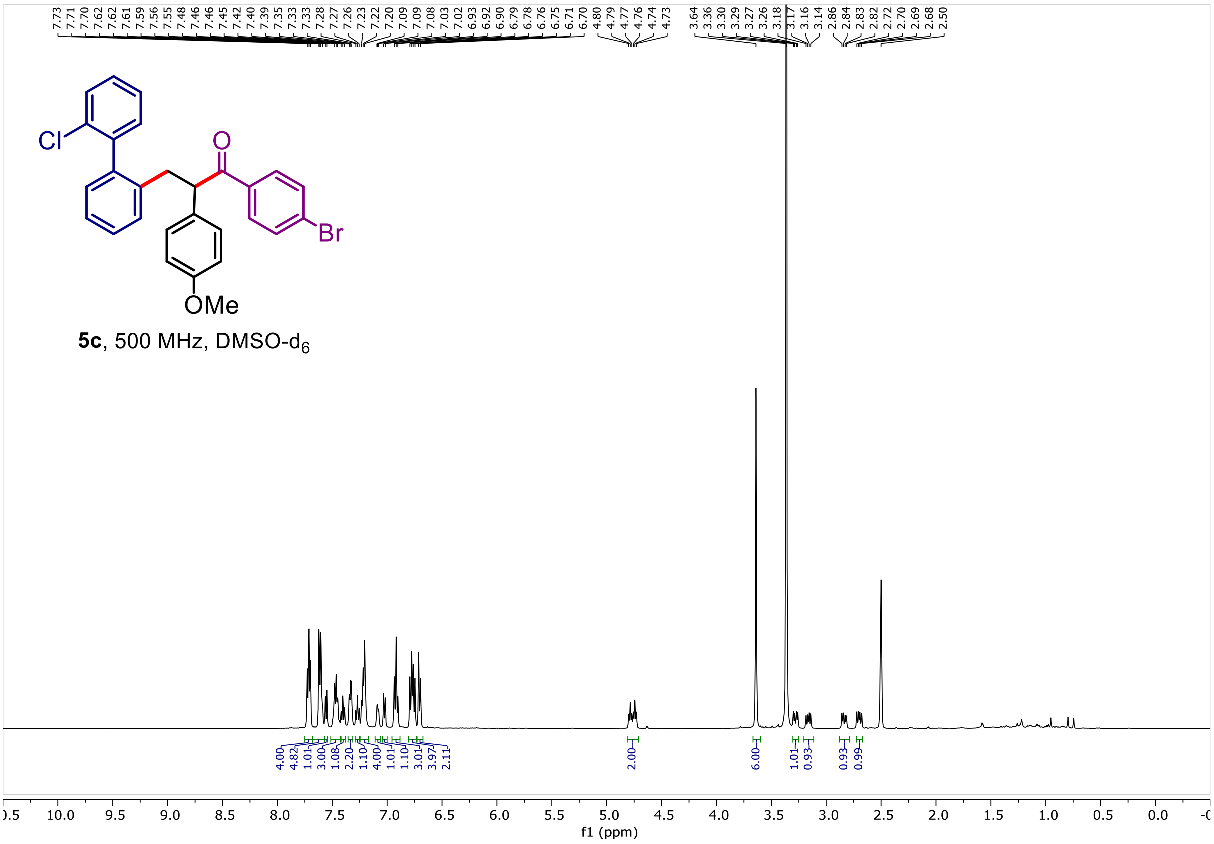

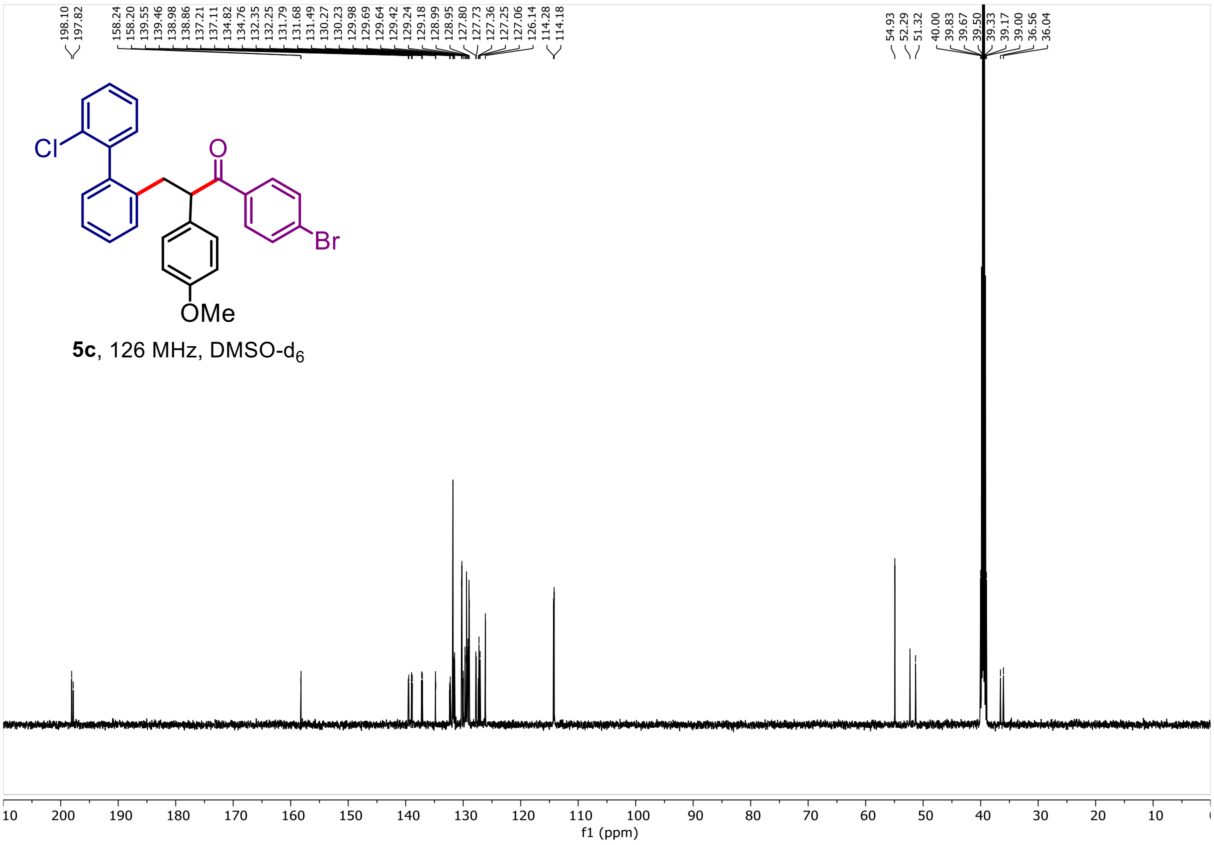

1-(4-bromophenyl)-3-(2'-chloro-[1,1'-biphenyl]-2-yl)-2-(4-chlorophenyl)propan-1-one(5d)

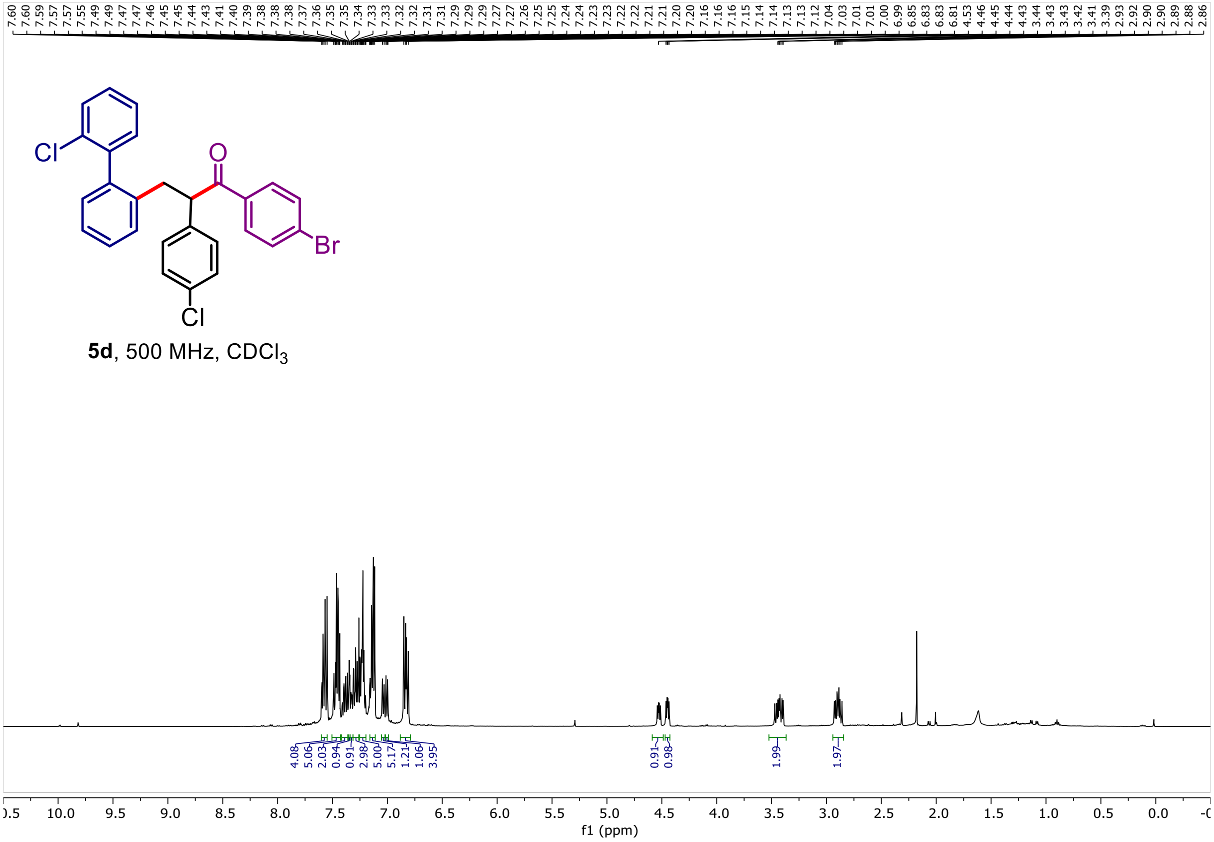

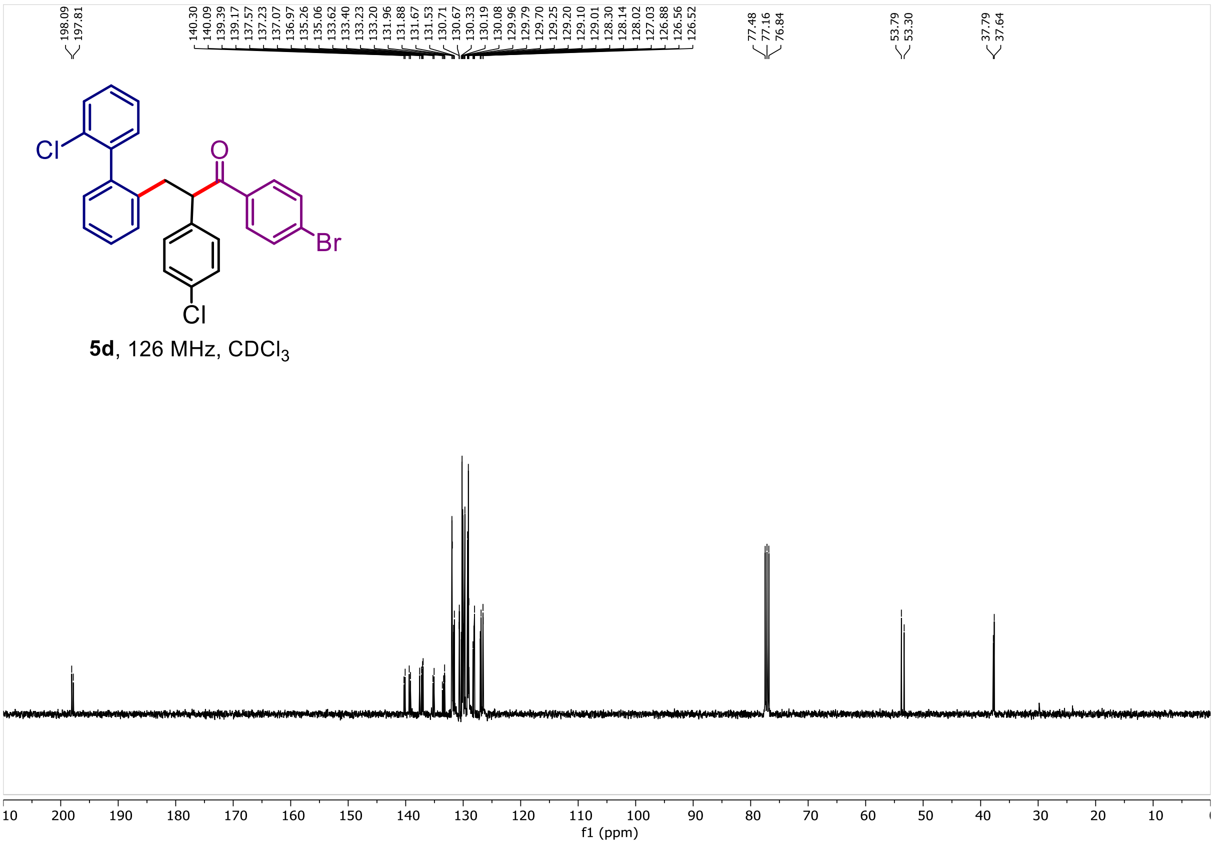

1-(4-bromophenyl)-3-(2'-chloro-[1,1'-biphenyl]-2-yl)-2-(*m*-tolyl)propan-1-one (5e)

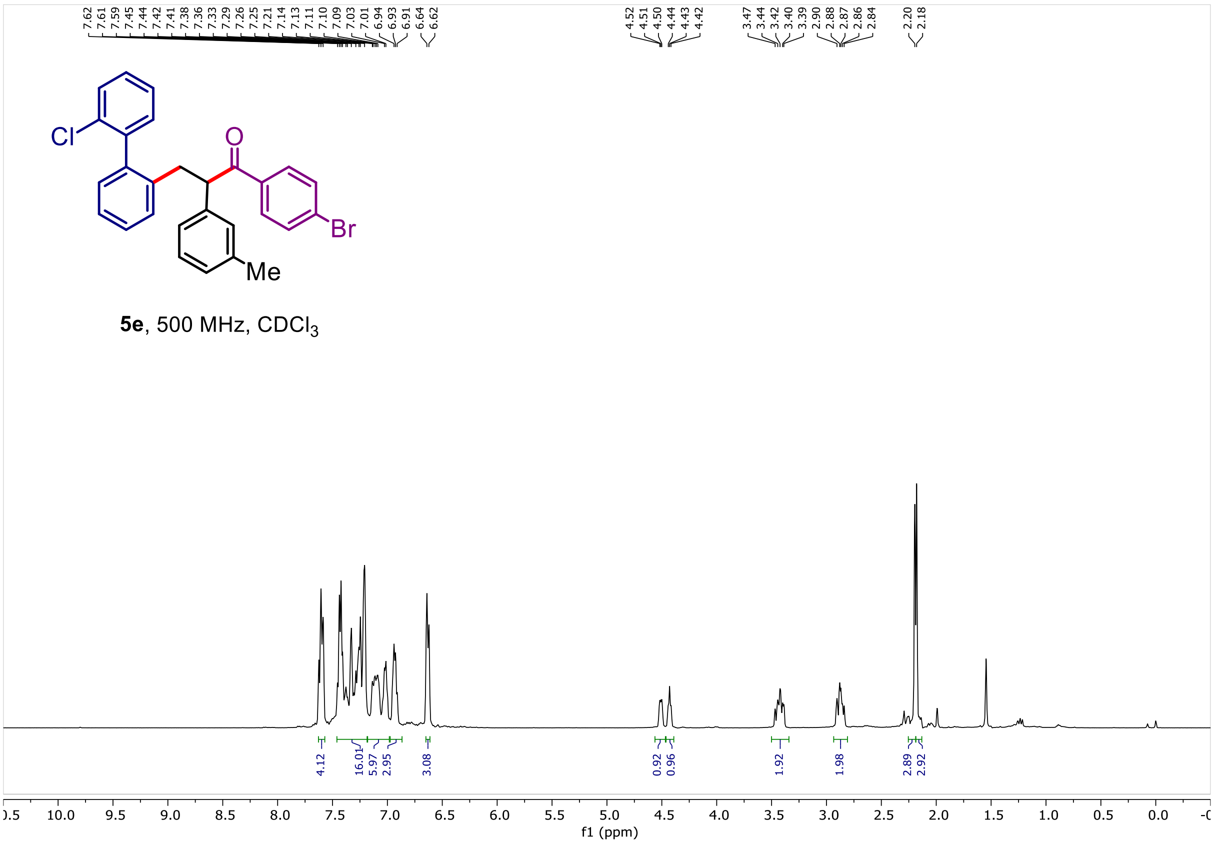

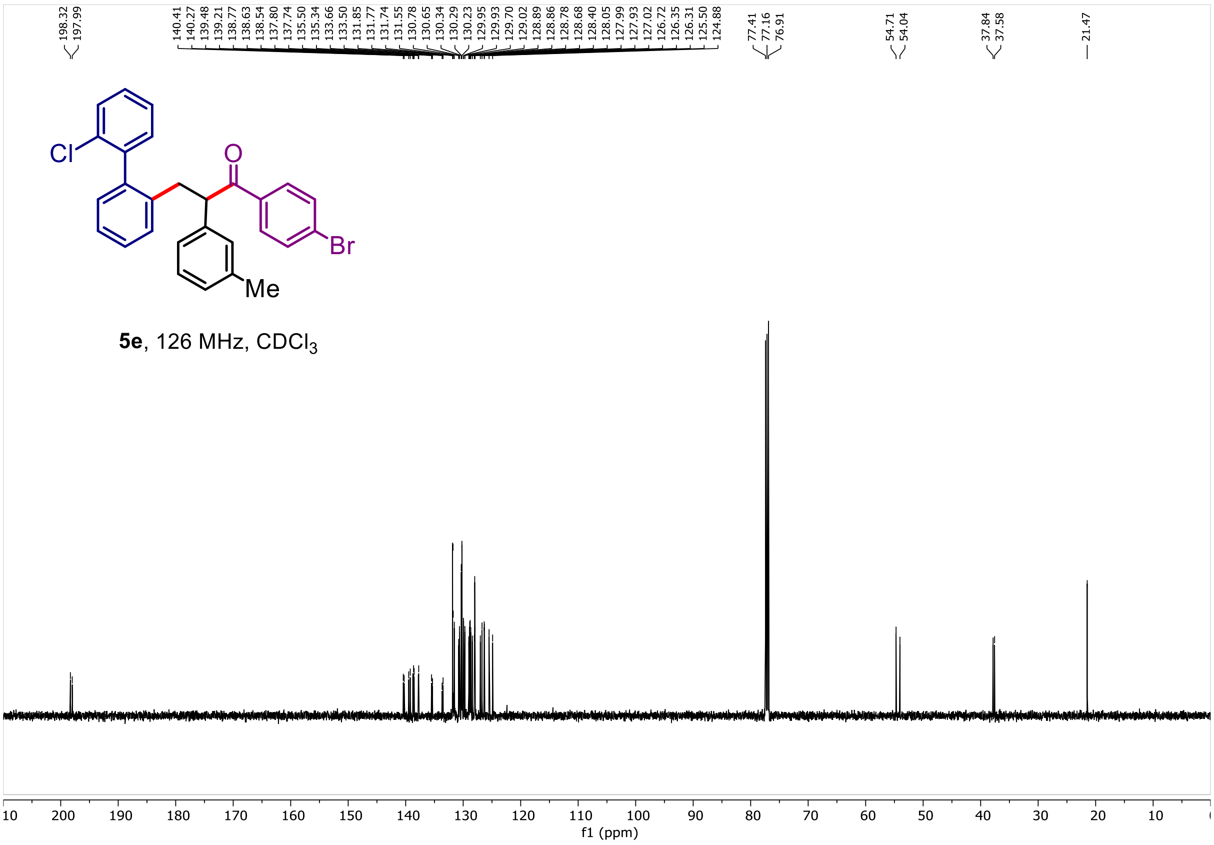

1-(4-bromophenyl)-3-(2'-chloro-[1,1'-biphenyl]-2-yl)-2-(3-phenoxyphenyl)propan-1-one (5f)

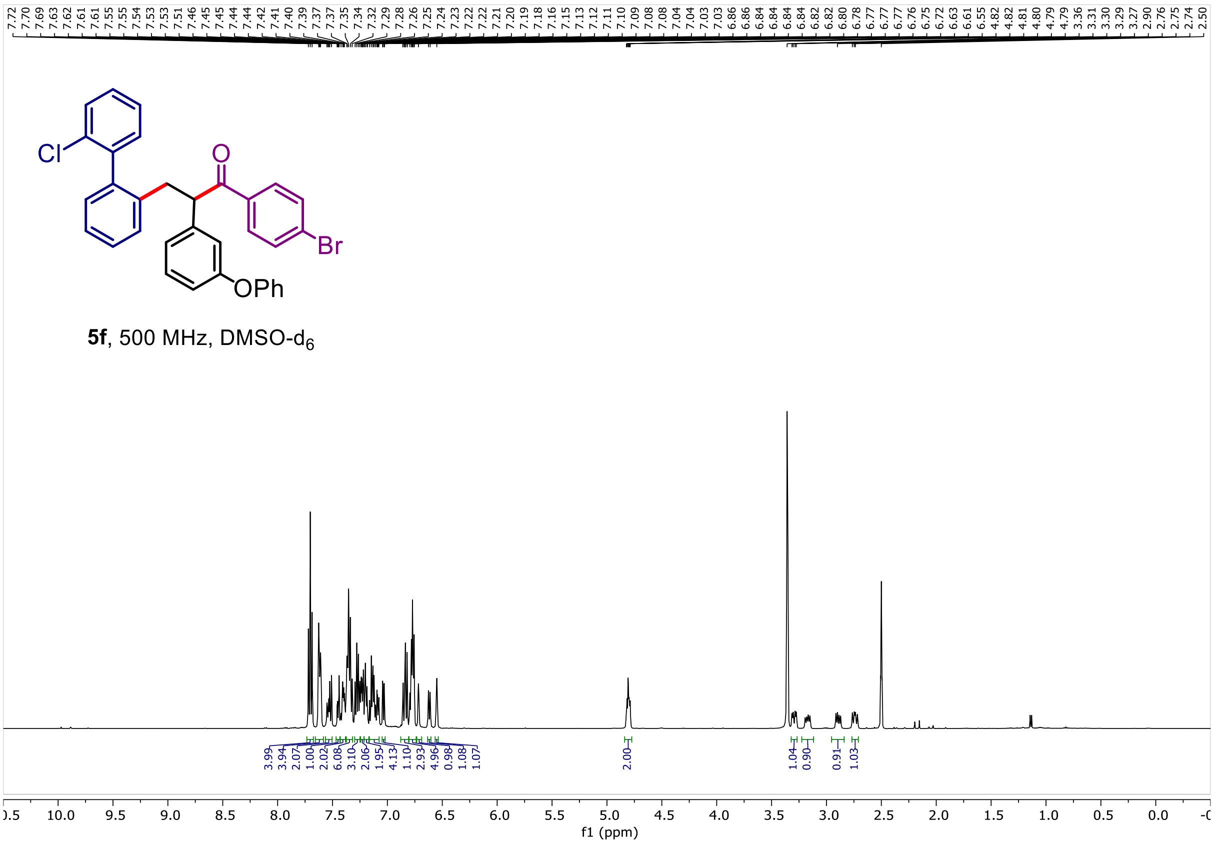

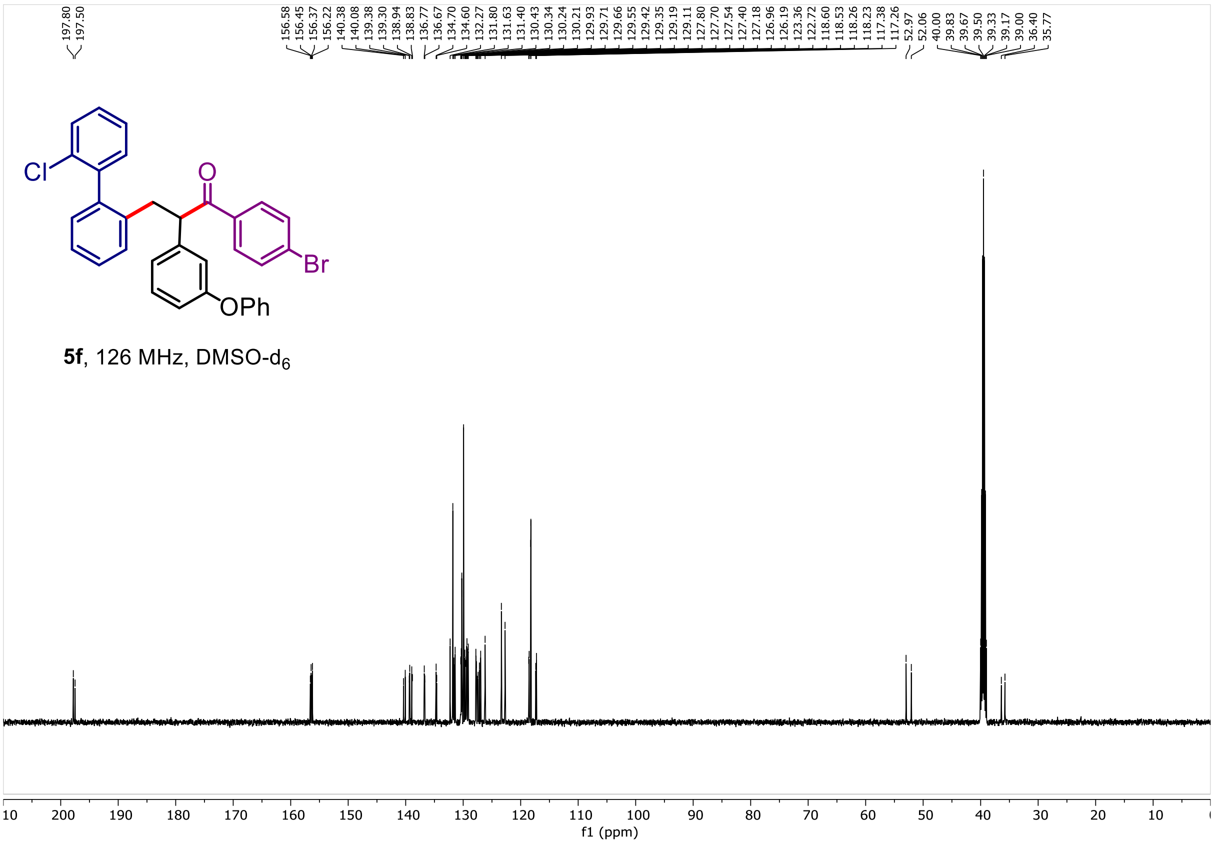

1-(4-bromophenyl)-3-(2'-chloro-[1,1'-biphenyl]-2-yl)-2-(o-tolyl)propan-1-one (5g)

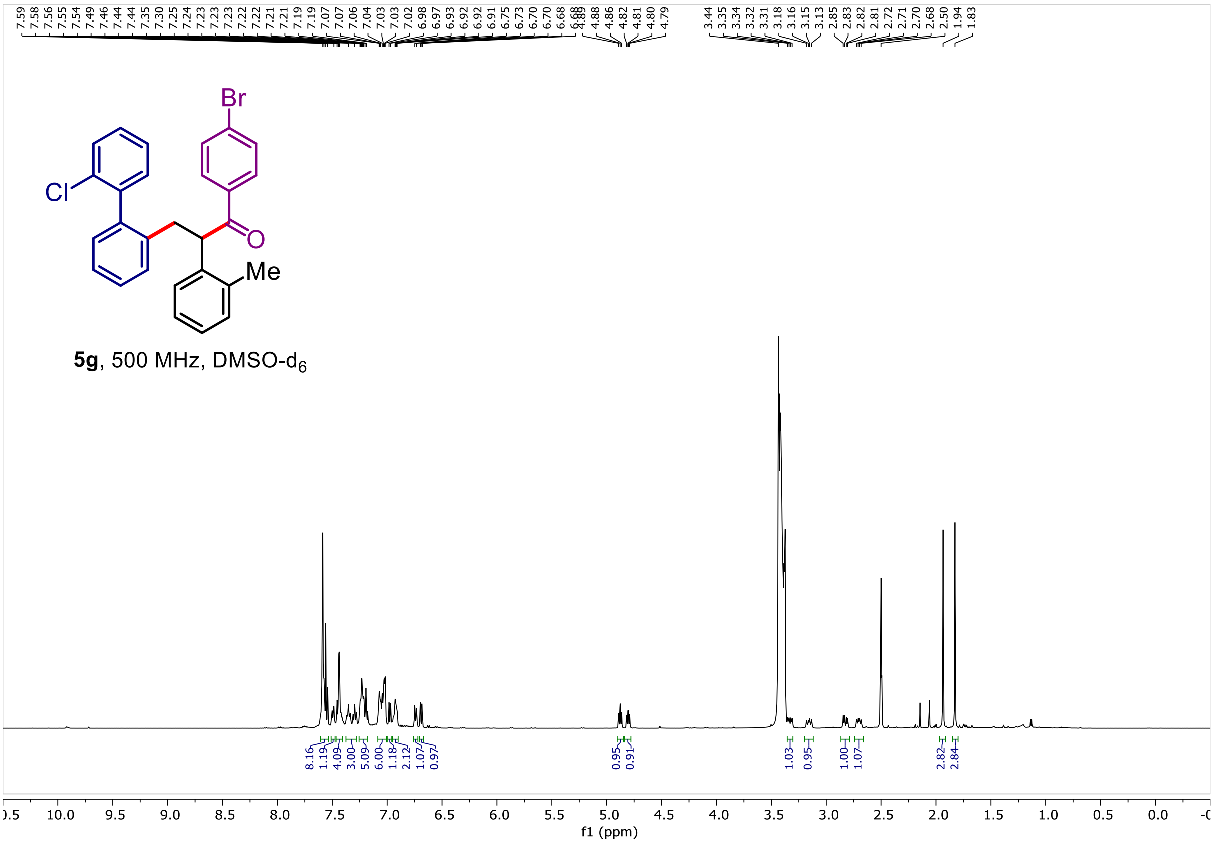

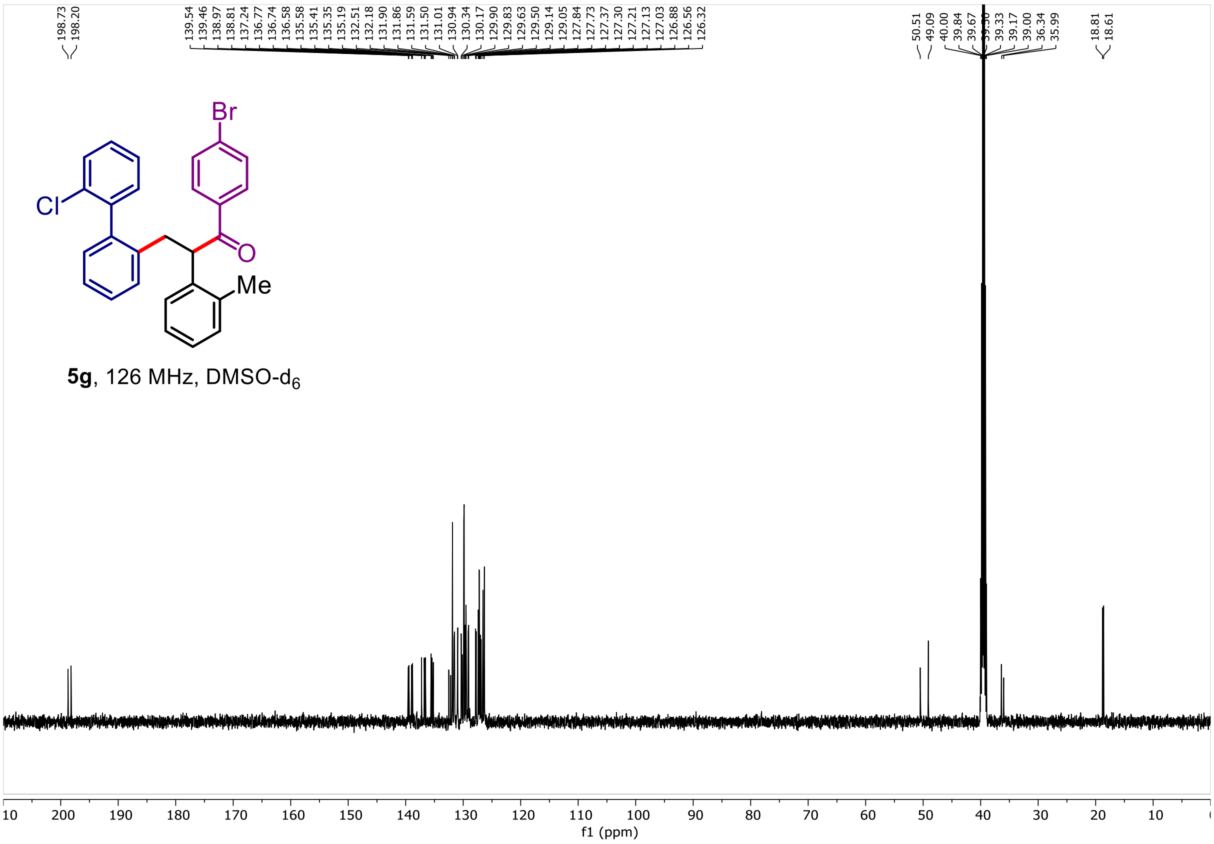

1-(4-bromophenyl)-3-(2'-chloro-[1,1'-biphenyl]-2-yl)-2-(3,5-dimethylphenyl)propan-1-one (5h)

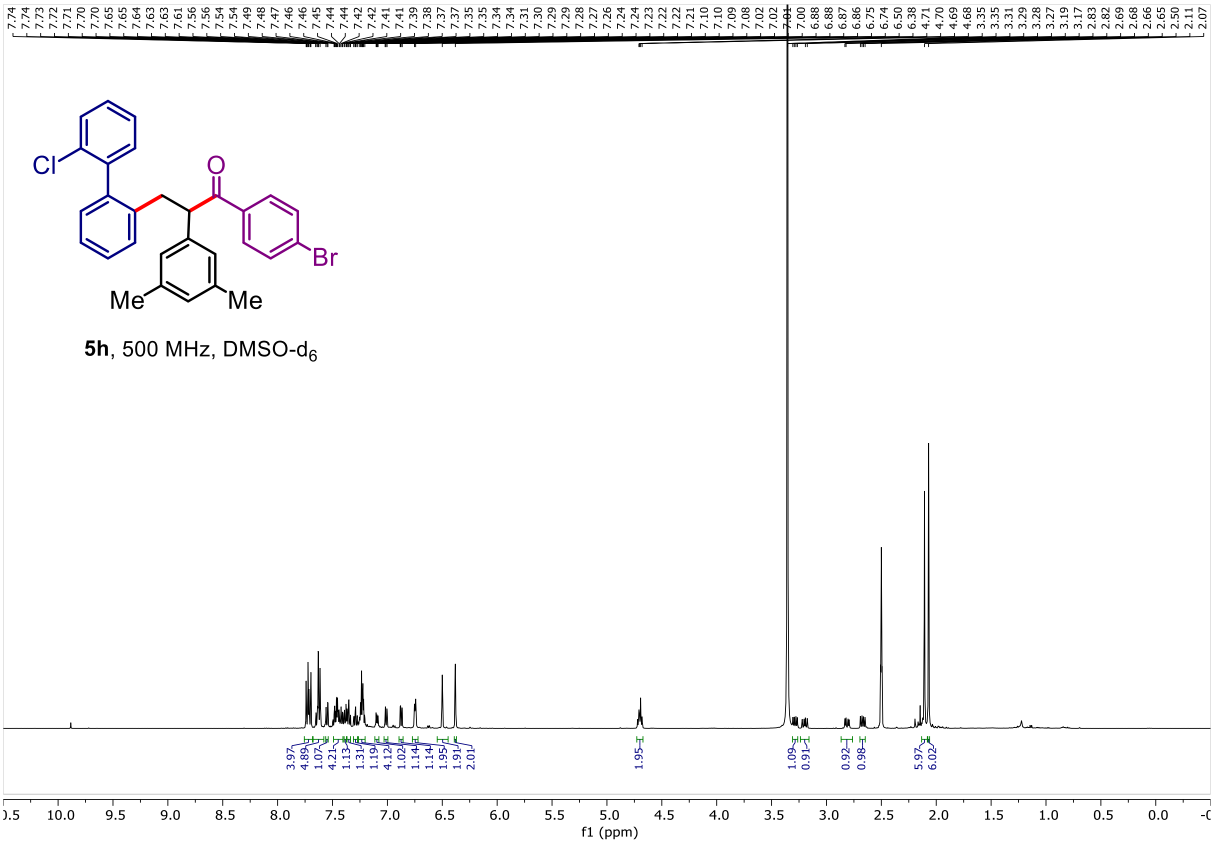

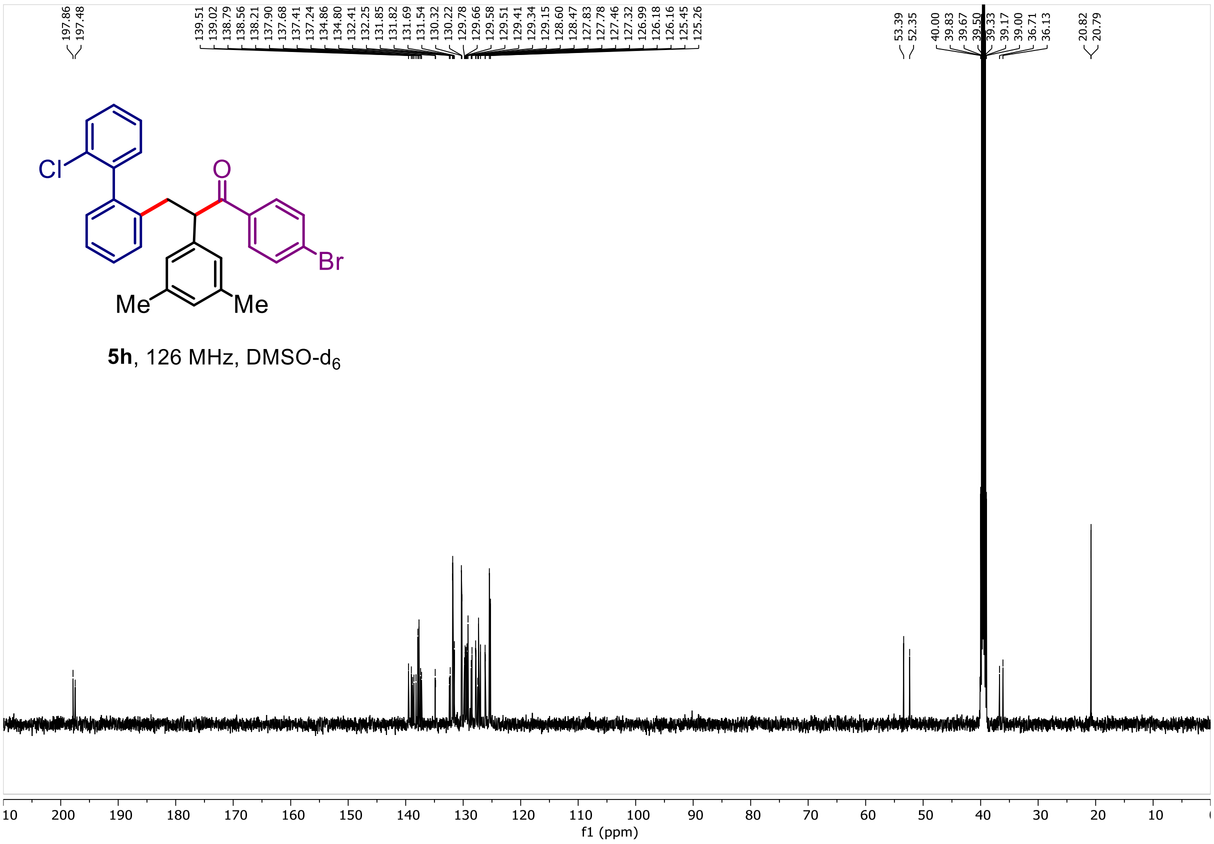

4-(1-(4-bromophenyl)-3-(2'-chloro-[1,1'-biphenyl]-2-yl)-1-oxopropan-2-yl)phenyl acetate (5i)

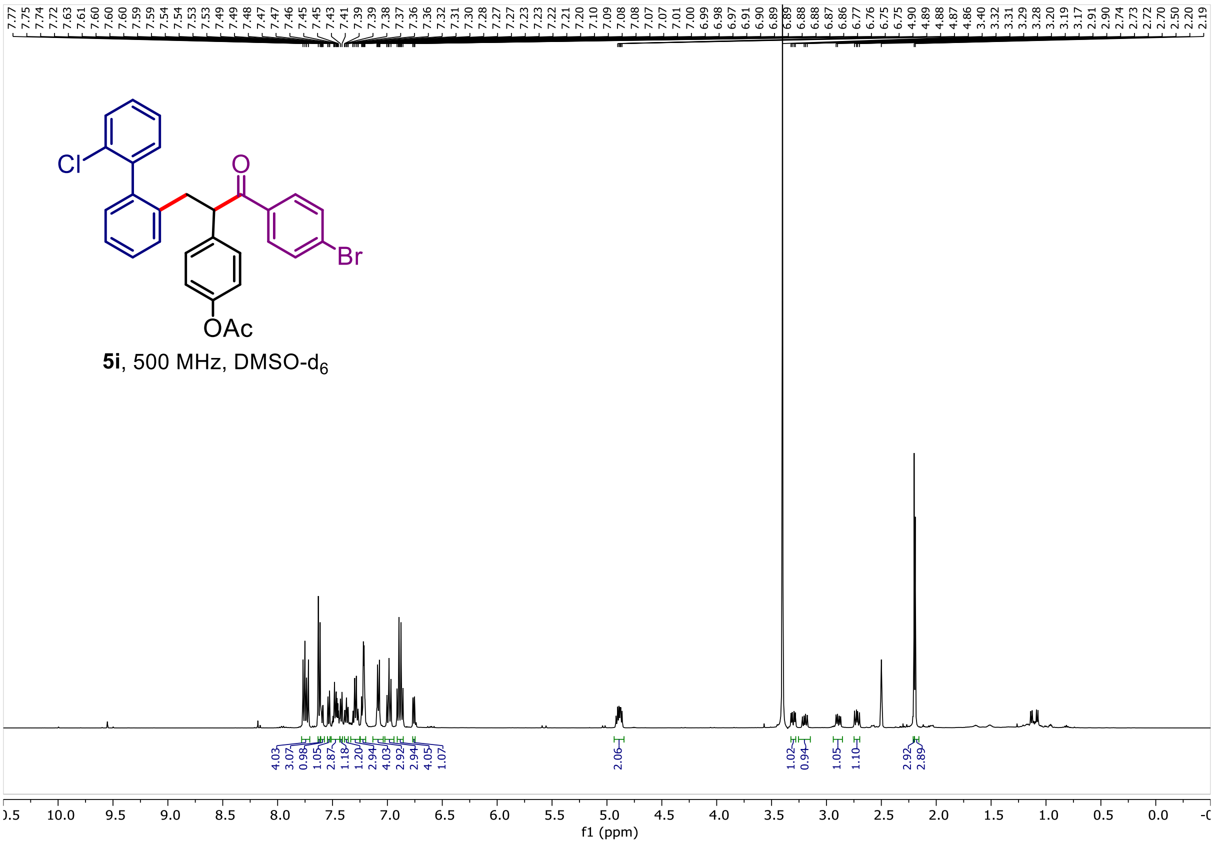

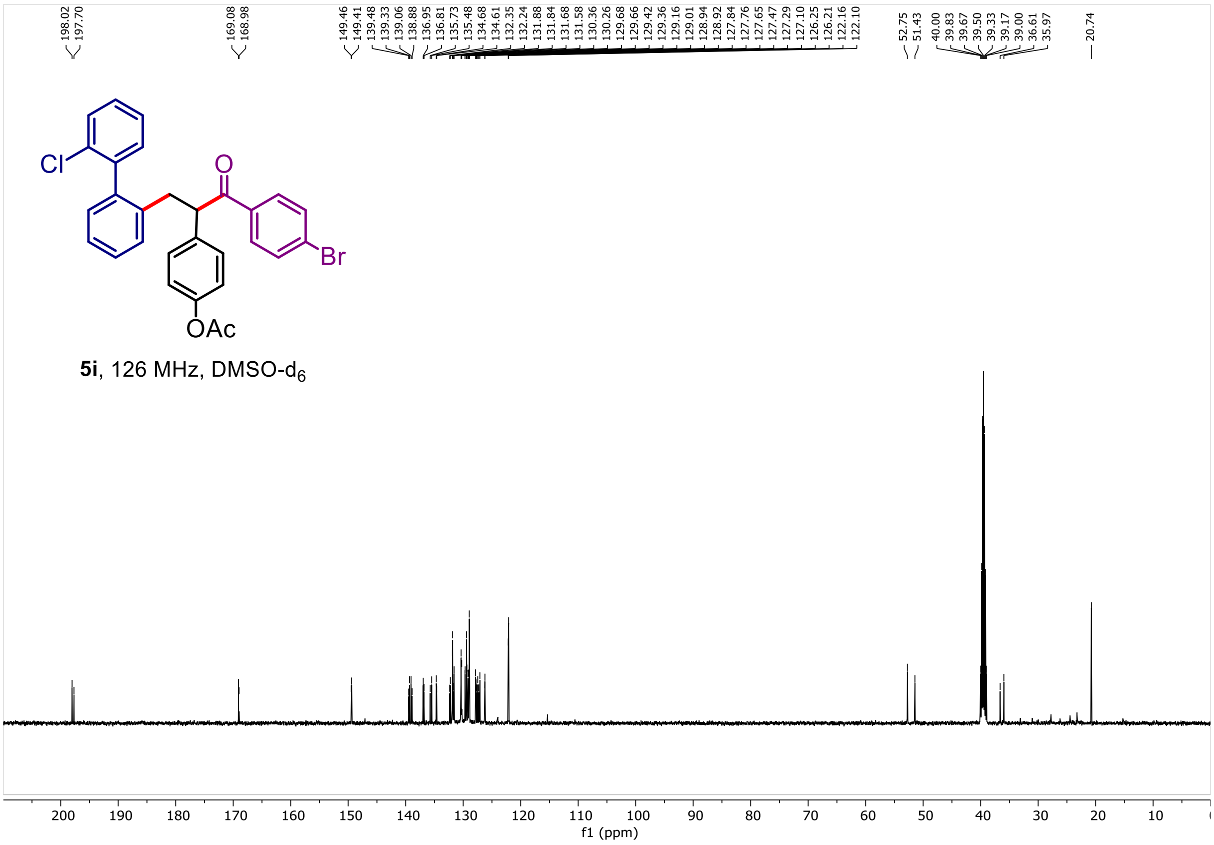

4-(1-(4-bromophenyl)-3-(2'-chloro-[1,1'-biphenyl]-2-yl)-1-oxopropan-2-yl)phenyl *tert*-butyl carbonate (5j)

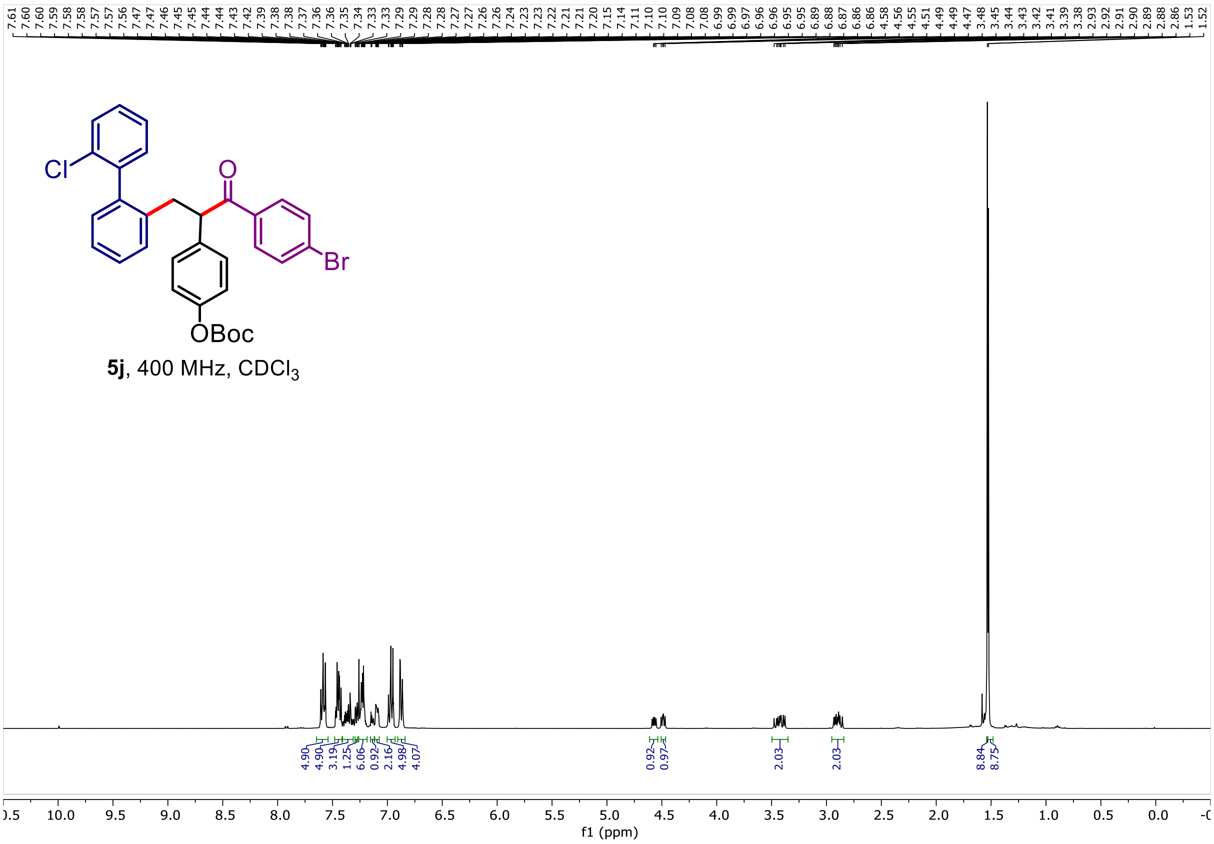

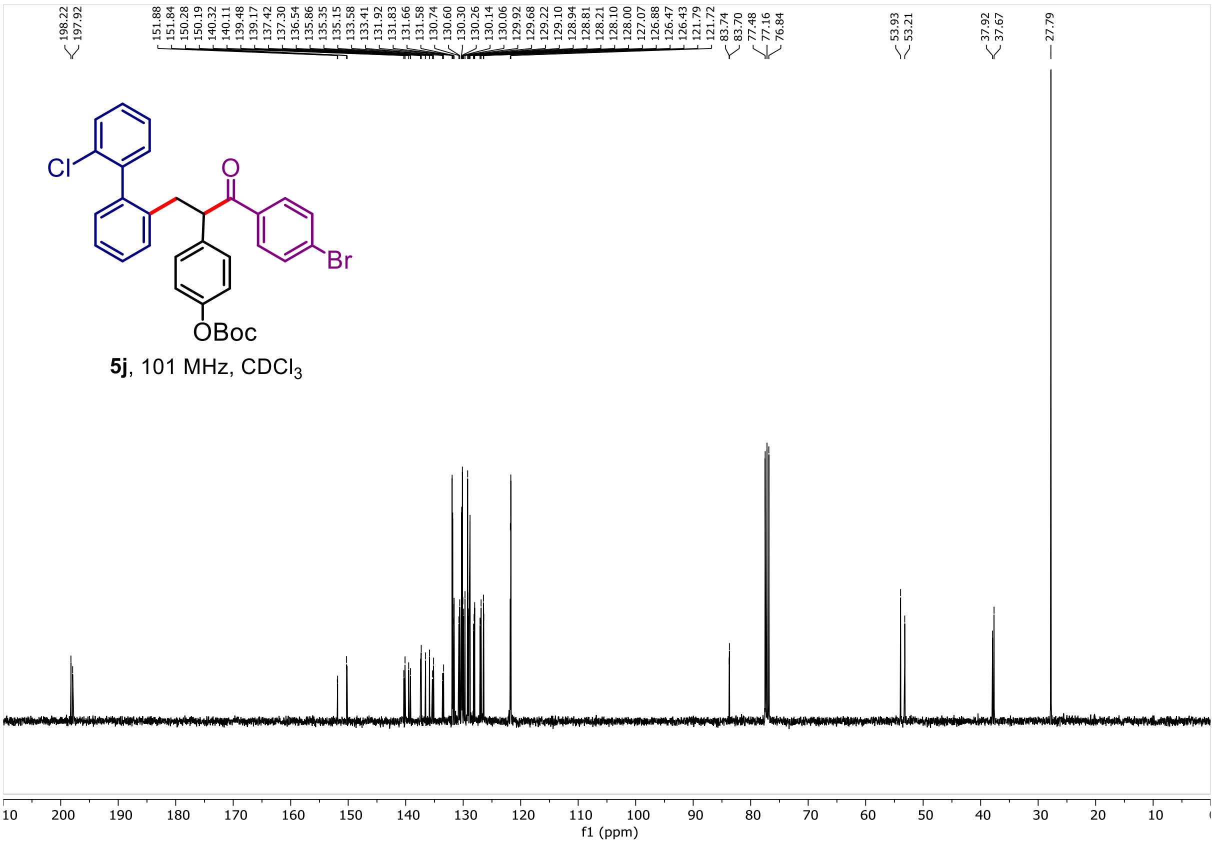

1-(4-bromophenyl)-3-(2'-chloro-[1,1'-biphenyl]-2-yl)-2-(naphthalen-2-yl)propan-1-one (5k)

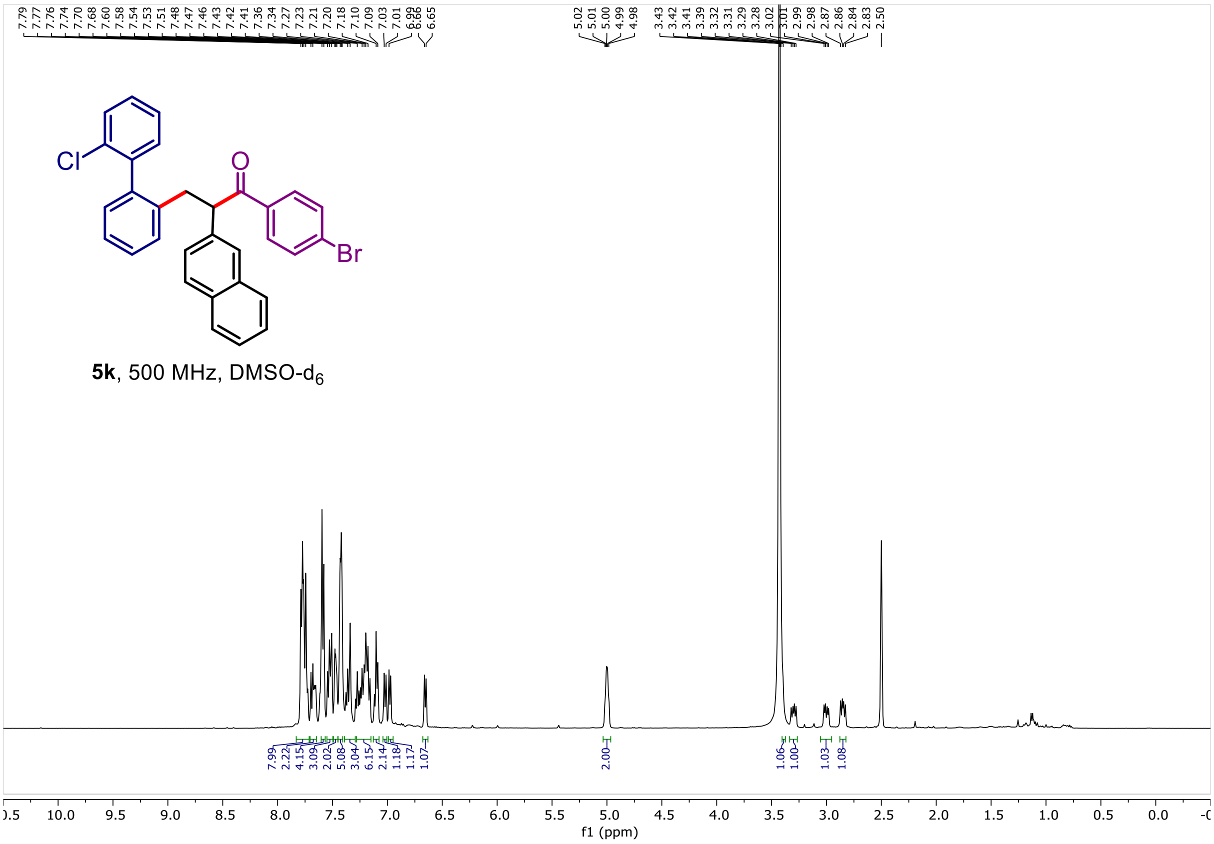

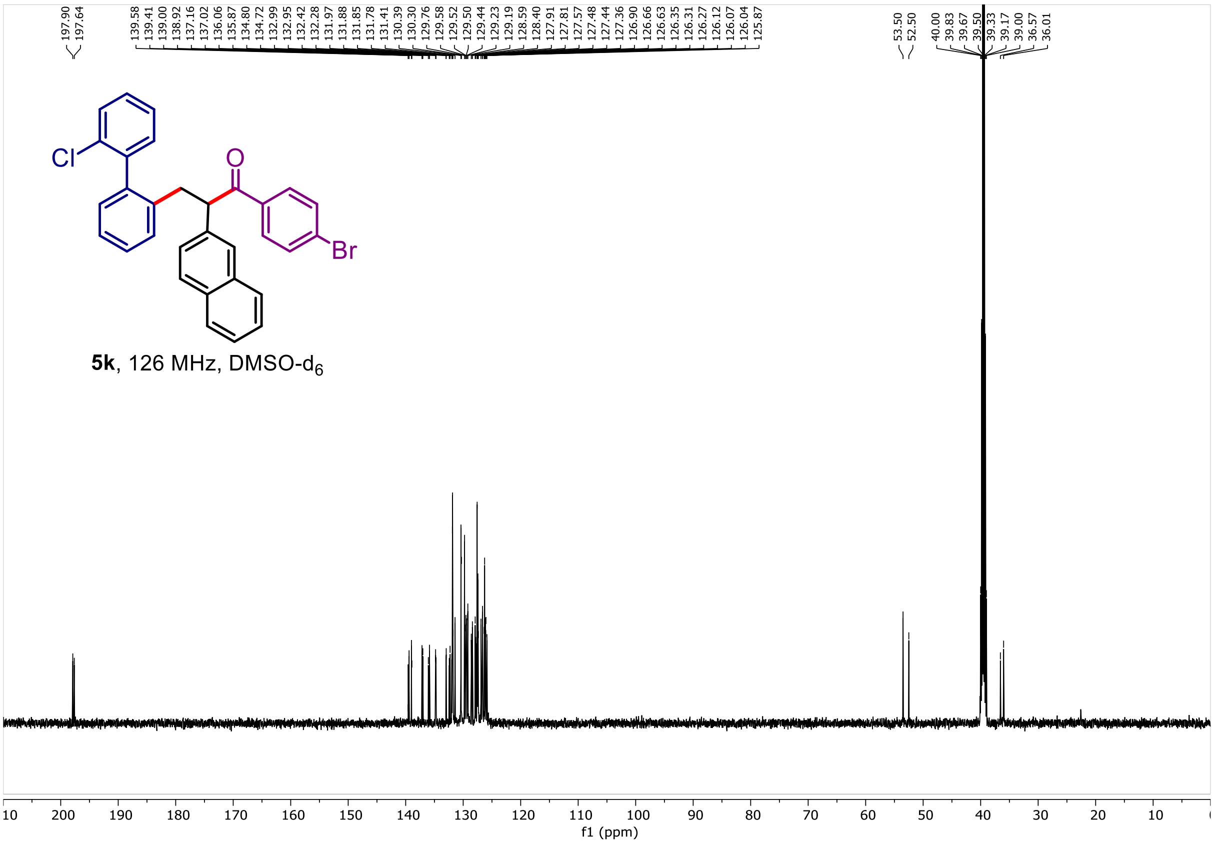

1-(4-bromophenyl)-3-(2'-chloro-[1,1'-biphenyl]-2-yl)-2-(pyridin-2-yl)propan-1-one (5l)

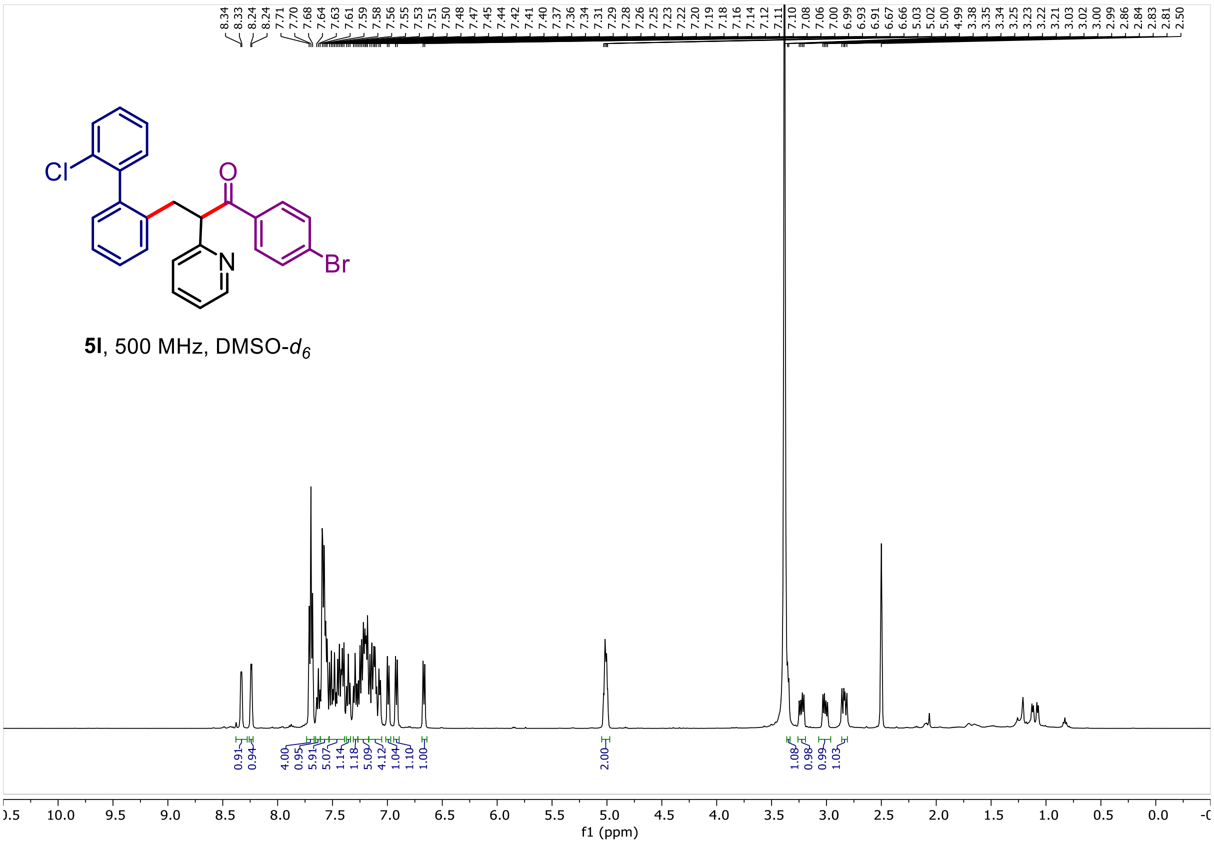

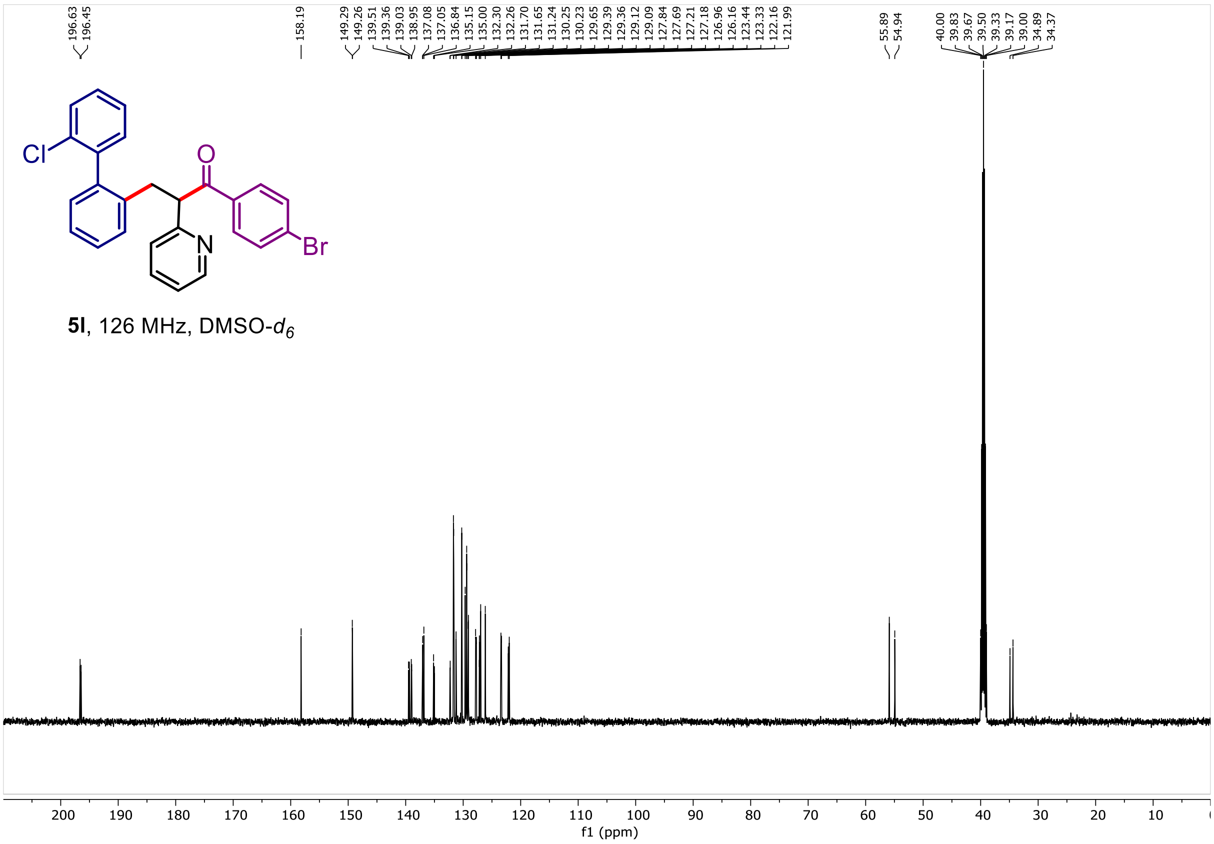

1-(4-bromophenyl)-3-(2'-chloro-[1,1'-biphenyl]-2-yl)-2-(thiophen-2-yl)propan-1-one (5m)

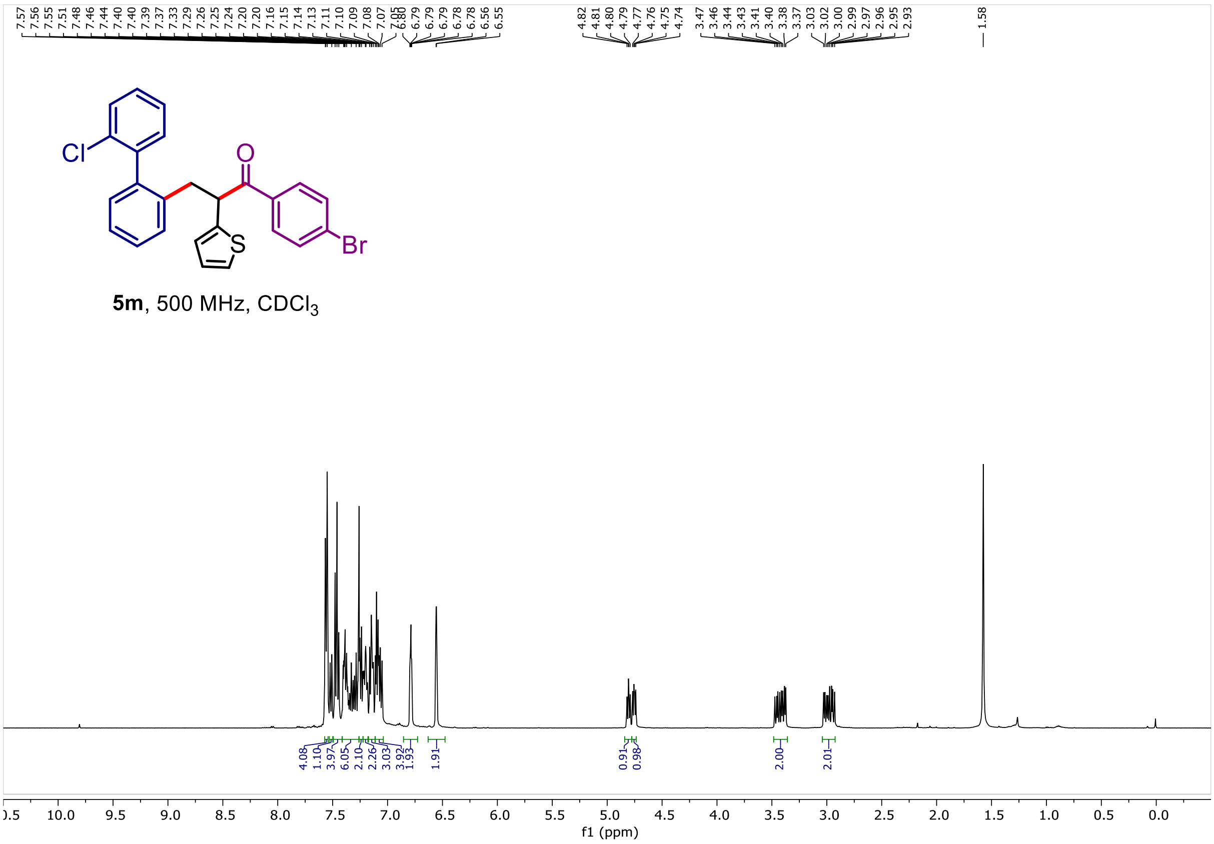

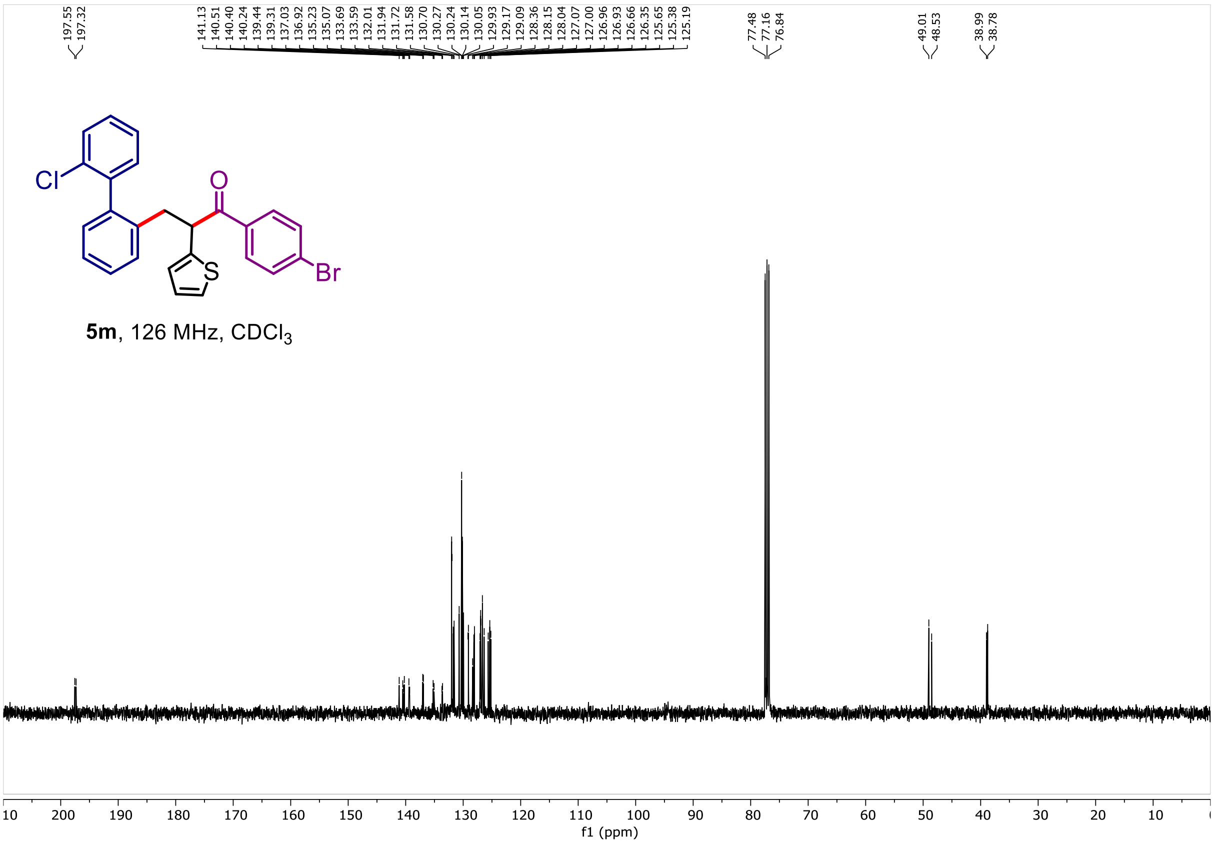

Ethyl -3-(4-bromophenyl)-2-((2'-chloro-[1,1'-biphenyl]-2-yl)methyl)-3-oxopropanoate (5n)

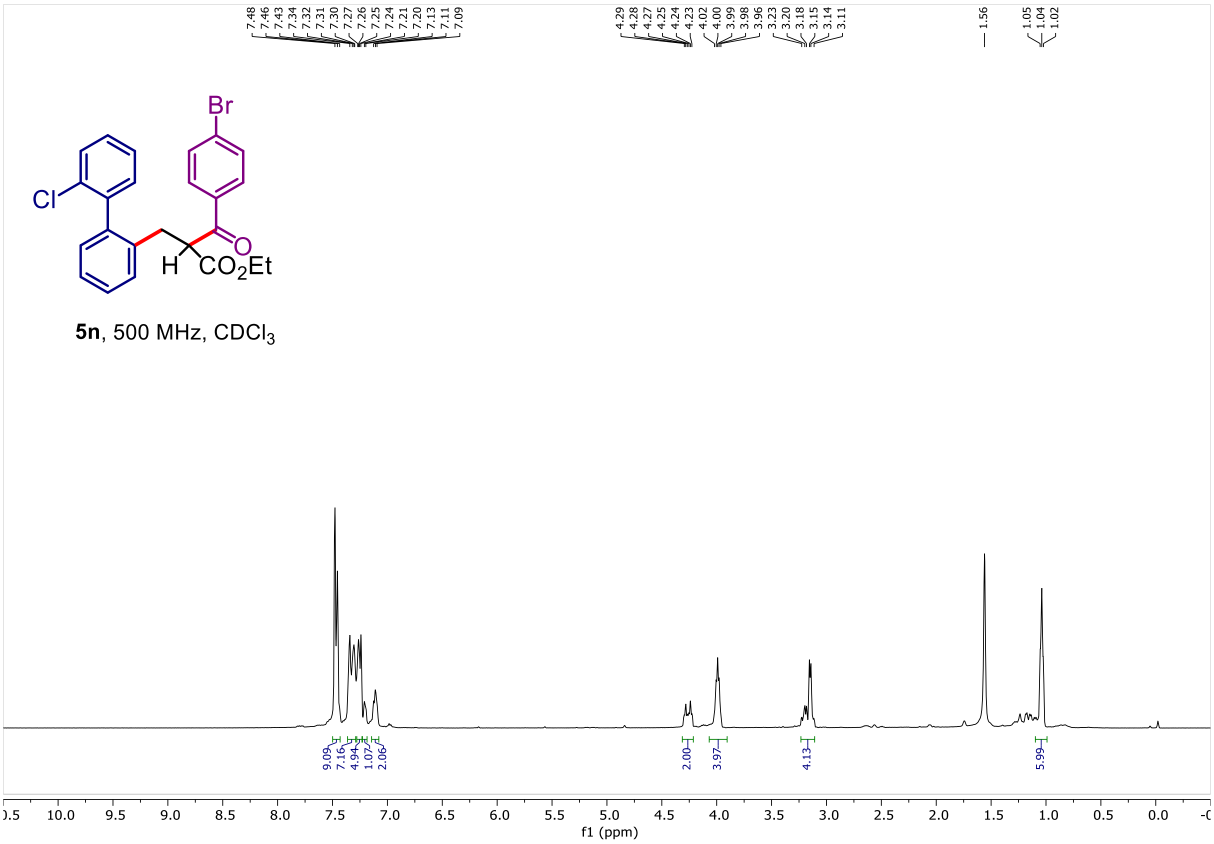

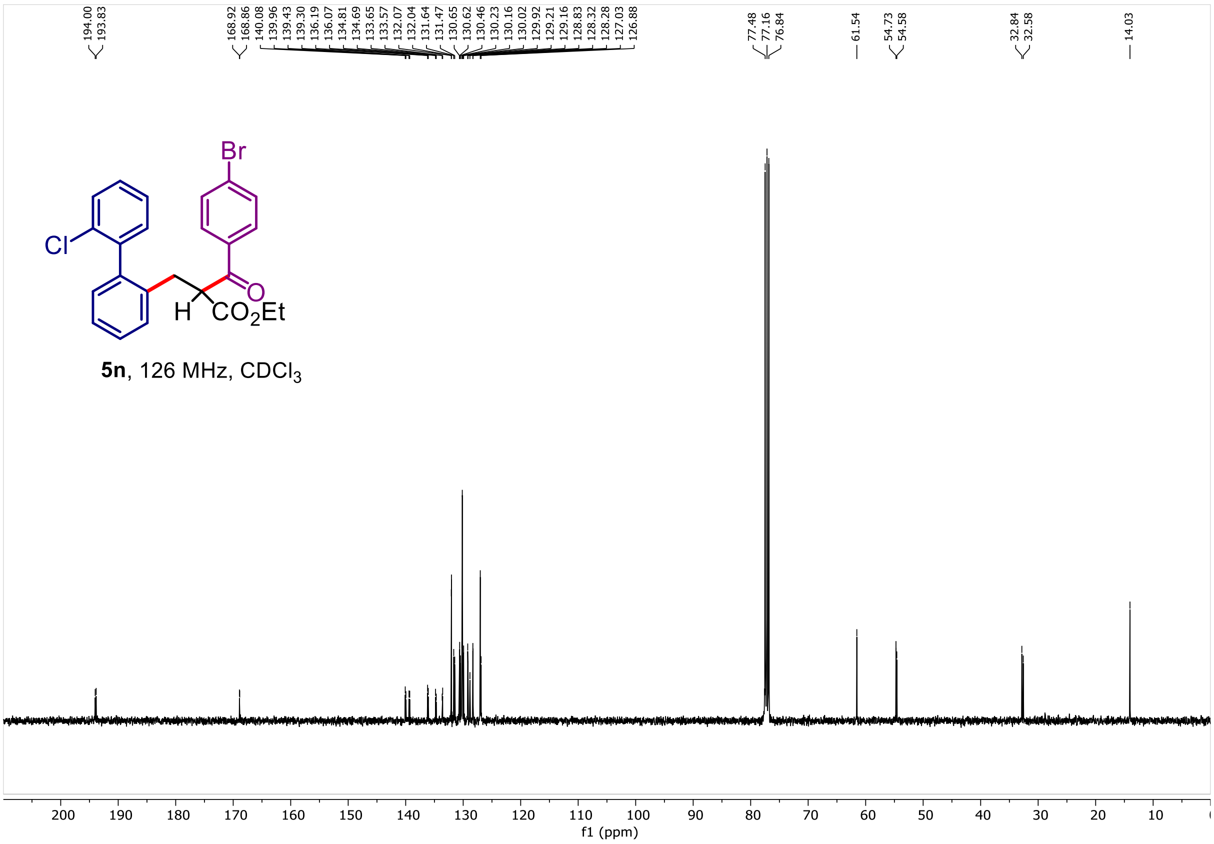

Methyl 3-(4-bromophenyl)-2-((2'-chloro-[1,1'-biphenyl]-2-yl)methyl)-2-methyl-3-oxopropanoate (5o)

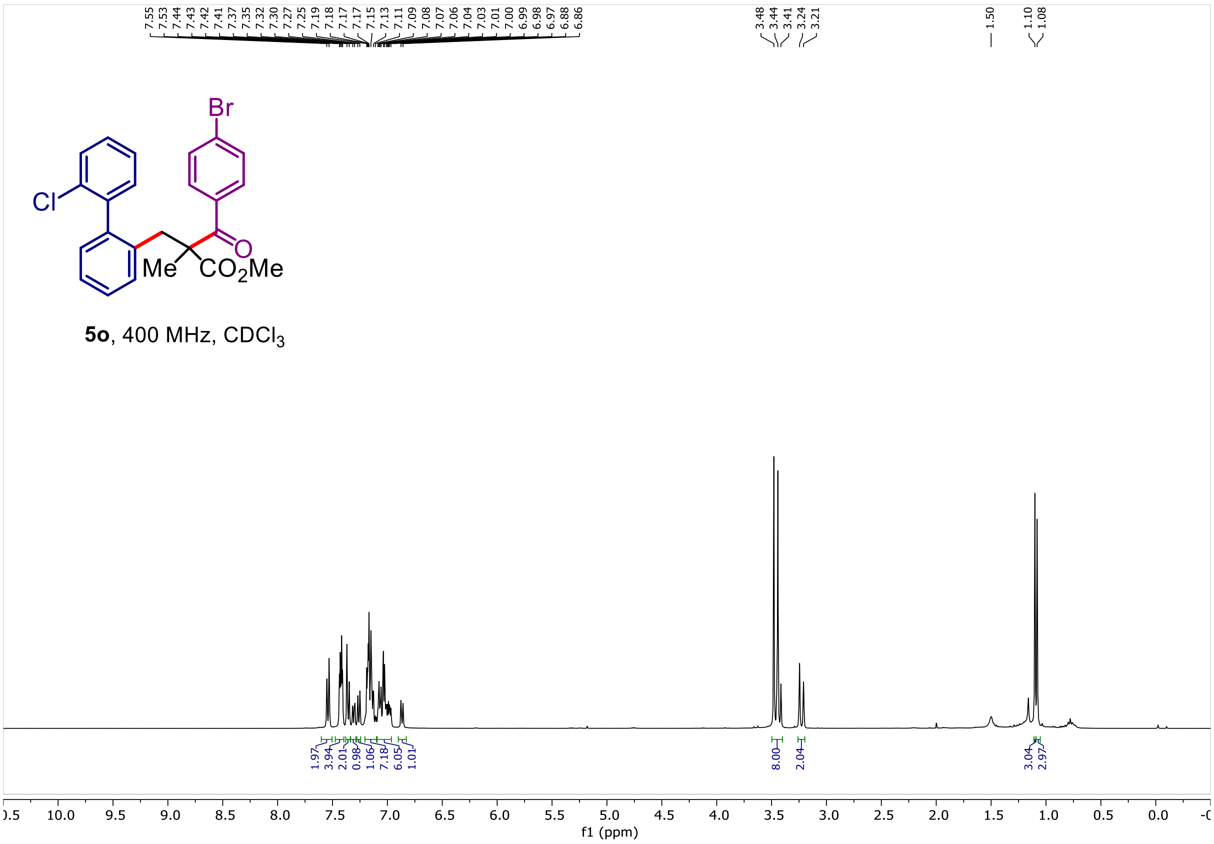

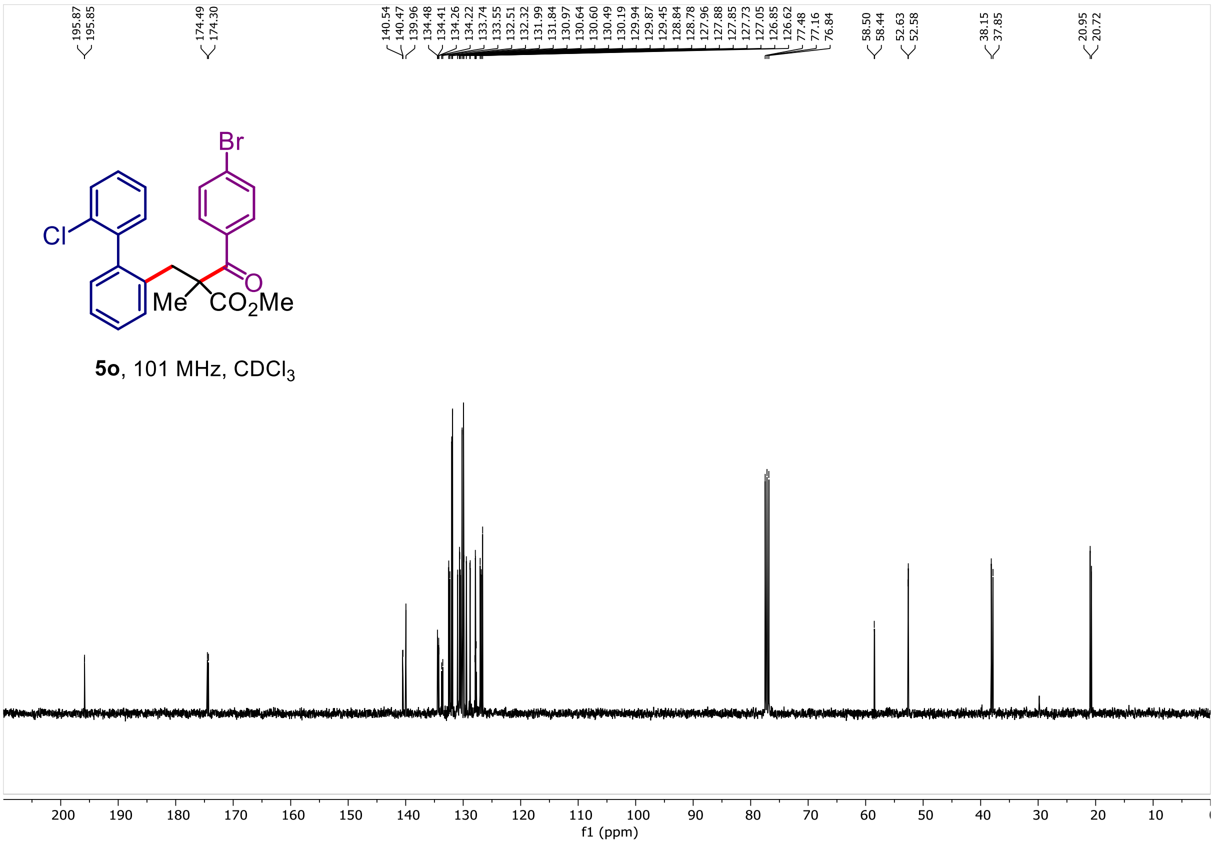

1-(4-bromophenyl)-3-(2'-chloro-5,5'-dimethyl-[1,1'-biphenyl]-2-yl)-2-phenylpropan-1-one (5p)

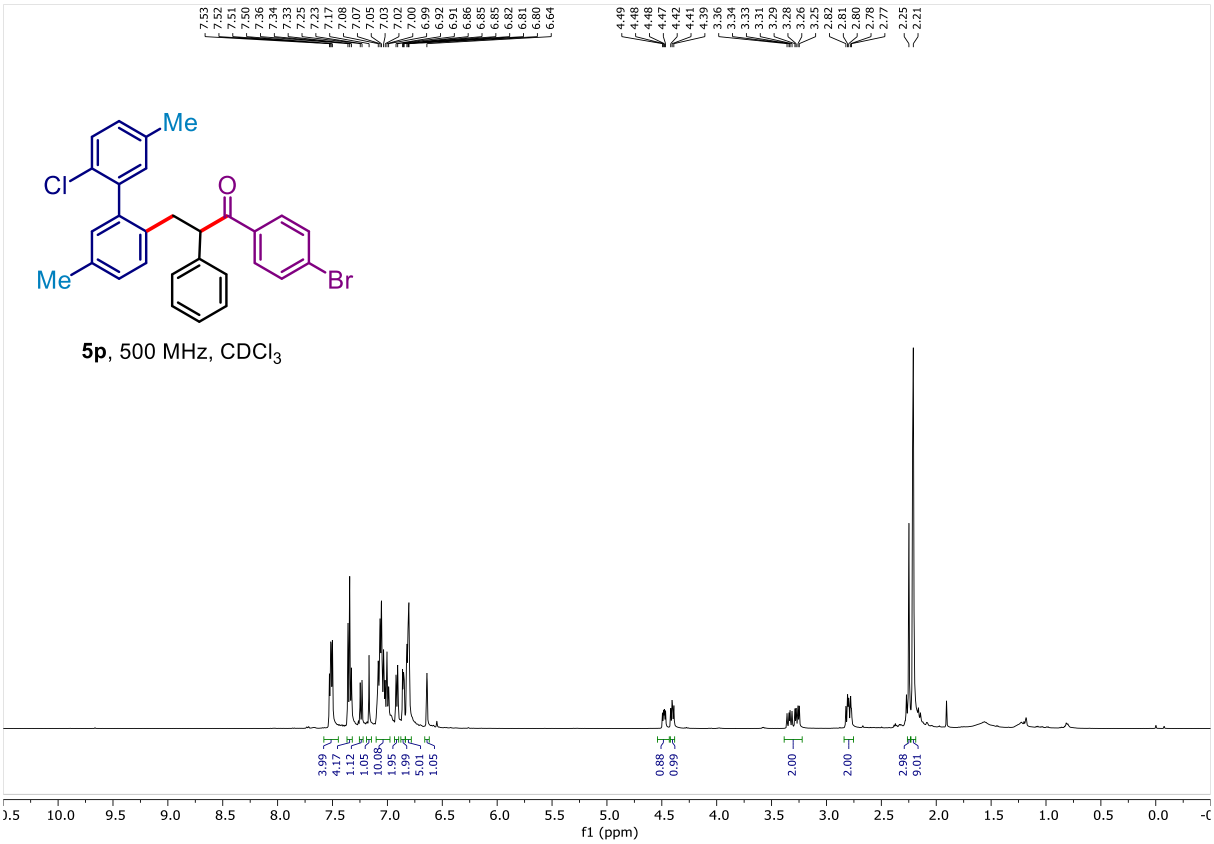

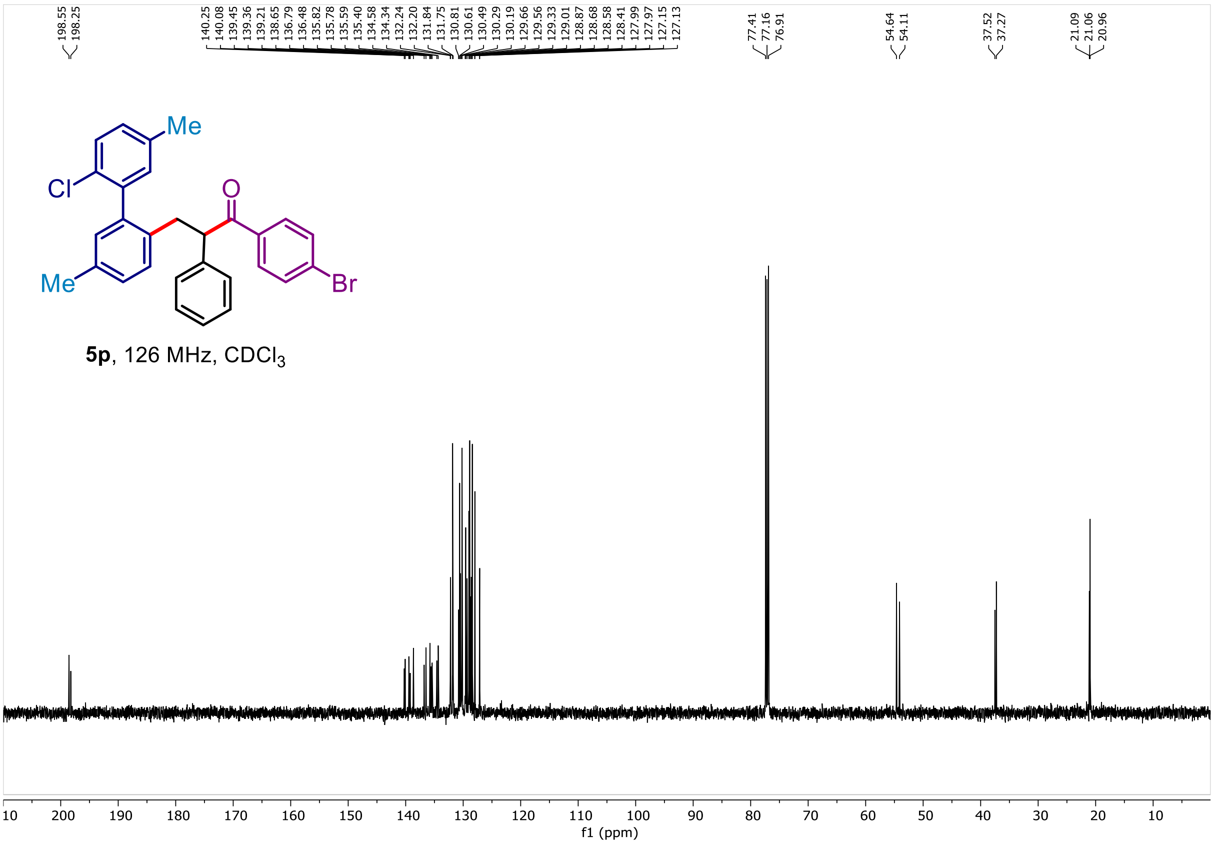

1-(4-bromophenyl)-3-(2'-chloro-5,5'-bis(trifluoromethyl)-[1,1'-biphenyl]-2-yl)-2-phenylpropan-1-one (5q)

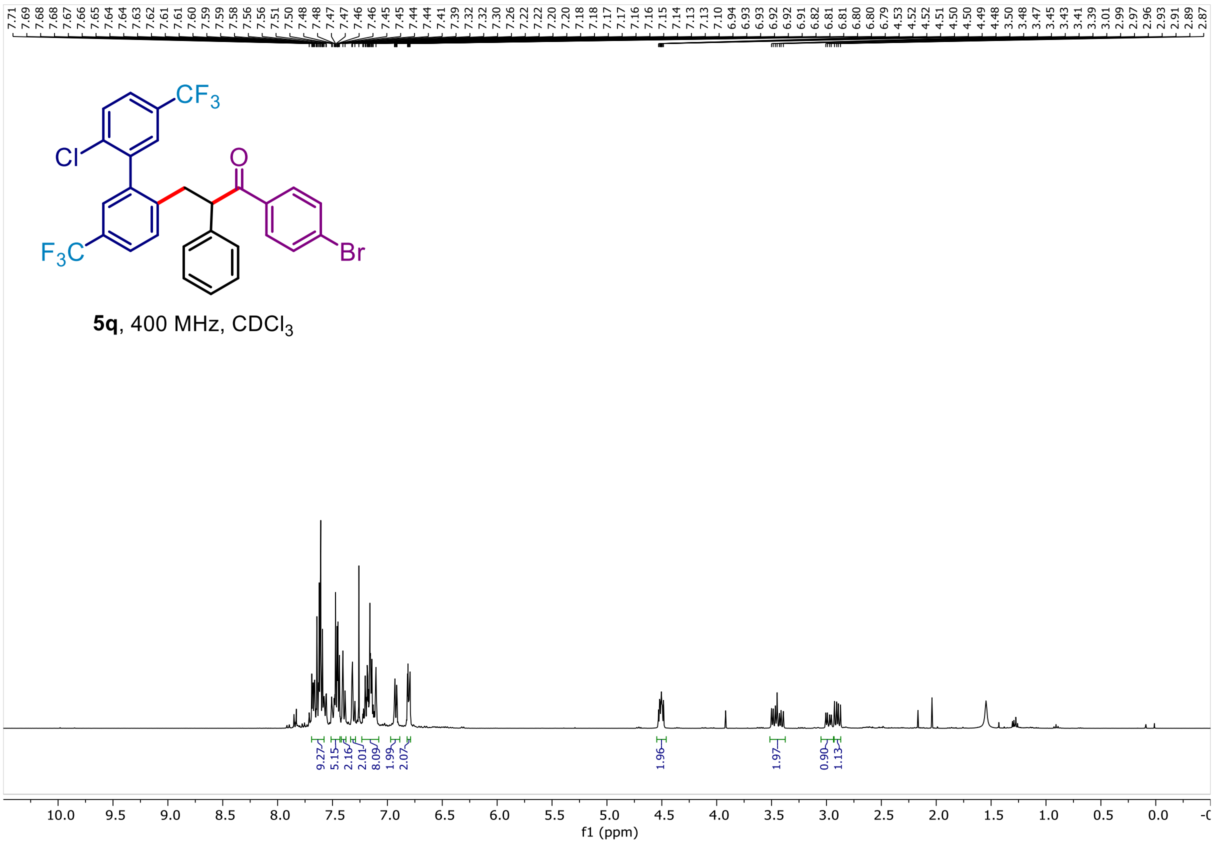

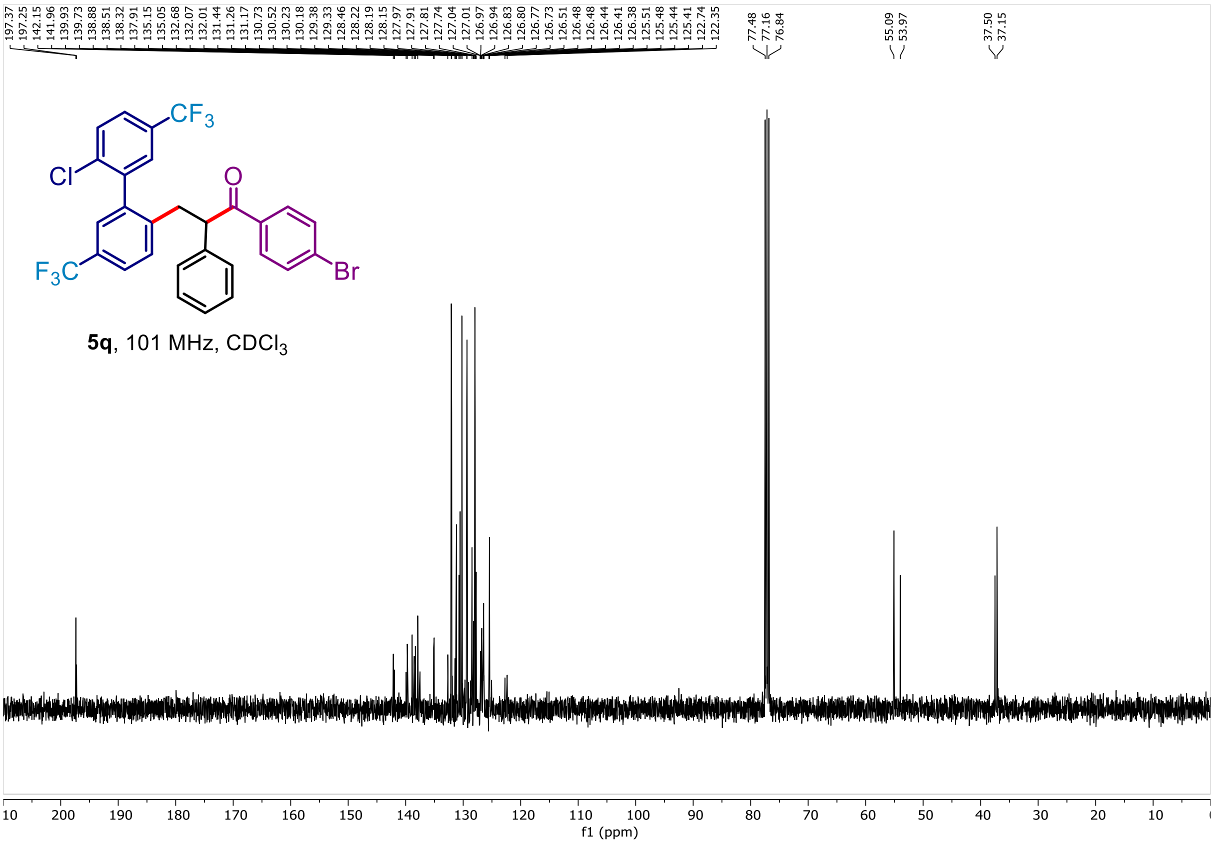
197.37
197.25
142.15
141.96
139.93
139.73
138.88
138.51
138.32
137.91
135.15
135.05
132.68
132.07
132.01
131.44
131.26
131.17
130.73
130.52
130.23
130.18
129.38
129.33
128.46
128.22
128.19
128.15
127.97
127.91
127.81
127.74
127.04
127.01
126.97
126.94
126.83
126.80
126.77
126.73
126.51
126.48
126.48
126.44
126.41
126.38
125.51
125.48
125.44
125.41
122.74
122.35
77.48
77.16
76.84
55.09
53.97
37.50
37.15
CF3
Cl
O
F3C
Br
5q, 101 MHz, CDCl3
f1 (ppm)

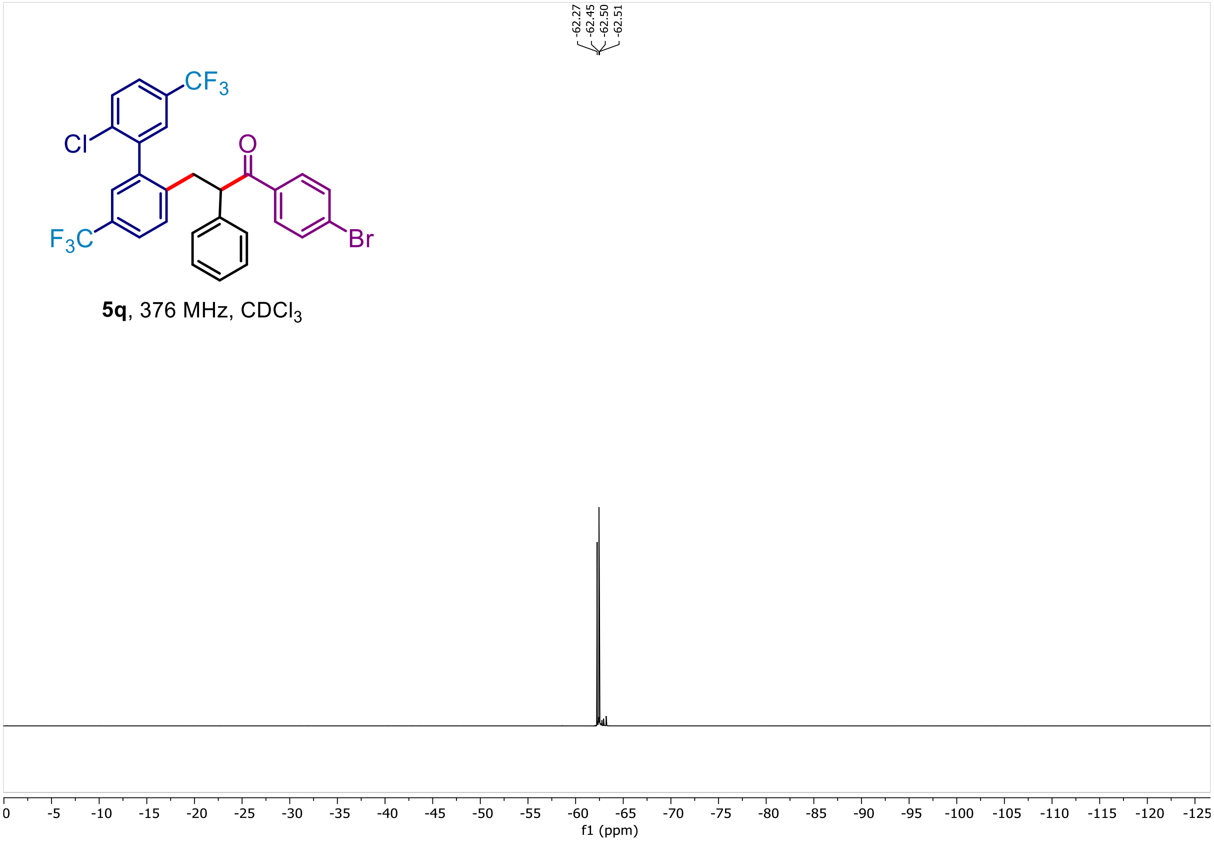
-62.27
-62.45
-62.50
-62.51
CF3
Cl
O
F3C
Br
5q, 376 MHz, CDCl3
f1 (ppm)

1-(4-bromophenyl)-3-(2'-chloro-5-methyl-[1,1'-biphenyl]-2-yl)-2-phenylpropan-1-one (5r) & 1-(4-bromophenyl)-3-(2'-chloro-5'-methyl-[1,1'-biphenyl]-2-yl)-2-phenylpropan-1-one (5r')

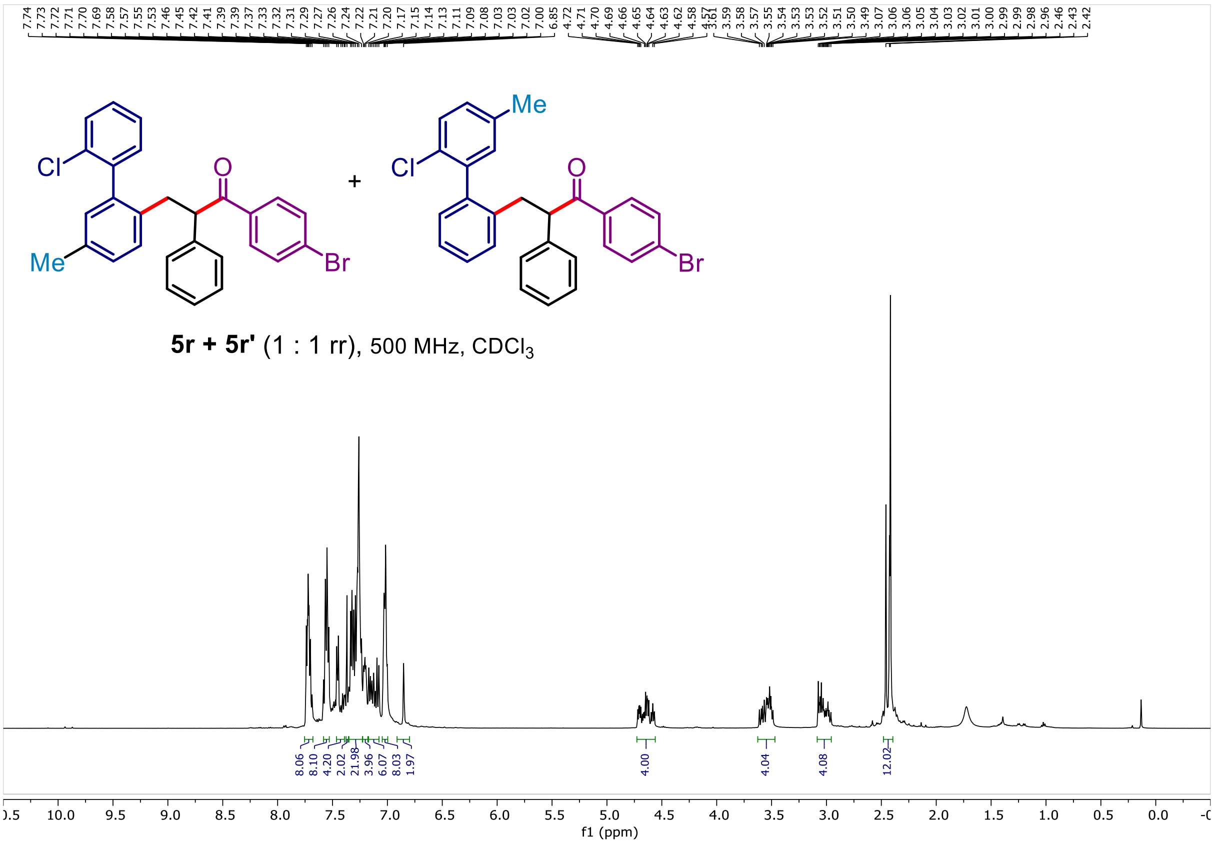

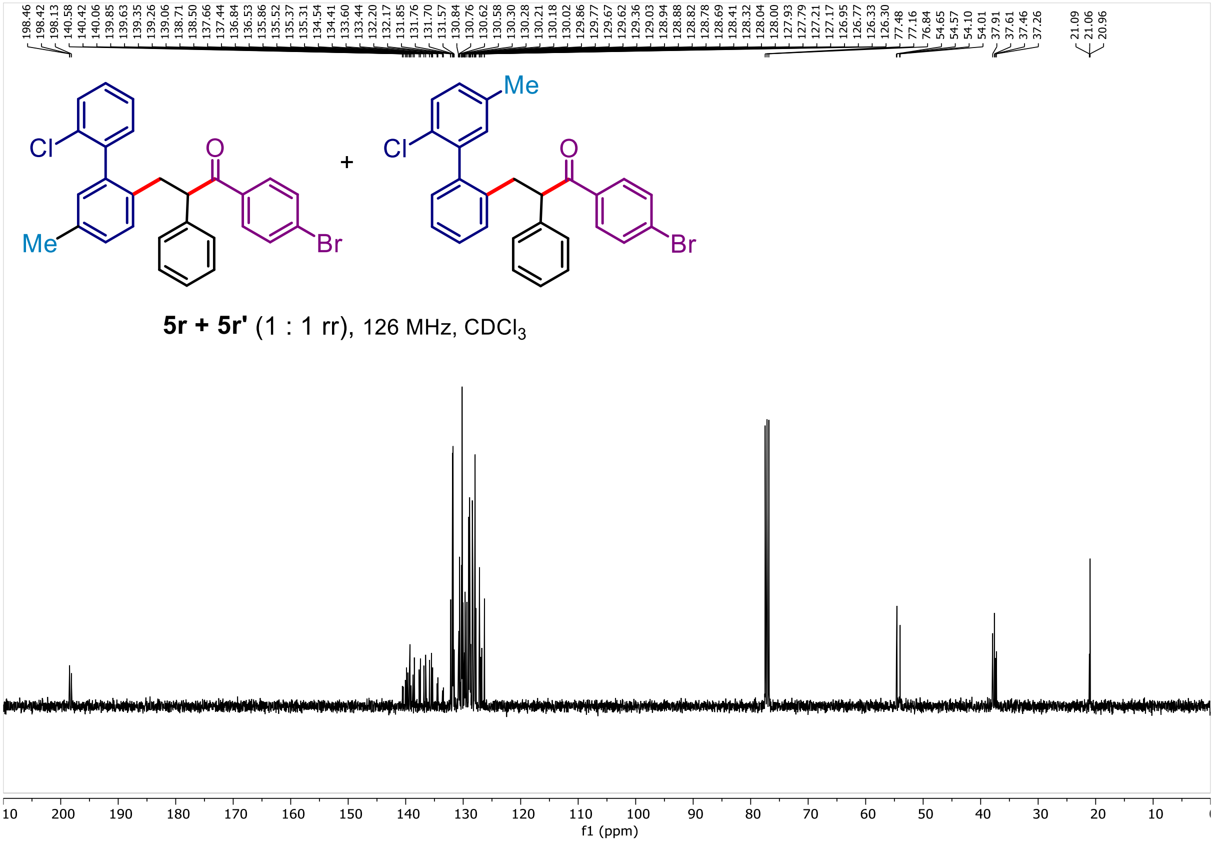

Methyl-6-(3-(4-bromophenyl)-3-oxo-2-phenylpropyl)-2'-chloro-[1,1'-biphenyl]-3-carboxylate(5s) & methyl-2'-(3-(4-bromophenyl)-3-oxo-2-phenylpropyl)-6-chloro-[1,1'-biphenyl]-3-carboxylate (5s')

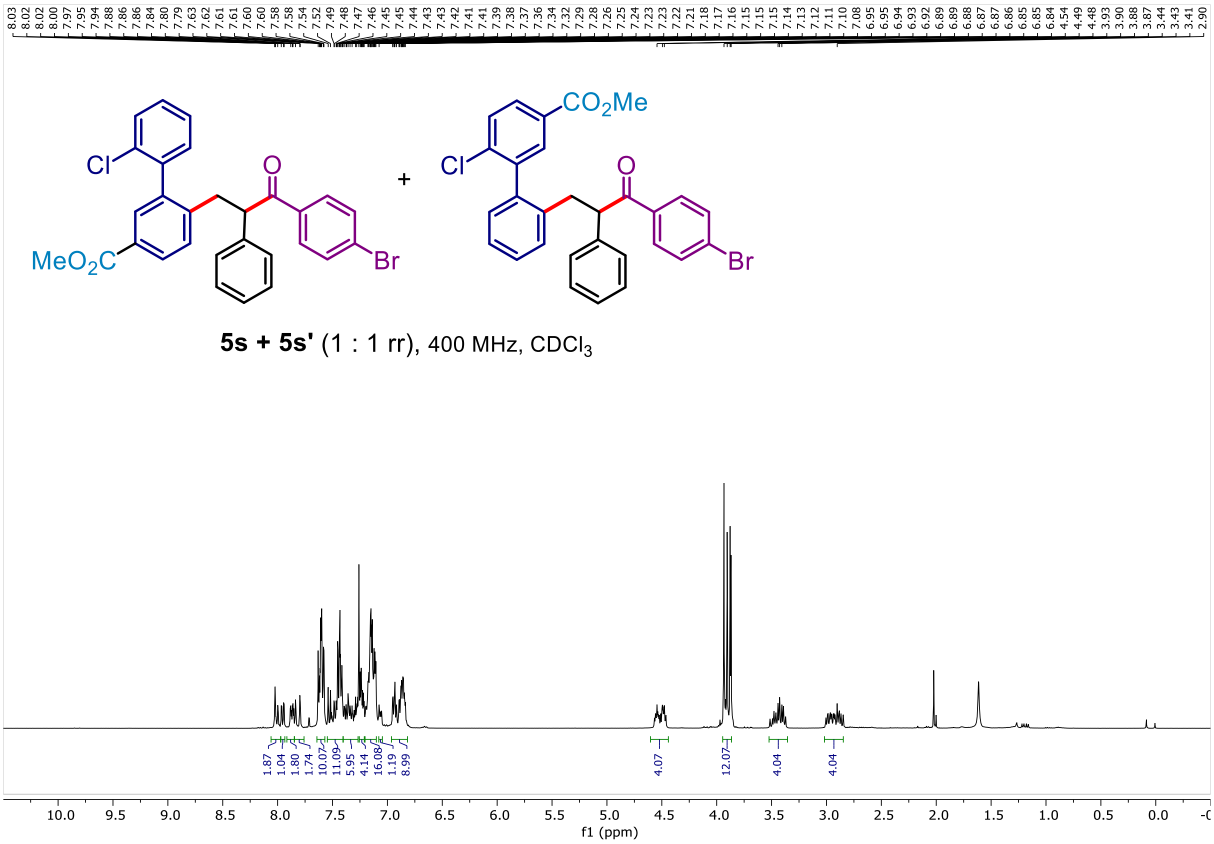

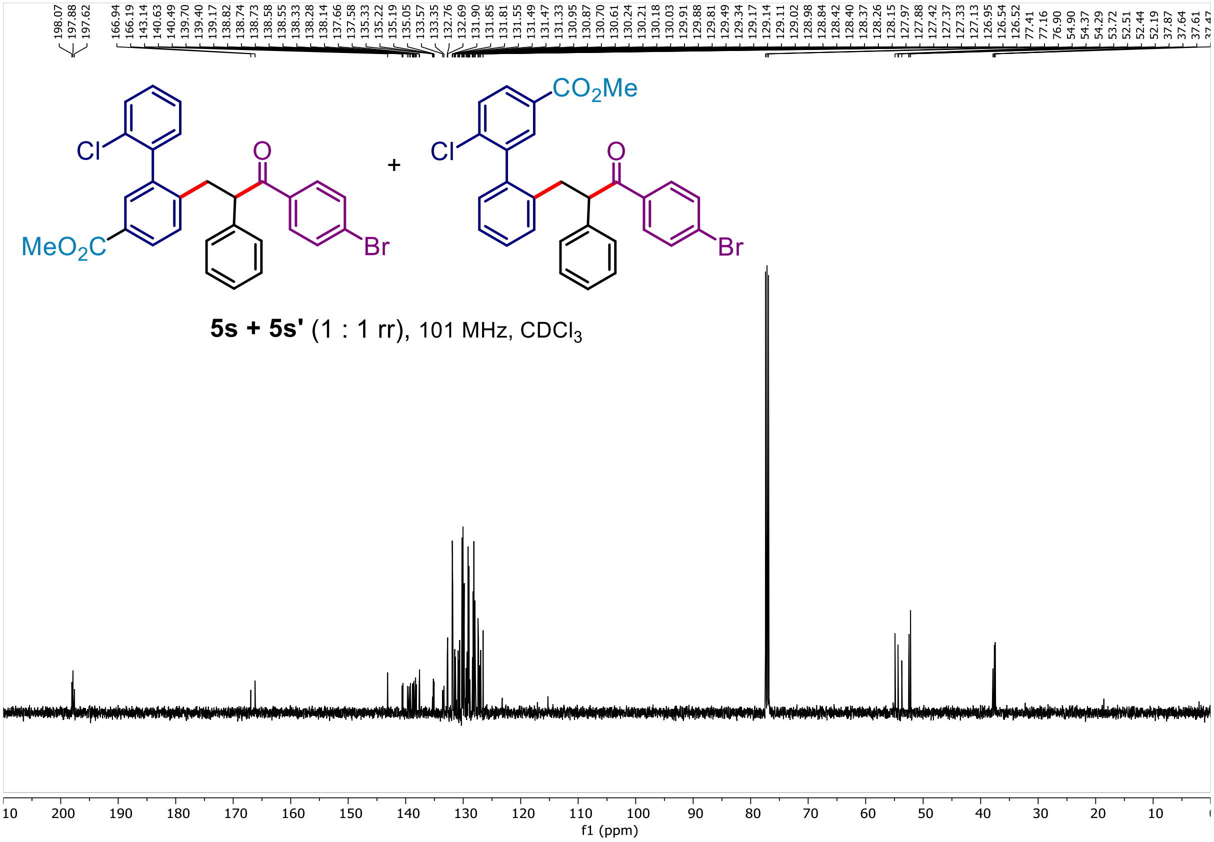

Methyl 2-(3-(4-bromophenyl)-3-oxo-2-phenylpropyl)-2'-chloro-[1,1'-biphenyl]-4-carboxylate(5t)

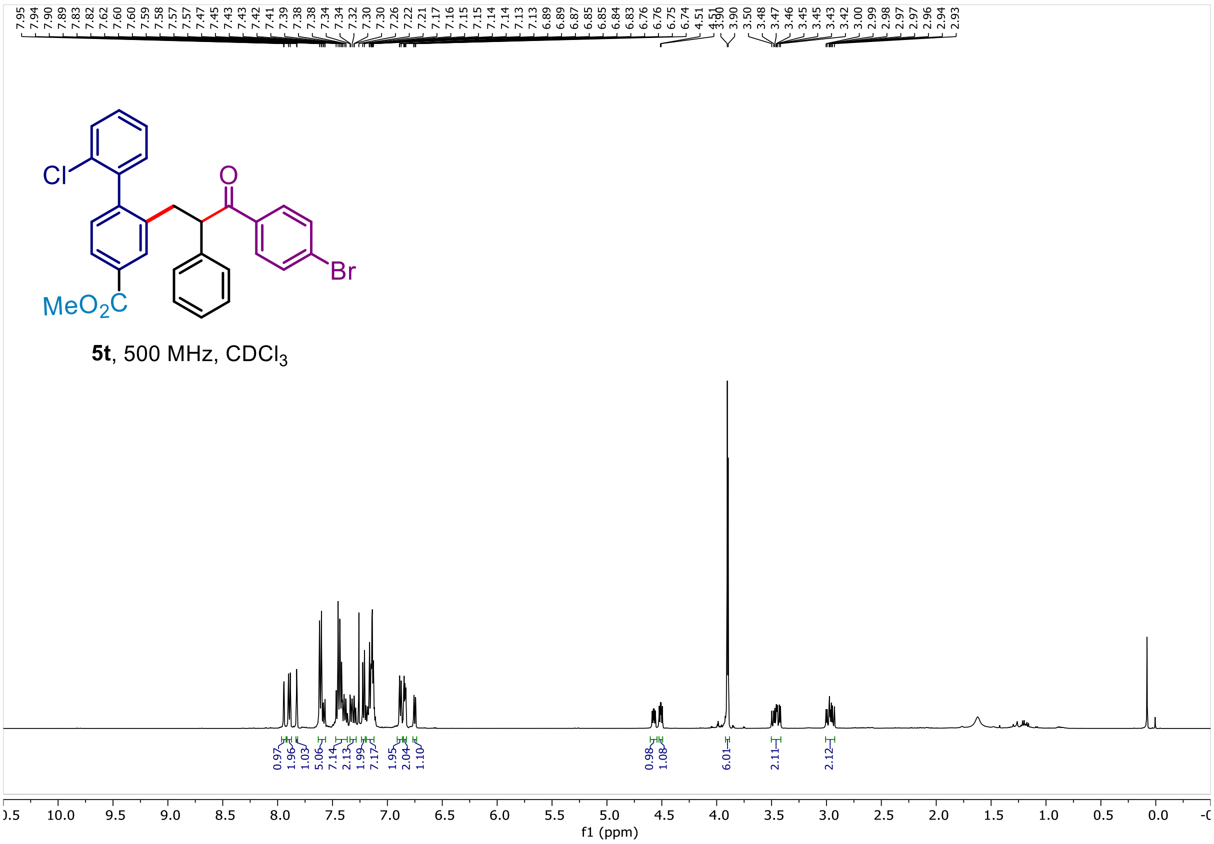

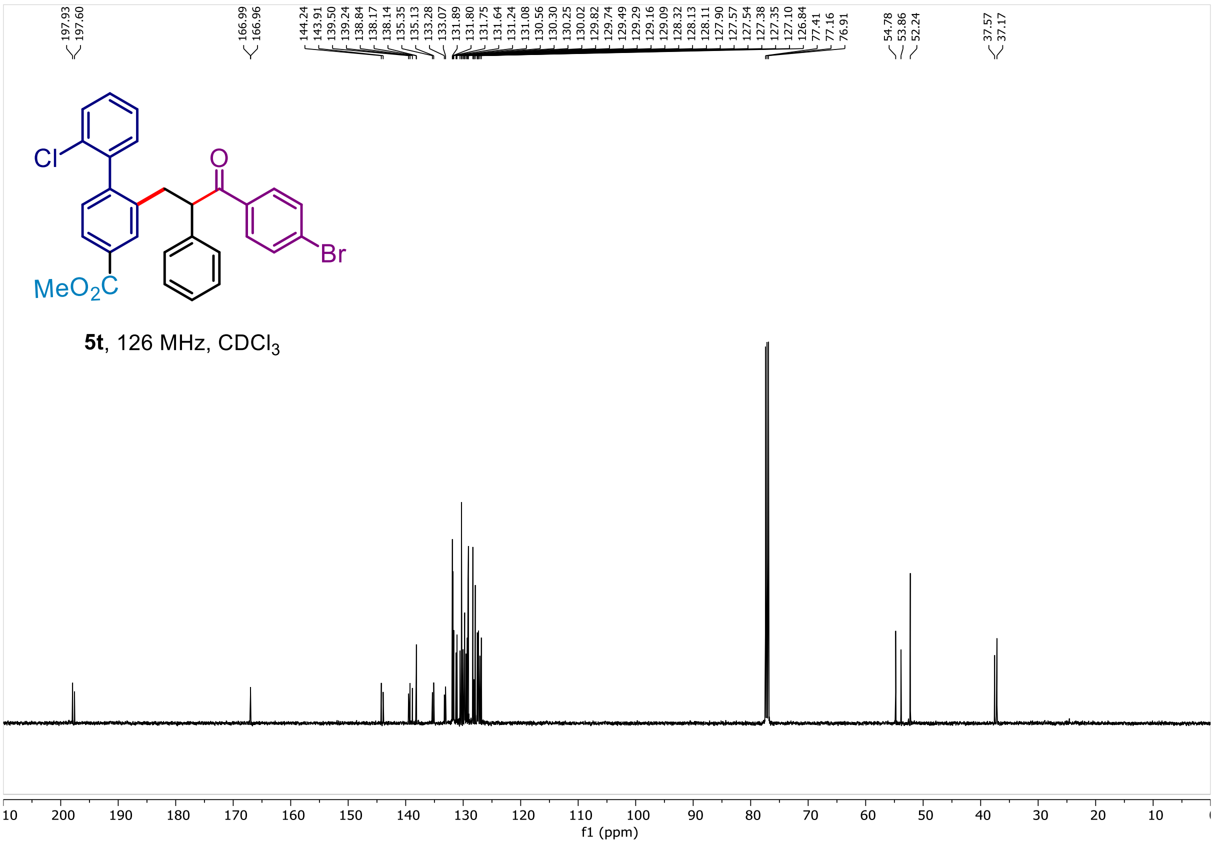

Methyl -2'-(3-(4-bromophenyl)-3-oxo-2-phenylpropyl)-2-chloro-[1,1'-biphenyl]-4-carboxylate (5t')

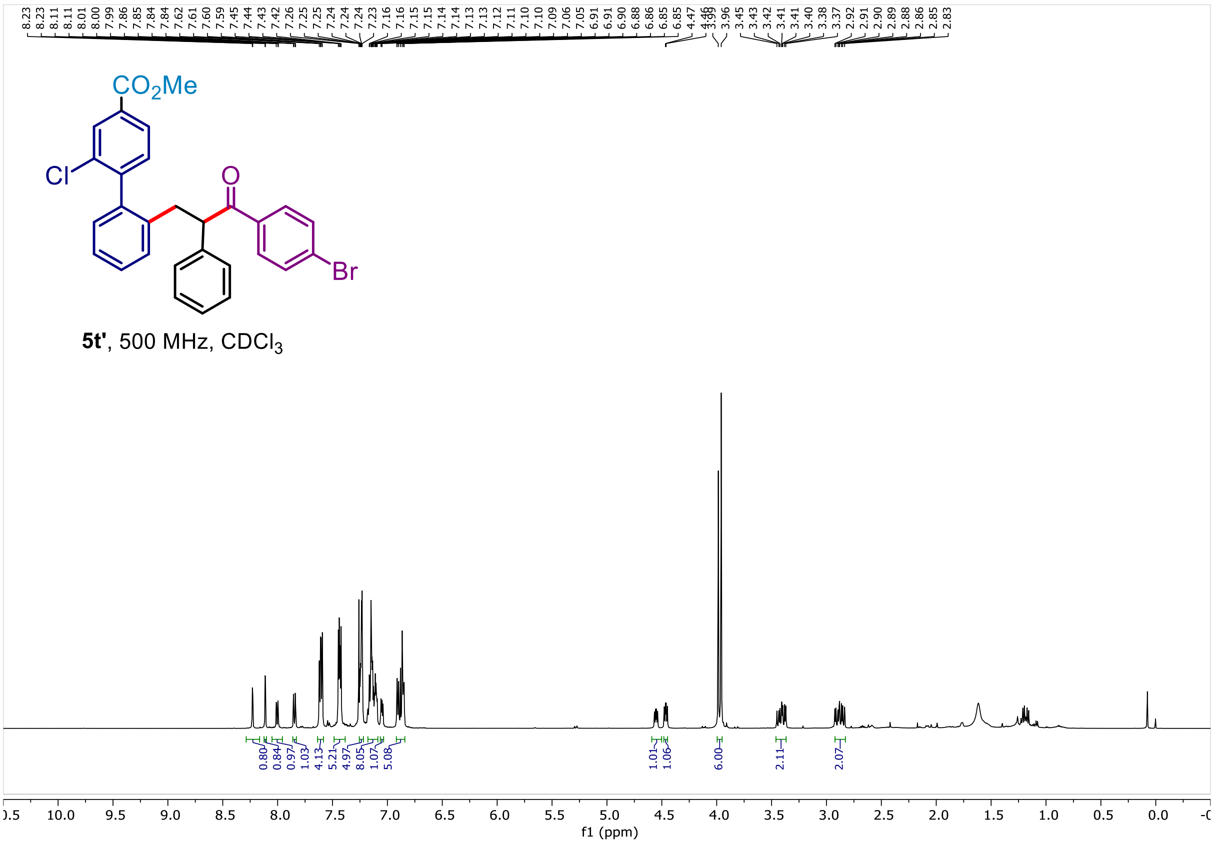

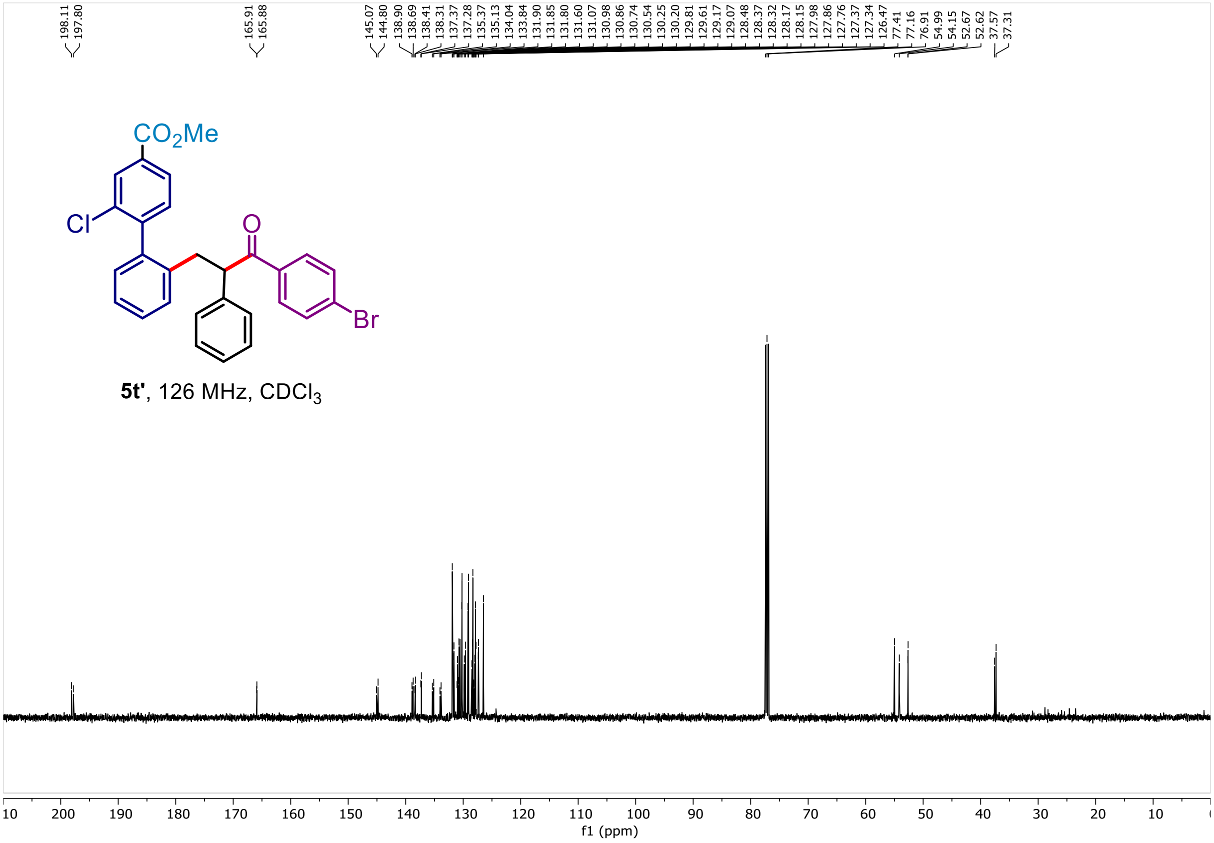

(4-bromophenyl)(2-(2'-chloro-[1,1'-biphenyl]-2-yl)-1,2-dihydroacenaphthylen-1-yl)methanone (7a)

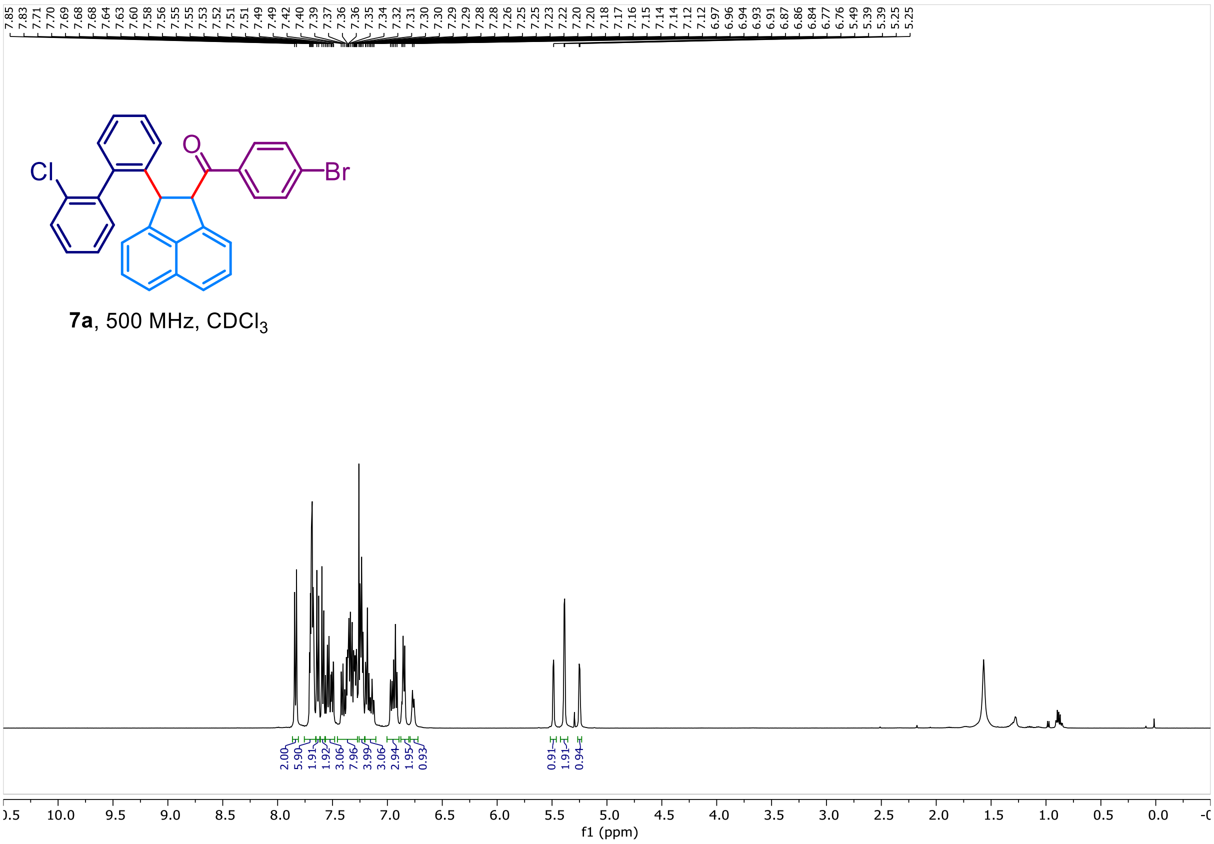

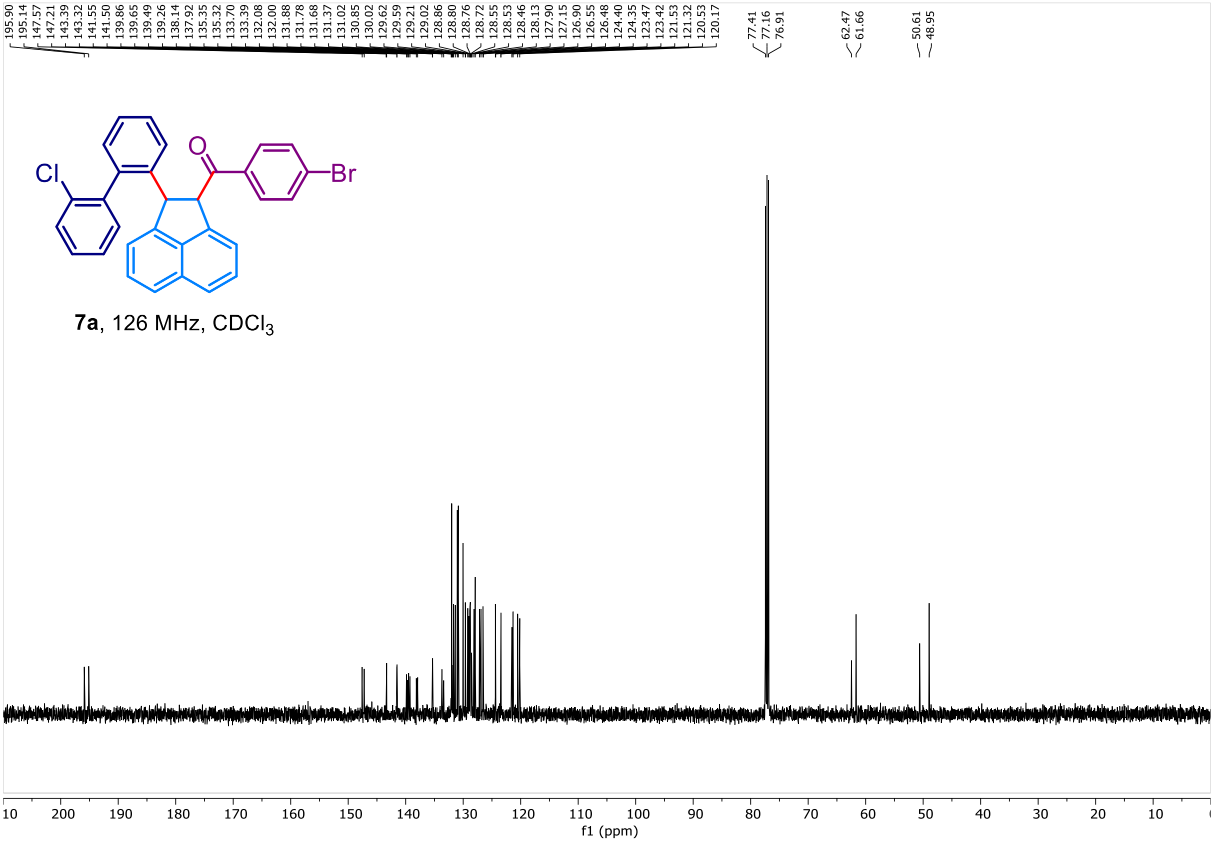

(2-(2'-chloro-[1,1'-biphenyl]-2-yl)-1,2-dihydroacenaphthylen-1-yl)(4-nitrophenyl)methanone(7b)

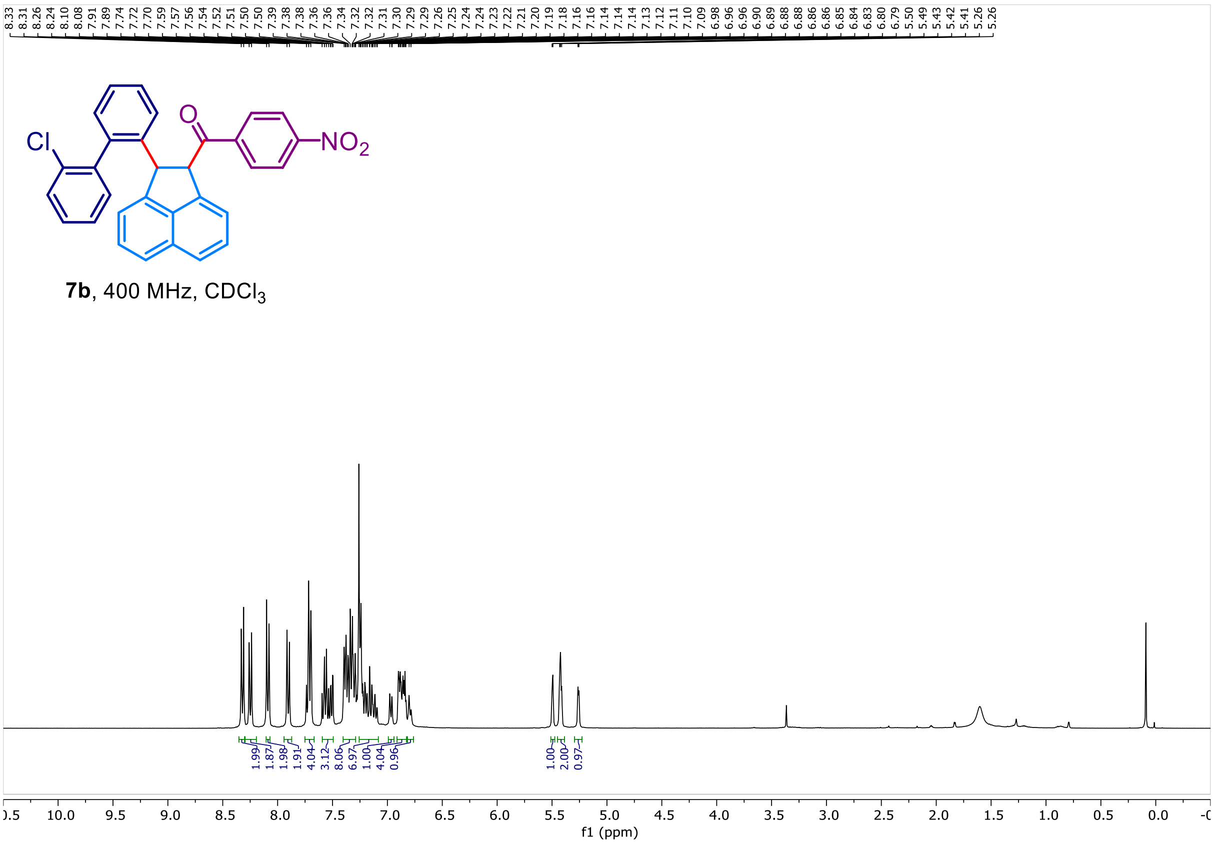

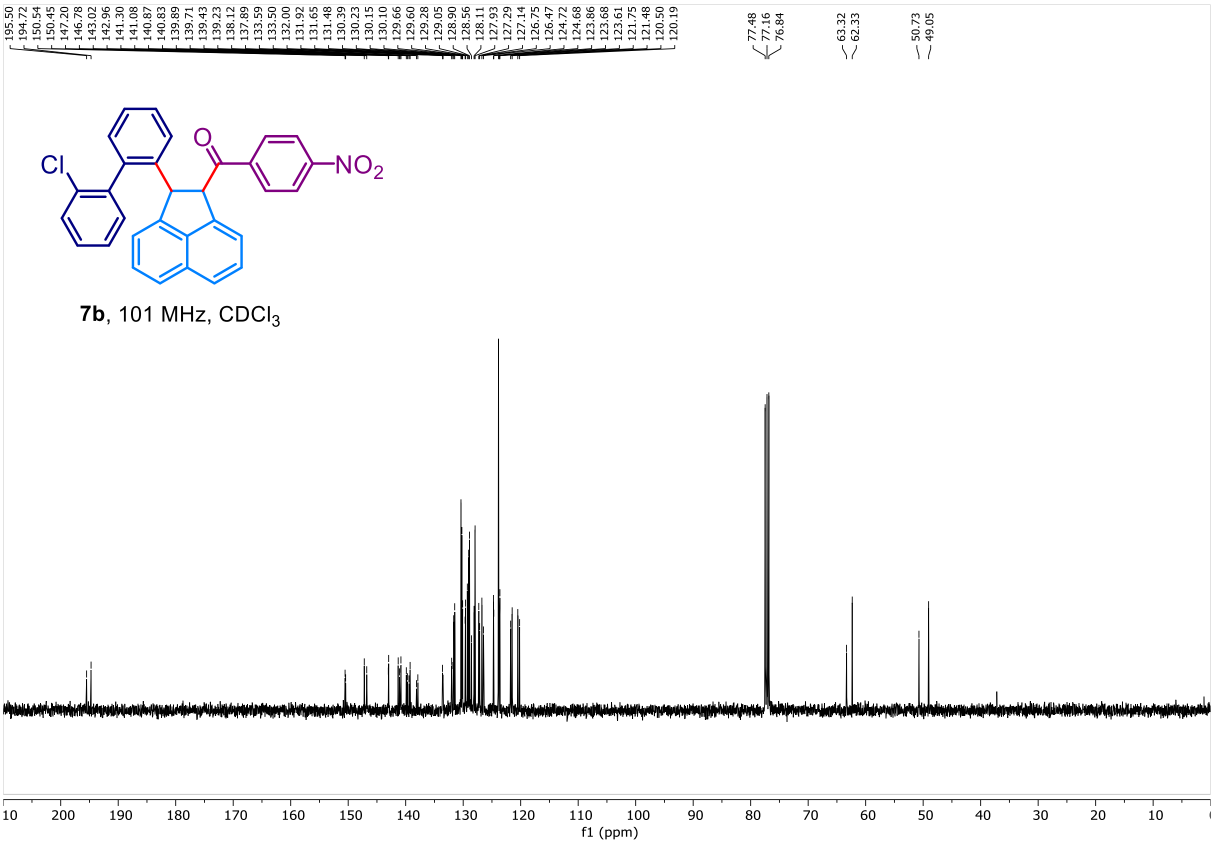

4-(2-(2'-chloro-[1,1'-biphenyl]-2-yl)-1,2-dihydroacenaphthylene-1-carbonyl)benzonitrile(7c)

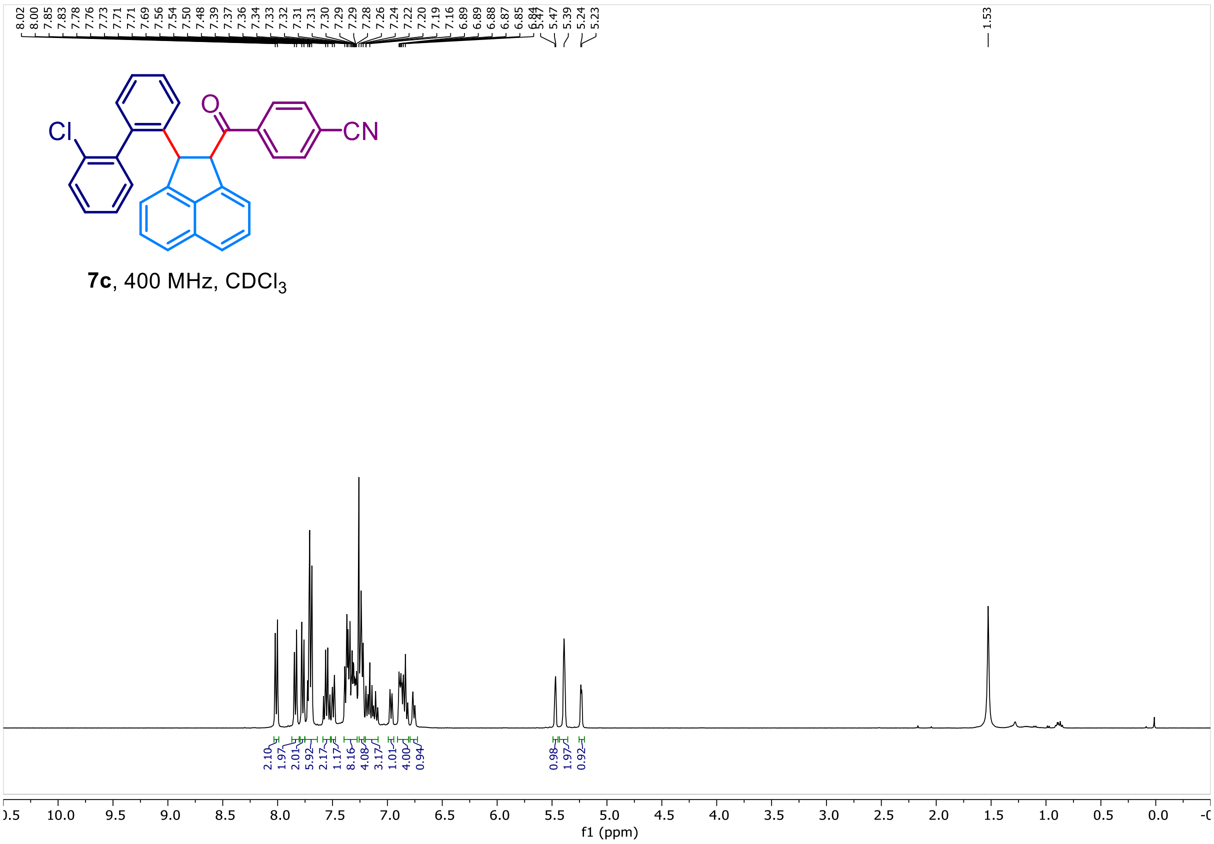

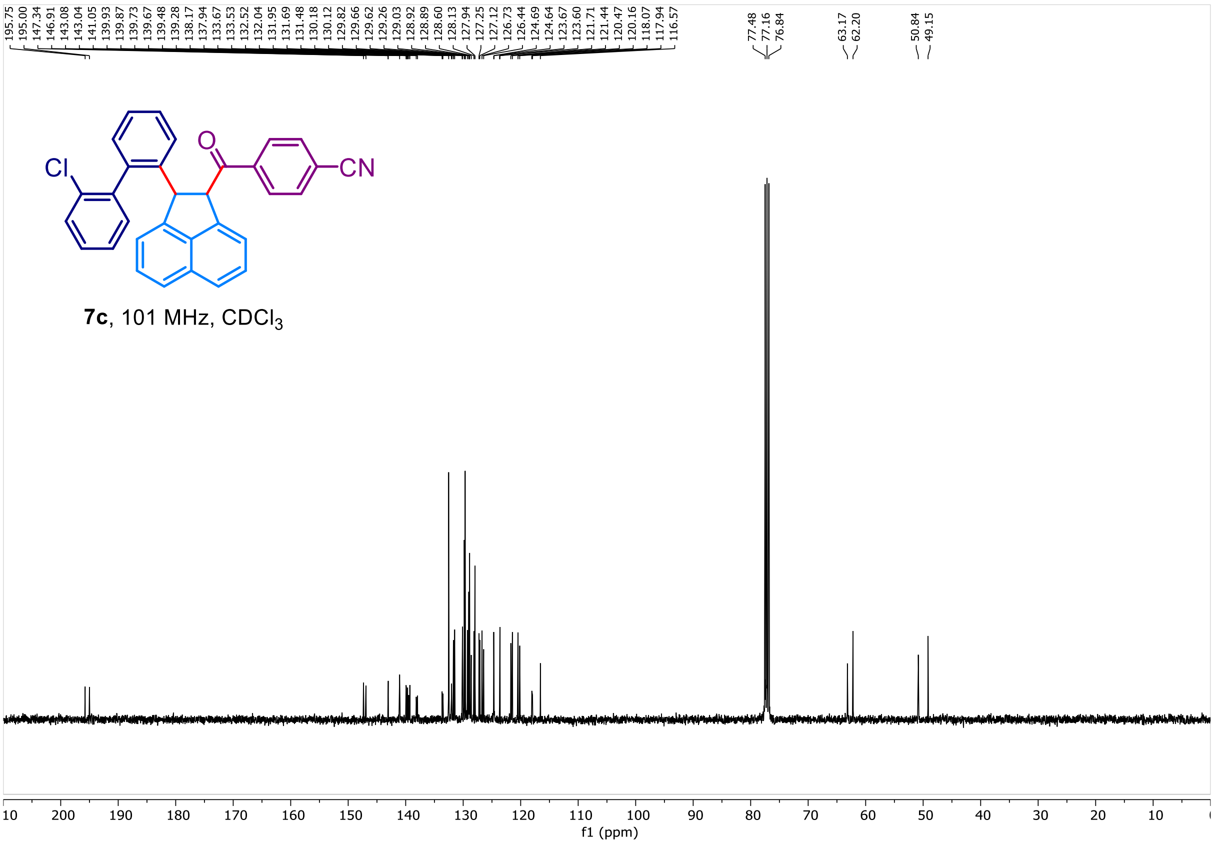

(2-(2'-chloro-[1,1'-biphenyl]-2-yl)-1,2-dihydroacenaphthylen-1-yl)(p-tolyl)methanone (7d)

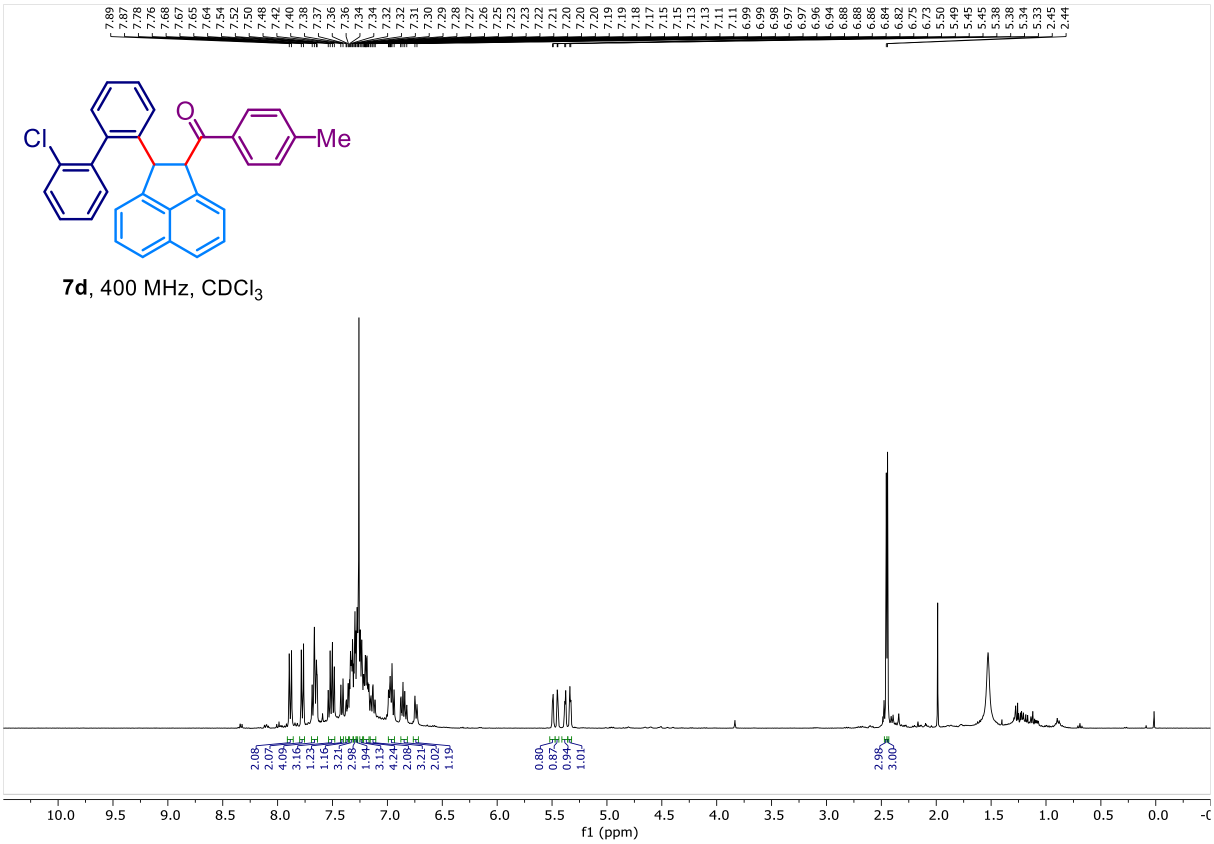

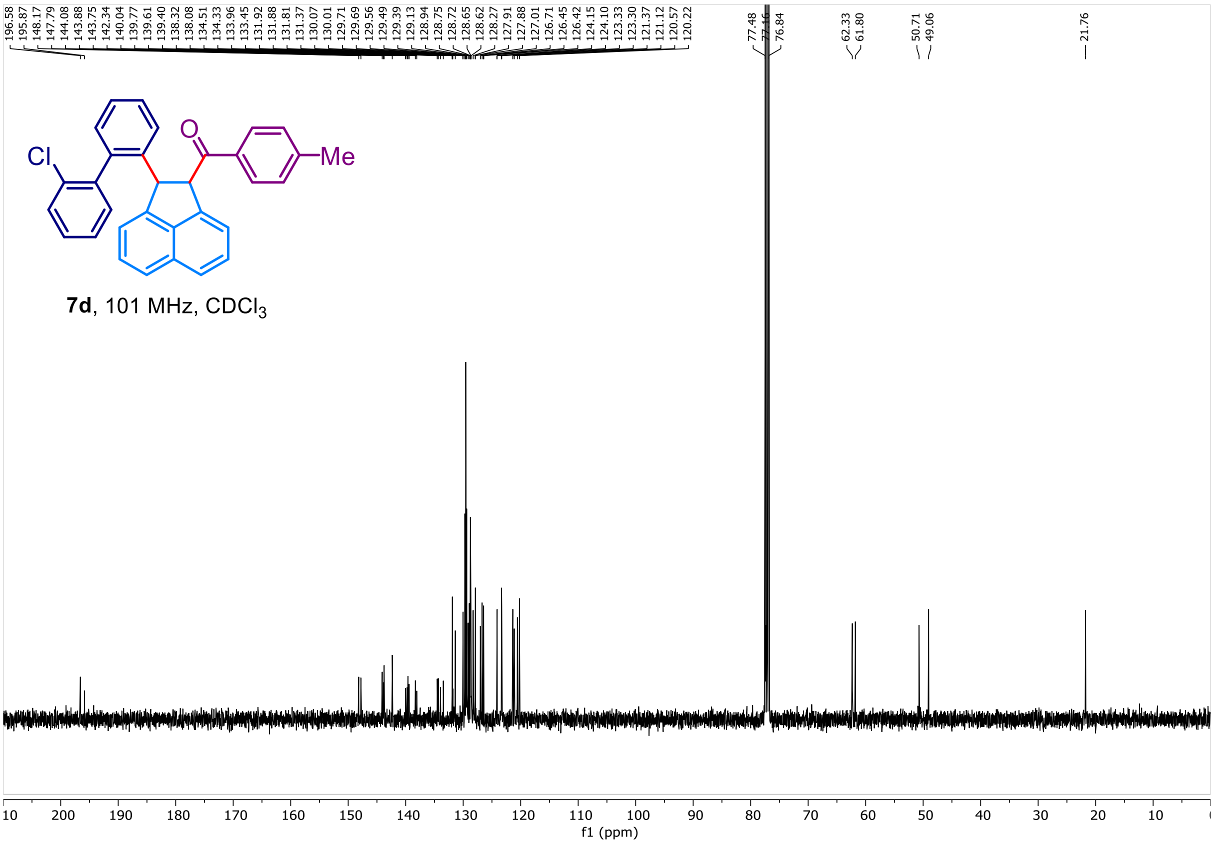

2-isopropyl-5-methylphenyl 4-(2-(2'-chloro-[1,1'-biphenyl]-2-yl)-1,2-dihydroacenaphthylene-1-carbonyl)benzoate (7e)

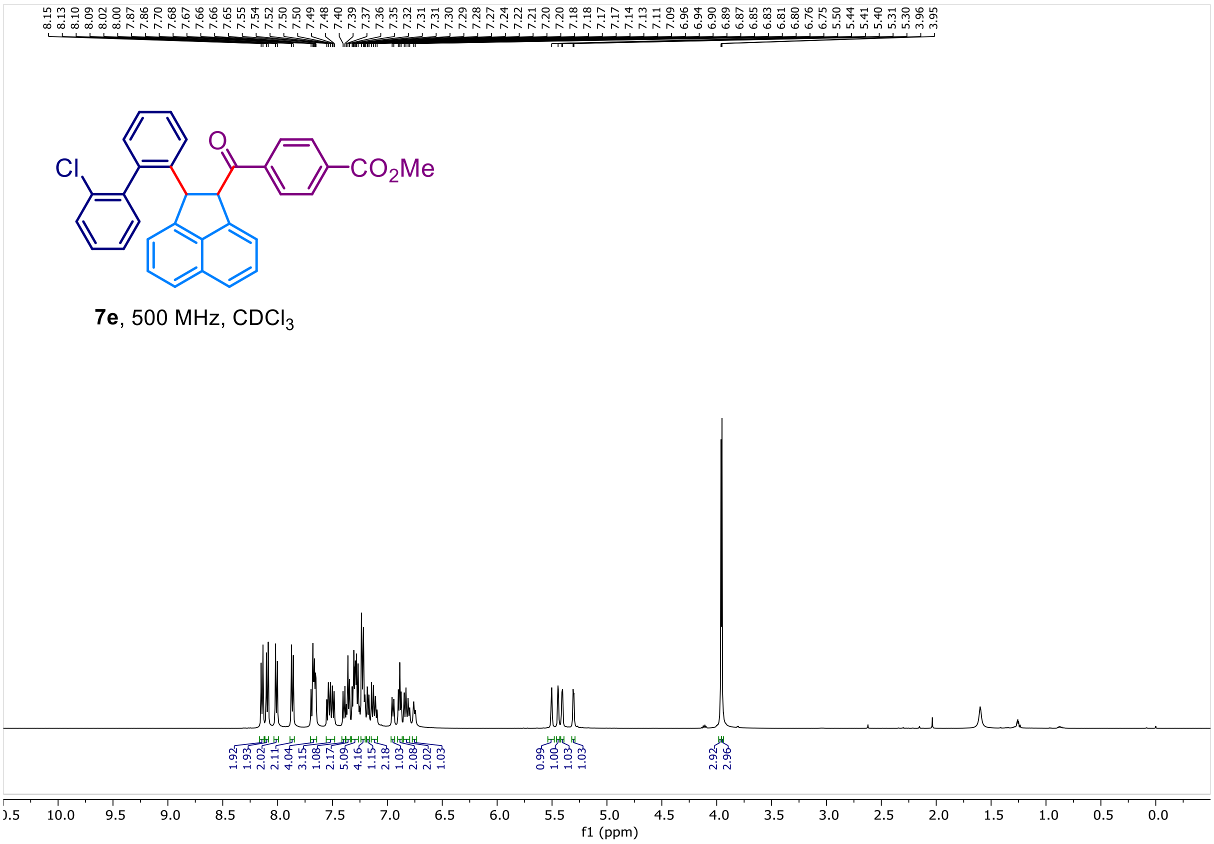

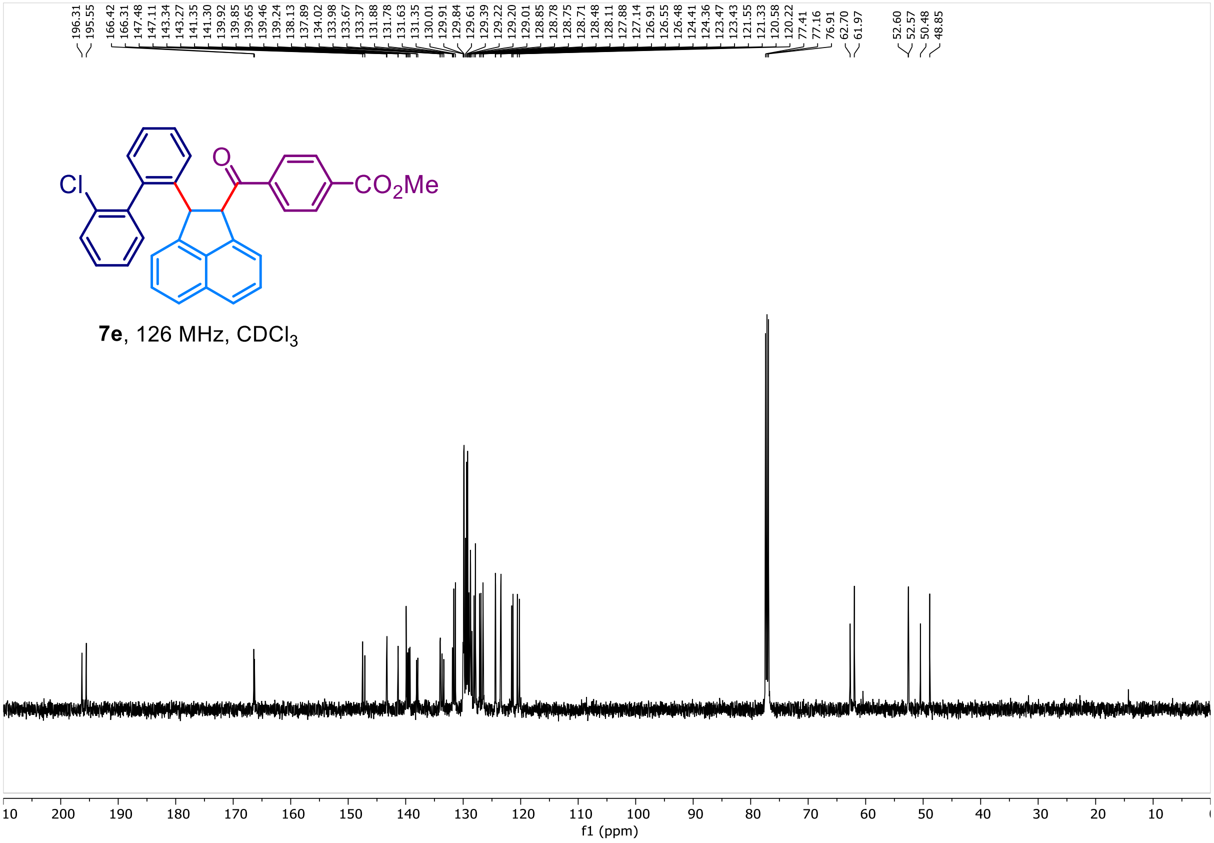

(2-(2'-chloro-[1,1'-biphenyl]-2-yl)-1,2-dihydroacenaphthylen-1-yl)(naphthalen-2-yl)methanone (7f)

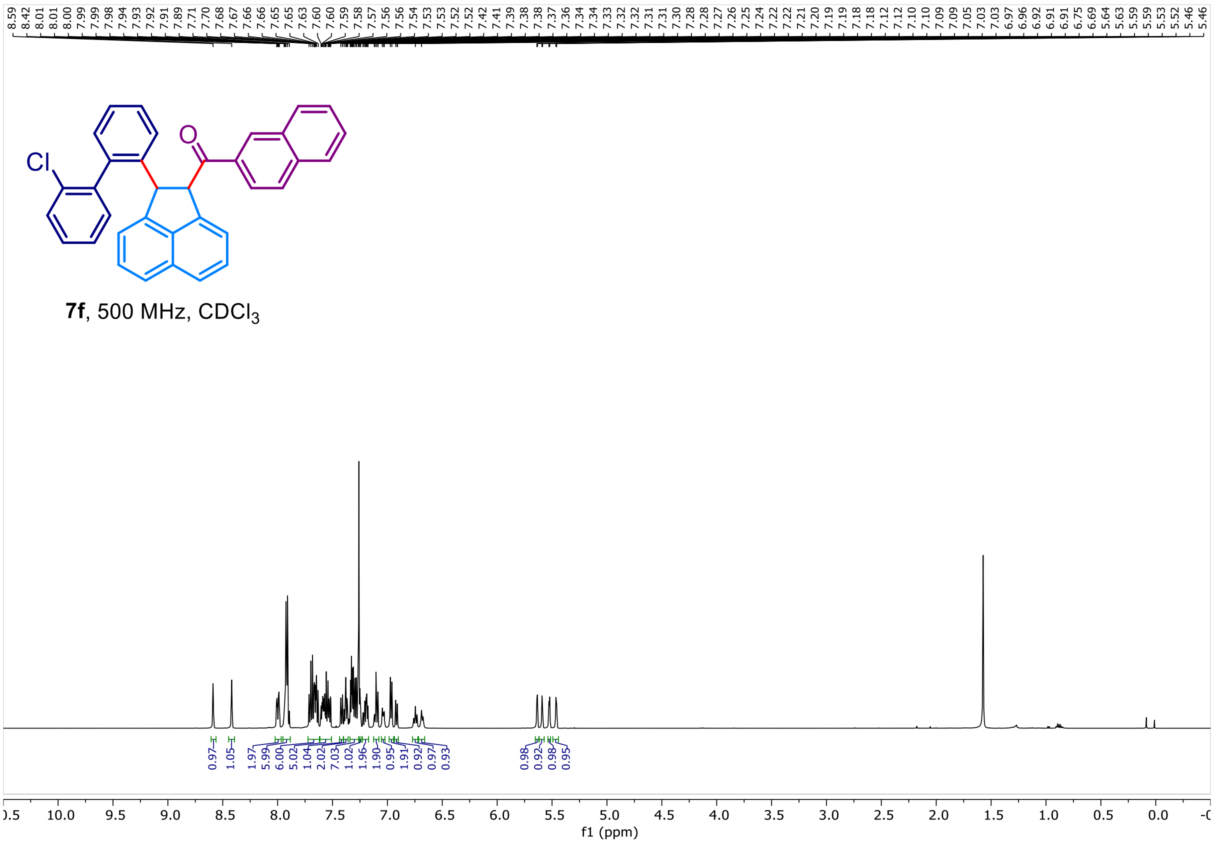

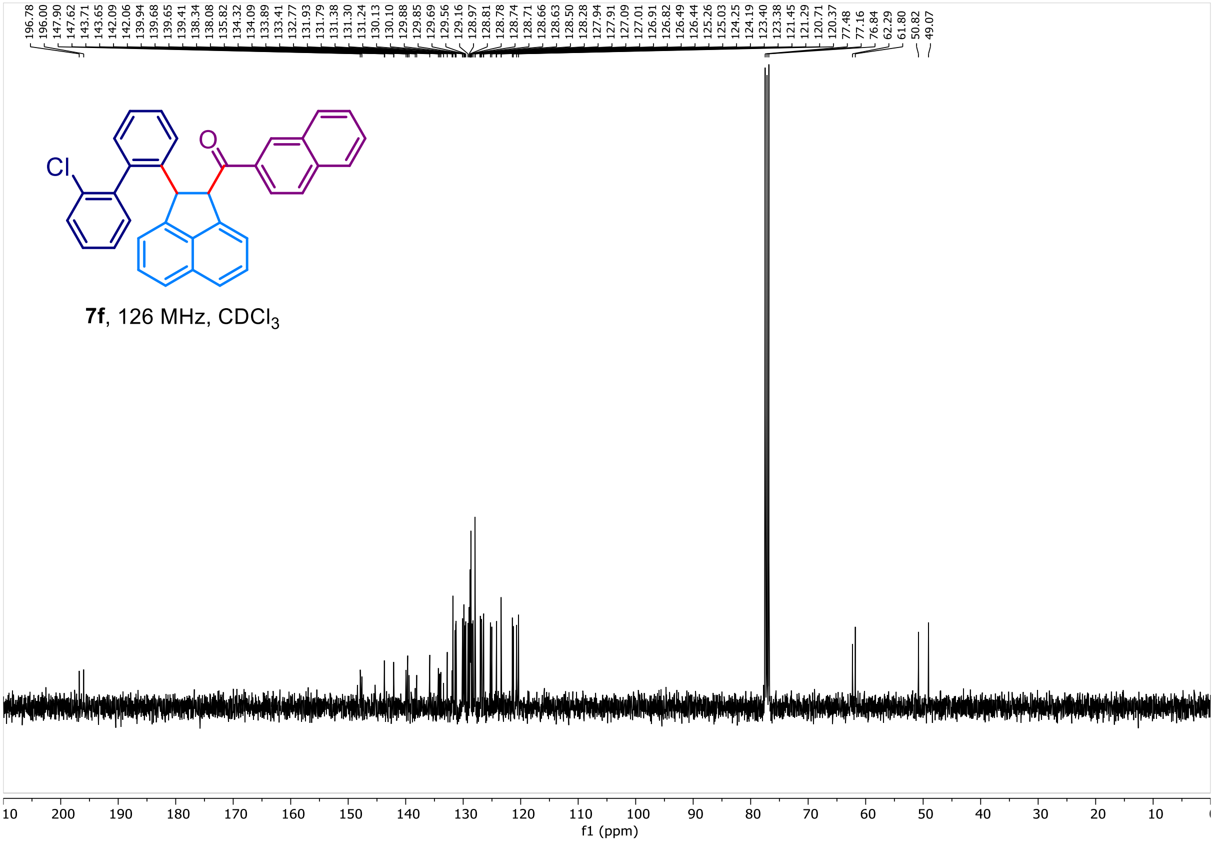

(2-(2'-chloro-[1,1'-biphenyl]-2-yl)-1,2-dihydroacenaphthylen-1-yl)(pyridin-2-yl)methanone(7g)

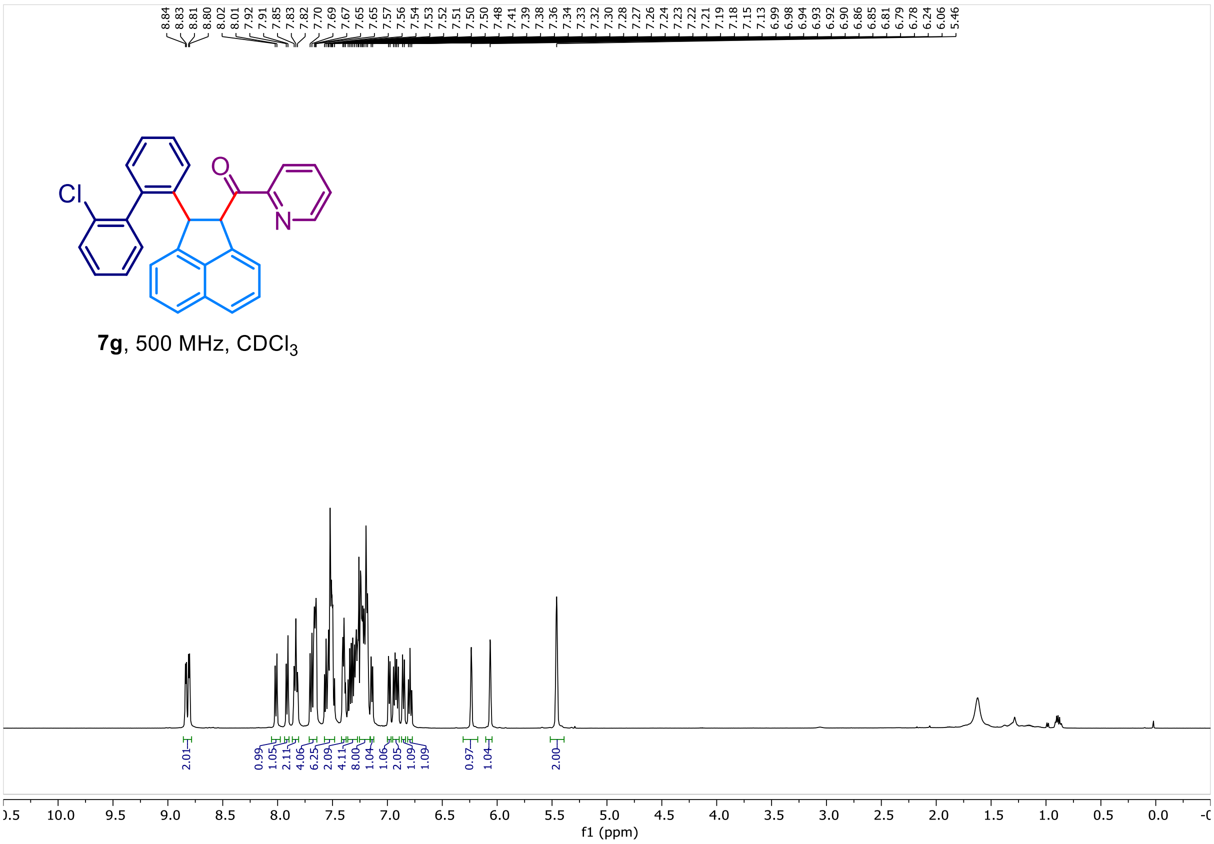

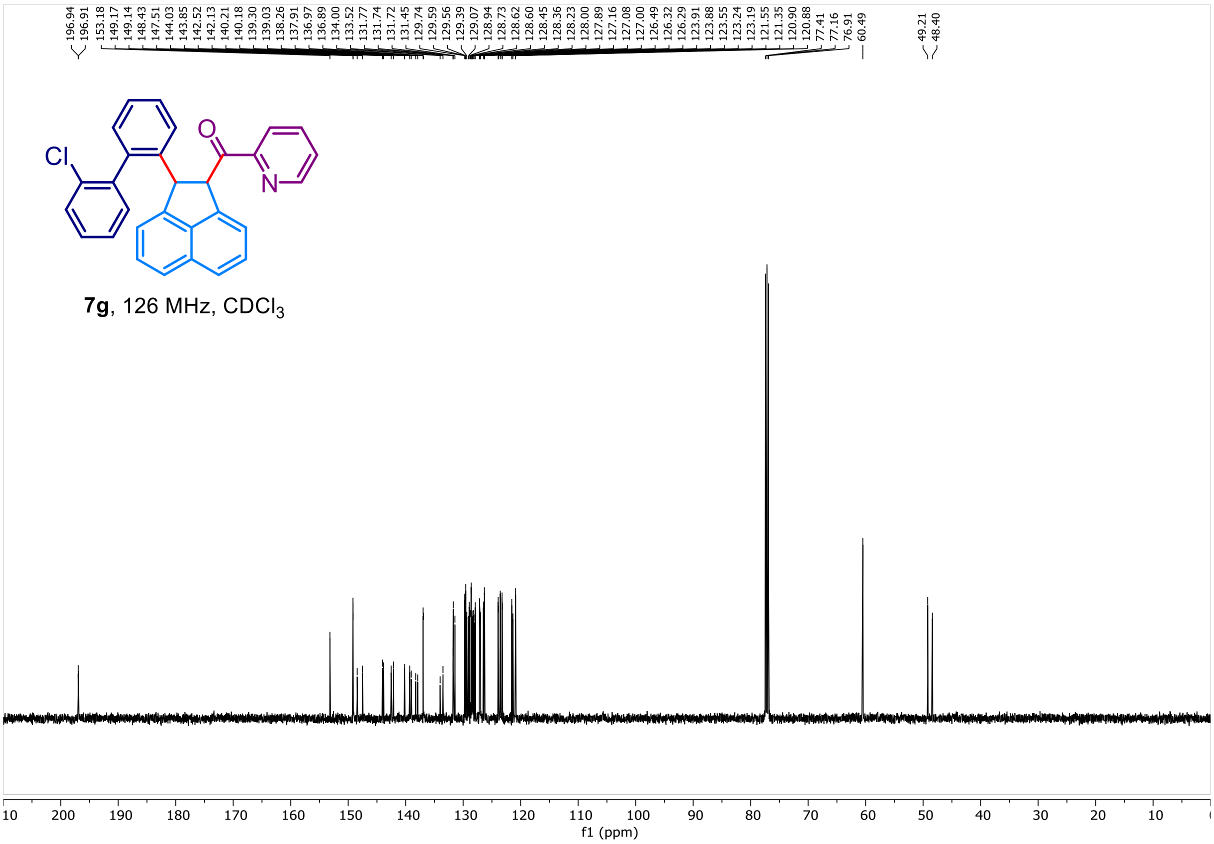

(2-(2'-chloro-[1,1'-biphenyl]-2-yl)-1,2-dihydroacenaphthylen-1-yl)(quinolin-2-yl)methanone(7h)

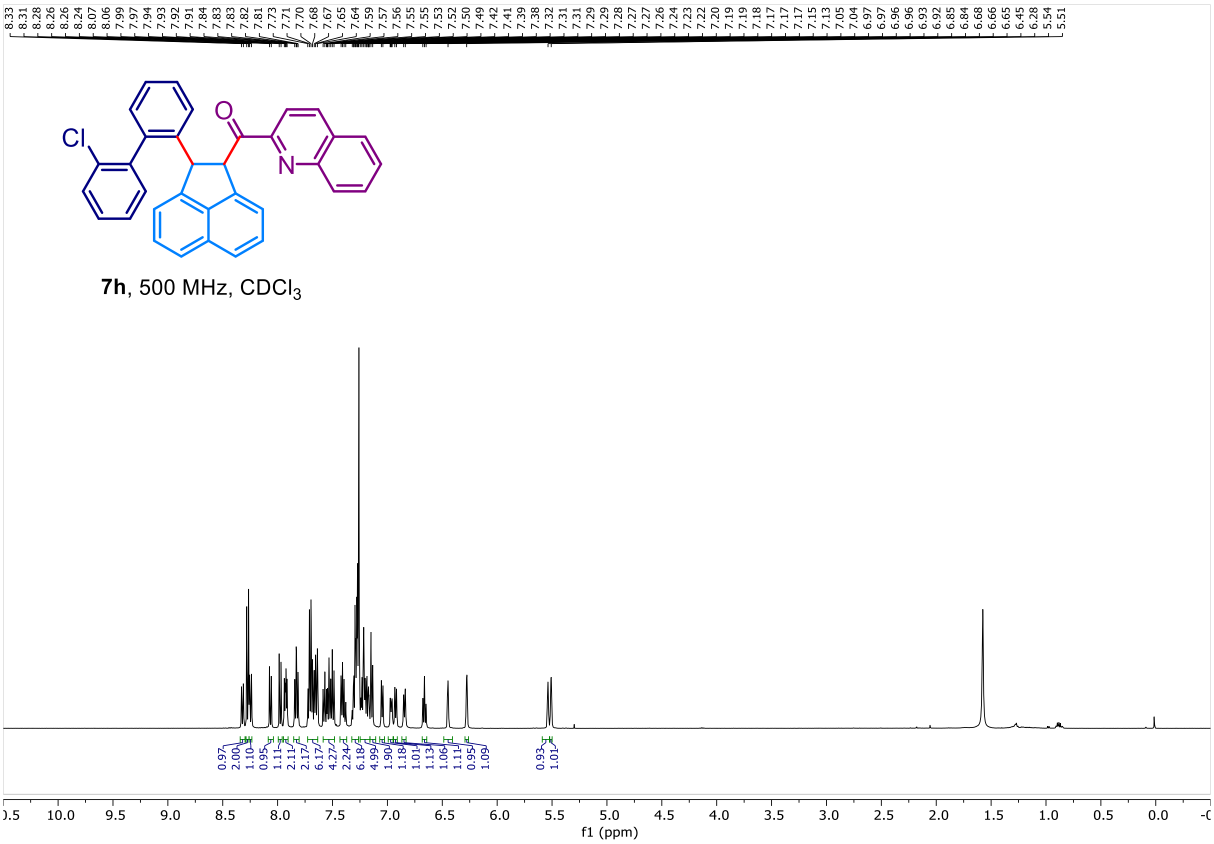

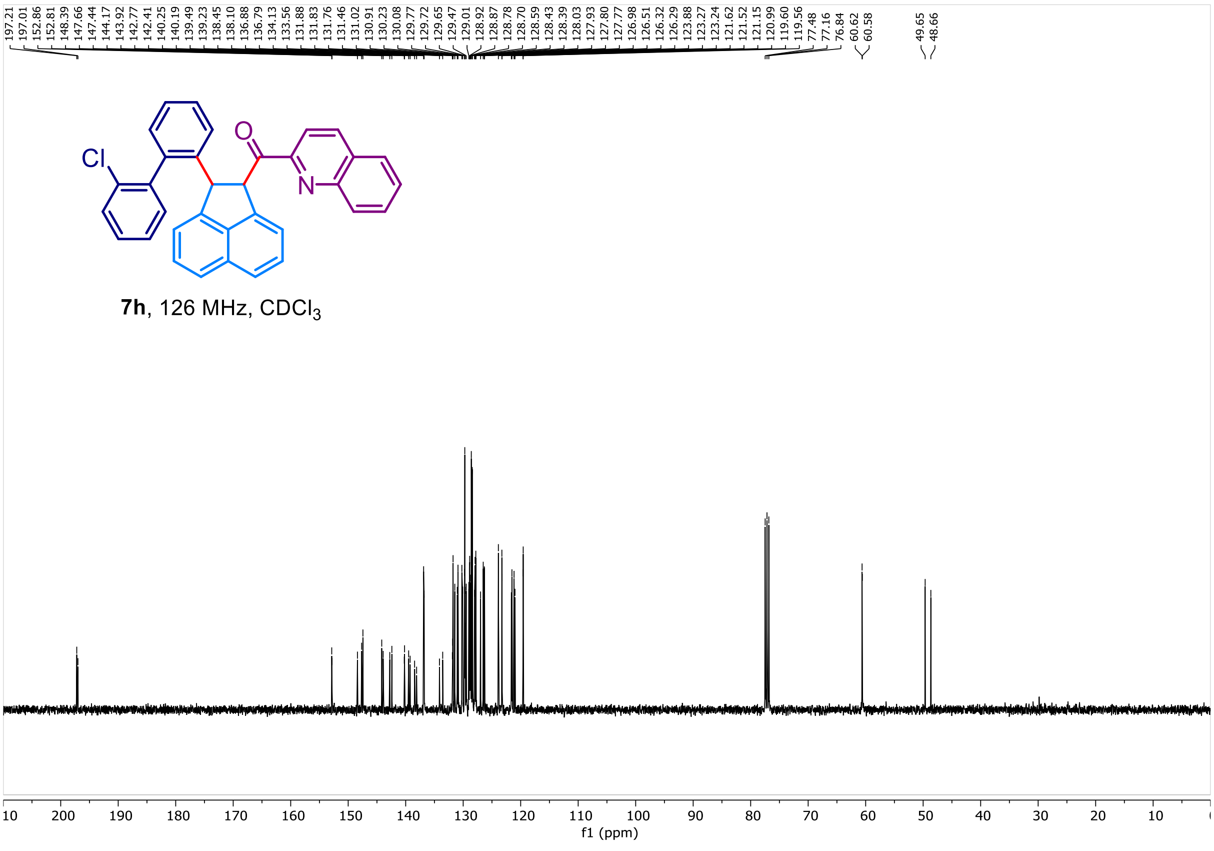

(2-(2'-chloro-[1,1'-biphenyl]-2-yl)-1,2-dihydroacenaphthylen-1-yl)(thiophen-2-yl)methanone (7i)

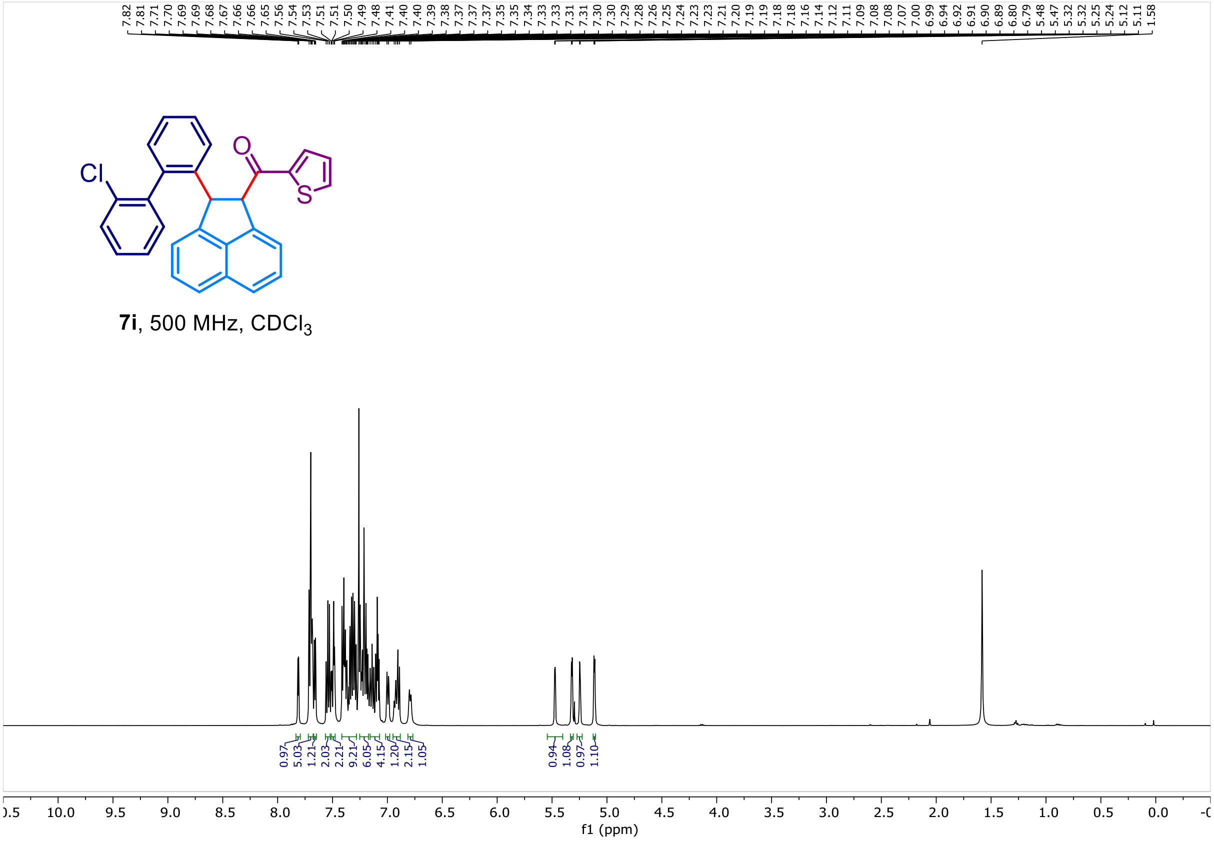

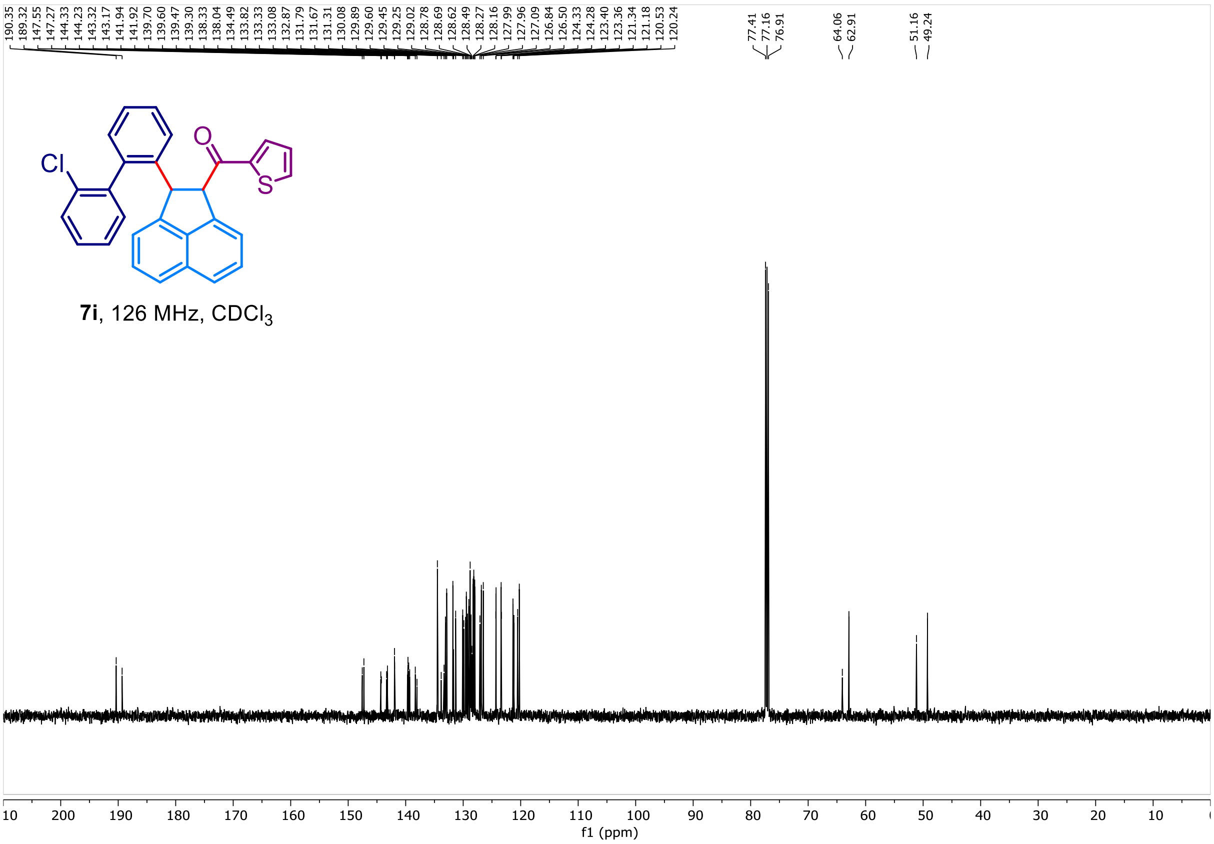

(4-bromophenyl)(2-(2'-chloro-[1,1'-biphenyl]-2-yl)-2,3-dihydro-1H-inden-1-yl)methanone (7j)

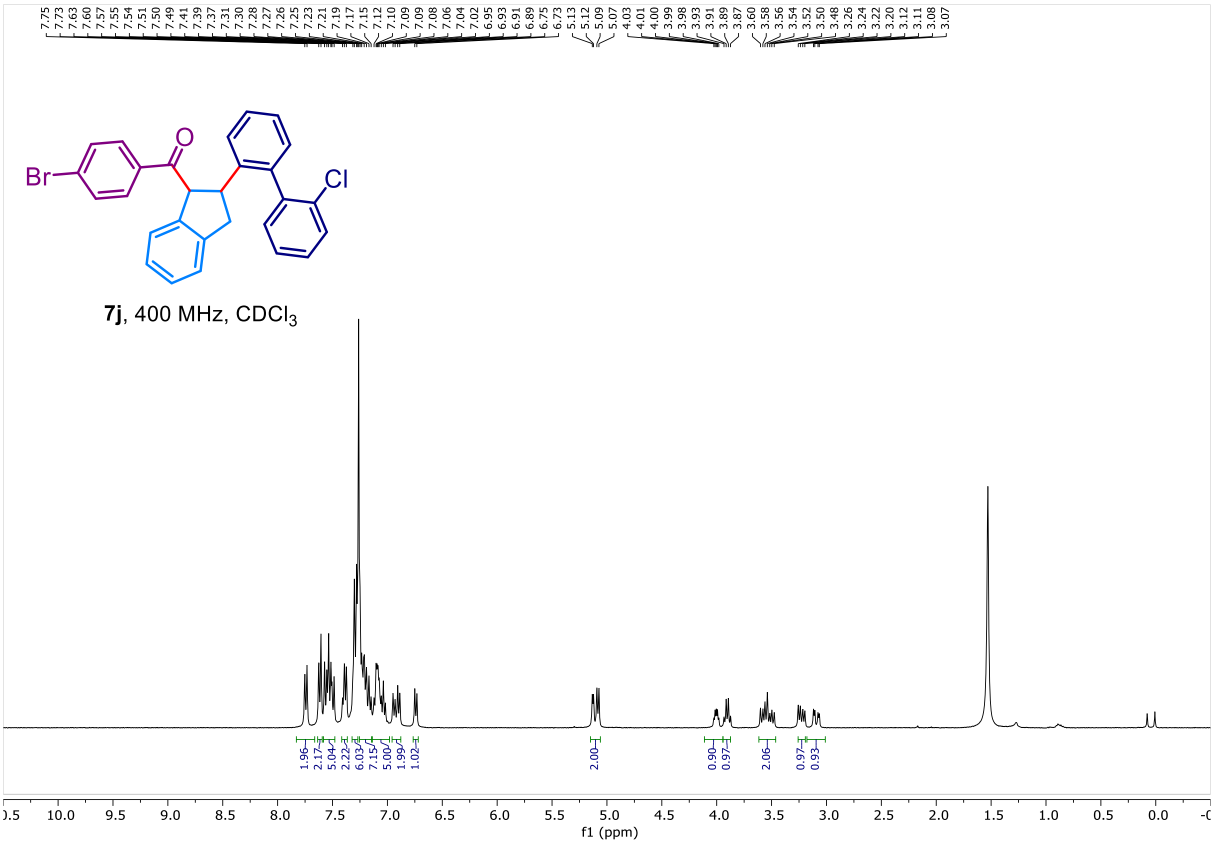

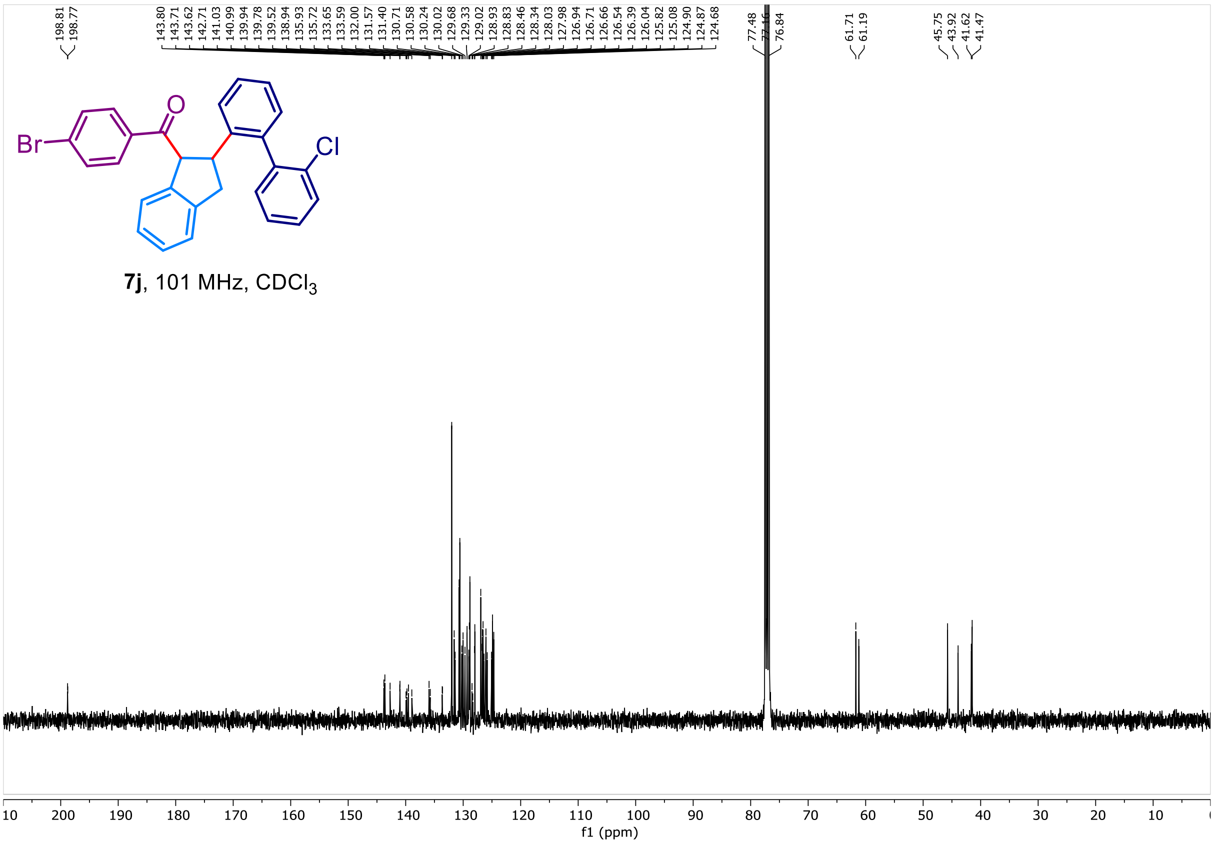

2-isopropyl-5-methylphenyl-4-(3-(2'-chloro-[1,1'-biphenyl]-2-yl)-2-phenylpropanoyl)benzoate (8a)

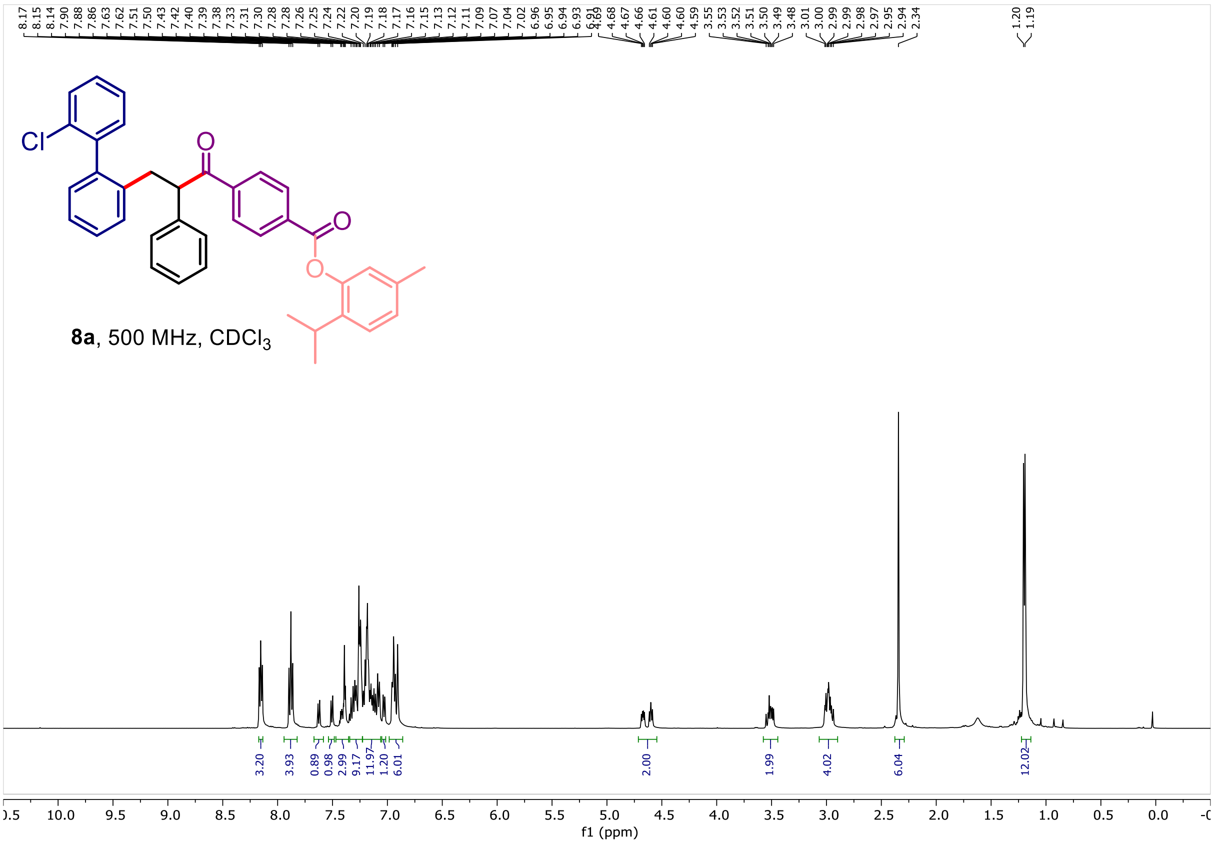

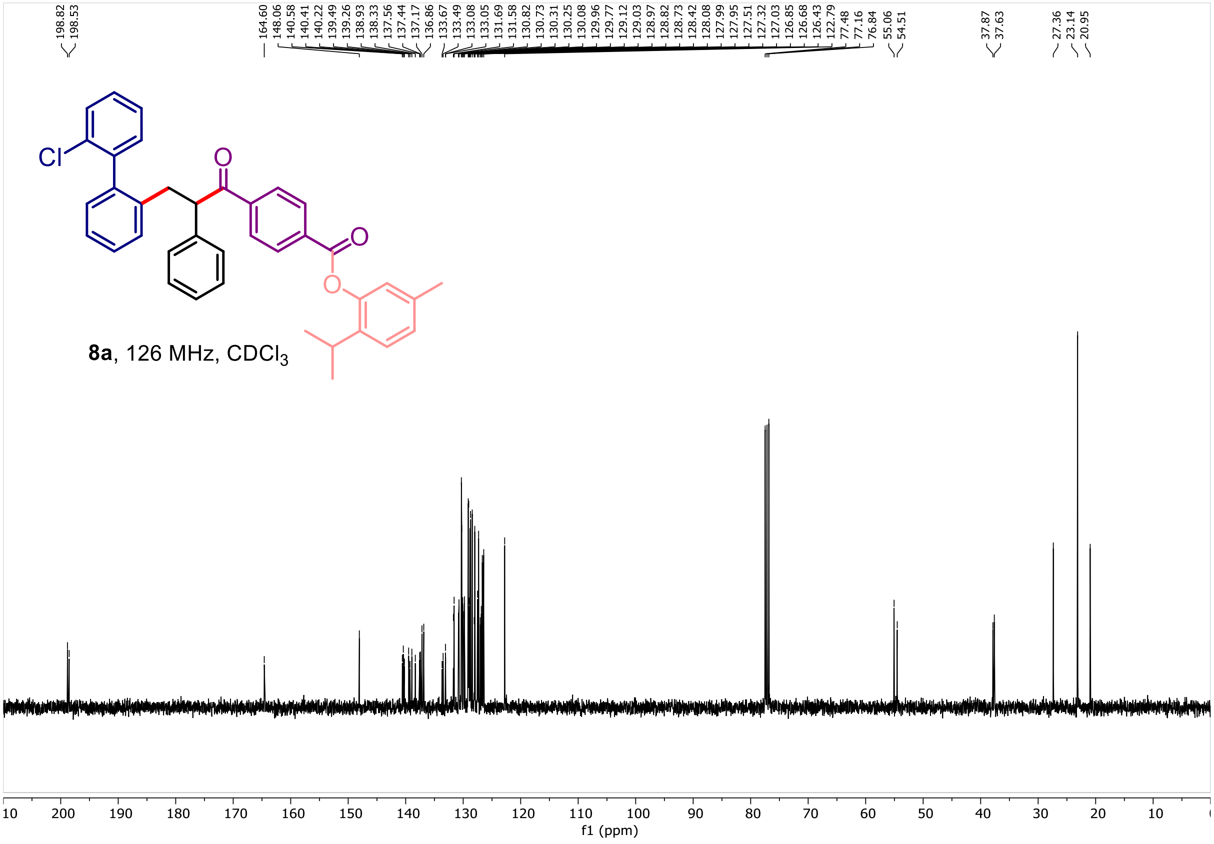

2-isopropyl-5-methylcyclohexyl 4-((R)-3-(2'-chloro-[1,1'-biphenyl]-2-yl)-2-phenylpropanoyl)benzoate(8b)

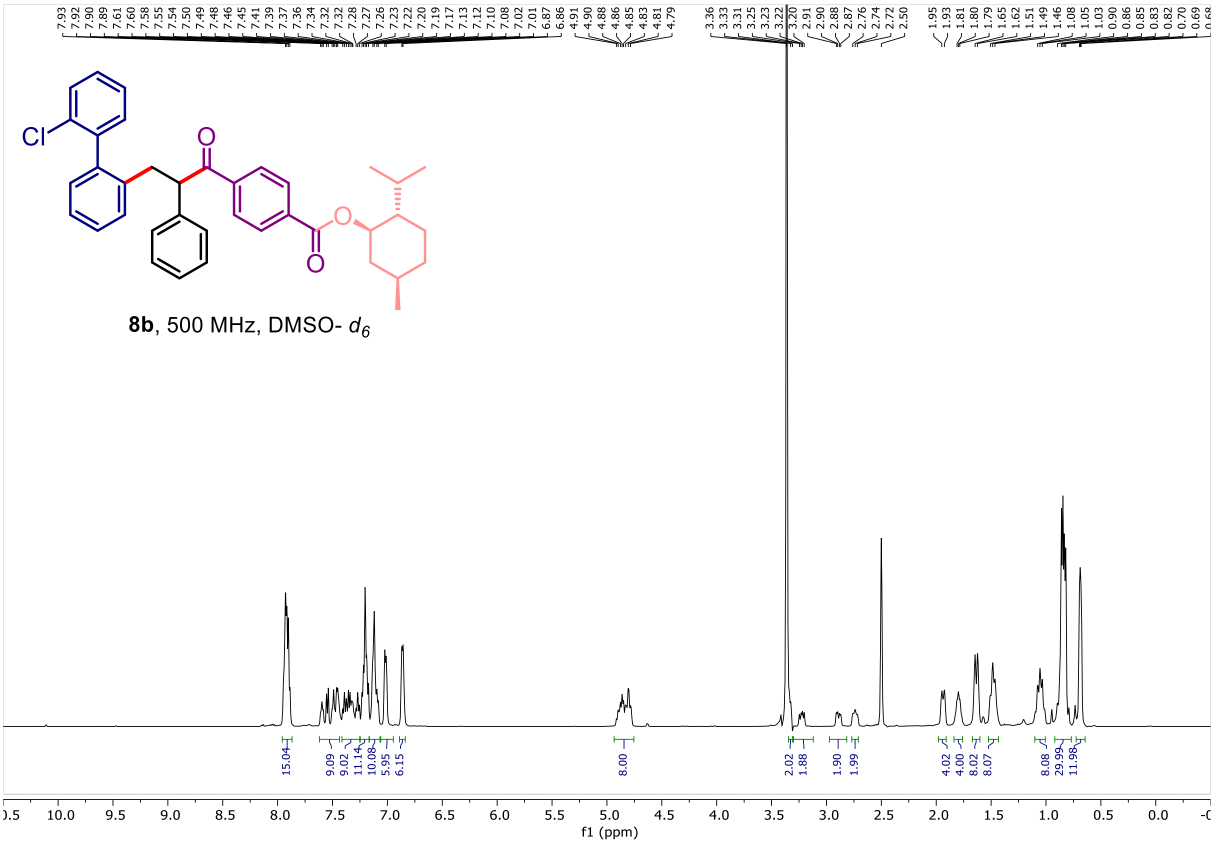

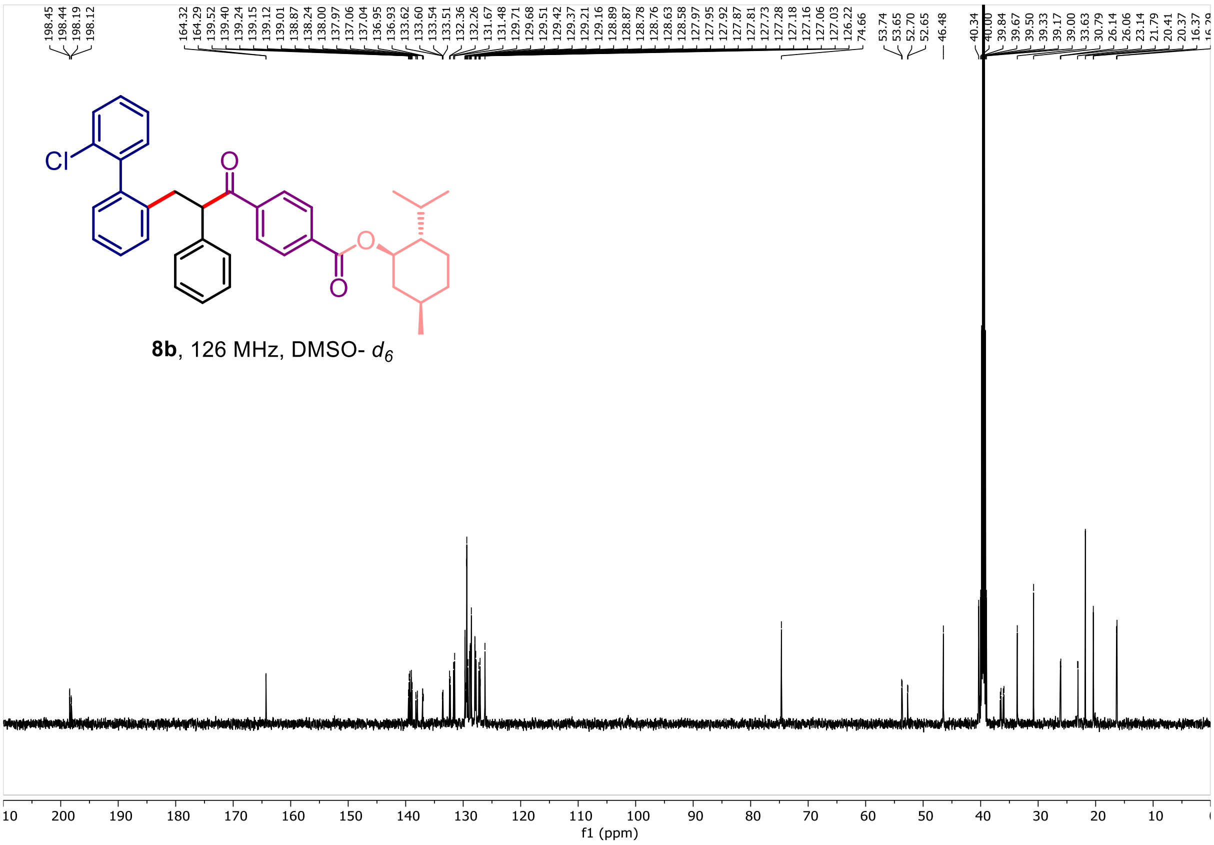

3,7-dimethyloct-6-en-1-yl 4-(-3-(2'-chloro-[1,1'-biphenyl]-2-yl)-2-phenylpropanoyl)benzoate (8c)

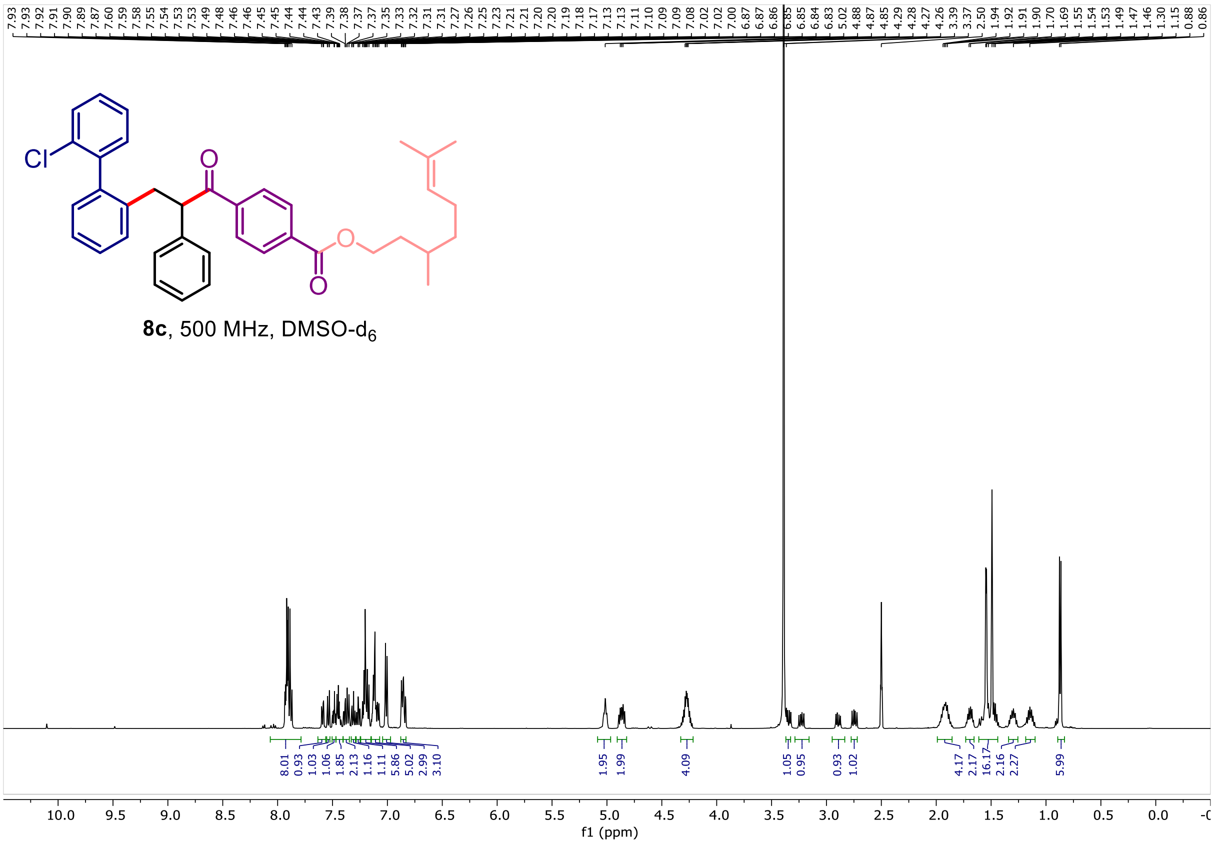

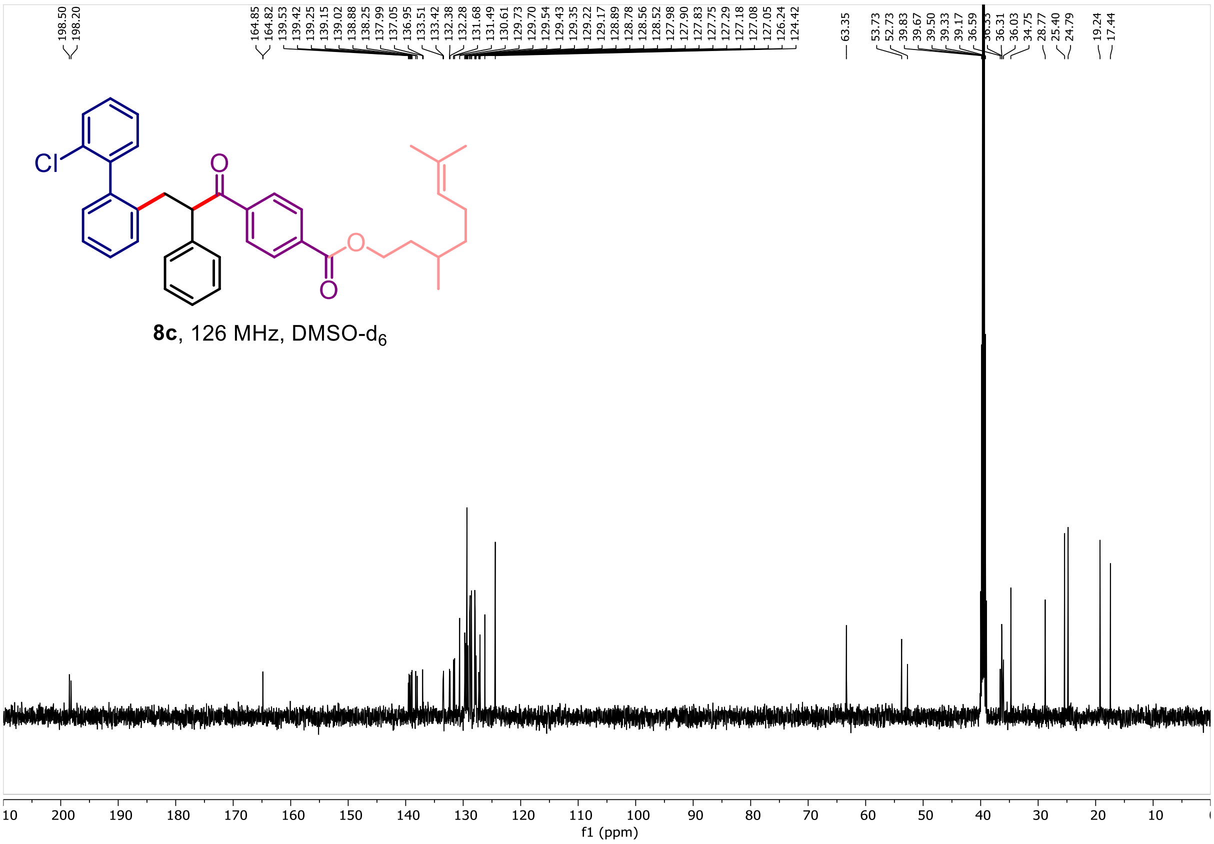

2-oxo-2H-chromen-7-yl-4-(3-(2'-chloro-[1,1'-biphenyl]-2-yl)-2-phenylpropanoyl)benzoate(8d)

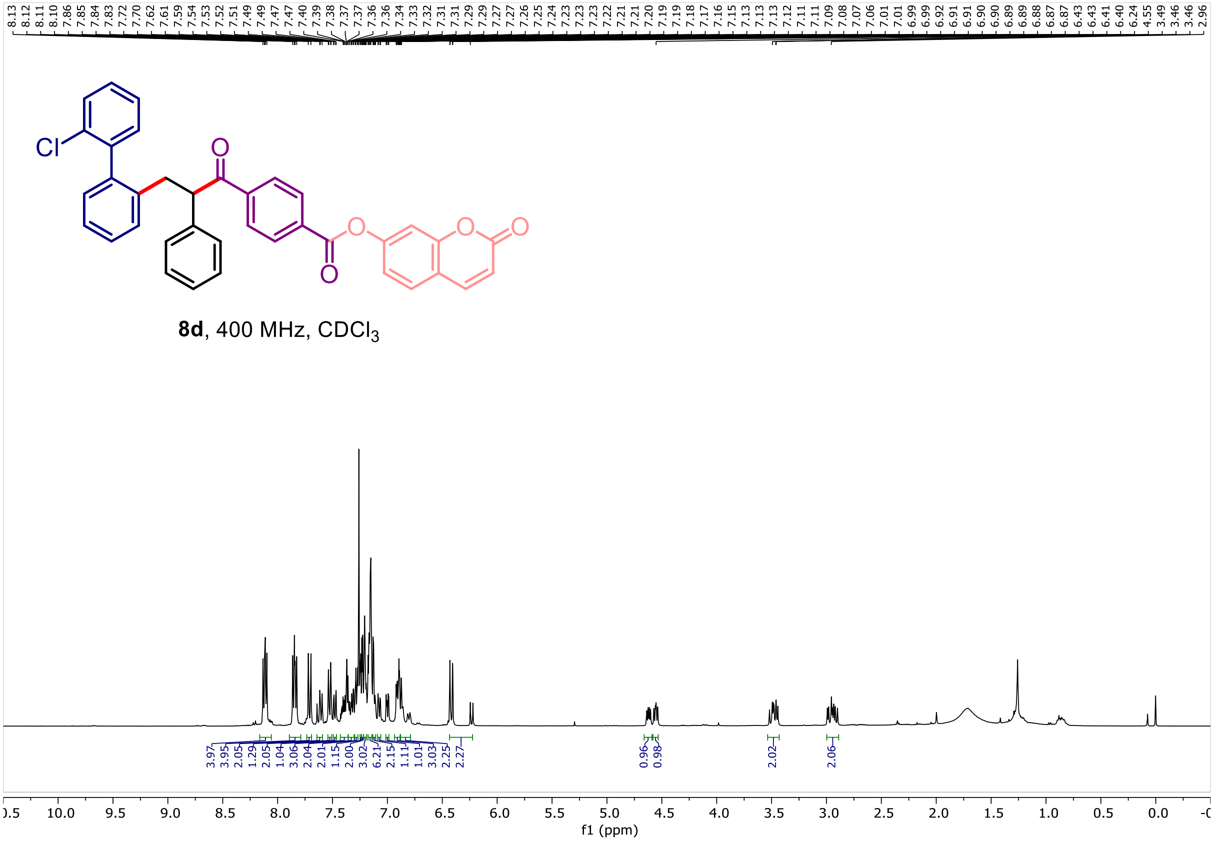

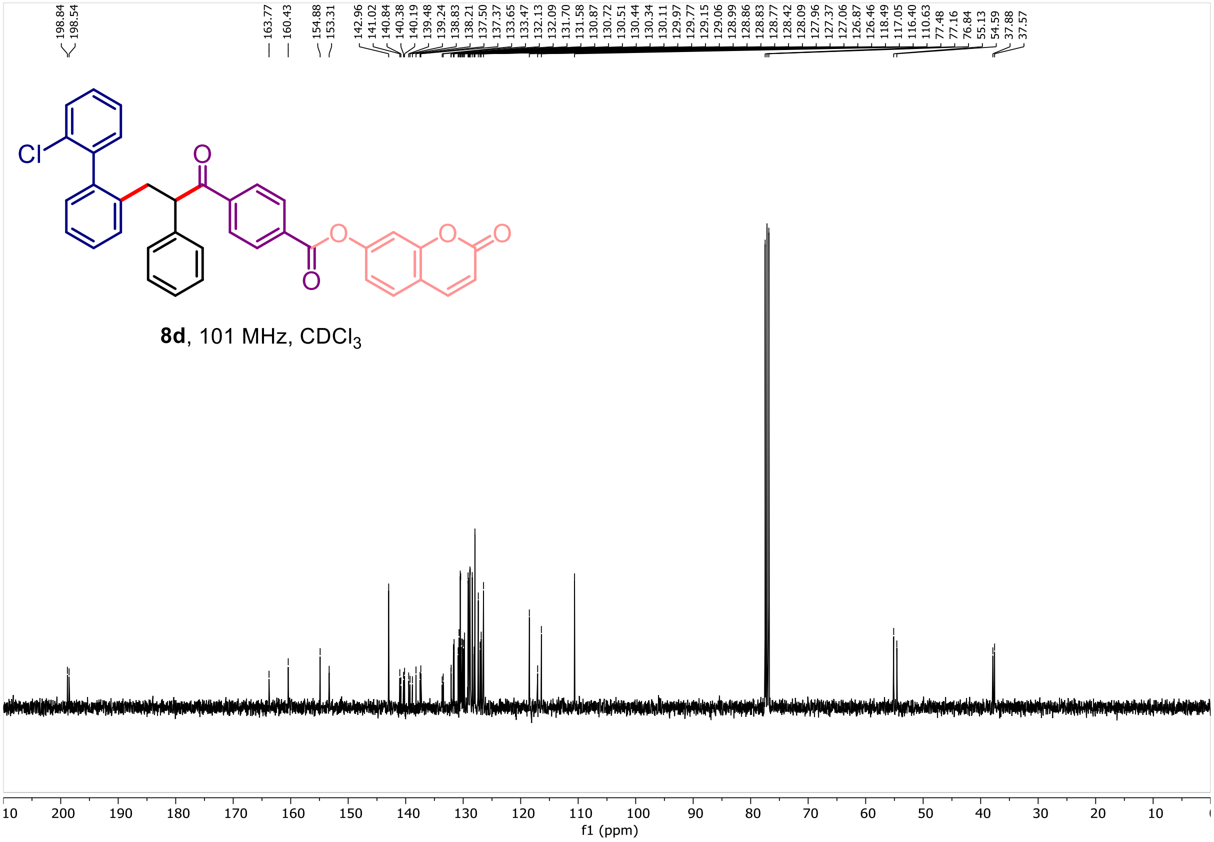

((3aR,5R,5aS,8aS,8bR)-2,2,7,7-tetramethyltetrahydro-5H-bis([1,3]dioxolo)[4,5-b:4',5'-d]pyran-5-yl)methyl 4-(3-(2'-chloro-[1,1'-biphenyl]-2-yl)-2-phenylpropanoyl)benzoate(8e)

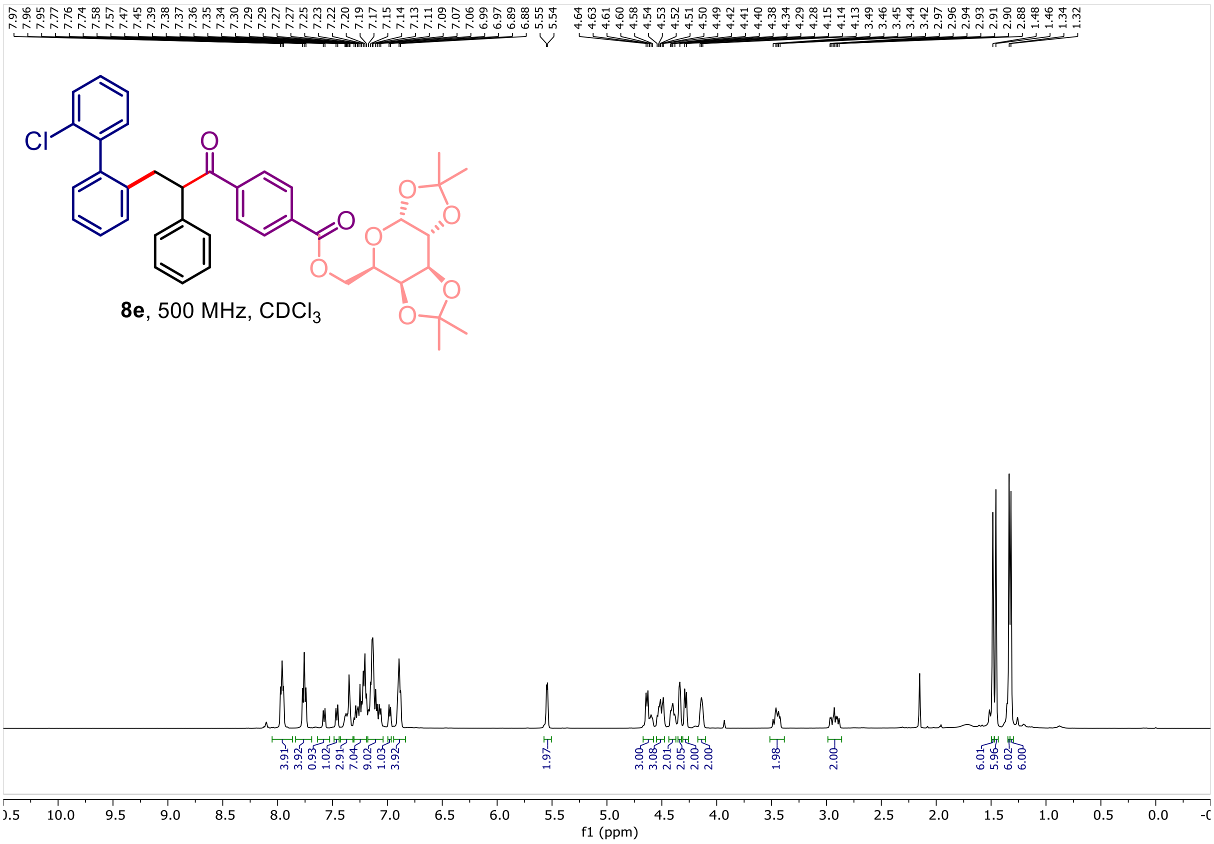

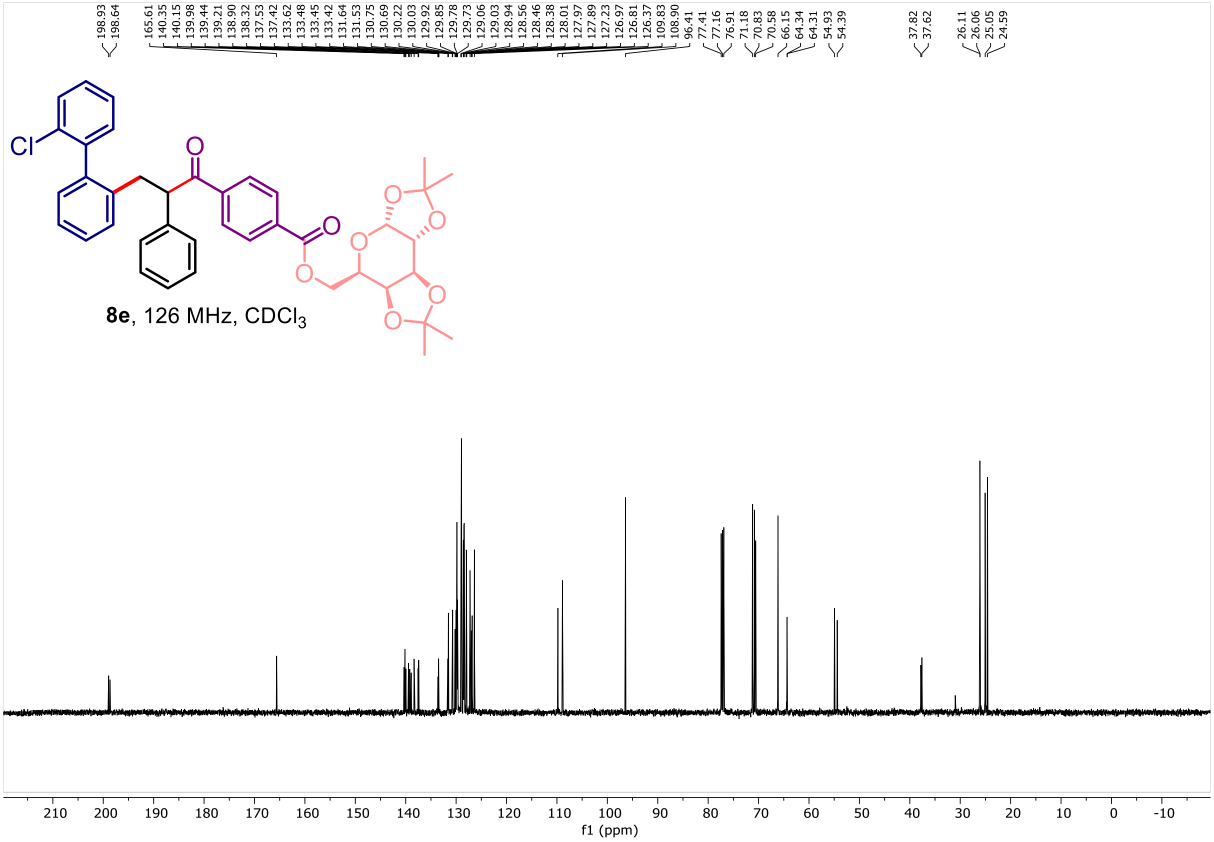

4-(-1-(4-bromophenyl)-3-(2'-chloro-[1,1'-biphenyl]-2-yl)-1-oxopropan-2-yl)phenyl 2-(4-isobutylphenyl)propanoate(8f)

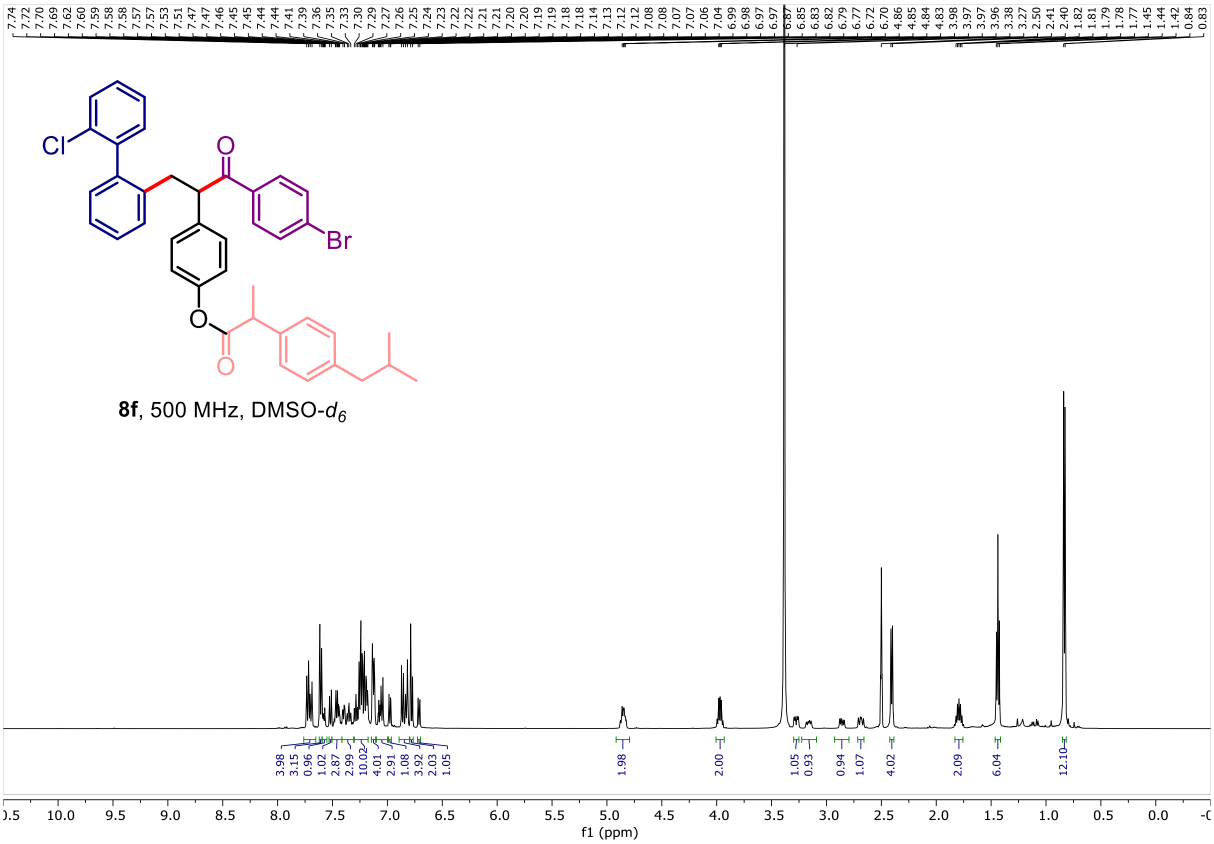

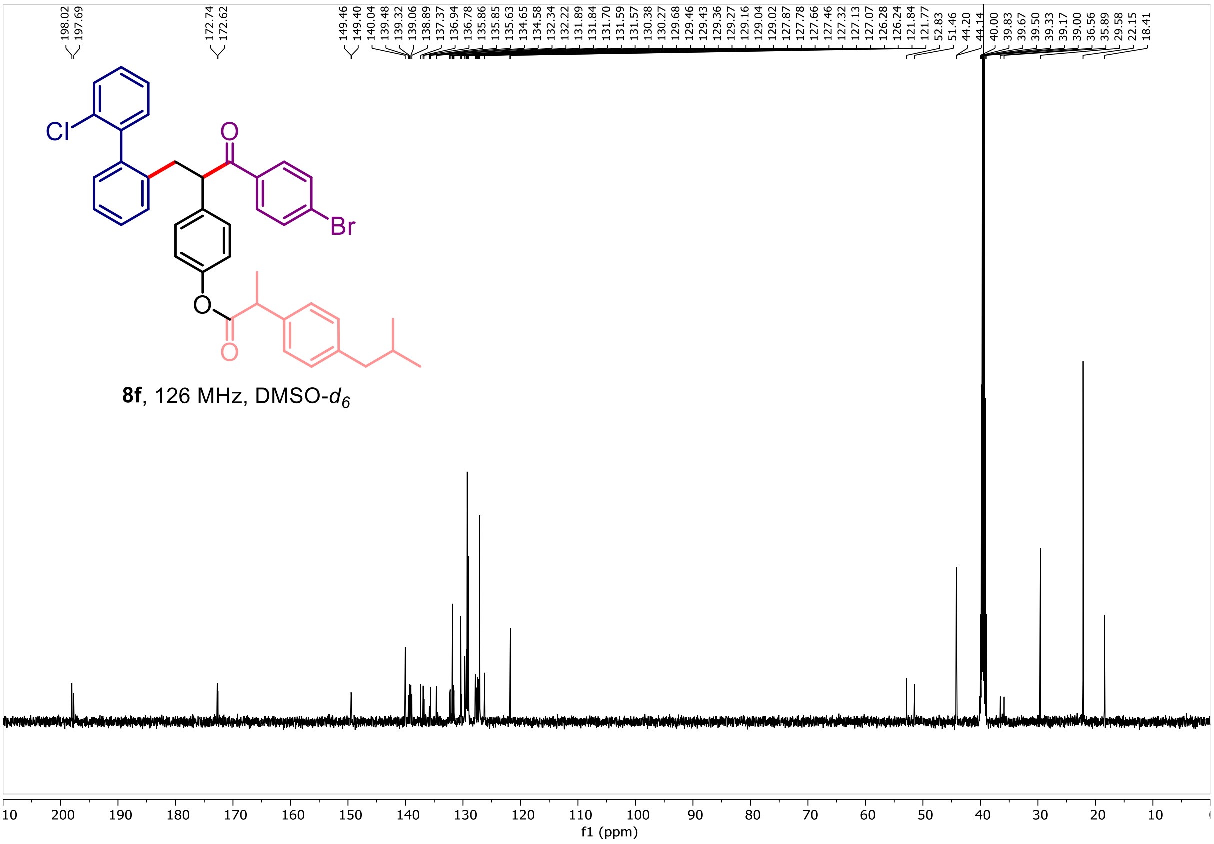

4-(1-(4-bromophenyl)-3-(2'-chloro-[1,1'-biphenyl]-2-yl)-1-oxopropan-2-yl)phenyl 2-(4-chlorophenoxy)-2-methylpropanoate(8g)

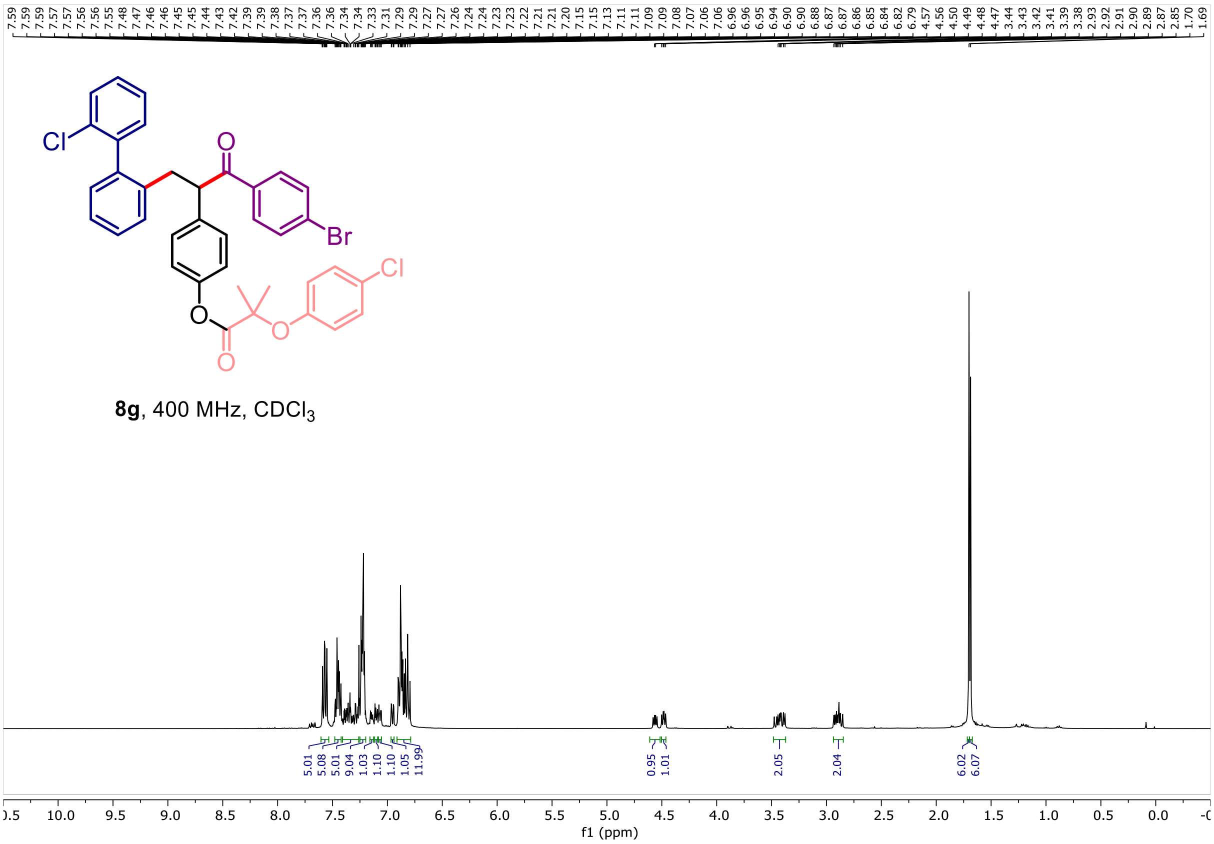

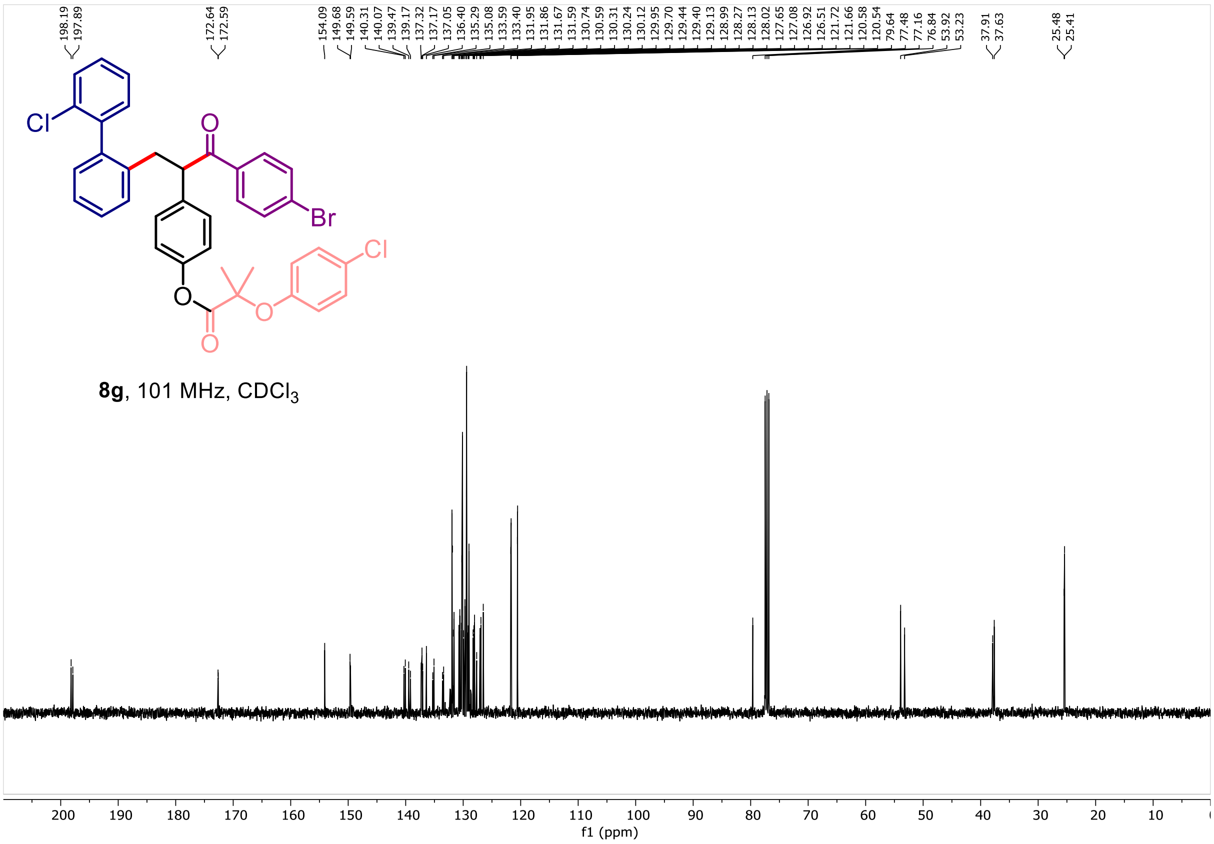

2-isopropyl-5-methylphenyl 4-(2-(2'-chloro-[1,1'-biphenyl]-2-yl)-1,2-dihydroacenaphthylene-1-carbonyl)benzoate (8h)

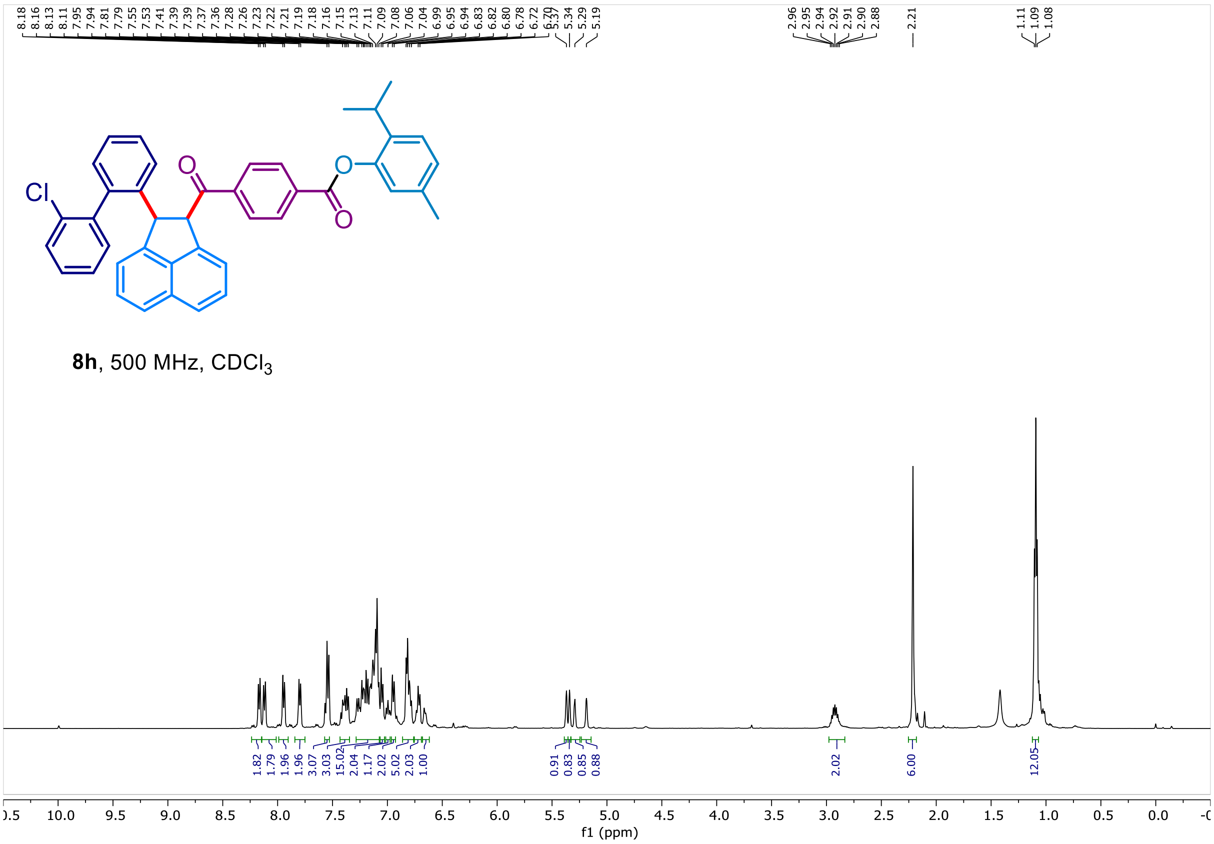

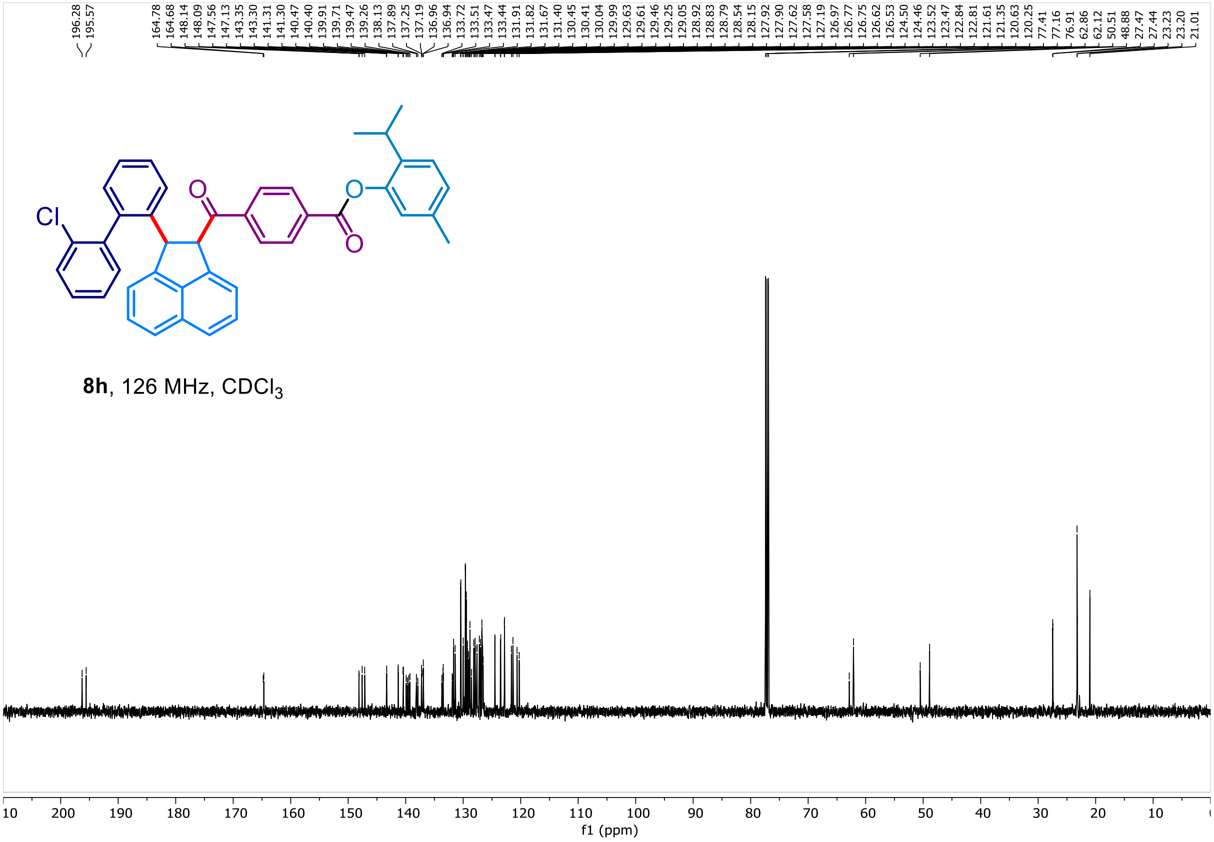

3-(4-bromophenyl)-5-(2'-chloro-[1,1'-biphenyl]-2-yl)-4-phenylisoxazole (9)

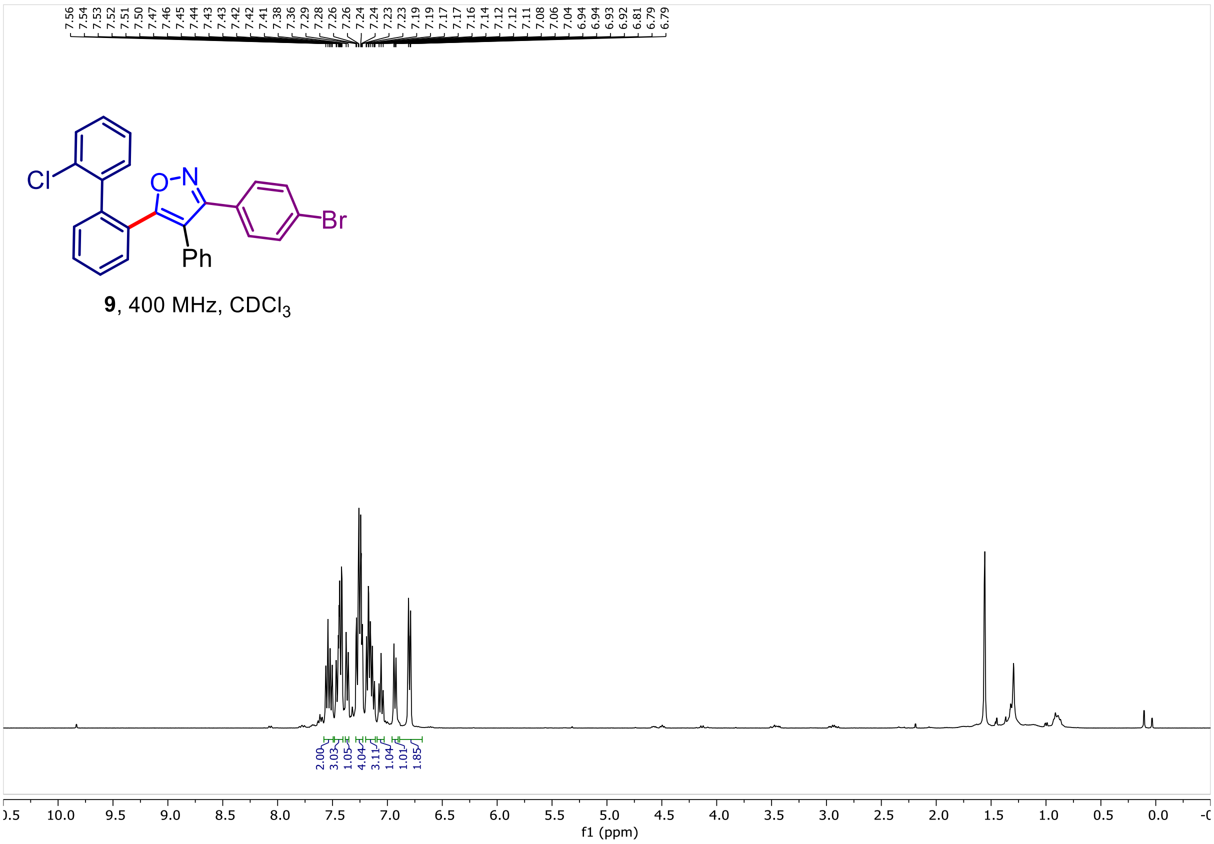

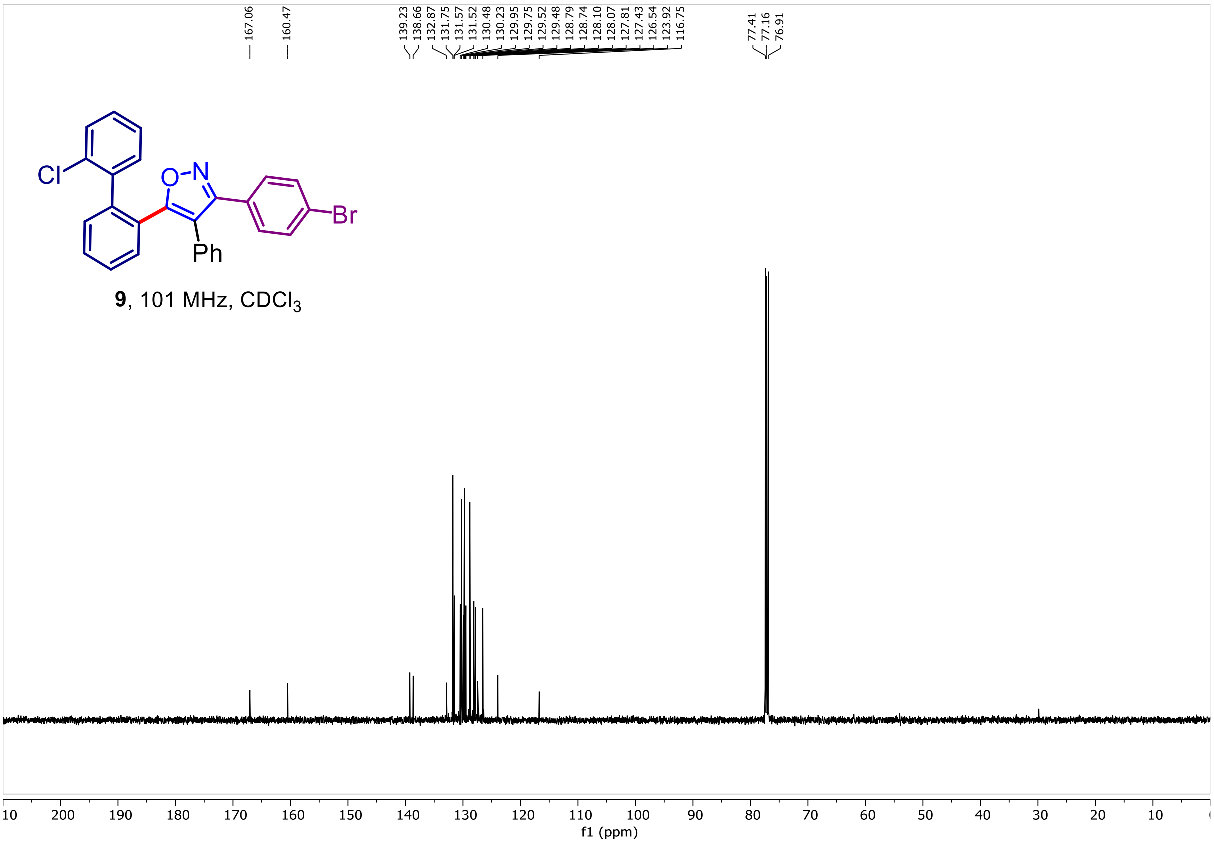

1-([1,1'-biphenyl]-4-yl)-3-(2'-chloro-[1,1'-biphenyl]-2-yl)-2-phenylpropan-1-one(10)

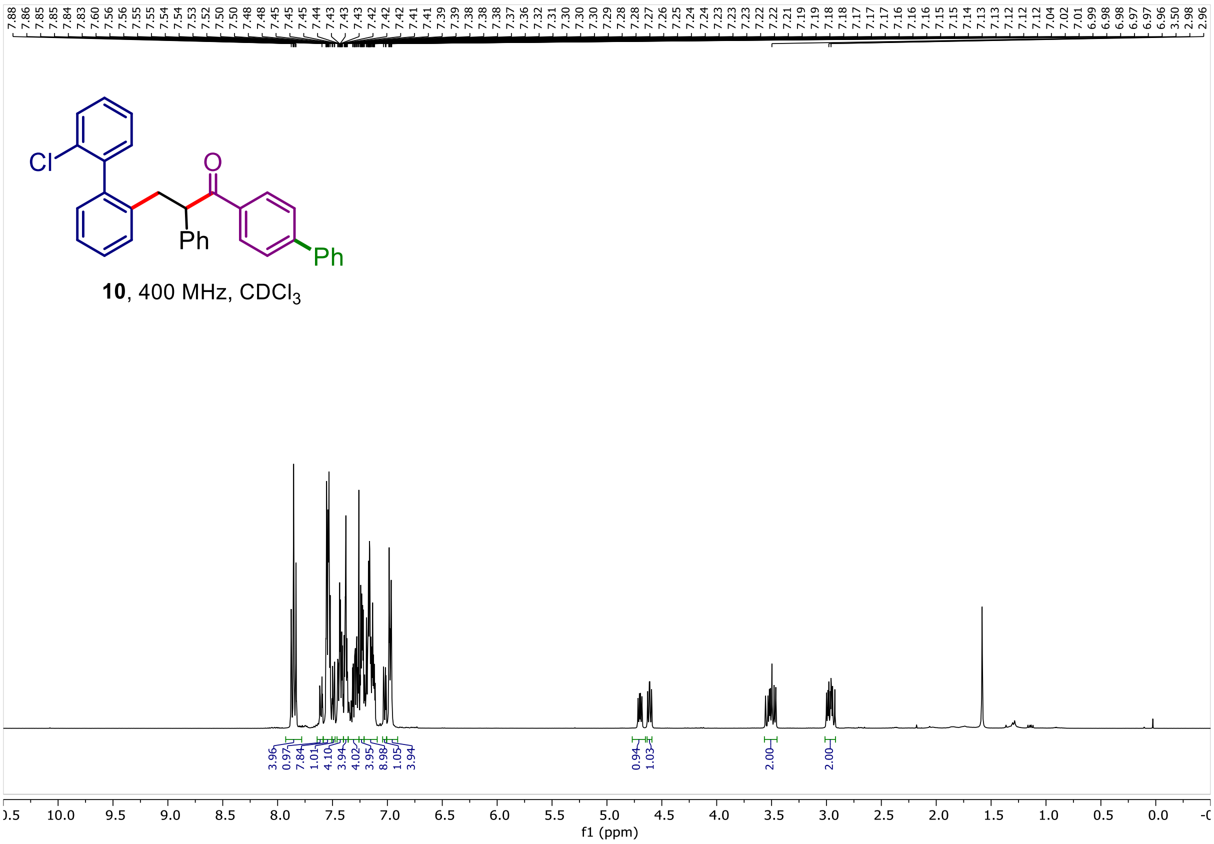

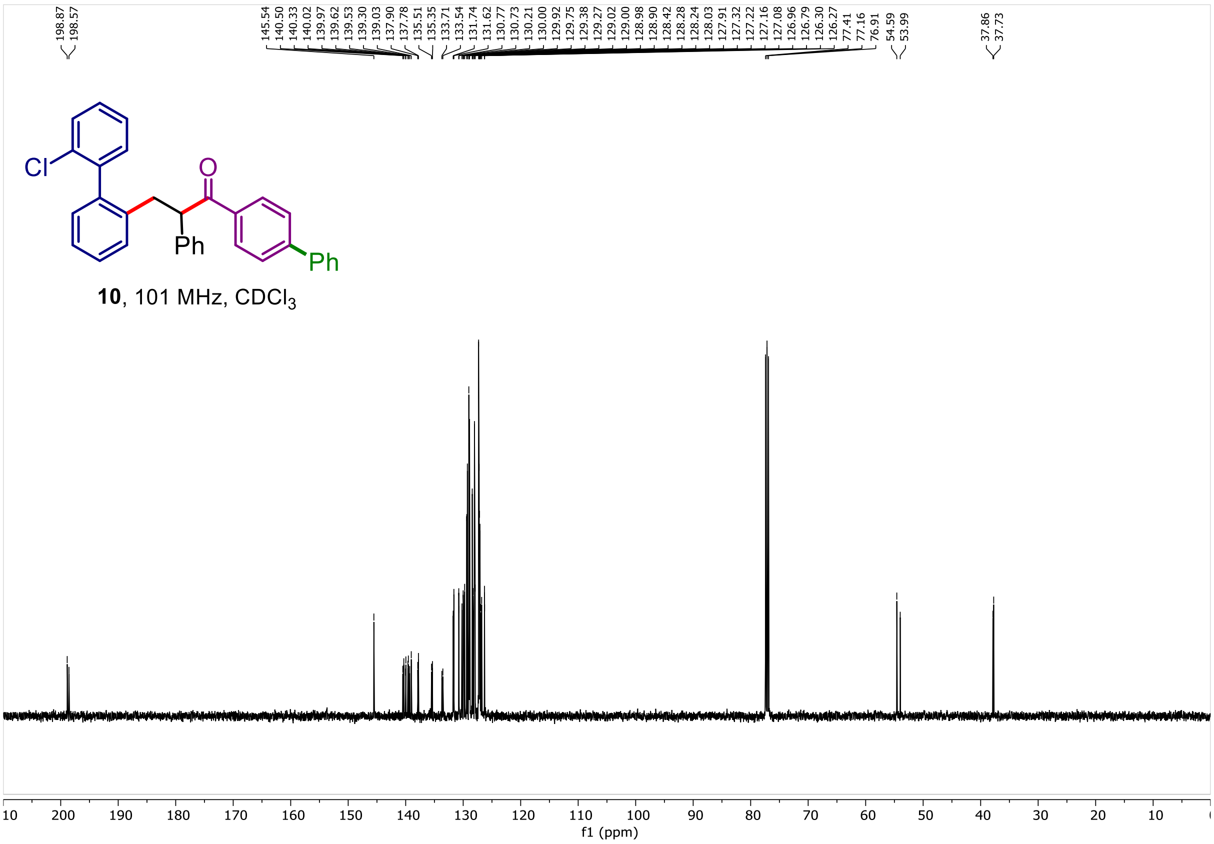

3-(4''-methoxy-[1,1':2',1''-terphenyl]-2-yl)-1,2-diphenylpropan-1-one(11a)

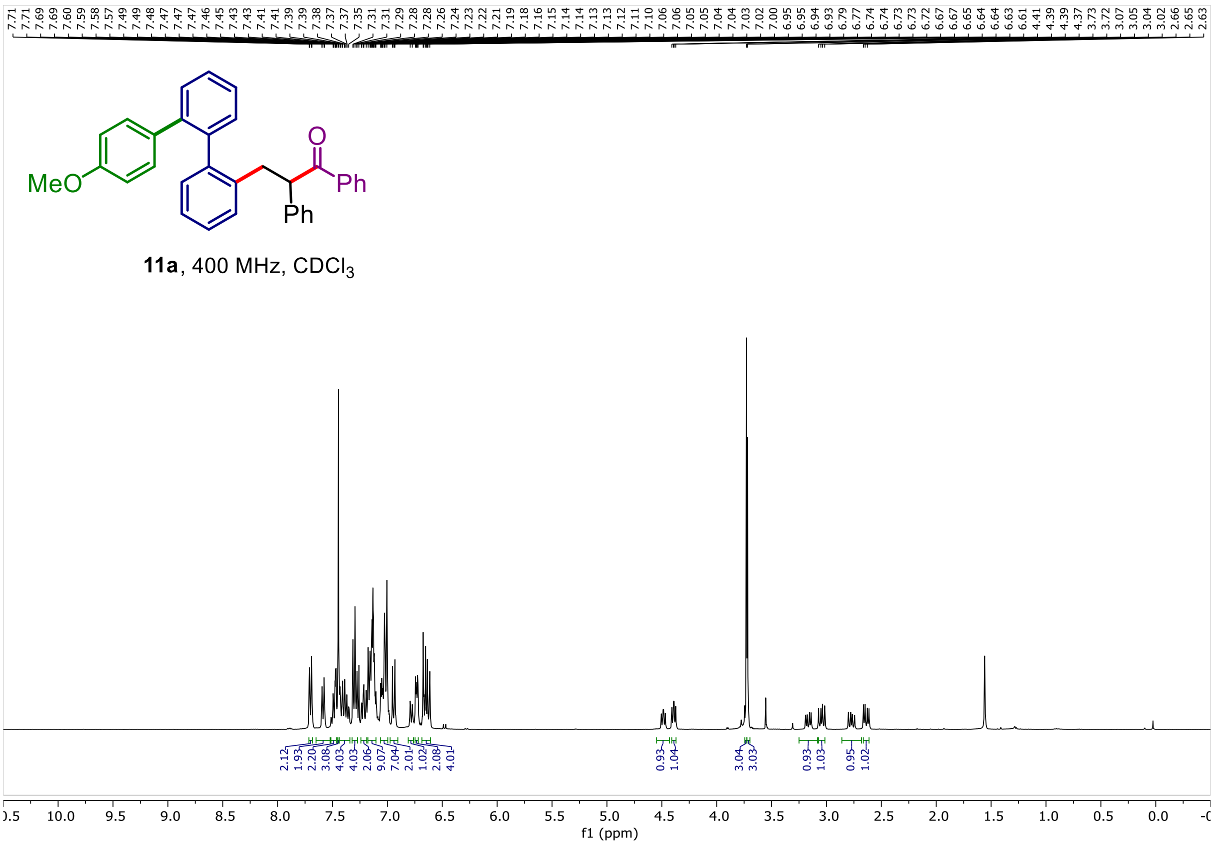

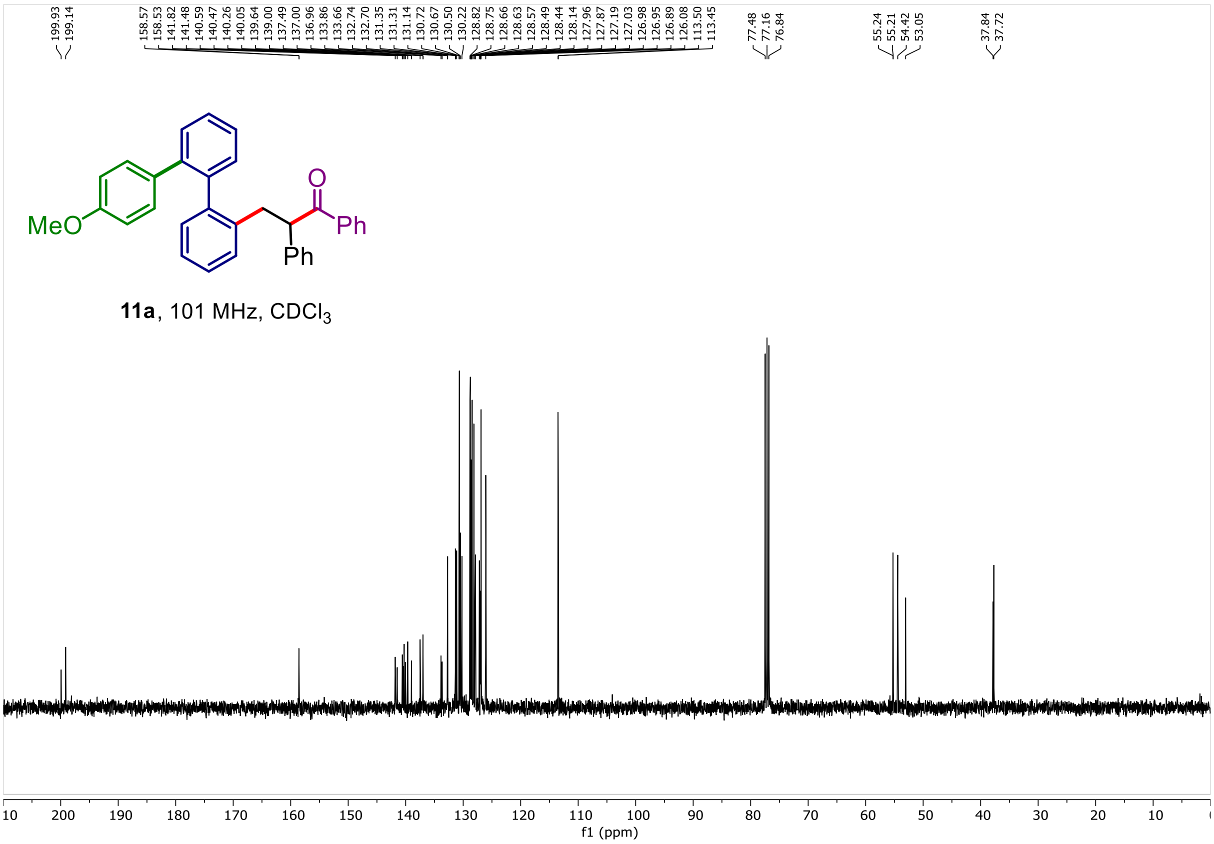

E-1,2-diphenyl-3-(2'-styryl-[1,1'-biphenyl]-2-yl)propan-1-one(11b)

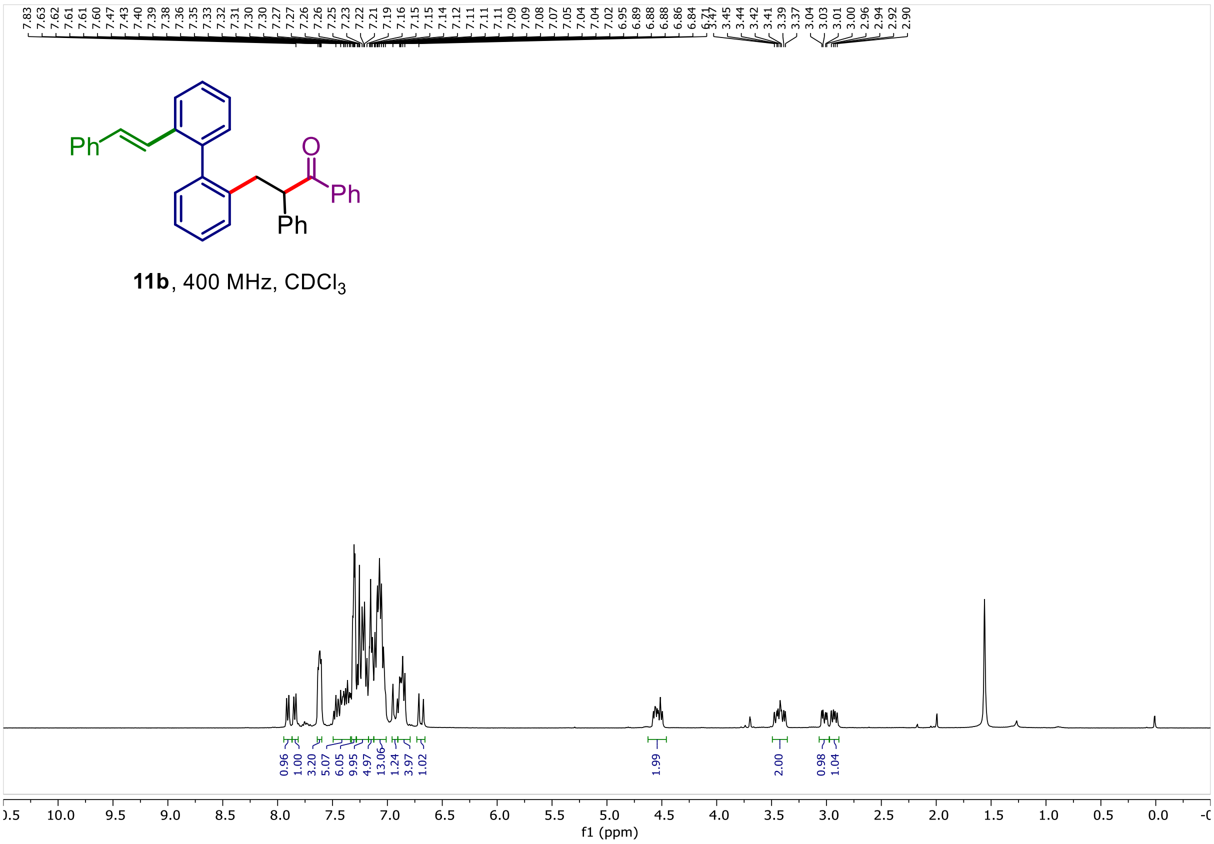

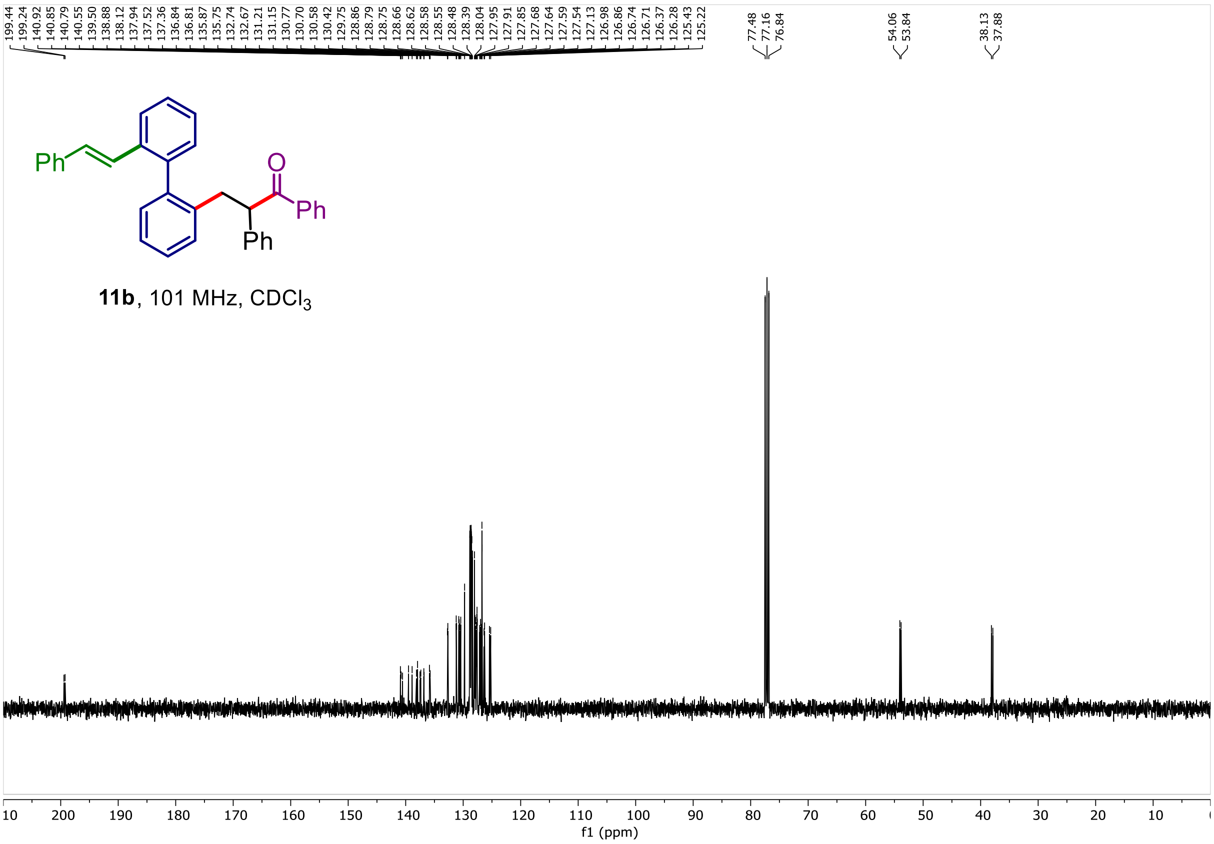

3-(2'-bromo-[1,1'-biphenyl]-2-yl)-1-(4-bromophenyl)-2-phenylpropan-1-one(4a')

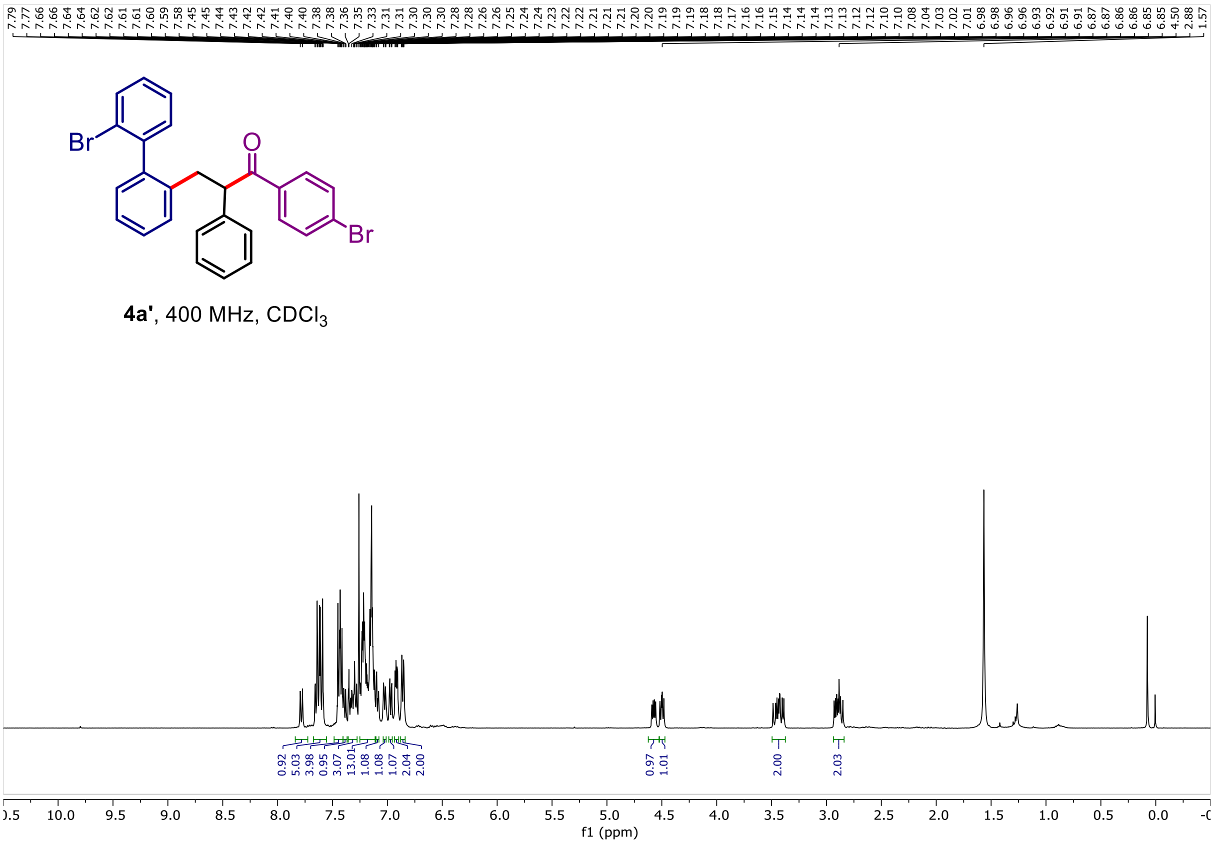

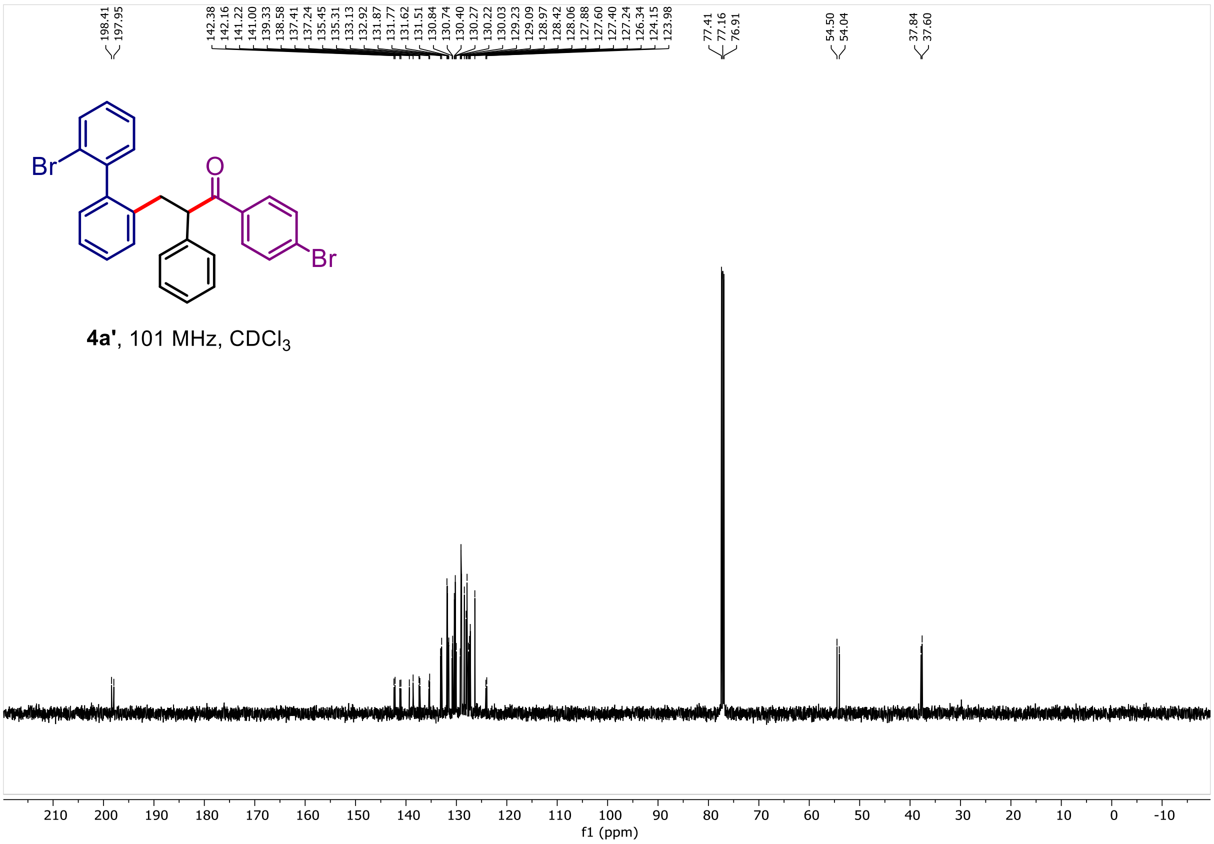

Supplement: SC-017-D5SC09326K-s001 [file SC-017-D5SC09326K-s001.pdf]
