# Supplementary material for: Unlocking radical reactivity of cyclic diaryl λ3-chloranes through NHC-catalyzed three-component coupling
Source: Chem Sci. 2025 Dec 30;17(8):4277–84. doi: 10.1039/d5sc09326k (PMC12805865; doi:10.1039/d5sc09326k)
Supplement: SC-017-D5SC09326K-s002 [file SC-017-D5SC09326K-s002.pdf]

Supplementary Information (SI) for Chemical Science.
This journal is © The Royal Society of Chemistry 2025

-Electronic Supporting Information-

Part B

Unlocking Radical Reactivity of Cyclic Diaryl λ³-Chloranes Through NHC-Catalyzed Three-Component Coupling

Anusree A. Kunhiraman[a], Koushik Patra[a], Venkata Surya Kumar Choutipalli[b], Manjeet Godara[a], Kevin L. Shuford[*b] and Mahiuddin Baidya[*a]

[a]Department of Chemistry, Indian Institute of Technology Madras,
Chennai, 600036, India.
E-mail: mbaidya@iitm.ac.in

[b]Department of Chemistry and Biochemistry, Baylor University, One Bear Place #97348,
Waco, Texas 76798-7348, United States
E-mail: kevin_shuford@baylor.edu

Computational Details

All the quantum chemical computations based on density functional theory (DFT) were carried out using the Gaussian 16 software package, Revision C.01.[1] Molecular geometries were optimized employing the ωB97X-D functional paired with the def2-TZVP basis set.[2,3] To verify the nature of the stationary points, harmonic vibrational frequency analyses were performed at the same level of theory and structures with no imaginary frequencies were identified as minima, while those with a single imaginary frequency were classified as transition states. Intrinsic reaction coordinate (IRC) analyses were also conducted at this level to ensure proper connectivity between each transition state and its corresponding intermediates. Solvent effects were accounted by performing single-point energy calculations within the SMD continuum model (DMSO as solvent) at the same level of theory, allowing for the evaluation of thermodynamic parameters such as Gibbs free energy and enthalpy at 298 K and 1 atm.[4]

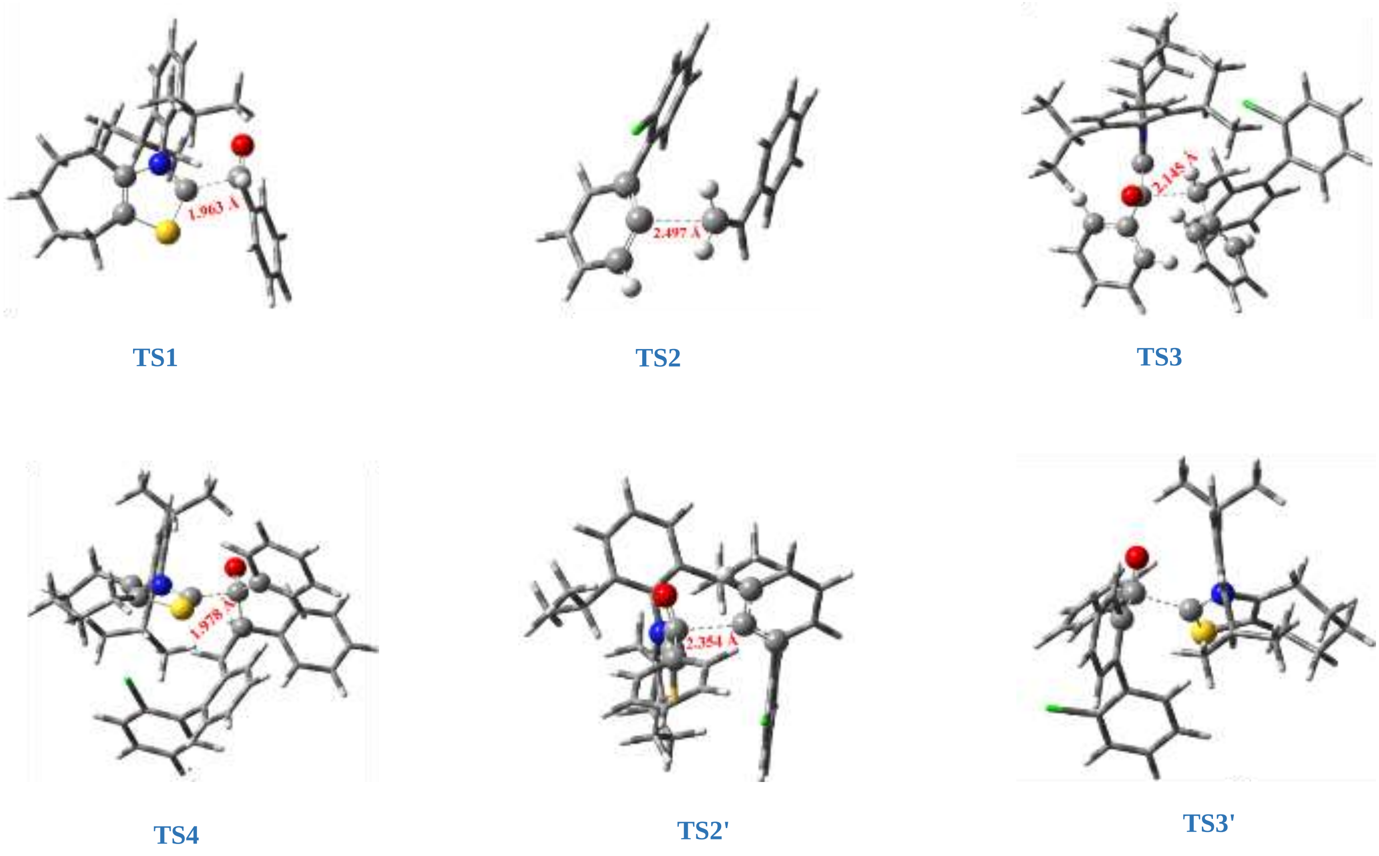

Figure S1: Optimized geometries of transition states computed at ωB97X-D/def2-TZVP level of theory.

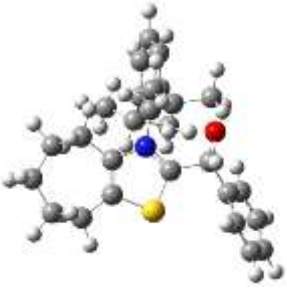

B

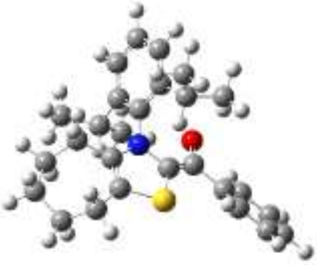

C

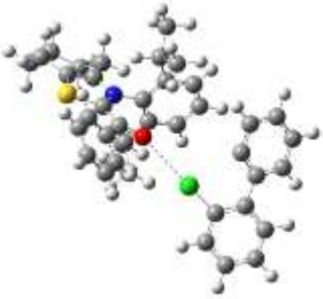

D + 1a$^{\bullet}$

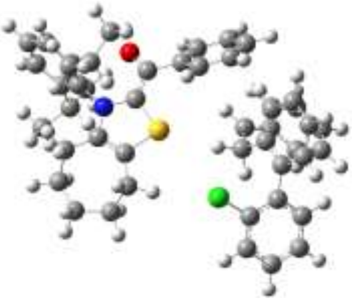

D + E

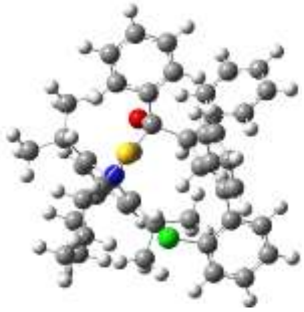

F

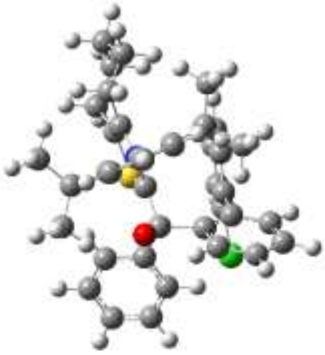

D-1a

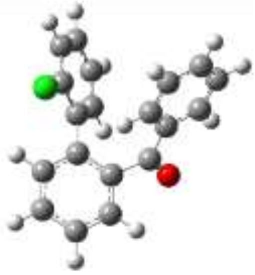

13

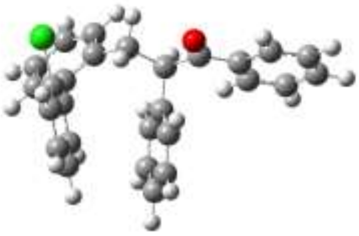

4a

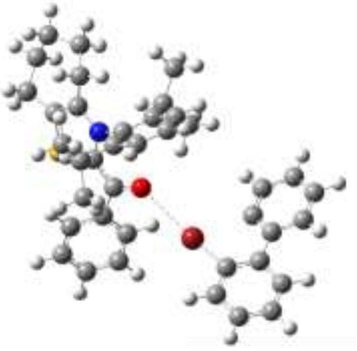

D + 1a'$^{\bullet}$

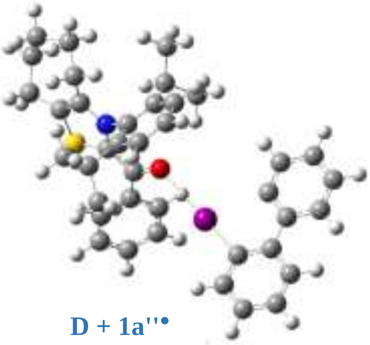

D + 1a''$^{\bullet}$

Figure S2: Optimized geometries of intermediates computed at ωB97X-D/def2-TZVP level of theory.

Relaxation energy profiles for the single electron transfer process:

To explore the experimental hypothesis of a single-electron transfer (SET) between intermediate **C** and the hypervalent chloronium species **1a**, we analyzed their electronic interactions. The optimized structure of cationic chlorine intermediate **1a** (charge = +1, multiplicity = 1) features two symmetric and equivalent C–Cl bonds. Upon addition of one electron, yielding the neutral radical species (charge = 0, multiplicity = 2), one of the two C–Cl bonds undergoes spontaneous cleavage, generating the mono-chlorinated neutral radical **1a•**. The relaxation energy profile for this process is depicted in **Figure S3b** and indicates that the transformation is barrierless. To further support the SET hypothesis, we constructed a combined system of **C** and **1a**, mimicking the proposed experimental conditions. Upon geometry optimization, the system evolved spontaneously, with cleavage of one C–Cl bond in the **1a** unit, resulting in the formation of two neutral radicals: **D** and **1a•** (**Figure S3c**).

Analogous calculations carried out for the bromonium (**1a'**, **Figure S4**) and iodonium (**1a''**, **Figure S5**) congeners of **1a** demonstrate that these transformations are likewise exothermic, with computed reaction energies of –17.51 kcal/mol and –7.76 kcal/mol), respectively.

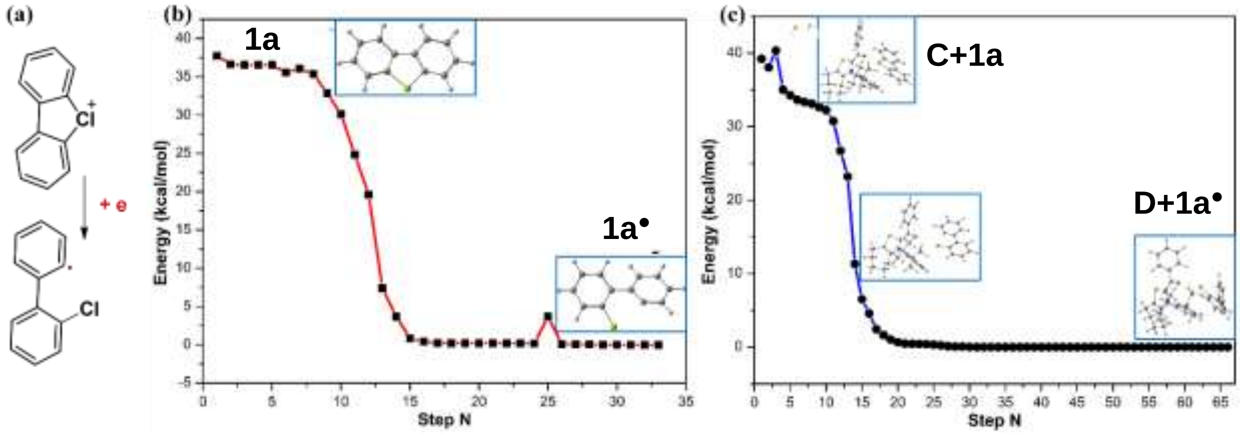

Figure S3: Calculated energy profile (kcal/mol) for Single electron Transfer (SET) between intermediate **C** and multivalent chloronium ion **1a** computed at ωB97X-D/def2-TZVP level of theory.

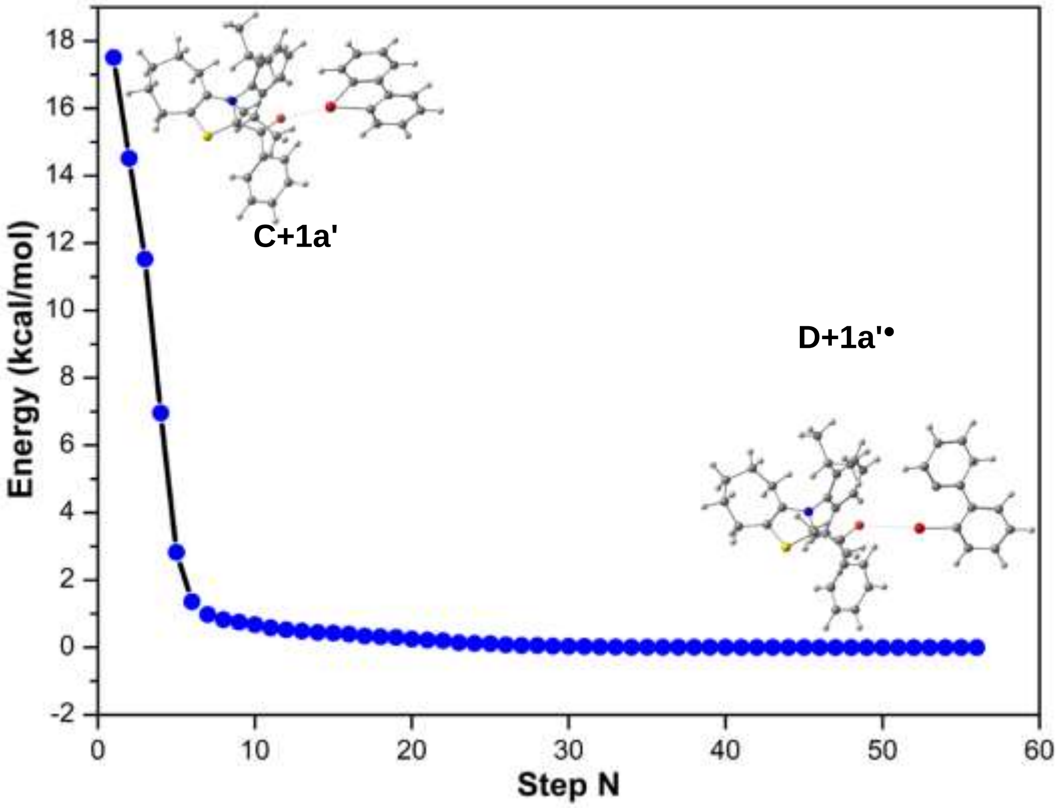

Figure S4: Calculated energy profile (kcal/mol) for single electron transfer (SET) between intermediate **C** and multivalent bromonium ion **1a'** computed at ωB97X-D/def2-TZVP level of theory.

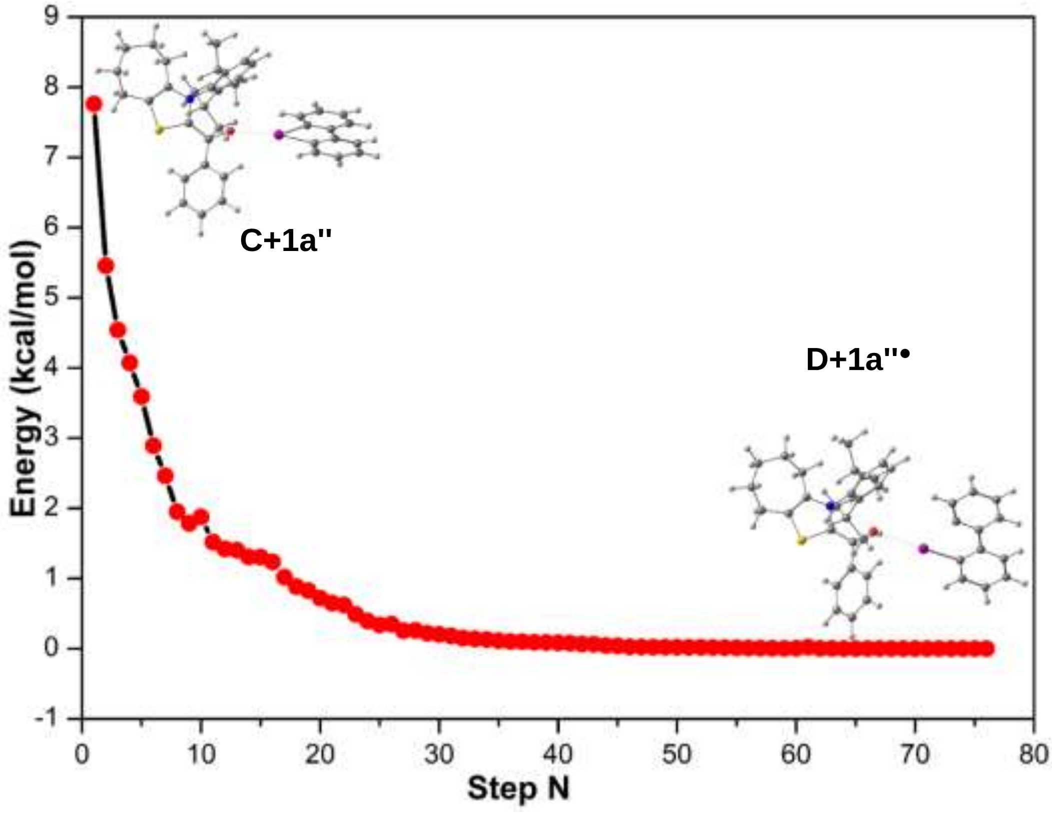

Figure S5: Calculated energy profile (kcal/mol) for Single electron Transfer (SET) between intermediate **C** and multivalent Iodonium ion **1a''** computed at ωB97X-D/def2-TZVP level of theory.

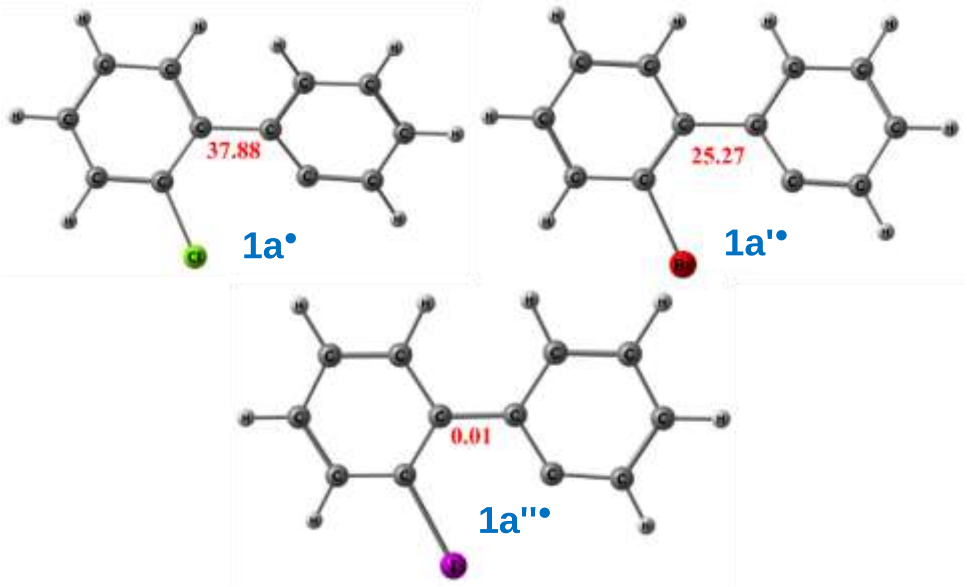

Figure S6: Measured dihedral angles of biphenyl moiety of radical intermediates computed at ωB97X-D/def2-TZVP level of theory.

Table S1: Cartesian coordinates (Å) of the optimized structures of all intermediates and transition states at ωB97X-D/def2-TZVP level of theory.

A

C	1.135161000	-0.506795000	-0.151088000
C	2.183637000	-0.969966000	0.572556000
C	0.056521000	-0.653303000	1.962419000
N	-0.010415000	-0.346984000	0.651964000
S	1.659964000	-1.180199000	2.225034000
C	-1.237519000	0.169962000	0.103004000
C	-1.421297000	1.560979000	0.073505000
C	-2.187337000	-0.733219000	-0.397584000
C	-2.619674000	2.044100000	-0.458827000
C	-3.370609000	-0.202717000	-0.919248000
C	-3.586150000	1.171264000	-0.948070000
H	-2.802700000	3.119890000	-0.490094000
H	-4.137871000	-0.874077000	-1.309310000
H	-4.517642000	1.566716000	-1.359044000
C	-1.958409000	-2.234062000	-0.322911000
H	-0.871286000	-2.400101000	-0.268153000
C	-0.376528000	2.503028000	0.649945000
H	0.585902000	1.968752000	0.655974000
C	-0.179987000	3.767937000	-0.187648000
H	0.670152000	4.349325000	0.200384000
H	0.024002000	3.526353000	-1.242221000
H	-1.062024000	4.426462000	-0.155926000
C	-0.717718000	2.830886000	2.109136000
H	-1.681153000	3.361894000	2.172171000
H	-0.792591000	1.908950000	2.704560000
H	0.057450000	3.473276000	2.555234000
C	-2.567744000	-2.791876000	0.970043000
H	-3.659657000	-2.643821000	0.977812000
H	-2.367807000	-3.871038000	1.059692000
H	-2.143846000	-2.283277000	1.848455000
C	-2.466132000	-2.983275000	-1.556342000
H	-3.565236000	-2.970527000	-1.623761000
H	-2.064392000	-2.552254000	-2.486115000
H	-2.159351000	-4.039033000	-1.508565000
C	1.141453000	-0.173568000	-1.614641000
C	2.224188000	0.834689000	-2.017278000
C	3.659232000	0.302606000	-1.990457000
C	3.576154000	-1.243122000	0.085096000
C	4.235859000	-0.049794000	-0.616782000
H	1.277622000	-1.104264000	-2.194070000
H	2.143221000	1.726050000	-1.371797000
H	3.720776000	-0.587536000	-2.642987000
H	3.560214000	-2.107257000	-0.603706000
H	0.155795000	0.219177000	-1.898601000
H	2.005060000	1.174768000	-3.042065000
H	4.313800000	1.059047000	-2.452856000
H	4.198346000	-1.549753000	0.939174000
H	5.306312000	-0.278274000	-0.744099000
H	4.181770000	0.830106000	0.046665000

2a

C	-1.996761000	0.466094000	0.000012000
O	-2.836506000	-0.396686000	-0.000039000
C	-0.532402000	0.215842000	0.000000000
C	0.360767000	1.291162000	0.000036000
C	-0.046927000	-1.097746000	0.000015000
C	1.734537000	1.058571000	-0.000034000
H	-0.025005000	2.314894000	0.000033000
C	1.323682000	-1.329337000	0.000023000
H	-0.766436000	-1.919413000	0.000043000
C	2.213428000	-0.251316000	-0.000029000
H	2.433242000	1.897573000	-0.000016000
H	1.706044000	-2.352206000	0.000035000
H	3.290121000	-0.435773000	-0.000015000
H	-2.283856000	1.548788000	0.000084000

TS1				B			
C	-1.492620000	1.088646000	-0.554124000	C	-1.238231000	1.195435000	-0.636100000
C	-0.902141000	2.242451000	-0.956154000	C	-0.463876000	2.250289000	-0.995674000
C	0.731479000	0.377203000	-0.554654000	C	0.840227000	0.197470000	-0.502161000
N	-0.547254000	0.071868000	-0.331215000	N	-0.474191000	0.053675000	-0.341655000
S	0.818580000	2.003566000	-1.043638000	S	1.208805000	1.796358000	-0.966982000
C	2.123787000	-0.999852000	-0.697284000	C	1.856798000	-0.987662000	-0.384978000
O	1.804149000	-2.016306000	-0.059411000	O	1.430020000	-1.936139000	0.392059000
C	3.343760000	-0.186700000	-0.288968000	C	3.243992000	-0.383715000	-0.065830000
C	3.887019000	0.789368000	-1.128022000	C	4.012335000	0.280398000	-1.026371000
C	3.961545000	-0.457136000	0.932559000	C	3.754778000	-0.539392000	1.222827000
C	5.005572000	1.521189000	-0.731994000	C	5.253010000	0.824799000	-0.692108000
H	3.434586000	0.971704000	-2.108756000	H	3.648144000	0.356122000	-2.056733000
C	5.080096000	0.271713000	1.331758000	C	4.993630000	0.001505000	1.560412000
H	3.551198000	-1.263767000	1.543986000	H	3.161831000	-1.116631000	1.935180000
C	5.600151000	1.268289000	0.504577000	C	5.742808000	0.692822000	0.606509000
H	5.422222000	2.284106000	-1.394052000	H	5.846194000	1.339235000	-1.452141000
H	5.557294000	0.056037000	2.290935000	H	5.385270000	-0.124874000	2.572878000
H	6.478723000	1.837900000	0.816474000	H	6.716484000	1.112297000	0.870519000
C	-0.971127000	-1.211866000	0.191313000	C	-1.144369000	-1.146126000	0.141725000
C	-1.144966000	-1.320360000	1.578946000	C	-1.551473000	-1.158778000	1.484567000
C	-1.252170000	-2.250512000	-0.699816000	C	-1.493065000	-2.141529000	-0.774784000
C	-1.644924000	-2.527184000	2.069781000	C	-2.353707000	-2.222483000	1.898400000
C	-1.755806000	-3.437655000	-0.161230000	C	-2.300029000	-3.184049000	-0.310875000
C	-1.955106000	-3.574301000	1.206664000	C	-2.734255000	-3.222354000	1.008024000
H	-1.787964000	-2.656473000	3.143871000	H	-2.680223000	-2.276965000	2.938302000
H	-1.982318000	-4.274285000	-0.825971000	H	-2.579923000	-3.988713000	-0.994269000
H	-2.345972000	-4.511485000	1.608677000	H	-3.364394000	-4.045641000	1.351010000
C	-0.992109000	-2.135269000	-2.190569000	C	-0.940592000	-2.173685000	-2.187284000
H	-0.545117000	-1.148358000	-2.380932000	H	-0.420195000	-1.221762000	-2.373897000
C	-0.720254000	-0.198390000	2.511446000	C	-1.046729000	-0.124106000	2.475556000
H	-0.772553000	0.747265000	1.948882000	H	-0.783155000	0.786584000	1.913357000
C	-1.627292000	-0.036438000	3.731366000	C	-2.084203000	0.284942000	3.521870000
H	-1.337552000	0.860181000	4.299970000	H	-1.699978000	1.120190000	4.126932000
H	-2.684367000	0.068026000	3.441271000	H	-3.032392000	0.602087000	3.060293000
H	-1.548463000	-0.892373000	4.419247000	H	-2.308249000	-0.537853000	4.218045000
C	0.746271000	-0.407445000	2.910608000	C	0.244802000	-0.653534000	3.116512000
H	0.844963000	-1.302360000	3.545405000	H	0.010781000	-1.481921000	3.804764000
H	1.371382000	-0.572595000	2.022289000	H	0.911281000	-1.052986000	2.333499000
H	1.131454000	0.459450000	3.469225000	H	0.753323000	0.137315000	3.690070000
C	0.033637000	-3.185010000	-2.634520000	C	0.107453000	-3.292345000	-2.283703000
H	-0.391873000	-4.200427000	-2.589840000	H	-0.377404000	-4.280810000	-2.233392000
H	0.347886000	-3.002391000	-3.673806000	H	0.654021000	-3.233903000	-3.237882000
H	0.910815000	-3.151648000	-1.970770000	H	0.811459000	-3.212676000	-1.438943000
C	-2.291446000	-2.209841000	-2.998453000	C	-2.034600000	-2.299115000	-3.250206000
H	-2.791149000	-3.182225000	-2.863610000	H	-2.581874000	-3.250384000	-3.160074000
H	-3.000541000	-1.424512000	-2.693440000	H	-2.768129000	-1.481313000	-3.173713000
H	-2.086676000	-2.086455000	-4.073092000	H	-1.593473000	-2.269794000	-4.258294000
C	-2.962444000	0.883023000	-0.336652000	C	-2.736385000	1.210646000	-0.549651000
C	-3.582968000	1.865980000	0.663704000	C	-3.294959000	2.292195000	0.383336000
C	-3.729369000	3.304839000	0.162645000	C	-3.196983000	3.726844000	-0.139031000
C	-1.573079000	3.552115000	-1.248979000	C	-0.914649000	3.636715000	-1.351513000
C	-2.433527000	4.078695000	-0.093342000	C	-1.787172000	4.304258000	-0.281612000
H	-3.478396000	0.976738000	-1.308528000	H	-3.139113000	1.360757000	-1.566883000
H	-2.994451000	1.850467000	1.596504000	H	-2.795515000	2.215788000	1.363080000
H	-4.331171000	3.299649000	-0.764410000	H	-3.709650000	3.788524000	-1.116179000
H	-2.198944000	3.444075000	-2.152915000	H	-1.468465000	3.598909000	-2.306524000

H	-3.133909000	-0.146469000	0.003493000
H	-4.584883000	1.491249000	0.925674000
H	-4.322499000	3.866797000	0.901732000
H	-0.804822000	4.298360000	-1.500474000
H	-2.697641000	5.124482000	-0.317106000
H	-1.823581000	4.101316000	0.825635000
H	2.002806000	-0.984960000	-1.817193000

H	-3.088601000	0.224723000	-0.223210000
H	-4.357278000	2.063145000	0.560858000
H	-3.767138000	4.379719000	0.540810000
H	-0.028465000	4.260666000	-1.540926000
H	-1.876000000	5.371821000	-0.537542000
H	-1.263634000	4.258767000	0.688286000
H	1.915420000	-1.245443000	-1.497606000

C

C	1.462035000	1.135165000	0.396251000
C	0.870697000	2.311761000	0.689805000
C	-0.791374000	0.505035000	0.062740000
N	0.582888000	0.089008000	0.126100000
S	-0.895414000	2.152717000	0.764805000
C	-1.761093000	-0.266187000	-0.547110000
O	-1.517726000	-1.411535000	-1.066011000
C	-3.186569000	0.209561000	-0.595470000
C	-3.792334000	1.107789000	0.297700000
C	-3.994998000	-0.348906000	-1.602289000
C	-5.132461000	1.473851000	0.154102000
H	-3.233770000	1.502745000	1.148437000
C	-5.326654000	0.019187000	-1.750968000
H	-3.522310000	-1.094047000	-2.245290000
C	-5.906255000	0.943608000	-0.875949000
H	-5.577607000	2.172902000	0.868429000
H	-5.926412000	-0.422478000	-2.552766000
H	-6.955218000	1.232477000	-0.987093000
C	0.977900000	-1.274298000	0.212231000
C	1.715562000	-1.843594000	-0.847655000
C	0.675812000	-2.029897000	1.361735000
C	2.189497000	-3.149278000	-0.713223000
C	1.151563000	-3.341578000	1.446141000
C	1.918053000	-3.897725000	0.428454000
H	2.757973000	-3.601508000	-1.530444000
H	0.914832000	-3.940916000	2.329635000
H	2.289066000	-4.922454000	0.515778000
C	-0.219605000	-1.471618000	2.452557000
H	-0.314024000	-0.391108000	2.279486000
C	1.886343000	-1.097146000	-2.160209000
H	1.643809000	-0.043060000	-1.966025000
C	3.308802000	-1.156069000	-2.720101000
H	3.391260000	-0.524975000	-3.619826000
H	4.051886000	-0.801054000	-1.988150000
H	3.591936000	-2.179817000	-3.015622000
C	0.843960000	-1.609529000	-3.161439000
H	1.053456000	-2.658037000	-3.435545000
H	-0.154912000	-1.559833000	-2.693038000
H	0.858841000	-1.004758000	-4.083947000
C	-1.621242000	-2.083098000	2.312207000
H	-1.617450000	-3.137651000	2.639920000
H	-2.354830000	-1.536738000	2.926917000
H	-1.939426000	-2.055811000	1.259087000
C	0.352939000	-1.659310000	3.859447000
H	0.436087000	-2.723573000	4.135830000
H	1.354313000	-1.209129000	3.947189000
H	-0.302665000	-1.179351000	4.603900000
C	2.954849000	0.937486000	0.387694000

D

C	1.420019000	1.138863000	0.361769000
C	0.821380000	2.352928000	0.426058000
C	-0.772813000	0.498678000	-0.108107000
N	0.542270000	0.100481000	0.064646000
S	-0.897077000	2.226783000	0.124975000
C	-1.827321000	-0.368648000	-0.538342000
O	-1.603115000	-1.516601000	-0.952575000
C	-3.243144000	0.135919000	-0.505793000
C	-3.761935000	0.904723000	0.542798000
C	-4.098415000	-0.262861000	-1.541158000
C	-5.101439000	1.293175000	0.539529000
H	-3.125043000	1.184465000	1.384986000
C	-5.431438000	0.133943000	-1.550222000
H	-3.695560000	-0.897450000	-2.332804000
C	-5.936306000	0.917321000	-0.510604000
H	-5.495647000	1.885424000	1.368364000
H	-6.084915000	-0.173590000	-2.369765000
H	-6.984346000	1.225277000	-0.514844000
C	0.937555000	-1.278182000	0.165430000
C	1.561906000	-1.889928000	-0.932010000
C	0.725289000	-1.945676000	1.380900000
C	1.979869000	-3.213597000	-0.786965000
C	1.152970000	-3.272844000	1.475920000
C	1.776479000	-3.901938000	0.404841000
H	2.461166000	-3.720728000	-1.626356000
H	0.991849000	-3.824512000	2.405068000
H	2.103684000	-4.939806000	0.497519000
C	0.018582000	-1.282909000	2.552068000
H	-0.122183000	-0.220314000	2.306546000
C	1.737337000	-1.165773000	-2.254905000
H	1.459533000	-0.112890000	-2.096363000
C	3.188178000	-1.189789000	-2.744166000
H	3.296098000	-0.579415000	-3.654078000
H	3.879644000	-0.794097000	-1.984520000
H	3.515831000	-2.211041000	-2.994393000
C	0.771428000	-1.734891000	-3.300921000
H	1.029967000	-2.777827000	-3.545743000
H	-0.256809000	-1.722186000	-2.913479000
H	0.817157000	-1.147867000	-4.231562000
C	-1.375246000	-1.891322000	2.750136000
H	-1.303923000	-2.944224000	3.066861000
H	-1.933008000	-1.343171000	3.525281000
H	-1.954006000	-1.864334000	1.815850000
C	0.852701000	-1.337330000	3.835280000
H	1.001852000	-2.371835000	4.182259000
H	1.845912000	-0.887296000	3.685047000
H	0.347496000	-0.788377000	4.644817000
C	2.884170000	0.889174000	0.583722000
C	3.801845000	1.711050000	-0.329567000

C	3.702938000	1.843659000	-0.597023000
C	3.823542000	3.309674000	-0.174268000
C	1.545657000	3.614152000	1.005675000
C	2.519489000	4.104013000	-0.073175000
H	3.346855000	1.116425000	1.405809000
H	3.217123000	1.777048000	-1.585029000
H	4.337313000	3.351120000	0.804900000
H	2.089008000	3.549123000	1.969893000
H	3.182935000	-0.111514000	0.161364000
H	4.724018000	1.445254000	-0.727040000
H	4.491414000	3.823469000	-0.887207000
H	0.770740000	4.382681000	1.152452000
H	2.780476000	5.156571000	0.136298000
H	2.001178000	4.093510000	-1.046945000

C	3.899849000	3.201444000	0.002170000
C	1.463178000	3.677863000	0.718835000
C	2.631408000	4.030103000	-0.209296000
H	3.125941000	1.108512000	1.638744000
H	3.477163000	1.581612000	-1.375829000
H	4.229049000	3.312423000	1.051505000
H	1.813619000	3.693669000	1.767126000
H	3.090240000	-0.179682000	0.442670000
H	4.813288000	1.278972000	-0.264905000
H	4.703195000	3.638321000	-0.612646000
H	0.697613000	4.465094000	0.647264000
H	2.884478000	5.090982000	-0.052136000
H	2.296409000	3.939852000	-1.256414000

1a

C	-0.000001000	-0.609138000	0.730853000
C	0.000000000	-1.683006000	1.625928000
C	0.000000000	-1.434172000	2.996343000
C	-0.000001000	-0.129485000	3.499741000
C	0.000000000	0.972117000	2.641337000
C	-0.000001000	0.663507000	1.297431000
C	-0.000001000	-0.609138000	-0.730853000
C	-0.000001000	0.663507000	-1.297431000
C	0.000000000	0.972117000	-2.641337000
C	-0.000001000	-0.129485000	-3.499741000
C	0.000000000	-1.434172000	-2.996343000
C	0.000000000	-1.683006000	-1.625928000
H	0.000000000	-2.707223000	1.249701000
H	0.000000000	-2.274814000	3.691707000
H	-0.000001000	0.041018000	4.577433000
H	-0.000001000	1.995350000	3.017494000
H	-0.000001000	1.995350000	-3.017494000
H	-0.000001000	0.041018000	-4.577433000
H	0.000000000	-2.274814000	-3.691707000
H	0.000000000	-2.707223000	-1.249701000
Cl	0.000002000	1.913733000	0.000000000

1a•

C	0.577740000	-0.460900000	0.074211000
C	1.172246000	-1.720793000	0.245405000
C	2.552649000	-1.888401000	0.256211000
C	3.384858000	-0.782673000	0.096436000
C	2.827434000	0.481353000	-0.067828000
C	1.441652000	0.638049000	-0.075263000
C	-0.902872000	-0.340568000	0.052159000
C	-1.714057000	-1.328121000	-0.539632000
C	-3.103871000	-1.217829000	-0.528788000
C	-3.728050000	-0.121459000	0.067671000
C	-2.951598000	0.884307000	0.657913000
C	-1.586994000	0.720675000	0.620023000
H	0.525415000	-2.587419000	0.396577000
H	2.977896000	-2.883662000	0.398378000
H	4.470424000	-0.898338000	0.104237000
H	3.460694000	1.360427000	-0.194153000
H	-3.707157000	-1.995171000	-1.001955000
H	-4.817880000	-0.042083000	0.070319000
H	-3.416789000	1.755029000	1.126648000
Cl	0.819828000	2.252446000	-0.295092000
H	-1.244494000	-2.182204000	-1.034600000

TS2

C	-0.527602000	0.897137000	-0.632983000
C	0.383528000	0.724366000	-1.684912000
C	1.524356000	1.510086000	-1.801417000
C	1.781675000	2.499792000	-0.855649000
C	0.903736000	2.686875000	0.206537000
C	-0.235256000	1.889074000	0.314177000
C	-1.768690000	0.075876000	-0.602425000
C	-2.638201000	0.077436000	-1.707163000
C	-3.796713000	-0.699139000	-1.709718000
C	-4.113155000	-1.498065000	-0.611281000
C	-3.259716000	-1.523787000	0.498189000
C	-2.131978000	-0.732102000	0.463121000
H	0.184234000	-0.059526000	-2.418639000
H	2.218593000	1.341021000	-2.626235000
H	2.673286000	3.124869000	-0.936080000
H	1.086921000	3.456493000	0.957565000
H	-4.458719000	-0.677307000	-2.577811000

E

C	-1.753290000	-0.408119000	0.497759000
C	-1.446366000	-1.432979000	1.403027000
C	-2.041797000	-2.688507000	1.314306000
C	-2.972584000	-2.942368000	0.309173000
C	-3.307328000	-1.939690000	-0.596815000
C	-2.702276000	-0.687752000	-0.496125000
C	-1.088884000	0.920404000	0.636361000
C	-1.436415000	1.727816000	1.726748000
C	-0.827094000	2.961781000	1.928951000
C	0.150427000	3.396216000	1.036243000
C	0.507729000	2.593927000	-0.042707000
C	-0.099540000	1.353330000	-0.265219000
H	-0.715564000	-1.226854000	2.188932000
H	-1.777323000	-3.468833000	2.030394000
H	-3.445656000	-3.922978000	0.227631000
H	-4.040538000	-2.116885000	-1.384739000
H	-1.113368000	3.580387000	2.782006000

H	-5.020949000	-2.106536000	-0.616601000
H	-3.486432000	-2.157725000	1.359798000
Cl	-1.313713000	2.194166000	1.654198000
C	-0.432601000	-1.369960000	2.178874000
H	-0.230886000	-0.320576000	2.405152000
H	-1.273803000	-1.827268000	2.702091000
C	0.398055000	-2.122228000	1.426691000
H	0.126828000	-3.167454000	1.242470000
C	1.615475000	-1.656063000	0.750270000
C	2.119273000	-2.381704000	-0.340867000
C	2.310834000	-0.507234000	1.161144000
C	3.268901000	-1.968407000	-1.009136000
H	1.593308000	-3.281645000	-0.670469000
C	3.464073000	-0.100158000	0.500449000
H	1.950461000	0.066639000	2.017449000
C	3.948086000	-0.826131000	-0.588602000
H	3.640538000	-2.545836000	-1.858709000
H	3.991278000	0.794565000	0.838445000
H	4.855629000	-0.504249000	-1.104143000
H	-2.401652000	0.709724000	-2.567457000

H	0.641406000	4.360681000	1.183294000
H	1.289238000	2.929640000	-0.729007000
Cl	-3.162173000	0.548096000	-1.638642000
C	0.319159000	0.515421000	-1.465739000
H	-0.054730000	-0.513605000	-1.333557000
H	-0.198656000	0.906285000	-2.355550000
C	1.795973000	0.485886000	-1.715054000
H	2.189864000	1.011240000	-2.589663000
C	2.726352000	-0.174550000	-0.872369000
C	4.117241000	-0.134488000	-1.164971000
C	2.323245000	-0.899156000	0.282915000
C	5.041340000	-0.775390000	-0.355351000
H	4.454016000	0.415334000	-2.047844000
C	3.256560000	-1.537951000	1.085766000
H	1.264648000	-0.953417000	0.542732000
C	4.619738000	-1.482346000	0.777088000
H	6.104189000	-0.728119000	-0.603465000
H	2.921189000	-2.088766000	1.967698000
H	5.349321000	-1.986498000	1.414176000
H	-2.204239000	1.375855000	2.419962000

TS3

C	1.386581000	-2.084066000	-0.978898000
C	0.901791000	-1.693805000	-2.181095000
C	1.352199000	0.215020000	-0.640977000
N	1.624098000	-1.000865000	-0.110407000
S	0.766375000	0.036780000	-2.247915000
C	1.595988000	1.531170000	0.005579000
O	2.339682000	1.612669000	1.010038000
C	1.655583000	2.694669000	-0.984143000
C	2.612569000	2.625810000	-2.006977000
C	0.942983000	3.886862000	-0.825671000
C	2.818034000	3.694255000	-2.876871000
H	3.219808000	1.723955000	-2.120533000
C	1.142604000	4.955540000	-1.698353000
H	0.224183000	3.987687000	-0.015161000
C	2.073646000	4.863248000	-2.731266000
H	3.568471000	3.613998000	-3.666732000
H	0.565280000	5.872591000	-1.560578000
H	2.228041000	5.703478000	-3.412026000
C	2.328144000	-1.196970000	1.143809000
C	1.632408000	-1.650966000	2.278614000
C	3.718137000	-0.988978000	1.141468000
C	2.373410000	-1.841433000	3.449807000
C	4.406982000	-1.200912000	2.335205000
C	3.742062000	-1.611757000	3.484853000
H	1.863030000	-2.182682000	4.353136000
H	5.485833000	-1.034061000	2.367180000
H	4.297343000	-1.762829000	4.412927000
C	4.487908000	-0.574745000	-0.103294000
H	3.764458000	-0.386473000	-0.909404000
C	0.146249000	-1.989088000	2.290413000
H	-0.269235000	-1.741563000	1.303945000
C	-0.087903000	-3.488739000	2.521530000
H	-1.159458000	-3.722143000	2.424253000
H	0.459291000	-4.115649000	1.805621000
H	0.229435000	-3.786987000	3.533057000
C	-0.633578000	-1.188392000	3.342770000

F

C	-2.598046000	0.114397000	-0.900505000
C	-2.061726000	0.321780000	-2.132527000
C	-0.537659000	-0.892649000	-0.592453000
N	-1.718479000	-0.571812000	-0.049300000
S	-0.465804000	-0.334868000	-2.196165000
C	0.573978000	-1.801422000	0.105824000
O	-0.004818000	-2.605393000	0.962955000
C	1.207857000	-2.659627000	-1.040860000
C	0.322098000	-3.436877000	-1.806521000
C	2.574724000	-2.869554000	-1.248667000
C	0.769964000	-4.338408000	-2.763655000
H	-0.751702000	-3.345271000	-1.634219000
C	3.034379000	-3.779875000	-2.205003000
H	3.315356000	-2.336196000	-0.657340000
C	2.138952000	-4.513311000	-2.974100000
H	0.046068000	-4.918089000	-3.341595000
H	4.110707000	-3.913989000	-2.336385000
H	2.499605000	-5.223584000	-3.721531000
C	-2.155082000	-0.985102000	1.278883000
C	-2.013597000	-0.100702000	2.356687000
C	-2.803849000	-2.226642000	1.392420000
C	-2.476793000	-0.531642000	3.605637000
C	-3.255894000	-2.599538000	2.656167000
C	-3.080789000	-1.770383000	3.759854000
H	-2.368696000	0.126763000	4.470403000
H	-3.745911000	-3.567345000	2.782374000
H	-3.430858000	-2.089283000	4.743821000
C	-3.037217000	-3.154512000	0.211968000
H	-2.577010000	-2.697245000	-0.676432000
C	-1.454649000	1.307955000	2.228026000
H	-1.116636000	1.451317000	1.192170000
C	-2.546038000	2.354069000	2.488539000
H	-2.151013000	3.366636000	2.317199000
H	-3.417398000	2.212816000	1.834719000
H	-2.900958000	2.304583000	3.529831000
C	-0.258649000	1.556834000	3.154501000

H	-0.353763000	-1.497532000	4.362004000	H	-0.575200000	1.593081000	4.208588000
H	-0.459197000	-0.107368000	3.263115000	H	0.499268000	0.767872000	3.065171000
H	-1.713692000	-1.369062000	3.227652000	H	0.215939000	2.520685000	2.914505000
C	5.263961000	0.728746000	0.108180000	C	-2.358726000	-4.512222000	0.417295000
H	6.068086000	0.603188000	0.850599000	H	-2.813234000	-5.063140000	1.256266000
H	5.734289000	1.047770000	-0.835295000	H	-2.471148000	-5.133129000	-0.485948000
H	4.580115000	1.515692000	0.455690000	H	-1.291568000	-4.343822000	0.622436000
C	5.397069000	-1.714472000	-0.578672000	C	-4.535521000	-3.303388000	-0.079920000
H	6.170206000	-1.947172000	0.170542000	H	-5.057381000	-3.795445000	0.755929000
H	4.824932000	-2.636709000	-0.763882000	H	-5.021057000	-2.329390000	-0.245037000
H	5.909387000	-1.436175000	-1.512578000	H	-4.692309000	-3.920717000	-0.977929000
C	1.756315000	-3.488737000	-0.605372000	C	-3.978670000	0.510193000	-0.468765000
C	0.673382000	-4.534520000	-0.889546000	C	-4.346167000	1.967102000	-0.772946000
C	0.460251000	-4.875951000	-2.364941000	C	-4.599366000	2.292330000	-2.245734000
C	0.646129000	-2.517043000	-3.412314000	C	-2.708817000	0.919156000	-3.350052000
C	-0.187263000	-3.784594000	-3.216119000	C	-3.366631000	2.286685000	-3.149150000
H	2.676576000	-3.760408000	-1.153493000	H	-4.705180000	-0.159422000	-0.962146000
H	-0.279017000	-4.201505000	-0.445250000	H	-3.557037000	2.629786000	-0.381495000
H	1.429239000	-5.161306000	-2.813751000	H	-5.350665000	1.589330000	-2.649296000
H	1.622176000	-2.790075000	-3.853900000	H	-3.464103000	0.205366000	-3.725515000
H	2.029947000	-3.516272000	0.456160000	H	-4.081288000	0.319019000	0.607192000
H	0.959417000	-5.457590000	-0.360730000	H	-5.260758000	2.199942000	-0.205312000
H	-0.174854000	-5.774217000	-2.422247000	H	-5.059860000	3.291341000	-2.303260000
H	0.147952000	-1.879948000	-4.158778000	H	-1.950884000	0.999759000	-4.143644000
H	-0.393696000	-4.200569000	-4.214995000	H	-3.661152000	2.659858000	-4.142679000
H	-1.165405000	-3.512402000	-2.791112000	H	-2.619783000	2.998753000	-2.762769000
C	-0.340655000	1.243838000	0.884189000	C	1.523319000	-0.752356000	0.882387000
C	-1.493831000	0.452925000	0.301120000	C	1.620012000	0.722072000	0.449849000
C	-0.637591000	2.486161000	1.625672000	C	2.817791000	-1.349477000	1.406209000
C	-2.365176000	0.990295000	-0.810696000	C	2.306298000	1.245181000	-0.793229000
H	-2.150476000	0.180423000	1.147070000	H	2.057554000	1.256761000	1.308459000
H	-1.107470000	-0.513705000	-0.063451000	H	0.593361000	1.113876000	0.402991000
C	0.266868000	2.922466000	2.616803000	C	2.776610000	-2.577048000	2.089288000
C	-1.789574000	3.268466000	1.406892000	C	4.056471000	-0.712542000	1.264516000
C	-3.673194000	0.489845000	-0.969710000	C	2.396053000	2.645415000	-0.948180000
C	-1.908044000	1.947827000	-1.719716000	C	2.823045000	0.442509000	-1.810035000
C	0.056330000	4.111620000	3.308158000	C	3.941493000	-3.155477000	2.581273000
H	1.159071000	2.324872000	2.805116000	H	1.806124000	-3.069330000	2.184470000
C	-2.002595000	4.449723000	2.112395000	C	5.224610000	-1.292751000	1.764018000
H	-2.529075000	2.959183000	0.668350000	H	4.130006000	0.241252000	0.739138000
C	-4.260031000	-0.456258000	0.026078000	C	1.887067000	3.567226000	0.111550000
C	-4.472900000	0.943904000	-2.024181000	C	2.987151000	3.194852000	-2.087616000
C	-2.705283000	2.392595000	-2.770422000	C	3.409144000	0.993372000	-2.948898000
H	-0.913747000	2.373048000	-1.582656000	H	2.765692000	-0.638909000	-1.717632000
C	-1.076055000	4.886692000	3.059257000	C	5.173908000	-2.518836000	2.420332000
H	0.783990000	4.432418000	4.057577000	H	3.887205000	-4.116556000	3.098484000
H	-2.905038000	5.035098000	1.918973000	H	6.179923000	-0.778387000	1.633636000
C	-5.214820000	0.018192000	0.936182000	C	2.761870000	4.077759000	1.079251000
C	-3.898770000	-1.806096000	0.128821000	C	0.547871000	3.968717000	0.186102000
C	-3.992853000	1.885672000	-2.930506000	C	3.494024000	2.373919000	-3.093324000
H	-5.486522000	0.548941000	-2.128736000	H	3.045298000	4.281907000	-2.183633000
H	-2.316464000	3.145223000	-3.459883000	H	3.801048000	0.330318000	-3.723278000
H	-1.243459000	5.815942000	3.608648000	H	6.087437000	-2.975035000	2.808928000
C	-5.772185000	-0.805865000	1.909932000	C	2.323255000	4.950163000	2.071973000
H	-5.503951000	1.069852000	0.877385000	H	3.809179000	3.769313000	1.042938000
C	-4.445123000	-2.645886000	1.097653000	C	0.093740000	4.854547000	1.160963000
Cl	-2.726870000	-2.488073000	-0.975800000	Cl	-0.610136000	3.334860000	-0.961578000
H	-4.627228000	2.229185000	-3.750359000	H	3.953317000	2.814075000	-3.980889000
C	-5.383607000	-2.141313000	1.993480000	C	0.986921000	5.343985000	2.109767000
H	-6.506508000	-0.401447000	2.609074000	H	3.028468000	5.326482000	2.815307000

H	-4.133113000	-3.690364000	1.139675000	H	-0.954997000	5.154423000	1.168958000
H	-5.810703000	-2.796689000	2.755089000	H	0.633935000	6.033668000	2.878879000
H	0.230855000	0.584097000	1.535767000	H	0.883054000	-0.688838000	1.775259000

**TS4**				**4a**			
C	-2.538394000	-1.035559000	-0.800146000	C	2.680242000	-0.402529000	-0.005689000
C	-2.223749000	-0.608410000	-2.050783000	C	2.525041000	-1.414909000	0.947903000
C	-0.216876000	-0.897496000	-0.572436000	C	2.804247000	-2.745252000	0.649437000
N	-1.391199000	-1.194255000	-0.001297000	C	3.252590000	-3.090222000	-0.623393000
S	-0.507754000	-0.392293000	-2.166872000	C	3.426018000	-2.102695000	-1.589648000
C	1.529245000	-1.388961000	0.216446000	C	3.145755000	-0.773995000	-1.275596000
O	1.312743000	-2.315682000	1.033877000	C	2.386175000	1.012223000	0.364638000
C	2.264158000	-1.882725000	-1.050160000	C	1.266899000	1.699549000	-0.137927000
C	1.647261000	-2.880030000	-1.818454000	C	1.061410000	3.023821000	0.267295000
C	3.584881000	-1.550694000	-1.372382000	C	1.921975000	3.659038000	1.157446000
C	2.290363000	-3.479973000	-2.894525000	C	3.020515000	2.967927000	1.664245000
H	0.638056000	-3.199743000	-1.558344000	C	3.246441000	1.654852000	1.263579000
C	4.240016000	-2.152795000	-2.449748000	H	2.150567000	-1.142456000	1.936876000
H	4.133700000	-0.821577000	-0.779132000	H	2.660413000	-3.513000000	1.412055000
C	3.595936000	-3.112471000	-3.222832000	H	3.471554000	-4.130929000	-0.871040000
H	1.771890000	-4.245844000	-3.476269000	H	3.782711000	-2.351275000	-2.589996000
H	5.270287000	-1.866715000	-2.673966000	H	0.199273000	3.569291000	-0.128300000
H	4.108391000	-3.581202000	-4.065972000	H	1.736717000	4.694271000	1.452332000
C	-1.485792000	-1.741963000	1.341088000	H	3.705364000	3.452177000	2.363471000
C	-1.635424000	-0.872595000	2.432137000	H	4.112035000	1.108209000	1.645245000
C	-1.468086000	-3.139511000	1.485579000	Cl	3.391265000	0.437314000	-2.507342000
C	-1.718693000	-1.442800000	3.706379000	C	0.248875000	1.048415000	-1.042867000
C	-1.563497000	-3.656044000	2.777887000	H	0.020951000	1.718832000	-1.885068000
C	-1.675511000	-2.819117000	3.881654000	H	0.633077000	0.122691000	-1.488268000
H	-1.827688000	-0.792058000	4.576705000	C	-1.062904000	0.733436000	-0.299296000
H	-1.538057000	-4.738019000	2.923449000	H	-1.432939000	1.667124000	0.155897000
H	-1.737464000	-3.244227000	4.885667000	C	-2.135092000	0.249394000	-1.275860000
C	-1.371049000	-4.095709000	0.307736000	C	-3.567703000	0.209472000	-0.822187000
H	-1.228701000	-3.497075000	-0.604190000	C	-4.527513000	-0.218233000	-1.748773000
C	-1.777852000	0.633954000	2.288172000	C	-3.978478000	0.579087000	0.464944000
H	-1.585865000	0.897920000	1.238668000	C	-5.872212000	-0.272634000	-1.399248000
C	-3.212637000	1.074697000	2.602921000	H	-4.189193000	-0.503668000	-2.746440000
H	-3.325400000	2.156302000	2.435042000	C	-5.325609000	0.523288000	0.815140000
H	-3.947793000	0.553544000	1.974512000	H	-3.249953000	0.898210000	1.211342000
H	-3.467306000	0.868142000	3.654431000	C	-6.273313000	0.098964000	-0.115103000
C	-0.782090000	1.404028000	3.163226000	H	-6.612641000	-0.606201000	-2.129347000
H	-1.048834000	1.330981000	4.229160000	H	-5.635906000	0.809672000	1.822052000
H	0.240118000	1.019142000	3.052154000	H	-7.328999000	0.055957000	0.162257000
H	-0.779965000	2.470678000	2.893111000	O	-1.837277000	-0.101782000	-2.395805000
C	-0.163875000	-5.031377000	0.430905000	C	-0.862256000	-0.276095000	0.819648000
H	-0.288871000	-5.735325000	1.269129000	C	-0.614954000	0.155993000	2.127448000
H	-0.051978000	-5.629407000	-0.487346000	C	-0.885178000	-1.649919000	0.558874000
H	0.745854000	-4.437348000	0.594695000	C	-0.414011000	-0.763648000	3.155069000
C	-2.675531000	-4.883702000	0.133506000	H	-0.572897000	1.227813000	2.339453000
H	-2.862533000	-5.536424000	1.000849000	C	-0.691370000	-2.571339000	1.585735000
H	-3.545660000	-4.219393000	0.025836000	H	-1.053819000	-2.006145000	-0.460678000
H	-2.621121000	-5.524808000	-0.759874000	C	-0.458630000	-2.131945000	2.887758000
C	-3.917631000	-1.321157000	-0.285634000	H	-0.223535000	-0.408524000	4.170276000
C	-4.928416000	-0.195074000	-0.534239000	H	-0.718175000	-3.640344000	1.364138000
C	-5.382974000	-0.024249000	-1.984902000	H	-0.309651000	-2.854051000	3.693564000
C	-3.140389000	-0.384714000	-3.219570000				
C	-4.337073000	0.532253000	-2.951145000				

H	-4.288830000	-2.249213000	-0.754937000
H	-4.508564000	0.756467000	-0.168427000
H	-5.751323000	-0.994142000	-2.366213000
H	-3.508307000	-1.366302000	-3.569114000
H	-3.857236000	-1.533244000	0.790562000
H	-5.815885000	-0.404627000	0.083686000
H	-6.251924000	0.652937000	-1.995169000
H	-2.551327000	0.029070000	-4.051763000
H	-4.827625000	0.730491000	-3.917398000
H	-3.975155000	1.506641000	-2.584349000
C	1.916477000	-0.022468000	0.915298000
C	1.314353000	1.325323000	0.485151000
C	3.388053000	0.032528000	1.307457000
C	1.547929000	2.026303000	-0.834696000
H	1.576337000	2.037413000	1.285412000
H	0.224192000	1.226391000	0.575221000
C	3.949197000	-1.045812000	2.010335000
C	4.208459000	1.128871000	1.017733000
C	0.916580000	3.273182000	-1.031688000
C	2.311197000	1.510012000	-1.881552000
C	5.289241000	-1.036082000	2.382692000
H	3.313893000	-1.905044000	2.234874000
C	5.553393000	1.139627000	1.393089000
H	3.809805000	1.985838000	0.472205000
C	0.124135000	3.918111000	0.059004000
C	1.047333000	3.947222000	-2.247620000
C	2.441122000	2.185294000	-3.094169000
H	2.808998000	0.552845000	-1.756974000
C	6.101811000	0.056013000	2.072884000
H	5.704729000	-1.891279000	2.921017000
H	6.174044000	2.004641000	1.147144000
C	0.761688000	4.744599000	0.993791000
C	-1.260810000	3.759850000	0.186515000
C	1.804872000	3.406931000	-3.284878000
H	0.545206000	4.909183000	-2.377143000
H	3.043277000	1.743310000	-3.890970000
H	7.154604000	0.062273000	2.364268000
C	0.051454000	5.390824000	2.001730000
H	1.843614000	4.874452000	0.915095000
C	-1.991814000	4.414673000	1.176467000
Cl	-2.113785000	2.695652000	-0.906057000
H	1.898467000	3.942362000	-4.231997000
C	-1.330689000	5.231670000	2.088555000
H	0.577918000	6.025876000	2.716613000
H	-3.072839000	4.277412000	1.225224000
H	-1.899255000	5.741530000	2.868675000
H	1.395173000	-0.213841000	1.866441000

D+1a''•

C	4.365301000	-0.316493000	0.199989000
C	5.019838000	0.787565000	-0.294583000
C	2.500928000	0.994806000	-0.392286000
N	2.967886000	-0.203776000	0.144849000
S	3.874057000	2.002175000	-0.839174000
C	1.124851000	1.302331000	-0.682207000
O	0.220599000	0.411654000	-0.608727000
C	0.764615000	2.686621000	-1.136323000
C	1.330715000	3.864664000	-0.592817000

D+1a'•

C	4.201885000	-0.433655000	0.094390000
C	4.891391000	0.576597000	-0.533520000
C	2.404407000	1.019614000	-0.365552000
N	2.822893000	-0.191268000	0.186932000
S	3.803845000	1.869440000	-1.016815000
C	1.032124000	1.435506000	-0.519587000
O	0.071310000	0.643582000	-0.283120000
C	0.738955000	2.820666000	-1.023768000
C	1.483943000	3.969536000	-0.665109000
C	-0.399876000	2.992041000	-1.848278000

C	-0.253832000	2.819500000	-2.111601000	C	1.117023000	5.240251000	-1.137138000
C	0.907797000	5.130758000	-1.028137000	H	2.340499000	3.882728000	0.021222000
H	2.085084000	3.797731000	0.206287000	C	-0.757461000	4.258223000	-2.329159000
C	-0.665621000	4.083337000	-2.554242000	H	-0.999208000	2.105224000	-2.103480000
H	-0.713490000	1.907456000	-2.521435000	C	0.001795000	5.390069000	-1.978497000
C	-0.084984000	5.246413000	-2.015803000	H	1.702463000	6.122651000	-0.833867000
H	1.350699000	6.034999000	-0.581634000	H	-1.637533000	4.366433000	-2.983014000
H	-1.447600000	4.164483000	-3.325873000	H	-0.281807000	6.386506000	-2.351831000
H	-0.412179000	6.240136000	-2.359417000	C	1.944379000	-1.056636000	0.949605000
C	2.097161000	-1.180177000	0.771500000	C	1.387417000	-2.202592000	0.325298000
C	1.693618000	-2.323678000	0.035366000	C	1.722081000	-0.752754000	2.317232000
C	1.726914000	-0.977880000	2.125879000	C	0.572117000	-3.041824000	1.108953000
C	0.887484000	-3.272457000	0.693573000	C	0.892448000	-1.623333000	3.053484000
C	0.912632000	-1.955820000	2.733376000	C	0.320695000	-2.755040000	2.458164000
C	0.495861000	-3.091605000	2.027576000	H	0.119253000	-3.934818000	0.650565000
H	0.554877000	-4.168357000	0.146246000	H	0.696167000	-1.410008000	4.115992000
H	0.604598000	-1.823501000	3.782434000	H	-0.322748000	-3.423280000	3.051909000
H	-0.137507000	-3.844943000	2.521867000	C	2.331308000	0.470295000	2.998356000
C	2.168041000	0.243856000	2.928612000	H	2.997421000	0.971387000	2.267496000
H	2.832250000	0.854930000	2.285062000	C	1.612072000	-2.517987000	-1.150310000
C	2.075466000	-2.532414000	-1.426634000	H	2.363776000	-1.799039000	-1.535588000
H	2.756040000	-1.705869000	-1.716562000	C	2.171359000	-3.934298000	-1.366589000
C	2.830694000	-3.855202000	-1.643248000	H	2.401637000	-4.098067000	-2.439979000
H	3.162174000	-3.942646000	-2.698965000	H	3.103949000	-4.104423000	-0.790638000
H	3.730256000	-3.929115000	-0.998722000	H	1.441396000	-4.714380000	-1.064477000
H	2.186588000	-4.733034000	-1.425509000	C	0.316297000	-2.300551000	-1.954953000
C	0.834710000	-2.445348000	-2.335589000	H	-0.469474000	-3.023908000	-1.649836000
H	0.129524000	-3.279260000	-2.132669000	H	-0.075944000	-1.277122000	-1.790581000
H	0.298562000	-1.489346000	-2.171911000	H	0.501293000	-2.444204000	-3.040305000
H	1.129428000	-2.509605000	-3.404039000	C	1.243844000	1.485233000	3.396178000
C	0.965614000	1.126530000	3.310368000	H	0.556130000	1.062753000	4.158976000
H	0.268229000	0.590728000	3.988304000	H	1.702877000	2.398612000	3.828887000
H	1.307385000	2.041518000	3.837723000	H	0.633885000	1.790267000	2.521765000
H	0.391432000	1.442900000	2.415655000	C	3.196899000	0.078943000	4.208939000
C	2.981470000	-0.156151000	4.172393000	H	2.592298000	-0.394702000	5.010535000
H	2.370395000	-0.740373000	4.891915000	H	3.999510000	-0.632524000	3.924650000
H	3.865606000	-0.769831000	3.902652000	H	3.680056000	0.977793000	4.645490000
H	3.345601000	0.747901000	4.703459000	C	4.827573000	-1.672614000	0.675010000
C	5.037649000	-1.527049000	0.787825000	C	5.744528000	-2.454640000	-0.284035000
C	6.129125000	-2.162935000	-0.093237000	C	7.086545000	-1.792485000	-0.624448000
C	7.430365000	-1.362385000	-0.238897000	C	6.369983000	0.698162000	-0.772660000
C	6.496876000	1.057190000	-0.353619000	C	7.018167000	-0.512366000	-1.466924000
C	7.334136000	-0.047029000	-1.022244000	H	5.411637000	-1.382860000	1.578289000
H	5.483960000	-1.241419000	1.767681000	H	5.188258000	-2.680232000	-1.220265000
H	5.706758000	-2.381101000	-1.098599000	H	7.633398000	-1.575028000	0.321500000
H	7.841563000	-1.151661000	0.774820000	H	6.882367000	0.875483000	0.201495000
H	6.875946000	1.223677000	0.681468000	H	4.024572000	-2.342070000	1.037287000
H	4.266704000	-2.286885000	1.017149000	H	5.953920000	-3.435078000	0.193784000
H	6.376855000	-3.147325000	0.357199000	H	7.708679000	-2.534191000	-1.170037000
H	8.180120000	-2.010972000	-0.740855000	H	6.558320000	1.607554000	-1.379245000
H	6.662679000	2.013474000	-0.890644000	H	8.052933000	-0.221200000	-1.746686000
H	8.360579000	0.353384000	-1.162770000	H	6.481863000	-0.715422000	-2.420096000
H	6.932245000	-0.242094000	-2.040939000	C	-5.768860000	-0.291147000	0.167876000
C	-5.672886000	-0.265578000	0.348777000	C	-7.136432000	-0.244136000	0.543869000
C	-6.991459000	-0.125090000	0.855682000	C	-7.623919000	0.676332000	1.477659000
C	-7.325037000	0.844822000	1.806476000	C	-6.748792000	1.596405000	2.075768000
C	-6.340440000	1.721112000	2.290722000	C	-5.390826000	1.587556000	1.724865000
C	-5.026848000	1.614507000	1.809969000	C	-4.917642000	0.660883000	0.784261000
C	-4.703915000	0.637967000	0.855083000	C	-5.302093000	-1.298129000	-0.820508000
C	-5.345369000	-1.309741000	-0.658024000	C	-4.148474000	-1.130965000	-1.588896000

C	-4.058457000	-1.421452000	-1.193418000
C	-3.652335000	-2.357605000	-2.132623000
C	-4.614274000	-3.280400000	-2.596113000
C	-5.927378000	-3.219013000	-2.093056000
C	-6.293136000	-2.255983000	-1.141537000
H	-7.780767000	-0.797045000	0.488580000
H	-8.361572000	0.916572000	2.170060000
H	-6.587453000	2.489498000	3.039410000
H	-4.248200000	2.298307000	2.180933000
H	-2.615506000	-2.390645000	-2.511124000
H	-4.338400000	-4.042267000	-3.343378000
H	-6.682633000	-3.937466000	-2.447772000
H	-7.329728000	-2.246894000	-0.770635000
I	-2.638893000	0.593762000	0.195578000

C	-3.652102000	-2.013515000	-2.532383000
C	-4.383310000	-3.202682000	-2.749885000
C	-5.553865000	-3.440067000	-2.007129000
C	-6.009097000	-2.511529000	-1.059410000
H	-7.839000000	-0.943263000	0.066654000
H	-8.694567000	0.679003000	1.732608000
H	-7.115644000	2.328131000	2.811712000
H	-4.697588000	2.305974000	2.186643000
H	-2.728298000	-1.809065000	-3.099711000
H	-4.032473000	-3.937957000	-3.492046000
H	-6.119734000	-4.371747000	-2.161654000
H	-6.916270000	-2.739597000	-0.477734000
Br	-3.000269000	0.734933000	0.376059000

13

C	0.388499000	0.034335000	0.247604000
O	-0.834587000	0.007363000	0.115981000
C	1.234340000	0.950327000	-0.589212000
C	0.576300000	1.948016000	-1.342647000
C	2.634194000	0.810462000	-0.703211000
C	1.304888000	2.800750000	-2.178352000
H	-0.517673000	2.023963000	-1.251246000
C	3.362229000	1.661390000	-1.547411000
H	3.158654000	0.029380000	-0.133320000
C	2.700982000	2.658789000	-2.282300000
H	0.786109000	3.580512000	-2.757165000
H	4.453776000	1.545279000	-1.628549000
H	3.274940000	3.328126000	-2.941929000
C	1.047480000	-0.933536000	1.207338000
C	1.928770000	-0.541342000	2.251158000
C	0.705074000	-2.294759000	1.058458000
C	2.187133000	0.896997000	2.551342000
C	2.474735000	-1.541491000	3.086464000
C	1.264249000	-3.272967000	1.888051000
H	-0.007306000	-2.571179000	0.266612000
C	1.094091000	1.759808000	2.801393000
C	3.483594000	1.461004000	2.635009000
C	2.157457000	-2.893068000	2.903425000
H	3.144740000	-1.242300000	3.905927000
H	0.999244000	-4.332000000	1.747568000
C	1.276910000	3.111812000	3.118944000
H	0.077736000	1.339863000	2.755084000
C	3.679800000	2.814505000	2.952329000
Cl	4.904139000	0.494171000	2.280178000
H	2.596699000	-3.651901000	3.568953000
C	2.574138000	3.640402000	3.197541000
H	0.402553000	3.751538000	3.310349000
H	4.704495000	3.210165000	2.993865000
H	2.732732000	4.700521000	3.446823000

TS3'

C	2.551358000	1.312952000	-0.129500000
C	1.889599000	2.510602000	0.019789000
C	0.388889000	0.481871000	0.377954000
N	1.689026000	0.209488000	0.095828000
S	0.217287000	2.190141000	0.413293000
C	-0.947230000	-0.809135000	1.334839000
O	-0.204043000	-1.485889000	2.099494000
C	-1.811352000	0.223039000	2.079948000
C	-1.196351000	1.043191000	3.054831000
C	-3.216331000	0.226581000	2.009322000
C	-1.955304000	1.882360000	3.880674000
H	-0.102900000	1.004668000	3.171593000
C	-3.985577000	1.060195000	2.842985000
H	-3.733430000	-0.448514000	1.315022000
C	-3.359233000	1.901698000	3.772923000
H	-1.450375000	2.522017000	4.622102000
H	-5.084035000	1.042336000	2.759467000
H	-3.958014000	2.559498000	4.422416000
C	2.179468000	-1.164578000	0.053233000
C	2.072437000	-1.888863000	-1.162664000
C	2.769835000	-1.716569000	1.222768000
C	2.502143000	-3.232307000	-1.160752000
C	3.188815000	-3.060743000	1.159949000
C	3.040783000	-3.818359000	-0.009152000
H	2.425310000	-3.822732000	-2.086775000
H	3.644195000	-3.521693000	2.050118000
H	3.367934000	-4.869813000	-0.028253000
C	3.039049000	-0.913107000	2.495396000
H	2.566409000	0.083331000	2.376442000
C	1.609033000	-1.253863000	-2.472478000
H	1.248034000	-0.229736000	-2.246850000
C	2.792719000	-1.125716000	-3.451825000
H	2.482619000	-0.574907000	-4.364330000
H	3.649642000	-0.585763000	-3.002560000
H	3.155229000	-2.125377000	-3.771557000
C	0.451258000	-2.018917000	-3.131923000
H	0.761249000	-3.037141000	-3.447501000
H	-0.414822000	-2.122370000	-2.451303000
H	0.107859000	-1.483670000	-4.041500000
C	2.435895000	-1.560080000	3.753983000
H	2.890343000	-2.552158000	3.960609000

H	2.634777000	-0.920444000	4.639629000
H	1.342908000	-1.679038000	3.635564000
C	4.554606000	-0.697334000	2.684461000
H	5.072827000	-1.660751000	2.875544000
H	5.029927000	-0.230982000	1.799176000
H	4.744096000	-0.039085000	3.558059000
C	3.999623000	1.148694000	-0.494230000
C	4.457441000	1.954310000	-1.725172000
C	4.537423000	3.476573000	-1.544992000
C	2.436528000	3.906620000	-0.082526000
C	3.200311000	4.210812000	-1.382798000
H	4.623629000	1.444303000	0.378885000
H	3.798289000	1.714109000	-2.587746000
H	5.187235000	3.706501000	-0.669796000
H	3.110549000	4.088721000	0.785850000
H	4.204714000	0.073789000	-0.663510000
H	5.468352000	1.583576000	-1.996909000
H	5.059702000	3.900739000	-2.429283000
H	1.600064000	4.626407000	0.028088000
H	3.393690000	5.304184000	-1.405650000
H	2.541640000	3.991969000	-2.252194000
C	-1.617934000	-1.602572000	0.159938000
C	-2.504662000	-1.173663000	-0.878876000
C	-1.348227000	-2.989520000	0.244753000
C	-2.854413000	0.238577000	-1.252025000
C	-3.082248000	-2.148659000	-1.732111000
C	-1.913723000	-3.937732000	-0.614846000
H	-0.663549000	-3.293381000	1.050397000
C	-1.890008000	1.059488000	-1.878946000
C	-4.175279000	0.753651000	-1.194218000
C	-2.804544000	-3.515044000	-1.611221000
H	-3.761891000	-1.807409000	-2.528343000
H	-1.664592000	-5.004667000	-0.499923000
C	-2.199054000	2.336505000	-2.368556000
H	-0.874083000	0.659387000	-2.003001000
C	-4.498975000	2.037600000	-1.662227000
Cl	-5.498920000	-0.217633000	-0.555895000
H	-3.273369000	-4.236462000	-2.298479000
C	-3.504367000	2.834939000	-2.246084000
H	-1.415088000	2.940473000	-2.850326000
H	-5.534137000	2.398776000	-1.577621000
H	-3.761134000	3.838534000	-2.618533000

TS2'

C	-1.671573000	0.782283000	-1.172594000
C	-0.695740000	1.207802000	-2.017397000
C	0.034680000	-0.652377000	-0.538204000
N	-1.236937000	-0.269436000	-0.342757000
S	0.740764000	0.252604000	-1.808783000
C	0.716114000	-1.845793000	0.051450000
O	0.106758000	-2.680349000	0.723220000
C	1.962626000	-2.288489000	-0.700894000
C	1.747661000	-2.871636000	-1.959022000
C	3.253648000	-2.279720000	-0.168790000
C	2.808506000	-3.413902000	-2.682444000
H	0.736673000	-2.898388000	-2.377007000
C	4.315562000	-2.826094000	-0.892070000
H	3.429060000	-1.830323000	0.807985000

D-1a

C	-2.056571000	1.153957000	-0.780065000
C	-1.206786000	1.890524000	-1.576520000
C	-0.114650000	-0.134093000	-0.566131000
N	-1.428102000	0.011126000	-0.238086000
S	0.360419000	1.137930000	-1.620163000
C	0.787966000	-1.371269000	-0.244441000
O	0.025480000	-2.462377000	-0.251237000
C	1.852254000	-1.535722000	-1.396751000
C	1.399111000	-1.622370000	-2.737103000
C	3.201316000	-1.856657000	-1.144911000
C	2.264883000	-1.977875000	-3.780189000
H	0.340405000	-1.429142000	-2.969107000
C	4.071380000	-2.227210000	-2.187565000

C	4.098269000	-3.389697000	-2.149040000	H	3.589791000	-1.820457000	-0.118197000
H	2.626673000	-3.858955000	-3.662384000	C	3.613140000	-2.281853000	-3.511638000
H	5.321832000	-2.806612000	-0.469040000	H	1.882317000	-2.027374000	-4.812396000
H	4.932273000	-3.812286000	-2.712424000	H	5.121438000	-2.468296000	-1.955831000
C	-2.152162000	-0.960766000	0.545255000	H	4.294883000	-2.564976000	-4.329007000
C	-2.464088000	-0.391435000	1.790168000	C	-2.213945000	-0.994285000	0.481942000
C	-2.741182000	-2.149233000	0.079505000	C	-2.402572000	-0.868893000	1.880646000
C	-3.381226000	-1.075726000	2.598288000	C	-2.862418000	-1.998326000	-0.289583000
C	-3.655014000	-2.788285000	0.920770000	C	-3.195539000	-1.846914000	2.520561000
C	-3.967614000	-2.262608000	2.172816000	C	-3.650532000	-2.934969000	0.403135000
H	-3.646042000	-0.659556000	3.572572000	C	-3.803323000	-2.876639000	1.796269000
H	-4.131269000	-3.713125000	0.588363000	H	-3.348412000	-1.782092000	3.609000000
H	-4.682186000	-2.778443000	2.816783000	H	-4.155621000	-3.731900000	-0.164625000
C	-2.450855000	-2.729654000	-1.295080000	H	-4.418148000	-3.628622000	2.315316000
H	-1.626243000	-2.161231000	-1.749725000	C	-2.764543000	-2.093184000	-1.810766000
C	-1.907890000	0.942574000	2.260399000	H	-2.077288000	-1.297545000	-2.162151000
H	-1.159800000	1.277414000	1.524972000	C	-1.854940000	0.292052000	2.706097000
C	-3.016771000	1.999065000	2.346896000	H	-1.266954000	0.939194000	2.024876000
H	-2.583886000	2.985156000	2.575975000	C	-2.999393000	1.148096000	3.282638000
H	-3.592086000	2.086716000	1.415973000	H	-2.590907000	2.046416000	3.790904000
H	-3.720945000	1.746329000	3.155227000	H	-3.701815000	1.491181000	2.497454000
C	-1.216548000	0.829881000	3.621046000	H	-3.586084000	0.579310000	4.034307000
H	-1.941138000	0.562886000	4.406231000	C	-0.920713000	-0.175815000	3.834572000
H	-0.420339000	0.074207000	3.612734000	H	-1.470559000	-0.779506000	4.587022000
H	-0.772712000	1.798762000	3.898630000	H	-0.084776000	-0.792012000	3.450883000
C	-2.006843000	-4.192031000	-1.217844000	H	-0.489179000	0.698027000	4.365546000
H	-2.816410000	-4.835437000	-0.839774000	C	-2.164782000	-3.435746000	-2.263635000
H	-1.737145000	-4.556202000	-2.220987000	H	-2.821333000	-4.289411000	-1.991739000
H	-1.135088000	-4.294271000	-0.557028000	H	-2.046537000	-3.450150000	-3.367909000
C	-3.674310000	-2.573078000	-2.204080000	H	-1.172944000	-3.566138000	-1.787790000
H	-4.528265000	-3.145598000	-1.809289000	C	-4.134872000	-1.847158000	-2.470343000
H	-3.980458000	-1.519548000	-2.287966000	H	-4.864012000	-2.638421000	-2.196120000
H	-3.452230000	-2.948335000	-3.214401000	H	-4.570948000	-0.870881000	-2.175723000
C	-3.100233000	1.240892000	-1.171954000	H	-4.037590000	-1.854826000	-3.576182000
C	-3.291579000	2.759677000	-1.190706000	C	-3.502719000	1.473690000	-0.527327000
C	-2.953637000	3.438733000	-2.516524000	C	-3.782446000	2.920089000	-0.076943000
C	-0.771330000	2.199457000	-3.145198000	C	-3.602388000	4.007789000	-1.144378000
C	-1.463438000	3.526735000	-2.833022000	C	-1.504341000	3.120342000	-2.387217000
H	-3.594129000	0.808430000	-2.059794000	C	-2.163755000	4.269905000	-1.606607000
H	-2.697175000	3.214513000	-0.380108000	H	-4.076091000	1.268067000	-1.459367000
H	-3.477718000	2.917788000	-3.336751000	H	-3.155324000	3.156562000	0.810159000
H	-1.287423000	1.712167000	-3.991271000	H	-4.228854000	3.755686000	-2.030234000
H	-3.614352000	0.813554000	-0.302510000	H	-2.163278000	2.831179000	-3.237755000
H	-4.348870000	2.954623000	-0.955682000	H	-3.896650000	0.771884000	0.231505000
H	-3.356657000	4.462832000	-2.491689000	H	-4.835483000	2.949131000	0.273736000
H	0.252835000	2.407035000	-3.490541000	H	-4.015003000	4.957539000	-0.741735000
H	-1.326904000	4.173490000	-3.712976000	H	-0.560629000	3.481925000	-2.843819000
H	-0.944497000	4.017635000	-1.995866000	H	-2.162872000	5.160664000	-2.269486000
C	1.541067000	-0.345562000	1.666360000	H	-1.526185000	4.529680000	-0.733058000
C	1.909896000	0.986484000	1.908795000	C	1.553704000	-1.164042000	1.149137000
C	1.611341000	-1.254342000	2.718951000	C	2.275148000	-0.044694000	1.658129000
C	1.829109000	2.064336000	0.874265000	C	1.543396000	-2.344808000	1.920343000
C	2.336343000	1.371878000	3.193971000	C	2.338286000	1.329357000	1.052953000
C	2.071552000	-0.886068000	3.987553000	C	2.960612000	-0.175104000	2.892480000
H	1.283023000	-2.287153000	2.540677000	C	2.229362000	-2.465041000	3.135576000
C	0.869589000	3.080730000	1.000561000	H	0.950717000	-3.168049000	1.488702000
C	2.710585000	2.152526000	-0.209816000	C	1.302414000	2.255244000	1.316367000
C	2.432208000	0.440453000	4.227048000	C	3.459843000	1.820901000	0.344239000
H	2.603506000	2.416812000	3.376633000	C	2.952607000	-1.367903000	3.627275000
H	2.136428000	-1.625981000	4.789657000	H	3.510356000	0.697004000	3.281167000

C	0.774819000	4.116606000	0.073447000	H	2.196412000	-3.410890000	3.700135000
H	0.189145000	3.050241000	1.855171000	C	1.345219000	3.576244000	0.851096000
C	2.631289000	3.179869000	-1.149526000	H	0.441531000	1.913821000	1.909464000
Cl	3.937056000	0.934667000	-0.431738000	C	3.509937000	3.135878000	-0.151865000
H	2.781183000	0.752556000	5.213446000	Cl	4.875991000	0.812526000	0.101216000
C	1.655068000	4.163010000	-1.008534000	H	3.499084000	-1.430850000	4.581449000
H	0.014037000	4.889248000	0.198156000	C	2.445250000	4.013086000	0.095554000
H	3.333916000	3.200856000	-1.983528000	H	0.517435000	4.264658000	1.080170000
H	1.588299000	4.967080000	-1.743640000	H	4.395240000	3.467223000	-0.713960000
				H	2.490588000	5.044216000	-0.287357000

References:

1. Gaussian 16, Revision C.01, M. J. Frisch, G. W. Trucks, H. B. Schlegel, G. E. Scuseria, M. A. Robb, J. R. Cheeseman, G. Scalmani, V. Barone, G. A. Petersson, H. Nakatsuji, X. Li, M. Caricato, A. V. Marenich, J. Bloino, B. G. Janesko, R. Gomperts, B. Mennucci, H. P. Hratchian, J. V. Ortiz, A. F. Izmaylov, J. L. Sonnenberg, D. Williams-Young, F. Ding, F. Lipparini, F. Egidi, J. Goings, B. Peng, A. Petrone, T. Henderson, D. Ranasinghe, V. G. Zakrzewski, J. Gao, N. Rega, G. Zheng, W. Liang, M. Hada, M. Ehara, K. Toyota, R. Fukuda, J. Hasegawa, M. Ishida, T. Nakajima, Y. Honda, O. Kitao, H. Nakai, T. Vreven, K. Throssell, J. A. Montgomery, Jr., J. E. Peralta, F. Ogliaro, M. J. Bearpark, J. J. Heyd, E. N. Brothers, K. N. Kudin, V. N. Staroverov, T. A. Keith, R. Kobayashi, J. Normand, K. Raghavachari, A. P. Rendell, J. C. Burant, S. S. Iyengar, J. Tomasi, M. Cossi, J. M. Millam, M. Klene, C. Adamo, R. Cammi, J. W. Ochterski, R. L. Martin, K. Morokuma, O. Farkas, J. B. Foresman, and D. J. Fox, Gaussian, Inc., Wallingford CT, 2019.

2. J.-D. Chai and M. Head-Gordon, Long-range corrected hybrid density functionals with damped atom–atom dispersion corrections. *Phys. Chem. Chem. Phys*. 2008, **10**, 6615-6620.

3. F. Weigend and R. Ahlrichs, Balanced basis sets of split valence, triple zeta valence and quadruple zeta valence quality for H to Rn: Design and assessment of accuracy. *Phys. Chem. Chem. Phys*. 2005, **7**, 3297–3305.

4. A. V. Marenich, C. J. Cramer and D. G. Truhlar, Universal Solvation Model Based on Solute Electron Density and on a Continuum Model of the Solvent Defined by the Bulk Dielectric Constant and Atomic Surface Tensions. *J. Phys. Chem. B.* 2009, **113**, 6378–6396.
